# Distinct SOX9 single-molecule dynamics characterize adult differentiation and fetal-like reprogrammed states in intestinal organoids

**DOI:** 10.1016/j.stemcr.2025.102787

**Published:** 2026-01-22

**Authors:** Nike Walther, Sathvik Anantakrishnan, Gina M. Dailey, Anna C. Maurer, Claudia Cattoglio

**Affiliations:** 1 Department of Molecular and Cell Biology, Li Ka Shing Center for Biomedical and Health Sciences, California Institute for Regenerative Medicine (CIRM) Center of Excellence, University of California, Berkeley, Berkeley, CA 94720, USA; 2 Department of Genetics, University of Bayreuth, 95440 Bayreuth, Germany; 3 Biophysics Graduate Group, University of California, Berkeley, Berkeley, CA 94720, USA; 4 Howard Hughes Medical Institute, Berkeley, CA 94720, USA

**Keywords:** transcription factor, SOX9, mouse small intestinal organoid, 2D enteroid monolayer culture, automated live-cell single-molecule tracking, cellular feature extraction and protein diffusion correlation, cellular protein diffusion and self-association, differentiation, fetal-like reversion, cell state transition, transcription factor dosage

## Abstract

Transcription factors (TFs) mediate gene expression changes during differentiation and development. However, how TF biophysical properties and abundance dynamically regulate specific cell state transitions remains poorly understood. Using automated live-cell single-molecule tracking (SMT) in intestinal organoid models, we revealed an expression-level-independent decrease in the fraction of immobile sex-determining region Y box 9 (SOX9) molecules during differentiation from ∼48% to ∼38%, largely dependent on DNA binding. Strikingly, long-term SOX9 overexpression caused organoids to transition from budding to spheroid morphology accompanied by increased proliferation and a loss in gene expression signatures for intestinal identity and function. In this fetal-like reprogrammed state, a larger fraction of partially self-interacting SOX9 molecules (∼61%) binds to DNA. Our results suggest context-dependent SOX9 single-molecule dynamics during adult intestinal differentiation and fetal-like reversion in consequence to long-term SOX9 overexpression. Our work underpins the power of our automated live-cell SMT framework to generate testable hypotheses toward unraveling molecular mechanisms underlying tissue-level phenotypes.

## Highlights

• Heterogenous diffusion behavior of SOX9 across intestinal differentiation states
• Concentration-independent decrease in chromatin-bound SOX9 upon differentiation
• SOX9 overexpression leads to fetal-like reversion and more immobile SOX9 molecules
• Automated SMT in 2D monolayers reveals TF dynamics underlying organoid phenotypes

## Graphical abstract

**Figure:**
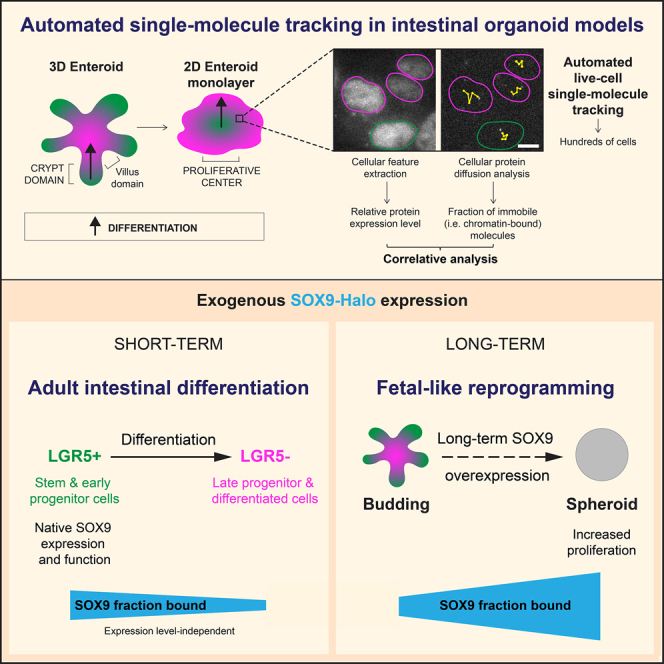

## Teaser

Walther and colleagues employed an automated live-cell single-molecule tracking pipeline to study the diffusive behavior of the transcription factor SOX9 during adult differentiation and fetal-like reprogrammed states in intestinal organoid models. The authors linked distinct fractions of chromatin-bound SOX9 molecules to specific cellular states in enteroid monolayers, thereby paving the way to unravel molecular mechanisms underlying differentiation and organoid phenotypes.

## Introduction

Gene expression programs are rewired during tissue development and homeostasis to ensure the formation and maintenance of healthy organs. This is accomplished by the differentiation of stem cells into various specialized cell types in a highly spatiotemporally controlled manner. One such regulatory layer is provided by lineage-specific transcription factors (TFs) (Weidemüller et al., 2021). Acting downstream of signaling pathways, TFs bind with co-factors to *cis*-regulatory elements of target genes, inducing or repressing their expression (Cramer, 2019). Misexpression of TFs conferring cell fate decisions can lead to cellular reprogramming and is associated with disease, including cancer (Huilgol et al., 2019; Lee and Young, 2013). It is thus crucial that lineage TFs are expressed in the correct tissue place at the correct dose. However, it remains poorly understood how the abundance and biophysical properties of cell fate-determining TFs change during stem cell differentiation to mature cell types. It is further unclear what TF dosage is tolerated for faithful differentiation and tissue formation, and whether TF overexpression alters their molecular dynamics. To address these questions, TF abundance and single-molecule dynamics need to be probed in differentiating multicellular systems, necessitating the choice of a model system that recapitulates *in vivo* differentiation trajectories and is amenable to live-cell single-molecule imaging across scales.

**Figure 1:**
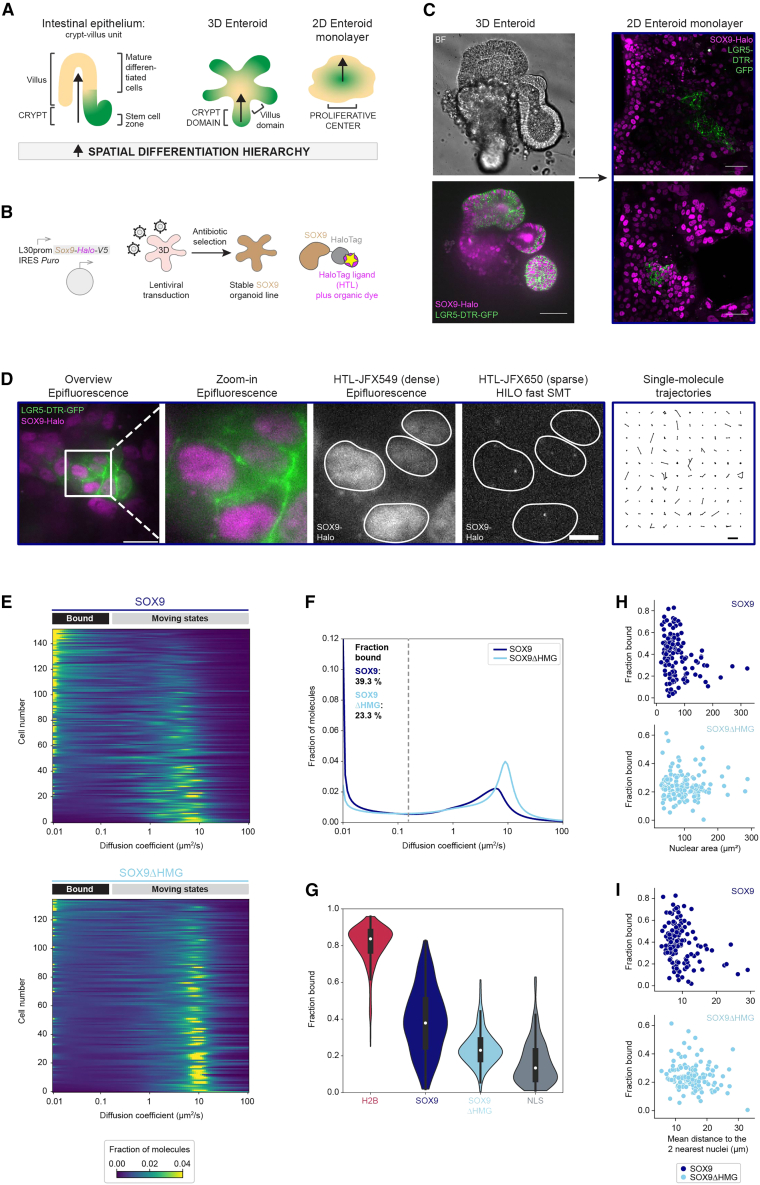
Automated live-cell fast single-molecule tracking in 2D enteroid monolayer cultures reveals a heterogenous diffusive behavior of SOX9-Halo with the fraction of immobile molecules largely depending on DNA binding (A) In the mammalian intestinal epithelium (left), a spatial differentiation hierarchy (arrow) guides directional movement of differentiating intestinal stem cells (ISCs) along the crypt-villus (green-beige) axis, also reflected in *in vitro* models of the intestinal epithelium: in mouse small intestinal organoids (mSIOs; enteroids; middle), ISCs in the crypt domain at bud tips (green) migrate inward upon differentiation, whereas 2D EMCs (right) grow outward from ISC-containing proliferative centers (green). (B) Generation of an organoid line stably expressing a SOX9-HaloTag(Halo)-V5 transgene through lentiviral transduction and antibiotic selection allows fluorescence detection (yellow star) of SOX9-Halo covalently labeled with dye-coupled HaloTag ligands (HTLs). (C) Confocal imaging of mSIOs (left) derived from LGR5::DTR-GFP mice (labeling ISCs/early progenitors [green]) stably expressing SOX9-Halo (magenta) and corresponding 2D EMCs (right) 5 days post-seeding. BF, bright field. Scale bars, 50 μm. (D) Double labeling of SOX9-Halo 2D EMCs with two different HTLs allows bulk labeling for nuclear segmentation (images 1–3 from left; white masks) and sparse labeling for HILO-based fast SMT (image 4; one representative frame of an SMT movie) resulting in single-molecule trajectories (image 5; 100 randomly selected single-molecule trajectories). Scale bars: 20 μm (overview), 5 μm (zoom-in), 1 μm (trajectories). (E) Single-cell diffusion heatmaps for 4 combined automated SMT experiments for SOX9-Halo (dark blue; *n* = 152 cells) and 5 combined manual fast SMT experiments for SOX9ΔHMG-Halo (light blue; *n* = 135 cells). Cells are ordered by decreasing fraction bound (top-bottom). Representative SMT movies in Videos S1 and S2. (F) Mean diffusion spectra for SOX9-Halo (dark blue) and SOX9ΔHMG-Halo (light blue). Bootstrap analysis of combined experiments with *n* = 10, 44, 42, and 56 cells for SOX9 and *n* = 43, 23, 1, 34, and 34 cells for SOX9ΔHMG determined a mean fraction bound (*D* < 0.15 μm^2^/s) of 39.3% (95% confidence interval [CI]: 32.9%–46.7%) and 23.3% (95% CI: 19.5%–28.5%), respectively. (G) Violin plots for fractions bound (white point: median; whiskers: first/third quartile) of Halo-tagged H2B (red), SOX9 (dark blue), SOX9ΔHMG (light blue), and nuclear localization sequence (NLS; gray). SOX9 and SOX9ΔHMG data are the same as in (E) and (F). Data are from 7 H2B and 3 NLS experiments with combined *n* = 367 and *n* = 100 cells (*n* = 65, 30, 12, 50, 123, 81, and 6 and *n* = 12, 47, and 41 cells per experiment). (H and I) Single-cell correlation of SMT-derived fraction bound for SOX9 (top, dark blue) or SOX9ΔHMG (bottom, light blue) with the morphological characteristics nuclear area (H) or mean distance to the two nearest nuclei (I). See also Figure S1.

The mammalian intestine as the fastest renewing organ, which consists of crypts containing intestinal stem cells (ISCs), early progenitor cells, and niche-providing Paneth cells and villi composed of mature cell types, provides such a system: a spatial differentiation hierarchy guides the directional movement of ISCs during differentiation and maturation (Beumer and Clevers, 2016; Gehart and Clevers, 2019) (Figure 1A, left). This feature is recapitulated in *in vitro* tissue models of the gut: in budding structures of 3D mouse small intestinal organoids (mSIOs; enteroids), ISC-containing domains are located at the tips, and differentiating ISCs move inward (Date and Sato, 2015; Sato et al., 2009) (Figure 1A, middle). In 2D enteroid monolayer cultures (EMCs) (Altay et al., 2019; Sanman et al., 2020; Thorne et al., 2018), ISCs in proliferative centers, also containing differentiated Paneth cells, move outward along a radial differentiation trajectory (Thorne et al., 2018) (Figure 1A, right). Such 3D/2D organoid models retain all intestinal epithelial cell types and are amenable to live imaging, which enabled the study of cellular behavior within multicellular tissue-resembling systems (Lukonin et al., 2020; McKinley et al., 2018; Schöneberg et al., 2018; Serra et al., 2019; Tallapragada et al., 2021), including live-cell TF dynamics at the cellular and single-molecule level (Walther et al., 2024).

TFs diffuse in the nucleus in search of their cognate DNA-binding motifs, to which they eventually bind to regulate target gene expression (Cramer, 2019). Diffusion and binding properties of single TF molecules can be measured in live cells by single-molecule tracking (SMT). In contrast to other bulk fluorescence imaging methods (Boka et al., 2021), SMT directly allows the detection, localization, and tracking of individual molecules, enabling the resolution of subpopulations with different diffusive behaviors within the same cell (Dahal et al., 2023). The obtained diffusion spectra provide the following parameters: (1) the diffusion coefficients of differentially mobile subpopulations (e.g., freely, fast, and slowly diffusing) and (2) the fraction of immobile molecules, which, in the case of TFs, are mainly chromatin-bound (fraction bound) (Dahal et al., 2023; Mazzocca et al., 2021).

Highly inclined and laminated optical sheet (HILO)-based SMT has been used extensively to probe TF dynamics in 2D cell culture systems (Chen et al., 2022; Dahal et al., 2025; Ferrie et al., 2024; Hansen et al., 2017; Hsieh et al., 2022; Kuchler et al., 2022; Mazzocca et al., 2023; Szczurek et al., 2024; Zhou et al., 2023), including cancer cells (Chen et al., 2022; Dahal et al., 2025; Ferrie et al., 2024; Mazzocca et al., 2023), embryonic stem cells (Hansen et al., 2017; Hsieh et al., 2022; Szczurek et al., 2024), primary neurons (Zhou et al., 2023), and distinct cell states obtained through directed differentiation protocols (Esbin et al., 2024; Kuchler et al., 2022), but not yet in a cell-type-resolved manner within a multicellular differentiation context. 2D EMCs, as multicellular differentiation systems, are highly heterogenous since they comprise various cell types. To deal with such complexity, we recently developed an automated SMT imaging and analysis pipeline in 2D EMCs that records hundreds of cells and resolves their heterogeneity in TF diffusion correlated with cellular features indicative of differentiation states (Walther et al., 2024). Additionally, we implemented proximity-assisted photoactivation (PAPA)-SMT in 2D EMCs (Walther et al., 2024), a technique probing molecular interactions in live cells at single-molecule resolution (Graham et al., 2022) and thus resolving diffusion parameters of specific molecular complexes (Dahal et al., 2023; 2025; Graham et al., 2025; Walther et al., 2024).

The TF sex-determining region Y (SRY) box 9 (SOX9) exerts important functions in multiple organs (Barrionuevo et al., 2006; Bi et al., 1999; Ming et al., 2022; Poché et al., 2008; Rockich et al., 2013; Seymour et al., 2007; Thomsen et al., 2008; Vidal et al., 2005), including the intestine (Mori-Akiyama et al., 2007), both during embryonic development and adult tissue differentiation. SOX9 mutations and an aberrant SOX9 dosage are implicated in a wide range of diseases, ranging from skeletal dysplasia and sex reversal (Wagner et al., 1994) to colorectal cancer (Abdel-Samad et al., 2011; Prévostel and Blache, 2017).

SOX9 contains a high-mobility group (HMG) box DNA-binding domain (Mertin et al., 1999) and can homodimerize via its dimerization domain (DIM). Dimerization is required for DNA binding and transactivation of cartilage-specific genes (Bernard et al., 2003; Coustry et al., 2010), but it is dispensable in other contexts (Bernard et al., 2003), where SOX9 functions as a monomer.

During mouse intestinal development, SOX9 is expressed in all epithelial cells at E13.5, but it becomes restricted to proliferating progenitor cells at E15.5 (Mori-Akiyama et al., 2007). Upon adulthood, SOX9 is expressed in ISCs, progenitor cells, and secretory Paneth cells (Mori-Akiyama et al., 2007). Here, SOX9 is required for progenitor cell maintenance (Blache et al., 2004) and Paneth cell differentiation (Bastide et al., 2007; Mori-Akiyama et al., 2007), whereby distinct SOX9 expression levels characterize various cell populations within the murine small intestinal crypt (Formeister et al., 2009).

Due to its diverse roles and expression levels during intestinal differentiation and beyond, we chose SOX9 to study how abundance and diffusive behavior of a cell fate-conferring TF change during differentiation. Using our automated SMT pipeline (Walther et al., 2024), we investigated the dynamics of SOX9 under homeostatic conditions in differentiating 2D EMCs. By directly recording cell type markers, we determined an expression-level-independent correlation between the fraction of immobile SOX9 molecules and the progression of differentiation, largely dependent on DNA binding. We further observed that long-term overexpression of SOX9 in mSIOs causes a change in organoid morphology, accompanied by increased proliferation, as well as a loss of intestinal gene expression signatures and the acquisition of a regenerative fetal-like gene expression program. Applying our (PAPA)-SMT pipelines (Walther et al., 2024) to 3D spheroids and spheroid-derived 2D EMCs, we observed increased DNA occupancy by SOX9 and evidence of oligomerization. Our results suggest context-dependent molecular dynamics of SOX9 during differentiation in adult intestinal homeostasis and upon SOX9 overexpression-induced fetal-like reprogramming.

## Results

### Automated fast SMT in live 2D EMCs reveals heterogenous SOX9-Halo diffusion across differentiating cells independent of expression levels

To use SOX9 for interrogating the diffusive behavior of a cell fate-conferring TF in the intestinal differentiation paradigm (Figure 1A), we used lentiviral transgene delivery (Figure 1B) to generate a stable mSIO line expressing SOX9-Halo from a weak ubiquitous L30 promoter (Chen et al., 2022). Nuclear SOX9-Halo expression in 3D mSIOs and 2D EMCs derived thereof was confirmed by live imaging (Figure 1C) after covalent labeling of the HaloTag (Halo) with HaloTag ligands (HTLs) coupled to bright and photostable dyes (Figure 1B) (Grimm et al., 2017), which was also key for SMT (Figure 1D). Here, we used HILO-based live-cell fast SMT on a total internal reflection fluorescence (TIRF) microscope and employed a stroboscopic illumination scheme to reduce motion blur (Dahal et al., 2023; Hansen et al., 2017). As previously observed for a different lineage TF in this system (Walther et al., 2024), a typical manual fast SMT experiment of 10 randomly selected cells did not provide a conclusive picture about the SOX9-Halo diffusion behavior in these heterogenous EMCs (Figures S1A and S1B), demonstrating the need to acquire larger datasets. We thus used our previously developed automated SMT pipeline (Walther et al., 2024) to acquire hundreds of cells (Figures 1D and 1E, top; Figure S1E). Bulk-labeled SOX9-Halo (Figure 1D, subpanels 1–3) allowed the automated detection of nuclei and triggered an SMT sequence in another channel with SOX9-Halo sparsely labeled with a second HTL-coupled fluorophore (Figure 1D, subpanel 4) to obtain typically hundreds to thousands of single-molecule trajectories with an average trajectory length of ∼3–5 localizations (Walther et al., 2024) (Figure 1D, subpanel 5; for tracking statistics, see methods). Automated SMT in hundreds of randomly chosen cells confirmed a large heterogeneity in the diffusive behavior of SOX9-Halo with cellular diffusion peaks ranging from freely diffusing (*D* ∼10 μm^2^/s) to immobile (*D* ∼0.01 μm^2^/s) (Figure 1E, top; Figure S1E) and an average fraction of immobile (*D* < 0.15 μm^2^/s; diffusion behavior indistinguishable from that of histone H2B-Halo (Walther et al., 2024; for details, see methods) SOX9-Halo molecules of 39.3% (95% confidence interval [CI]: 32.9%–46.7%; Figures 1F and 1G). Notably, cell-to-cell differences in the fraction bound covered the whole spectrum between the average fractions bound determined for the immobile H2B-Halo (83.7%; 95% CI: 81.4%–85.9%) and freely diffusing Halo-nuclear localization sequence (NLS; 19.2%; 95% CI: 15.0%–23.2%) controls (CTRLs) (Figures 1G and S1I). On a cell-by-cell basis, the fractions bound of SOX9-Halo did not correlate with the average nuclear SOX9-Halo intensity (Figure S1G), a proxy for transgenic SOX9-Halo levels, arguing against heterogeneity being caused by variable transgene expression in the initially polyclonal organoid line.

### Immobile SOX9-Halo molecules largely reflect DNA binding

A plausible explanation for the observation of immobile states of TFs in the cell nucleus is TF binding to DNA. To test whether this is the case for the immobile SOX9-Halo molecules detected by SMT, we transiently expressed a SOX9-Halo mutant lacking the HMG DNA-binding domain (SOX9ΔHMG-Halo) in wild-type (WT) organoid-derived 2D EMCs via recombinant adeno-associated viral (rAAV) vector delivery (Benyamini et al., 2023) (Figures S1C and S1D). Automated fast SMT of SOX9ΔHMG-Halo revealed a more uniform diffusive behavior (Figure 1E, bottom; Figures 1G and S1F) with a lower average fraction bound of 23.3% (95% CI: 19.5%–28.5%) compared to full-length SOX9-Halo (Figures 1F and 1G; *p* value of fraction bound distribution comparison between SOX9 and SOX9ΔHMG: 7.03e−14) independent of its nuclear expression level (Figure S1H). Hereby, most cells were characterized by a diffusion peak in the freely diffusing range, confirming that the measured immobile fraction largely represents SOX9-Halo molecules bound to DNA. Nevertheless, the average fraction bound of SOX9ΔHMG-Halo did not fully decrease to the 19.2% determined for the Halo-NLS freely diffusing CTRL (Figures 1G and S1I), suggesting that additional SOX9 protein domains might contribute to the bound fraction measured by SMT.

### Cells with more immobile SOX9-Halo molecules display morphological features of intestinal stem/early progenitor cells in 2D EMCs

Hypothesizing that the observed cell-to-cell heterogeneity in the diffusive behavior of SOX9-Halo might reflect differentiation states, we inspected our single-cell diffusion data with respect to nuclear area and the mean distance of a nucleus to its two nearest nuclei, two parameters extracted from our images that we previously found to be correlated with differentiation (Walther et al., 2024). For SOX9-Halo, we indeed identified a subpopulation of cells with smaller nuclei or a smaller nearest-nuclei distance, indicative of stem/early progenitor cells in proliferative centers (Walther et al., 2024) (Figure 1C, right), which was characterized by larger fractions bound (Figure 1H, top). In contrast, a subpopulation of cells with larger nuclei or a larger nearest-nuclei distance, indicative of differentiated cells further away from proliferative centers (Walther et al., 2024) (Figure 1C, right), was characterized by a smaller fraction bound (Figure 1I, top). These subpopulations with distinct diffusive and morphological features were not discernible for the SOX9ΔHMG-Halo mutant (Figures 1H and 1I), suggesting that the immobile fraction of SOX9-Halo in stem/early progenitor cells mainly reflects DNA binding.

### DNA binding of SOX9-Halo decreases upon intestinal differentiation

**Figure 2:**
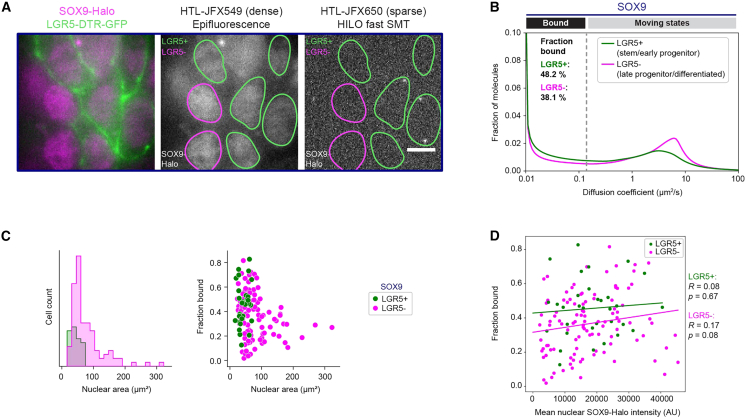
LGR5-based distinction of differentiation states demonstrates an expression-level-independent decrease in the fraction of immobile SOX9-Halo molecules upon differentiation (A) The green fluorescent LGR5-DTR-GFP marker enables classification into stem/early progenitor (LGR5^+^) and late progenitor/differentiated cells (LGR5^−^) for SMT in 2D EMCs. Left: epifluorescence of bulk-labeled SOX9-Halo (magenta) and LGR5-DTR-GFP (green); middle: epifluorescence of bulk-labeled SOX9-Halo (gray) with segmented nuclear masks color-coded according to classification into LGR5^+/−^ (green/magenta) categories; right: representative frame of a fast SMT movie of sparsely labeled SOX9-Halo (gray) with same classified masks depicted. Scale bars: 5 μm. Representative SMT movie in Video S3. (B) Mean diffusion spectra of SOX9-Halo for LGR5^+/−^ (green/magenta) subpopulations. Bootstrap analysis of 4 combined experiments with *n* = 3, 21, 2, and 10 LGR5^+^ and *n* = 7, 23, 40, and 46 LGR5^−^ cells determined a mean fraction bound of 48.2% (95% CI: 44.2%–52.3%) and 38.1% (95% CI: 31.9%–44.5%), respectively. Comparison of the fraction bound distributions between LGR5^+^ and LGR5^−^ cells yielded a *p* value of 0.0554. (C) Left: nuclear area distribution of LGR5^+/−^ (green/magenta) cells extracted from SMT data. Right: single-cell correlation of SMT-derived fraction bound with nuclear area for LGR5^+/−^ cells. (D) Fractions bound for each cell plotted against the mean nuclear SOX9-Halo intensity for LGR5^+/−^ cells. Correlations (fitted lines) were computed for each subpopulation; Pearson correlation coefficients (*R*) and *p* values are indicated. The data in (B)–(D) are the same as in Figures 1E–1I, 5C, S1E, S1G, S1I, and S2A–S2G. See also Figure S2.

To directly test our observation of distinct diffusive subpopulations with morphological characteristics indicative of differentiation states, we implemented the recording of fluorescent cell type markers and marker-based cell classification in our SMT pipeline (Walther et al., 2024). Using the green fluorescent ISC/early progenitor marker LGR5, present in our SOX9-Halo organoid line derived from LGR5::DTR-GFP mice (Tian et al., 2011), we classified the SOX9-Halo population into LGR5-positive (LGR5^+^) stem/early progenitor cells and LGR5-negative (LGR5^−^) late progenitor/differentiated cells (Figure 2A). The average fraction bound of SOX9-Halo decreased during differentiation from 48.2% (95% CI: 44.2%–52.3%) in stem/early progenitor cells to 38.1% (95% CI: 31.9%–44.5%) in late progenitor/differentiated cells (Figures 2B and 2C, right; Figures S2A, S2B, and S2C, bottom; *p* value of SOX9 fraction bound distribution comparison between LGR5^+/−^: 0.055). Such decrease was also apparent upon hierarchical clustering of LGR5^+/−^ single-cell diffusion spectra together (Walther et al., 2024) (Figures S2D–S2G). Notably, cell-to-cell variability persisted within both LGR5^+/−^ subpopulations (Figure 2C, right; Figures S2A, S2B, and S2C, bottom), indicating a potentially more complex grading of the SOX9 diffusion behavior into cell states and types within these heterogenous subpopulations. As observed for the whole population (Figure S1G), the differences in the diffusive behavior of SOX9-Halo were independent of its expression level in both LGR5^+/−^ subpopulations (Figure 2D). Importantly, for both H2B-Halo and Halo-NLS immobile and freely diffusing CTRLs, respectively, no difference in the diffusive behavior was measured between LGR5^+/−^ subpopulations (Figures S2H and S2I; *p* values of fraction bound distribution comparisons between LGR5^+/−^: H2B, 0.125; NLS, 0.914), further supporting that the observed difference in the SOX9-Halo diffusion behavior stems from differentiation and not from other differences between stem and differentiated cells, such as nuclear size or crowding. Notably, the LGR5^+^ subpopulation was characterized by smaller nuclei and a smaller nearest-nuclei distance in comparison to the LGR5^−^ subpopulation (Figure 2C, left; Figure S2C, top), further validating our cell morphology-based approach for an approximated discrimination of undifferentiated from differentiated cells in 2D EMCs (Walther et al., 2024) (Figures 1H and 1I). Nevertheless, we noted some LGR5^−^ cells with smaller nuclei and larger fractions bound (Figure 2C), for which one possible explanation could be differentiated Paneth cells residing within proliferative centers. Taken together, consistent with SOX9 functioning and natively being expressed in the proliferative intestinal crypt, our results suggest that the LGR5^+^ subpopulation is characterized by more SOX9-Halo molecules bound to DNA, possibly resulting from the occupancy of more DNA-binding sites.

### Long-term overexpression of SOX9-Halo results in a proliferative cell state transition accompanied by an organoid morphology change to spheroids devoid of signatures for intestinal identity and function

**Figure 3:**
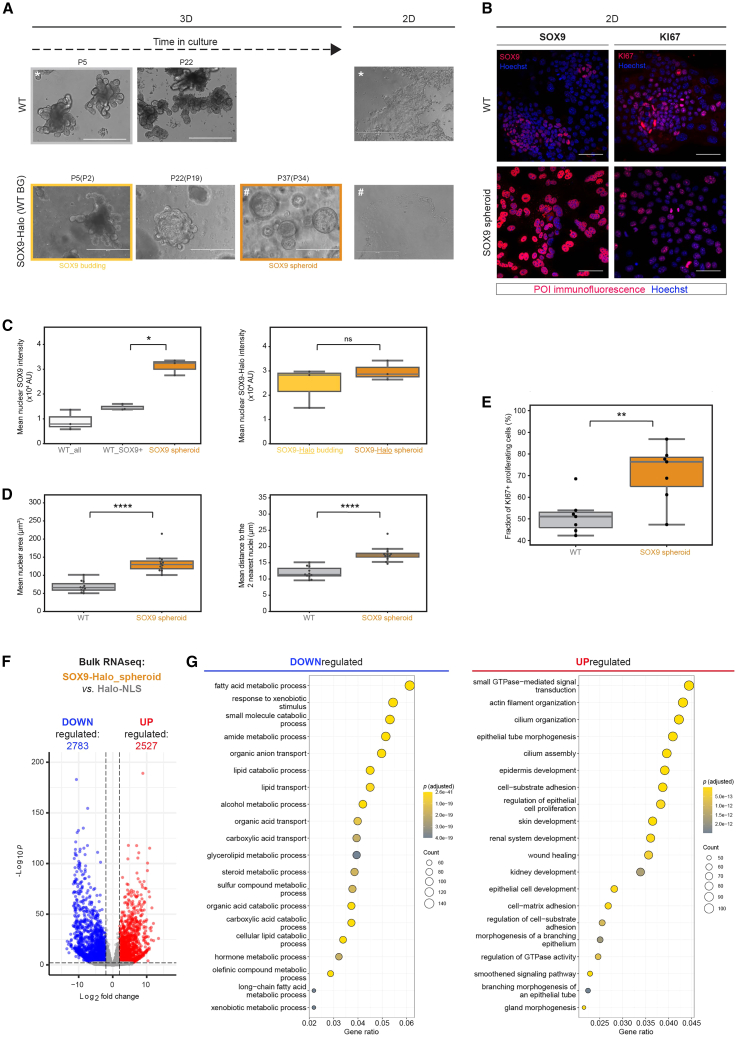
Long-term overexpression of SOX9-Halo in enteroids results in an organoid morphology change coinciding with a proliferative cell state transition and a loss of gene expression signatures for intestinal identity and function (A) In contrast to wild-type (WT) organoids (top), stable SOX9-Halo organoids (WT background; bottom) transitioned from budding to spheroid morphology upon long-term culture. This morphology change of 3D organoids (left) is also reflected in 2D EMCs derived from them (right). Epifluorescence images 5 days post-seeding with organoid passage number P(P) after crypt isolation (after lentiviral transduction to make stable line) indicated. ^∗^/# denotes the passage of 2D EMC derivation from 3D organoids. Scale bars: 400 μm. (B) Confocal images of 2D EMCs derived from WT organoids (top) or SOX9-Halo spheroids (bottom) immunostained (red) for SOX9 (left) or the proliferation marker KI67 (right) and co-stained with Hoechst (blue) 5 days post-seeding. Scale bars: 50 μm. (C) Quantification of total (left) or transgene (right) SOX9 protein levels in 2D EMCs derived from WT (gray), SOX9-Halo_budding (yellow), or SOX9-Halo_spheroid (orange) organoids based on fixed and immunostained (left) or live and HTL-stained (right) confocal images 5 days post-seeding. Total SOX9 levels: 3 FOVs with 613 cells (309 SOX9^+^, 304 SOX9^−^) (WT), 3 FOVs with 337 cells (SOX9_spheroid). Transgene SOX9-Halo levels: 3 FOVs with 864 cells (SOX9_budding), 3 FOVs with 537 cells (SOX9_spheroid). (D) Quantification of nuclear area (left) and mean distance to the two nearest nuclei (right) in 2D EMCs derived from WT organoids (gray) or SOX9-Halo spheroids (orange) 5 days post-seeding based on confocal images (WT: 13 FOVs, 1,821 cells; SOX9_spheroid: 16 FOVs, 1,681 cells). (E) Percentage of KI67^+^ proliferative cells in 2D EMCs derived from WT organoids (gray) or SOX9 spheroids (orange) quantified from confocal images as in (B). WT: 7 FOVs, 752 cells; SOX9_spheroid: 7 FOVs, 648 cells. For (C)–(E), each point represents one FOV; median: gray line, first/third quartile: whiskers; statistical testing based on Mann-Whitney U tests (see methods for details); ns, non-significant, *p* > 0.5; ^∗^*p* ≤ 0.5; ^∗∗^*p* ≤ 0.01; ^∗∗∗∗^*p* ≤ 0.0001. (F and G) Bulk RNA-seq experiment of SOX9-Halo spheroids versus Halo-NLS control (CTRL) organoids in biological triplicates. (F) Volcano plot displaying differentially expressed genes (DEGs; adjusted *p* value ≤ 0.01, fold change ≥2 and mean counts ≥10; red/blue: up-/downregulated). (G) Gene ontology (GO) analysis of the top 20 biological pathways enriched in DEGs down- (left, blue) or upregulated (right, red) with adjusted *p* values and gene counts indicated. Bulk RNA-seq data are the same as for Halo-NLS and SOX9-Halo spheroid samples in Figures 4B–4D, S4, and S5. See also Figure S3.

Prolonged culture of a stable SOX9-Halo organoid line resulted in progressive changes in organoid morphology: starting from the initially typical budding morphology (until ∼10 weeks after line establishment [LE]), organoids got rounder with smaller buds (∼15–25 weeks after LE) and finally completely spherical (∼30 weeks after LE) (Figures 3A and S3A, left). In contrast, non-transduced WT organoids continued to grow as budding structures until our longest observed culture time of ∼22 weeks (Figure 3A). This morphological change in 3D SOX9-Halo organoids was also reflected in 2D EMCs derived from them, which were characterized by smoother edges of the monolayer in comparison to WT EMCs (Figure 3A) and grew to complete confluency (Figure S3A, right), suggesting a loss in cell contact inhibition. While SOX9 expression was no longer restricted to proliferative centers (Figure 3B, left) (Mori-Akiyama et al., 2007), as expected for ubiquitous SOX9-Halo transgene expression, the average expression level of total (endogenous plus transgene) SOX9 protein in spheroids was only ∼2.3× higher than the endogenous SOX9 level in proliferative centers of WT organoids (Figure 3C, left), while the average SOX9-Halo transgene expression level was similar to that in budding SOX9-Halo organoids (Figure 3C, right). Notably, SOX9 spheroids were characterized by on average larger nuclei (Figure 3D, left) and a larger nearest-nuclei distance (Figure 3D, right) than WT organoids, indicative of a more differentiated cell population (Walther et al., 2024) (Figures 2C and S2C). Despite such morphology, spheroids contained more (76.4%) proliferative cells than WT organoids (51.1%) as determined by immunofluorescence (IF) for the proliferation marker KI67 (Miller et al., 2018) (Figures 3B, right, and 3E), consistent with the described role for SOX9 in maintaining proliferative progenitor cells (Blache et al., 2004). This increased proliferative capacity was accompanied by several aberrant cell division phenotypes, e.g., fusion of two or more nuclei, micronuclei, and fragmented nuclei (Figure S3B).

To reconcile the seemingly opposing effects of long-term SOX9-Halo overexpression, we performed bulk RNA sequencing (RNA-seq) of SOX9-Halo organoids in comparison to CTRL organoids stably expressing Halo-NLS, revealing thousands of differentially expressed genes (DEGs) (Figure 3F). Gene Ontology (GO) analysis of the 2,783 genes downregulated in spheroids revealed the top 20 biological processes to be metabolic and metabolite transport processes (Figure 3G, left), pointing toward a loss of the intestinal epithelial function in nutrient absorption and metabolism. Conversely, GO analysis of the 2,527 upregulated genes revealed pronounced association with epithelial morphogenesis and the development and differentiation of various non-intestinal tissues and organs (Figure 3G, right), indicating a loss in intestinal identity and the activation of a gene expression program resembling embryonic-like development. Wound healing, cell adhesion, actin filament organization, and cilium assembly were among other biological processes associated with genes upregulated in spheroids (Figure 3G, right), some of which we phenotypically confirmed by IF, including the formation of actin stress fibers and membrane spikes (Figure S3C). These results altogether suggest a large functional and epithelial rearrangement underlying SOX9-Halo spheroid formation.

### SOX9 overexpression results in a transient upregulation of stem cell markers followed by YAP activation and a regenerative fetal-like gene expression program

**Figure 4:**
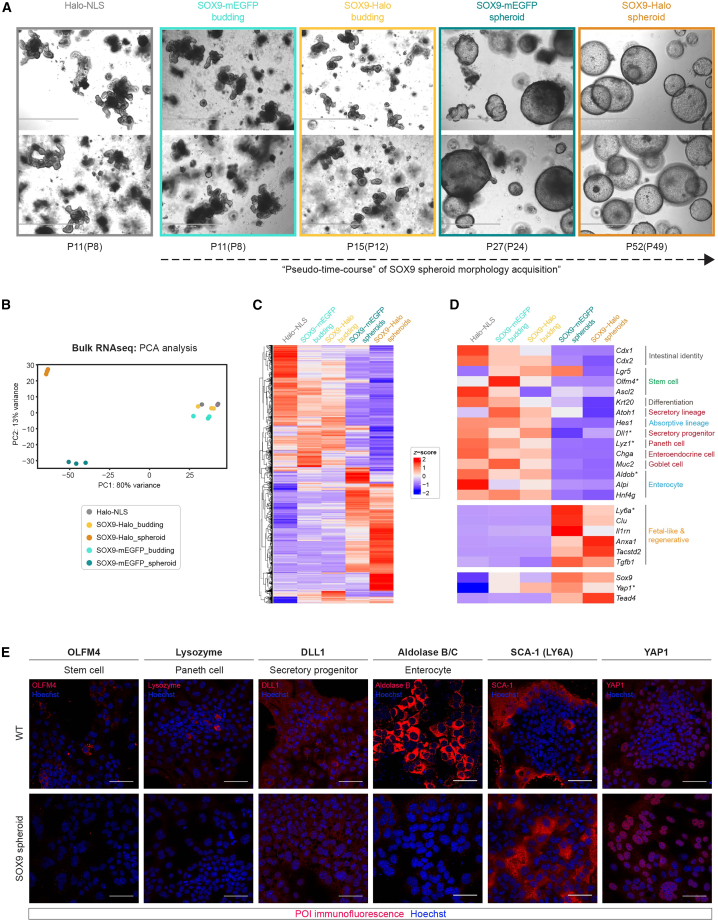
A “pseudo-time-course” of spheroid morphology acquisition upon SOX9 overexpression reveals a transient increase in stem cell markers followed by a reduction in intestinal epithelial signatures toward a fetal-like reversion (A) Representative images of the stable organoid lines Halo-NLS (gray), SOX9-mEGFP_budding (light turquoise), SOX9-Halo_budding (yellow), SOX9-mEGFP_spheroid (dark turquoise), and SOX9-Halo_spheroid (orange) at the indicated passage numbers P(P) after crypt isolation (after lentiviral transduction to make stable lines) 5 days post-seeding, chronologically ordered. Scale bars: 1 mm. (B) Principal-component analysis (PCA) of bulk RNA-seq experiments of the samples (biological triplicates) in (A). (C and D) *Z* score heatmaps of all DEGs (C) or selected genes (D) determined by bulk RNA-seq (adjusted *p* value ≤ 0.01, fold change ≥2 and mean counts ≥10; red/blue: up-/downregulated). (E) Confocal images of immunostained (POI, protein of interest) 2D EMCs derived from WT organoids (top) or SOX9-Halo spheroids (bottom) co-stained with Hoechst (blue) 5 days post-seeding. Scale bars: 50 μm. RNA-seq data in (B)–(D) are the same as in Figures S4 and S5. RNA-seq data from Halo-NLS and SOX9-Halo_spheroid samples are the same as in Figures 3F and 3G. See also Figures S4 and S5.

Given the extensive gene expression rearrangement in SOX9 spheroids, we questioned whether the HaloTag fused to the SOX9 transgene might have contributed to its emergence. Reassuringly, the budding-to-spheroid morphology transition was reproduced in a stable SOX9-mEGFP organoid line (Figure 4A) on a similar timescale, ruling out potential effects due to the introduced HaloTag. To further compare the two stable SOX9 organoid lines and gain insights into the gene expression changes underlying organoid morphology transition, we selected one budding and one spheroid phenotype for each line and ordered them phenotypically based on the progression of spheroid morphology acquisition, which also correlated with their time in culture (Figure 4A). The addition of three intermediate phenotypes (SOX9-mEGFP_budding, SOX9-Halo_budding, and SOX9-mEGFP_spheroid) (Figure 4A) to our previously analyzed SOX9-Halo spheroid and Halo-NLS organoid samples (Figures 3F and 3G) allowed us to perform a morphology “pseudo-time-course analysis” (Figure 4A). Both spheroid samples were very different from the two budding and the Halo-NLS CTRL samples (Figure S4A), which clustered together upon principal-component analysis (PCA) (Figure 4B). Nevertheless, comparing SOX9-mEGFP versus SOX9-Halo budding and spheroid samples, about a hundred (Figure S4B) or one thousand DEGs (Figure S4C) were detected, respectively, confirming that gene expression changes underlie the slightly different morphological phenotypes (Figure 4C) and ultimately lead to SOX9 spheroid formation (Figures 3F and 4A).

Focusing on selected markers of intestinal epithelial lineages, we confirmed our initial GO analysis results (Figure 3G) with a loss of expression in intestinal identity genes, stem cell markers, and markers of absorptive and secretory lineages (Beumer and Clevers, 2021) (Figures 4D and S5A), some of which we validated at the protein level by IF (Figure 4E). Inspecting more carefully the three samples Halo-NLS, SOX9-Halo_budding, and SOX9-Halo_spheroid revealed an initial mild gene expression deregulation (Figure S4D) followed by a second wave of major entity (Figure S4F). While both of these transitions were characterized by an overall downregulation of metabolic and metabolite transport processes (Figures S4E and S4G, left), including digestion for the second transition (Figure S4G left), genes upregulated in the first transition were associated with the Wnt signaling pathway and its regulation (Figure S4E, right), followed by upregulation of genes involved in development and differentiation of various non-intestinal tissues and organs in the second transition (Figure S4G, right). This transient upregulation of genes associated with Wnt signaling was in agreement with a transient increase in canonical stem cell markers including *Olfm4* (van der Flier et al., 2009) and the Wnt target gene *Lgr5* (Barker et al., 2007) in budding SOX9 organoids (Figures 4D and S5A). Instead of intestinal epithelial genes, spheroids were characterized by the expression of several genes previously associated with fetal-like and regenerative signatures (e.g., *Ly6a*, *Clu*, and *Anxa1*) (Figures 4D, S5B, and 4E), typically arising upon tissue regeneration after exposure of the intestinal epithelium to various sources of damage (Viragova et al., 2024).

The effector of the Hippo signaling pathway, Yes-associated protein (YAP1), has been associated with intestinal regeneration (Gregorieff et al., 2015; Namoto et al., 2024; Serra et al., 2019). While mRNA levels of *Yap1* were not significantly altered in the course of SOX9 spheroid phenotype acquisition (Figure S5C), 2D EMCs derived from these spheroids were characterized by an overall nuclear localization of YAP1 (Figure 4E), indicative of YAP1 activation (Yu et al., 2015). Furthermore, inspection of our bulk RNA-seq data revealed an increased expression of *Tead4* (Figures 4D and S5C), encoding a TEA domain (TEAD) TF interacting with nuclear YAP1 and thereby activating downstream genes (Zhao et al., 2008).

Taken together, our “pseudo-time-course” of spheroid morphology acquisition upon SOX9 overexpression revealed that a loss of adult homeostatic intestinal identity signatures was counteracted by a gain in fetal-like regenerative signatures. Hereby, a transient activation of Wnt signaling seems to be accompanied by a persistent activation of YAP1 signaling, resulting in a regenerative fetal-like reprogrammed phenotype.

### Increased DNA binding of SOX9-Halo in SOX9 spheroid-derived EMCs in an expression-level-dependent manner

**Figure 5:**
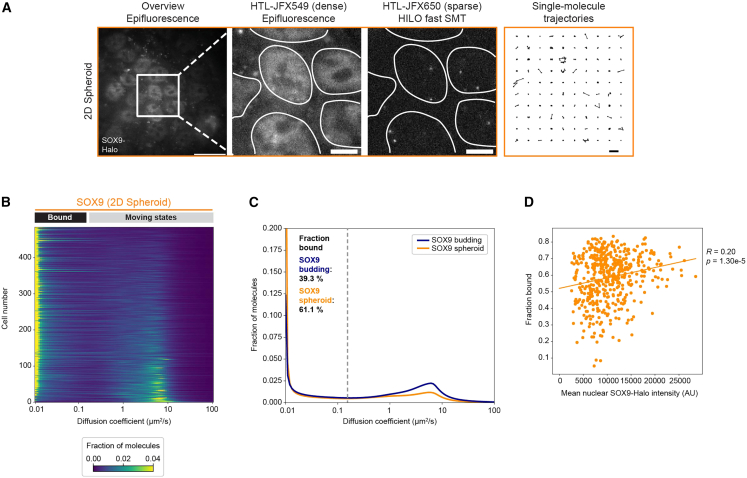
Automated fast SMT in SOX9 spheroid-derived EMCs reveals an expression-level-dependent increase in the fraction of immobile SOX9-Halo molecules (A) Double labeling of SOX9-Halo (gray) with two different HTLs allows bulk labeling (images 1, 2 from left) for nuclear segmentation (images 2, 3; white masks) and sparse labeling for fast SMT (image 3; one representative frame of an SMT movie) resulting in single-molecule trajectories (image 4; 100 randomly selected single-molecule trajectories). Scale bars: 20 μm (overview), 5 μm (zoom-in), 1 μm (trajectories). (B) Single-cell diffusion heatmap for 10 combined automated SMT experiments for SOX9-Halo in spheroid-derived 2D EMCs (*n* = 485 cells with 77, 73, 13, 24, 30, 58, 81, 71, 26, and 32 cells per experiment). Cells are ordered by decreasing fraction bound (top to bottom). Representative SMT movie in Video S6. (C) Mean diffusion spectra for SOX9-Halo in 2D EMCs derived from budding organoids (LGR5::DTR-GFP background; dark blue) or spheroids (WT background; orange). Bootstrap analysis of combined experiments with *n* = 10, 44, 42, and 56 cells for SOX9-Halo_budding and *n* = 77, 73, 13, 24, 30, 58, 81, 71, 26, and 32 cells for SOX9-Halo_spheroid determined a mean fraction bound of 39.3% (95% CI: 32.9%–46.7%) and 61.1% (95% CI: 55.4%–66.1%), respectively. Comparison of the fraction bound distributions between SOX9_budding and SOX9_spheroid yielded a *p* value of 3.39e−28. (D) Fractions bound for each spheroid-derived cell plotted against the mean nuclear SOX9-Halo intensity. The correlation (fitted line) was computed; Pearson correlation coefficient (*R*) and *p* value are indicated. The SMT data for SOX9_budding are the same as in Figures 1E–1I, 2B–2D, S1E, S1G, S1I, and S2A–S2G. See also Figure S6.

To investigate whether the diffusive behavior of SOX9-Halo changed during fetal-like reversion, we performed automated fast SMT in 2D EMCs derived from SOX9-Halo spheroids (Figure 5A). Here, the diffusive behavior of SOX9-Halo was more uniform with a dominating immobile diffusion peak for most of the about 500 cells measured (Figures 5B and S6A). Indeed, SOX9-Halo in 2D EMCs derived from spheroids was characterized by a higher fraction bound of 61.1% (95% CI: 55.4%–66.1%) in comparison to 39.3% in those derived from budding organoids (Figures 5C, S6A, and S6B; *p* value of fraction bound distribution comparison between SOX9_budding and SOX9_spheroid: 3.39e−28), raising the possibility that more SOX9 molecules bound to DNA served as a molecular driver of the observed proliferative cell state transition underlying spheroid formation. This increase in fraction bound was also apparent upon hierarchical clustering of the fast SMT data (Figures S6C–S6F). Notably, unlike the budding state, in the spheroid state, we measured a modest positive correlation between SOX9-Halo expression levels and fraction of immobile molecules (Figure 5D), indicating that, despite the presence of more SOX9-Halo molecules (Figure 3C), a larger fraction of them was immobile in the cell nucleus and thus presumably DNA-bound.

### PAPA-SMT reveals a chromatin-bound pool of self-associated SOX9 in live 2D EMCs

**Figure 6:**
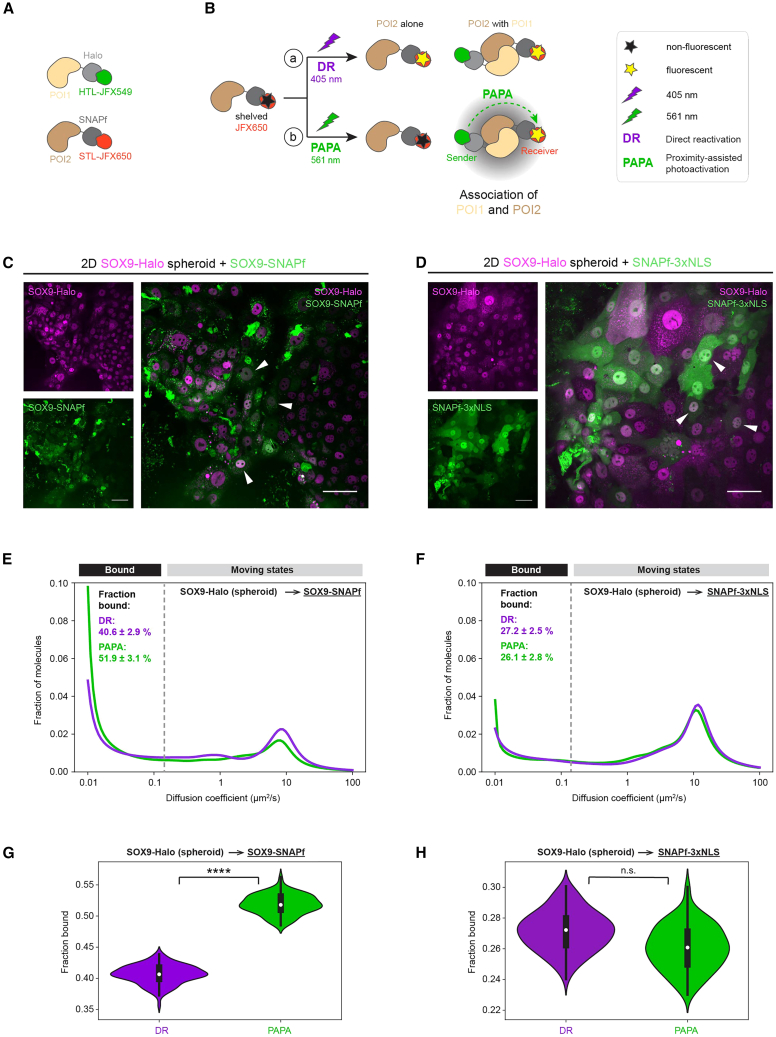
Proximity-assisted photoactivation-SMT reveals a chromatin-bound pool of self-associated SOX9 in spheroid-derived live 2D EMCs (A) Differential labeling of POI1-Halo (beige-light gray) and POI2-SNAPf (brown-dark gray) with different HTL/SNAPfTag ligand (STL)-coupled fluorophores (JFX549-sender, green; JFX650-receiver, red) for PAPA-SMT. (B) Principle underlying PAPA-SMT: through excitation using 639 nm light, shelved JFX650-labeled receiver molecules (red; black star) can either undergo (a) direct reactivation (DR; purple) upon illumination with a violet 405 nm light pulse (purple lightening arrow) making them detectable independent of any potential oligomerization states as in standard SMT, or (b) PAPA (green) upon illumination with a green 561 nm light pulse (green lightening arrow) making them only detectable if in proximity (gray cloud) to a JFX549-labeled sender molecule (green). (C and D) Confocal images of SOX9-Halo spheroid-derived 2D EMCs transduced with crude recombinant adeno-associated viral (rAAV) vectors for transient expression of (C) SOX9-SNAPf or (D) SNAPf-3xNLS (CTRL) for PAPA experiments (sender: SOX9-Halo [magenta]; receiver: SNAPf-tagged component [green]). Scale bars: 50 μm. (E and F) Mean PAPA (green) versus DR (purple) diffusion spectra for PAPA-SMT of SOX9-Halo_spheroid→SOX9-SNAPf (E) or SOX9-Halo_spheroid→SNAPf-3xNLS (F) in 2D EMCs. (G and H) Violin plots for fractions bound (white point: median; whiskers: first/third quartile) of SOX9→SOX9 (G) and SOX9→NLS (H) determined from DR/PAPA (purple/green) trajectories. Data in (E) and (G) are from 4 combined experiments with *n* = 129 cells (*n* = 35, 30, 28, and 36 cells; 14,156 DR and 9,255 PAPA trajectories; bootstrapped fractions bound: 40.6% ± 2.9% [DR], 51.9% ± 3.1% [PAPA]). Data in (F) and (H) are from 3 combined experiments with *n* = 70 cells (*n* = 21, 30, and 19 cells; 7,781 DR and 4,117 PAPA trajectories; bootstrapped fractions bound: 27.2% ± 2.5% [DR], 26.1% ± 2.8% [PAPA]). (n.s.) *p* > 0.05, ^∗∗∗∗^*p* ≤ 0.0001. For statistical details, see methods. See also Figure S7.

The increased fraction of SOX9 molecules bound to DNA in spheroids raised the question of whether SOX9 molecules might bind to DNA in an oligomerized state, as both SOX9 monomers and dimers have been reported as transcriptionally active units depending on the differentiation context (Bernard et al., 2003; Coustry et al., 2010). To address this, we employed PAPA-SMT, a recently developed single-molecule method allowing to detect whether differentially labeled sender and receiver molecules (Figure 6A) are associated or in vicinity to each other by using a PAPA illumination scheme (PAPA) distinct from that used in standard SMT (direct reactivation [DR]) (Figure 6B). To this end, we transiently expressed SOX9-SNAPf (Figure 6C) or a SNAPf-3xNLS CTRL (Figure 6D) via rAAV-based delivery (Benyamini et al., 2023) in SOX9-Halo spheroid-derived EMCs. PAPA-SMT using SOX9-Halo as a sender and SOX9-SNAPf as a receiver (Figure 6C) revealed both freely diffusing and immobile oligomerized states (Figure 6E) with a ∼11% higher bound fraction for PAPA versus DR trajectories (Figures 6E and 6G), indicating a chromatin-bound pool of oligomerized SOX9. In contrast, no difference between the bound fractions of PAPA versus DR trajectories was detected for CTRL experiments with SNAPf-3xNLS as the receiver, measuring non-specific background PAPA (Graham et al., 2025) (Figures 6F and 6H). Notably, self-associated SOX9 was also found to be partially chromatin bound in both undifferentiated and differentiated cells of 2D EMCs derived from budding SOX9-Halo organoids (Figure S7), arguing against DNA binding of an oligomerized form of SOX9 as a feature exclusive to spheroids. We nevertheless cannot exclude the existence of differences in the fraction of SOX9 molecules present in an oligomerized state or the number of SOX9 units that DNA-bound SOX9 oligomers consist of, as this PAPA-SMT assay does not provide information on whether more or larger SOX9-Halo oligomers might be immobile in the spheroid compared to the budding state.

### Fast SMT in 3D SOX9 spheroids confirms increased SOX9 binding upon fetal-like reversion

**Figure 7:**
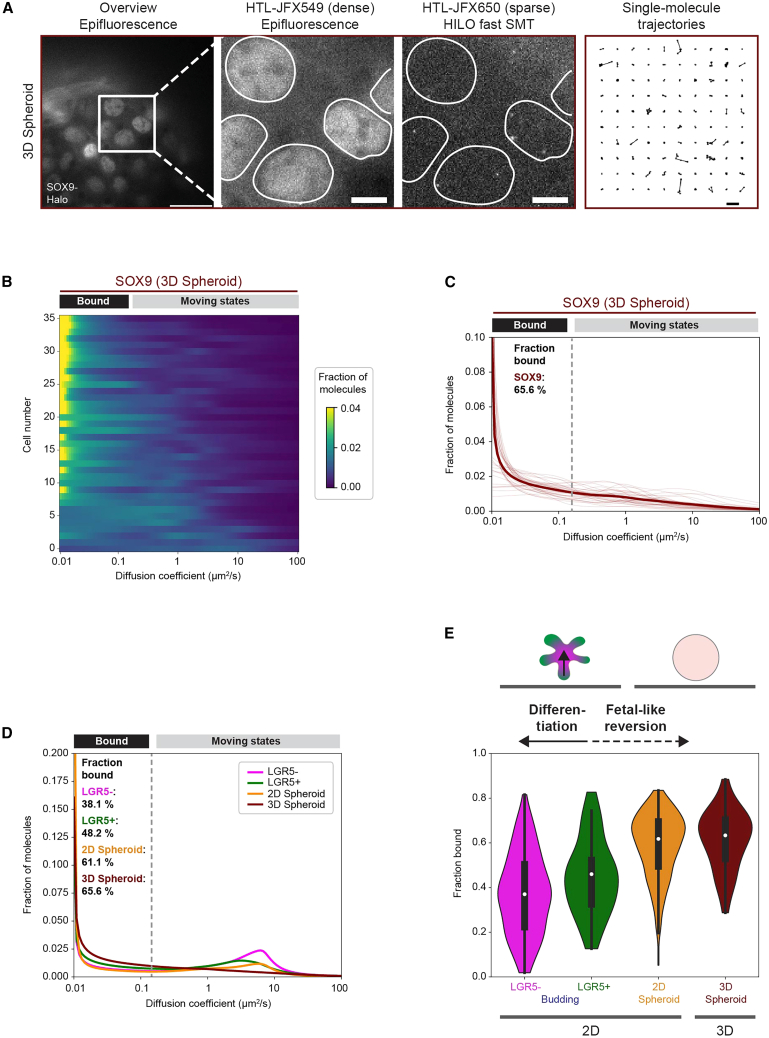
SMT in 3D spheroids confirms an increased fraction of DNA-bound SOX9-Halo molecules upon fetal-like reversion (A) Manual SMT pipeline for SOX9-Halo in 3D spheroids. Double labeling of SOX9-Halo (gray) with two different HTLs allows bulk labeling (images 1, 2) for nuclear segmentation (images 2, 3; white masks) and sparse labeling for HILO-based fast SMT (image 3; one representative frame of an SMT movie) resulting in single-molecule trajectories (image 4; 100 randomly selected single-molecule trajectories). Scale bars: 20 μm (overview), 5 μm (zoom-ins), 1 μm (trajectories). (B) Single-cell diffusion heatmap for 2 combined manual SMT experiments for SOX9-Halo in 3D spheroids (combined *n* = 36 cells with *n* = 26 and 10 cells per experiment). Cells are ordered by decreasing fraction bound from top to bottom. Representative SMT movie in Video S7. (C) Single-cell diffusion spectra for data in (B). Bootstrap analysis of combined experiments with *n* = 26 and 10 cells determined a mean fraction bound of 65.6% (95% CI: 62.5%–68.8%). (D and E) Summary of the diffusive behavior of SOX9-Halo determined by fast SMT under homeostatic differentiation conditions (budding) and upon SOX9 overexpression-induced fetal-like reversion (spheroid). (D) Mean diffusion spectra for the SOX9-Halo_budding LGR5^+/−^ (green/magenta) subpopulations and for SOX9-Halo_spheroid determined in 3D spheroids (brown) or 2D EMCs (orange) with fractions bound indicated. (E) Violin plots for fractions bound (white point: median; whiskers: first/third quartile) of samples in (D).

Finally, we sought to confirm the SOX9-Halo diffusive behavior observed in spheroid-derived 2D EMCs directly in 3D spheroids. As our HILO-based SMT approach is limited to the 10–20 μm of the sample directly above the culture glass surface, we optimized our organoid seeding procedure so that growing round spheroids could reach the glass surface, making them amenable to SMT. Using an experimental approach analogous to 2D EMCs (Figure 7A), SMT in spheroids revealed a rather homogenous diffusive behavior of SOX9-Halo across the 35 manually imaged cells (Figures 7B and 7C) with most of them being characterized by an immobile diffusion peak (Figure 7B). With an average fraction bound of 65.6% (95% CI: 62.5%–68.8%) (Figure 7C), the diffusive behavior of SOX9-Halo in 3D spheroids is thus in agreement with that measured in spheroid-derived 2D EMCs (Figure 5C; *p* value of fraction bound distribution comparison between 2D and 3D SOX9_spheroid: 0.435).

## Discussion

In this study, we combined *in vitro* models of the rapidly renewing small intestinal epithelium (Figure 1A) with automated live-cell SMT (Walther et al., 2024) to investigate how the molecular dynamics and abundance of a cell fate-conferring TF change during differentiation. We further interrogated the robustness of cell fate determination to aberrant TF expression and how TF excess might alter TF molecular dynamics. Focusing on SOX9, our results show that different cellular states in adult intestinal organoid models are characterized by distinct SOX9-Halo diffusion dynamics (Figure 7D and 7E). Under homeostatic conditions, the fraction of DNA-bound SOX9-Halo molecules decreases during differentiation from ∼48% in LGR5^+^ stem/early progenitor cells to ∼38% in LGR5^−^ late progenitor/differentiated cells (Figures 2B, 7D, and 7E), consistent with SOX9 functioning in ISC-containing crypts (Blache et al., 2004; Mori-Akiyama et al., 2007). In contrast, long-term SOX9 overexpression eventually results in an increased fraction bound of SOX9-Halo molecules (>60%) (Figures 5C and 7C–7E), underlying a proliferative cell state transition (Figures 3B and 3E) and a morphological transition from budding to spherical organoids (Figures 3A and 4A). Spheroids lack canonical epithelial cell type markers across lineages (Figures 4D, 4E, and S5A) and acquire a gene expression program resembling fetal-like reversion (Figures 4D, 4E, and S5B).

The extension of our previously developed automated SMT pipeline (Walther et al., 2024) to the recording of fluorescent cell type markers (LGR5) allowed us to measure TF diffusion dynamics in a cell-type-resolved manner (Figure 2A), validating our previously established morphology-based broad distinction between ISCs/early progenitors and late progenitors/differentiated cells (Walther et al., 2024) (Figures 1H and 1I). Parsing LGR5^+/−^ cells revealed differences in the diffusive behavior of SOX9 that correlated with differentiation (Figures 2 and S2A–S2D), while a subpopulation of small LGR5^−^ cells displaying SOX9 diffusion characteristics of LGR5^+^ cells (Figure 2C) potentially constitutes differentiated Paneth cells residing in proliferative centers, testable with additional cell type markers. Similar to our previous study on HES1 (Walther et al., 2024) and work in other differentiation contexts (Esbin et al., 2024; Kuchler et al., 2022), SOX9 is characterized by a larger fraction of immobile and hence DNA-bound molecules in the LGR5^+^ stem/early progenitor cell subpopulation (Figures 2B, 2D, and S2A–S2D) in which it has been described to function (Blache et al., 2004; Mori-Akiyama et al., 2007). Consistent with HES1 (Walther et al., 2024), also functioning during early differentiation, the diffusive behavior of SOX9 is characterized by a rather small average fraction bound of ∼39%, which could be explained by ISCs/early progenitor cells being broadly permissive epigenetic cell states that enable fast transitions to differentiated states (Jadhav et al., 2017; Kim et al., 2014). In addition, for both HES1 (Walther et al., 2024) and SOX9, the overall cell population (Figures 1E and S1E) and the proliferative/LGR5^+^ population (Figures 2C, S2A, and S2C) show a highly heterogenous TF diffusive behavior, suggesting the existence of multiple cell states, consistent with single-cell RNA-seq data (Zwick et al., 2024), and possibly distinct differentiation trajectories for each LGR5^+^ cell. Reasons for the latter could include differences in the cellular microenvironment (neighboring cell types), the progression along the same differentiation trajectory (distance from the proliferative center), or different lineages a progenitor cell has committed to. In future work, these could be probed using additional live-cell-compatible fluorescent cell type markers in combination with correlative IF for determining endpoint cell types and by tracking the same cell during differentiation with time-lapse SMT (Walther et al., 2024).

While the differences in SOX9 diffusive behavior were concentration-independent within the probed range of SOX9-Halo transgene expression levels (Figures S1G and 2D), long-term overexpression of SOX9 in mSIOs resulted in increased chromatin binding accompanied by a change in organoid morphology toward spherical (Figures 3A and 4A). In line with a recent report on SOX9 dosage robustness in the craniofacial context (Naqvi et al., 2023), these observations suggest that the intestinal epithelium can buffer modestly increased SOX9 levels (Figure 3C) for a limited time to maintain homeostasis (Figures 4A, S4D, and S4E). However, long-term SOX9 overexpression appears to break such robustness (Figures 3A, 3F, 3G, and 4). While we cannot completely rule out contributions to spheroid formation due to long-term organoid culture, our WT CTRL retaining budding morphology suggests that SOX9 overexpression at least accelerated spheroid formation if it was not the sole cause.

In addition to SOX9 dosage, future research will be required to unravel how other layers of control, such as posttranslational modifications, the availability of tissue-specific co-factors, and DNA methylation, and thus chromatin accessibility to gene-regulatory DNA motifs, come into play for SOX9 to achieve its various tissue- and context-dependent functions (Ming et al., 2022). In another differentiation system, SOX9 has been shown to act both via its capability to access closed chromatin and by competing for epigenetic factors to switch stem cell fates (Yang et al., 2023). Related to the SOX9 overexpression-induced spheroids described here, both in the fetal epithelium and upon fetal-like reversion in the adult intestinal epithelium, an increased accessibility was reported for genomic regions enriched for TF-binding sites of the SOX and TEAD families (Chen et al., 2023; Pikkupeura et al., 2023), both shown to form complexes with YAP/TAZ (Zanconato et al., 2015). In the future, it will be interesting to employ PAPA-SMT (Graham et al., 2022; 2025) (Figures 6A and 6B) to detect the interactions and study the molecular dynamics of such complexes in differentiation and reprogramming contexts. While our PAPA experiments suggest that SOX9 binds to DNA in an oligomerized form (Figures 6E, 6G, and S7A), possibly dimers as reported in other contexts (Bernard et al., 2003; Coustry et al., 2010), this assay cannot determine the degree of SOX9 oligomerization, including putative nuances in SOX9 oligomerization between undifferentiated and differentiated cells (Figures S7E and S7G), and whether such immobile self-associated SOX9 moieties are all bound at *cis*-regulatory elements of SOX9 target genes. Thus, another possible yet purely speculative scenario could be the formation of gene regulatory SOX9 hubs via weak multivalent interactions mediated by intrinsically disordered regions (IDRs) (Chong and Mir, 2021), testable with IDR mutants of SOX9 in future work. Beyond a potential link between the SOX9 expression level and its oligomerization, it remains to be tested whether an increased SOX9 dosage resulting in more immobile SOX9 molecules additionally extends the canonical DNA sites occupied by SOX9 in a “spill-over” effect, promoting fetal-like reprogramming.

Constitutive SOX9 expression is a common pathway to cancers through the activation of oncogenic transcription regulators (Grimm et al., 2020; Kumar and Mistri, 2020), consistent with the gain in proliferative capacity in the SOX9 overexpression-induced spheroids (Figures 3B and 3E). However, these spheroids were characterized by a loss in epithelial identity and function toward the activation of a gene expression program resembling that of regenerative fetal-like reversion upon tissue damage (Viragova et al., 2024) (Figures 3G, 4C–4E, and S5), consistent with SOX9’s essential role in several developmental pathways (Ming et al., 2022). A similar gene expression signature is characteristic of fetal intestinal epithelial cultures in comparison to enteroids generated from adult intestinal cells (Fordham et al., 2013; Mustata et al., 2013). Hyperproliferative cells and fetal-like gene expression signatures were further found in crypts associated with granulomas resulting from helminth infection (Nusse et al., 2018) and enriched in the colonic epithelium upon damage induced by dextran sodium sulfate (Yui et al., 2018). Similar fetal-like transcriptional programs have been reported upon other injuries to ISCs or the intestinal crypt, such as diphtheria toxin-mediated ISC ablation, ionizing radiation, and chemotherapy (Ayyaz et al., 2019; Iqbal et al., 2025; Malagola et al., 2024; Mustata et al., 2013; Singh et al., 2022). Consistent with the observation of YAP1 activation in SOX9 spheroids (Figure 4E), such a regenerative response of fetal-like reversion is typically mediated by YAP1, which has a well-established role in driving pattern formation during intestinal regeneration (Gregorieff et al., 2015; Serra et al., 2019). While during an intestinal tissue damage response this transient fetal-like state is exited and intestinal homeostasis is reestablished, it is unclear whether this can be achieved in the SOX9 overexpression-induced spheroid context. To this end, a downregulation of YAP activity together with an upregulation of Wnt and Notch signals to sustain homeostatic ISCs might be required (Tian et al., 2015), while retinoid X receptor signaling could also be involved (Lukonin et al., 2020).

The activation of a YAP1-SOX9 circuit is both necessary and sufficient to induce a regenerative fetal-like reversion (Viragova et al., 2024). However, it has not been reported that SOX9 overexpression alone, whether directly or through intermediate steps, can act as a driver of this circuit. Nevertheless, a recent study suggests that SOX9 can promote the nuclear translocation and hence activation of YAP through direct interaction (Qian et al., 2024). Further studies will be required to elucidate whether SOX9 overexpression in the intestinal epithelial context directly results in YAP activation and fetal-like reprogramming or whether this involves a more complex cascade of molecular events, including triggering of YAP activation through changes in cell-cell contacts or physical properties of the extracellular matrix (Aragona et al., 2013; Dupont et al., 2011). In this respect, the long timescale of several months required for the acquisition of the described fetal-like reprogrammed state will be advantageous for resolving intermediate steps during this organoid morphology transition in multi-pronged, scale-bridging time-course experiments. In addition, it will be interesting to investigate how the epithelial response across scales depends on varying initial SOX9 levels. Furthermore, a reduction in SOX9 expression at various time points during spheroid morphology acquisition will address a potential reversibility of this process. Altogether, while keeping the need of strict control mechanisms to avoid tumor initiation upon constitutive SOX9 expression in mind (Grimm et al., 2020; Kumar and Mistri, 2020), such studies will inform about the potential of the described SOX9 overexpression-induced fetal-like reversion to be exploited for applications in regenerative medicine.

### Limitations of the study

Here, we used SOX9-Halo fusions (Figure 1B), which were key for live imaging experiments including SMT (Figures 1C and 1D). By determining a decreased fraction bound for a SOX9 mutant lacking its DNA-binding domain (Figures 1E–1I), we confirmed functionality of SOX9-Halo for DNA binding. Furthermore, we could reproduce a SOX9 overexpression-induced spheroid phenotype with both Halo and mEGFP fusions (Figure 4A). Moreover, we modestly overexpressed transgene-encoded SOX9-Halo in addition to unlabeled endogenous SOX9 (Figure 3C), necessitating the consideration of the presence of a mixed SOX9 population for data interpretation, which might have resulted in underestimated fractions bound (Figures 1F, 1G, 2B, 5C, 7C–7E, S1A, S1E, S1F, S1I, S2A, S2B, S2H, S2I, and S6A) and oligomerized states (Figures 6E–6H and S7). Future studies could be improved by endogenous tagging (Koch et al., 2018) to investigate SOX9 molecular dynamics at physiological expression levels and to manipulate endogenous levels for interrogating TF dosage effects.

We found a large heterogeneity in the cellular diffusion behavior of SOX9-Halo (Figures 1E, 1G, 1H, 1I, 5D, S1E, S1G, S6A, and S6B), even within cell state-enriched subpopulations (Figures 2B–2D and 5D). Notably, we also observed some cell-to-cell heterogeneity in the bound fractions of the SOX9ΔHMG-Halo DNA-binding mutant (Figures 1E, 1H and 1I) as well as in the H2B-Halo and Halo-NLS CTRLs (Figure S8A). Potential sources for the observed cell-to-cell variability in our CTRLs are extracellular microenvironment, cell-intrinsic differences in the cell cycle or metabolic state, and cell fitness, with a few likely abnormal or dying cells not identified as such in manual QC captured across experiments (Figure S8B). By comparing LGR5^+^ vs. LGR5^−^ subpopulations (Figures S2H, S2I, and S8C) for these CTRLs, we ruled out cellular differentiation state as a source of variability in fractions bound. In contrast, the large cell-to-cell variability within LGR5^+^ (stem and early progenitor cells) and LGR5^−^ (late progenitor and differentiated cells) subpopulations for SOX9-Halo (Figures 2B–2D, S2A–S2C, and S8A) is largely of biological nature, as they contain several differentiation states. Nevertheless, despite the observed cell-to-cell heterogeneities, pairwise comparisons of the fractions bound between the various proteins measured in this study were all significant (Figure S8C).

3D mSIOs and 2D EMCs are well-characterized differentiation systems recapitulating important features of the intestinal epithelium (Figure 1A). Nevertheless, they are simplistic *in vitro* models, and therefore, further studies are required to test the validity of our results in the *in vivo* context and their clinical applicability. SMT deep in tissues remains challenging, and different microscopy techniques capable of imaging single molecules inside thicker specimens (Chen et al., 2014) are required for SMT in whole 3D mSIOs. However, our optimization of seeding and imaging conditions enabling HILO-based fast SMT in 3D spheroids (Figures 7A–7C) is an important step toward a broader use in the outer layer of organoid models for connecting multicellular phenotypes with molecular mechanisms.

## Methods

### Experimental model and subject participant details

#### Mice

C57BL/6J mice (WT) were obtained from The Jackson Laboratory (strain #000664, RRID:IMSR_JAX:000664). LGR5::DTR-EGFP mice (Tian et al., 2011) were kindly provided by Fred de Sauvage (Genentech). WT and LGR5::DTR-EGFP mice were bred with C57BL/6J mice and housed in an AAALAS-certified level 3 facility on a 14 h light cycle. Pups were weaned 21 days after birth and housed with four littermates per cage. Female offspring was used for mouse small intestinal crypt isolation and organoid generation at an age of 8–16 weeks. All procedures to maintain and use the mice were approved by the Institutional Animal Care and Use Committee of the University of California, Berkeley (IACUC protocol number AUP-2015-09-7988-2).

#### Cell lines

L-Wnt3a cells (CRL-2647, ATCC) were used to produce Wnt3a-conditioned medium. HEK293T cells (CRL-3216, ATCC) were used to generate rAAV vectors for crude rAAV vector preparations, and HEK293T Lenti-X cells (Takara, cat.# 632180) for making lentivirus. Cell lines were obtained via the UC Berkeley Cell Culture Facility. Cell lines and organoids were confirmed to be mycoplasma-free by regular PCR testing.

### Experimental procedures

Detailed methods can be found in the supplemental experimental procedures. Key methods are described in brief in the following text.

#### Culture of mouse small intestinal organoids and 2D enteroid monolayers

Intestinal organoids were derived from mouse small intestinal crypts and cultured as described before (Walther et al., 2024). 2D EMCs were derived from mSIOs and cultured as described before (Walther et al., 2024). For details, see supplemental experimental procedures.

#### Generation of stable organoid lines

Stable organoid lines were generated by lentivirus transduction and antibiotic selection as described before (Walther et al., 2024). For details, see supplemental experimental procedures.

#### Transient transduction of 2D enteroid monolayers using crude rAAV preparations

2D EMCs were transiently transduced using crude rAAV preparations, which were generated as previously described (Benyamini et al., 2023). For details, see supplemental experimental procedures.

#### Immunostaining

IF experiments on 2D EMCs were performed as described (Walther et al., 2024). For details, see supplemental experimental procedures.

#### Confocal imaging

Confocal imaging of mSIOs and 2D EMCs was performed as previously described (Walther et al., 2024). For details and modifications, see supplemental experimental procedures.

#### Single-molecule imaging and automation

Fast SMT and PAPA-SMT experiments were performed on a TIRF microscope and analyzed as described before (Walther et al., 2024), whereby most experiments were executed using the automated pipeline for SMT and analysis described before (Walther et al., 2024) with the add-on to record additional fluorescence markers. For details and modifications, see supplemental experimental procedures.

#### Bulk RNA-seq

RNA for bulk RNA-seq was extracted from intestinal organoids using TRIzol and phenol-chloroform extraction, followed by poly-A RNA-seq library preparation. RNA-seq data were analyzed using common bioinformatics tools. For details, see supplemental experimental procedures.

#### Statistical testing

Quantifications and comparisons between conditions were statistically tested as described in the supplemental experimental procedures.

## Resource availability

### Lead contact

Further information and requests for resources and reagents should be directed to and will be fulfilled by the lead contact and corresponding author, Nike Walther (nikewalther.science@gmail.com).

### Materials availability

Plasmids generated in this study are available upon request with a completed material transfer agreement and with reasonable compensation by the requestor for shipping. There are restrictions to the availability of stable organoid lines generated in this study due to the lack of an external centralized repository for their distribution and our need to maintain the stock.

### Data and code availability

• Microscopy data have been deposited to Mendeley Data and will be publicly available upon publication via the links indicated here: https://gitlab.com/nikewalther/walther_sox9organoid_2025/-/tree/main/DataDeposit.
• RNA-seq data have been deposited to the NCBI Gene Expression Omnibus (GEO) under accession number GSE287653 and will be publicly available upon publication.
• Original code has been deposited to GitLab (https://gitlab.com/nikewalther/walther_sox9organoid_2025/-/tree/main?ref_type=heads) and will be publicly available upon publication.
• Information required to reanalyze the data has been included.

## Acknowledgments

We are grateful to Robert Tjian and 10.13039/100010933Xavier Darzacq for hosting the experimental part of this study in their joint lab at the 10.13039/100006978University of California, Berkeley, and for providing funding (Robert Tjian via the 10.13039/100000011Howard Hughes Medical Institute [34430] and 10.13039/100010933Xavier Darzacq via a Dynamic Imaging Grant from the 10.13039/100014989Chan Zuckerberg Initiative [Dynamic-0000000091]). We thank current and past members of the Tjian/Darzacq lab for scientific discussions and comments on the manuscript. We are grateful to Qiulin Zhu and Brendan Wu for cloning assistance, Sanchitha Kannabran and Sophia Lim for assistance with plasmid preparations and organoid cultures, Xinyin Lu for plasmid preparations, and Shuang Zheng for assistance with mouse colony maintenance. We thank Thomas Graham for providing code for (PAPA)-SMT automation and analysis. We are thankful to Fred de Sauvage for providing LGR5::DTR-EGFP mice, Mark Kay for KP1 capsid plasmids, and Luke Lavis for JF dyes. We thank the UC Berkeley Cell Culture Facility supported by The University of California, Berkeley, for providing cell lines. We are grateful to Ophir Klein and current and past members of the Klein lab gut group for sharing protocols and providing a platform for discussion and feedback. We would like to thank Fred de Sauvage and Kim Boonekamp for critically reading and commenting on the manuscript. N.W. extends her gratitude to Olaf Stemmann and the 10.13039/100005685Department of Genetics at the 10.13039/100020618University of Bayreuth for hosting her for parts of this study. N.W. acknowledges funding from the Berkeley Stem Cell Center via a Siebel postdoctoral fellowship as well as from the 10.13039/501100001659German Research Foundation (DFG) via a Walter Benjamin postdoctoral fellowship (453309976) and a return grant of the Walter Benjamin program (552461306). S.A. was supported by a Donner 160 fellowship. A.C.M. acknowledges support via the 10.13039/100000900California Institute for Regenerative Medicine Training Program (EDUC4-12790).

## Author contributions

N.W. conceptualized, designed, and supervised the study, developed experimental strategies for rAAV-based transgene delivery into 2D EMCs as well as for SMT in 3D spheroids, devised the further development of the automated SMT imaging and analysis pipeline, manually segmented confocal images, analyzed imaging and RNA-seq data, and wrote the manuscript (original draft and review/editing). N.W. executed all experiments except for RNA extraction and library preparation for RNA-seq, which was performed by C.C., and confocal imaging of one IF condition, which was performed by S.A. S.A. further developed the automated SMT pipeline to accommodate the acquisition of additional fluorescent markers in 2D EMCs and to enable the separate analysis of manually classified SMT data with input from N.W., wrote tailored SMT and image analysis code including statistical analyses, and analyzed a subset of confocal data. C.C. processed RNA-seq data and performed initial analyses. S.A. and C.C. discussed data and their interpretation with N.W., provided a subset of raw figure panels as well as method details, and reviewed and edited the manuscript. G.M.D. designed and cloned DNA constructs. A.C.M. provided critical reagents and advice for rAAV-based transgene delivery. N.W. provided funding for this project. All authors agreed on the final version of the manuscript.

## Declaration of interests

The authors declare no competing interests.

## Supplemental information

Document S1. Figures S1–S10 and supplemental experimental procedures

Video S1. Representative SMT movie of SOX9-Halo in 2D EMCs, related to Figure 1 Nuclear segmentation masks are shown as white contour lines. Single molecules within nuclear masks that passed manual QC were classified as bound (red circle) or diffusing (yellow circle). The movie is playing in real time (min:s:ms, top left) and the frame number is indicated (top right). Scale bar: 5 μm.

Video S2. Representative SMT movie of SOX9ΔHMG-Halo in 2D EMCs, related to Figure 1 Nuclear segmentation masks are shown as white contour lines. Single molecules within nuclear masks that passed manual QC were classified as bound (red circle) or diffusing (yellow circle). The movie is playing in real time (min:s:ms, top left) and the frame number is indicated (top right). Scale bar: 5 μm.

Video S3. Representative SMT movie of SOX9-Halo in 2D EMCs, related to Figure 2 Green/magenta nuclear segmentation masks indicate LGR5^+/−^ cells. Single molecules within nuclear masks that passed manual QC were classified as bound (red circle) or diffusing (yellow circle). The movie is playing in real time (min:s:ms, top left) and the frame number is indicated (top right). Scale bar: 5 μm.

Video S4. Representative SMT movie of H2B-Halo in 2D EMCs, related to Figure S2 Green/magenta nuclear segmentation masks indicate LGR5^+/−^ cells. Single molecules within nuclear masks that passed manual QC were classified as bound (red circle) or diffusing (yellow circle). The movie is playing in real time (min:s:ms, top left) and the frame number is indicated (top right). Scale bar: 5 μm.

Video S5. Representative SMT movie of Halo-NLS in 2D EMCs, related to Figure S2 Green/magenta nuclear segmentation masks indicate LGR5^+/−^ cells. Single molecules within nuclear masks that passed manual QC were classified as bound (red circle) or diffusing (yellow circle). The movie is playing in real time (min:s:ms, top left) and the frame number is indicated (top right). Scale bar: 5 μm.

Video S6. Representative SMT movie of SOX9-Halo in spheroid-derived 2D EMCs, related to Figure 5 Nuclear segmentation masks are shown as white contour lines. Single molecules within nuclear masks that passed manual QC were classified as bound (red circle) or diffusing (yellow circle). The movie is playing in real time (min:s:ms, top left) and the frame number is indicated (top right). Scale bar: 5 μm.

Video S7. Representative SMT movie of SOX9-Halo in 3D spheroids, related to Figure 7 Nuclear segmentation masks are shown as white contour lines. Single molecules within nuclear masks that passed manual QC were classified as bound (red circle) or diffusing (yellow circle). The movie is playing in real time (min:s:ms, top left) and the frame number is indicated (top right). Scale bar: 5 μm.

Document S2. Article plus supplemental information
