# Supplementary material for: Distinct SOX9 single-molecule dynamics characterize adult differentiation and fetal-like reprogrammed states in intestinal organoids
Source: Stem Cell Reports. 2026 Jan 22;21(2):102787. doi: 10.1016/j.stemcr.2025.102787 (PMC12903095; doi:10.1016/j.stemcr.2025.102787)
Supplement: Document S1. Figures S1–S10 and supplemental experimental procedures [file mmc1.pdf]

Stem Cell Reports, Volume *21*

Supplemental Information

Distinct SOX9 single-molecule dynamics characterize adult differentiation and fetal-like reprogrammed states in intestinal organoids

Nike Walther, Sathvik Anantakrishnan, Gina M. Dailey, Anna C. Maurer, and Claudia Cattoglio

Figure S1

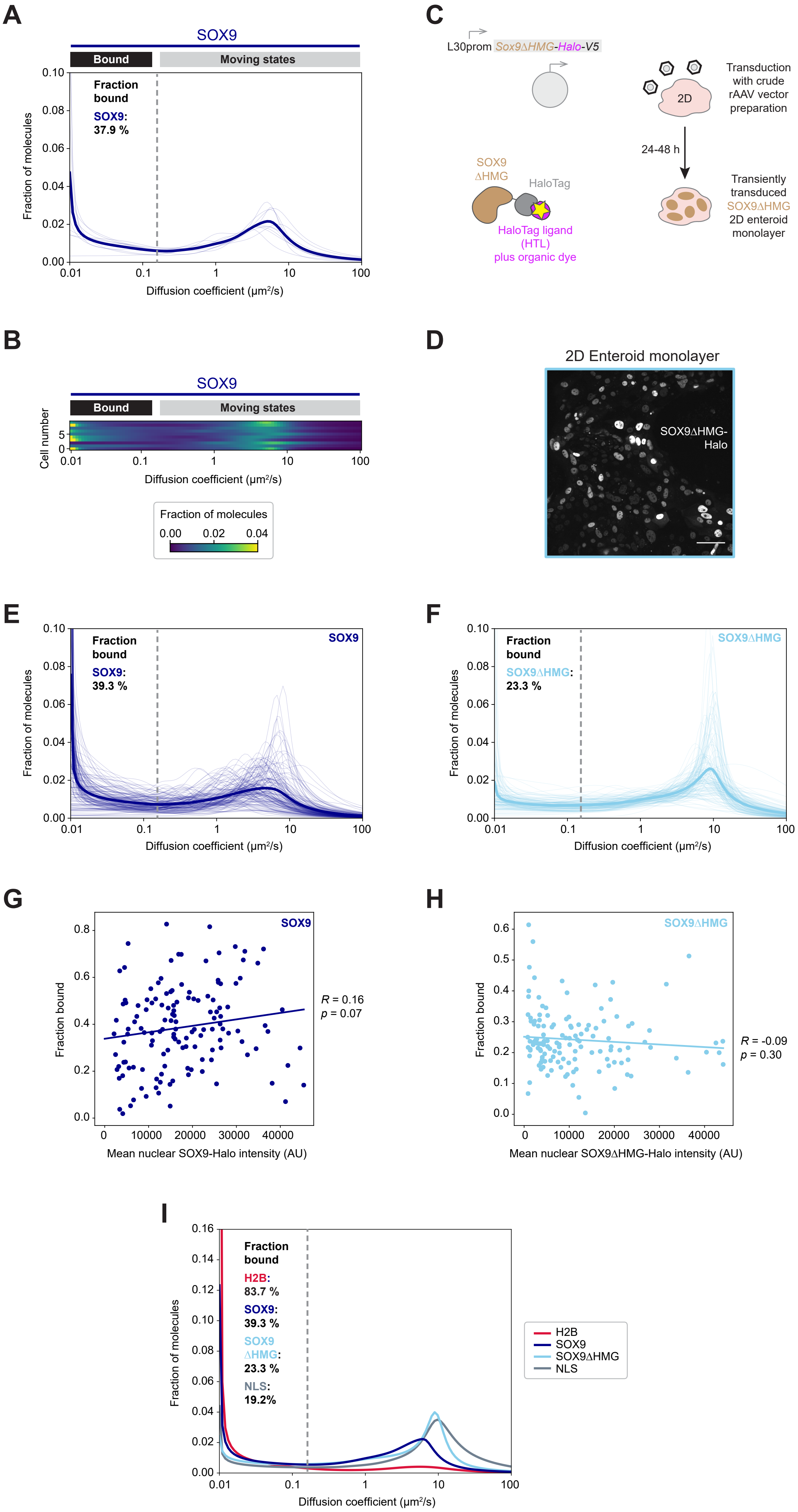
A
SOX9
Bound
Moving states
Fraction bound
SOX9:
37.9 %
Fraction of molecules
Diffusion coefficient (µm²/s)
C
L30prom Sox9ΔHMG-Halo-V5
Transduction with crude rAAV vector preparation
2D
24-48 h
SOX9 ΔHMG
HaloTag
HaloTag ligand (HTL) plus organic dye
Transiently transduced SOX9ΔHMG 2D enteroid monolayer
B
SOX9
Bound
Moving states
Cell number
Diffusion coefficient (µm²/s)
Fraction of molecules
D
2D Enteroid monolayer
SOX9ΔHMG-Halo
E
Fraction bound
SOX9:
39.3 %
SOX9
Fraction of molecules
Diffusion coefficient (µm²/s)
F
Fraction bound
SOX9ΔHMG:
23.3 %
SOX9ΔHMG
Fraction of molecules
Diffusion coefficient (µm²/s)
G
SOX9
Fraction bound
R = 0.16
p = 0.07
Mean nuclear SOX9-Halo intensity (AU)
H
SOX9ΔHMG
Fraction bound
R = -0.09
p = 0.30
Mean nuclear SOX9ΔHMG-Halo intensity (AU)
I
Fraction bound
H2B:
83.7 %
SOX9:
39.3 %
SOX9 ΔHMG:
23.3 %
NLS:
19.2%
H2B
SOX9
SOX9ΔHMG
NLS
Fraction of molecules
Diffusion coefficient (µm²/s)

<u>Figure S1:</u> The diffusive behaviors of SOX9-Halo and its DNA binding domain mutant SOX9ΔHMG-Halo are expression level-independent, related to Figure 1. **(A,B)** Single-cell diffusion spectra (A) or diffusion heatmap (B) of a manual fast SMT experiment in SOX9-Halo 2D EMCs with a mean fraction bound of 37.9% (95% CI: 35.8-39.4%). In (B) cells are ordered by decreasing fraction bound from top to bottom (*n*=10 cells). **(C)** Generation of an organoid line transiently overexpressing a SOX9ΔHMG-HaloTag-V5 transgene through transduction with a crude rAAV vector preparation allows fluorescence detection (yellow star) of SOX9ΔHMG-Halo (brown-gray) covalently labeled with a dye-coupled HTL (magenta). **(D)** Confocal live imaging of a 2D EMC 48h post-transduction with a crude rAAV preparation to transiently express SOX9ΔHMG-Halo. Scale bar: 50 µm. **(E,F)** Single-cell diffusion spectra for 4 independent automated experiments for SOX9-Halo (E) and 5 independent manual experiments for SOX9ΔHMG-Halo (F). **(G,H)** Fractions bound for each cell are plotted against the mean nuclear fluorescence intensity for SOX9-Halo (G) or SOX9ΔHMG-Halo (H). Correlations between POI intensity and fraction bound (fitted line) were computed for each POI and the Pearson correlation coefficients (*R*) and *p*-values are indicated. **(I)** Mean diffusion spectra for H2B-Halo (red), SOX9-Halo (dark blue), SOX9ΔHMG-Halo (light blue), and Halo-NLS (gray). Bootstrap analysis of combined experiments with *n*=65,30,12,50,123,81,6 cells for H2B, *n*=10,44,42,56 cells for SOX9, *n*=16,42,34 cells for SOX9ΔHMG, and *n*=12,47,41 cells for NLS determined mean fractions bound of 83.7% (95% CI: 81.4-85.9%), 39.3% (95% CI: 32.9-46.7%), 23.3% (95% CI: 19.5-28.5%), and 19.2% (95% CI: 15.0-23.2%) respectively. Cells in (E-I) correspond to the 4 experiments for SOX9 (dark blue; combined *n*=152 cells) and the 5 experiments for SOX9ΔHMG (light blue; combined *n*=135 cells) plotted in Fig. 1E-I. Cells in (I) further correspond to the 7 experiments for H2B (red; combined *n*=367 cells) and the 3 experiments for NLS (gray; combined *n*=100 cells) plotted in Fig. 1G and Fig. S2H,I.

Figure S2

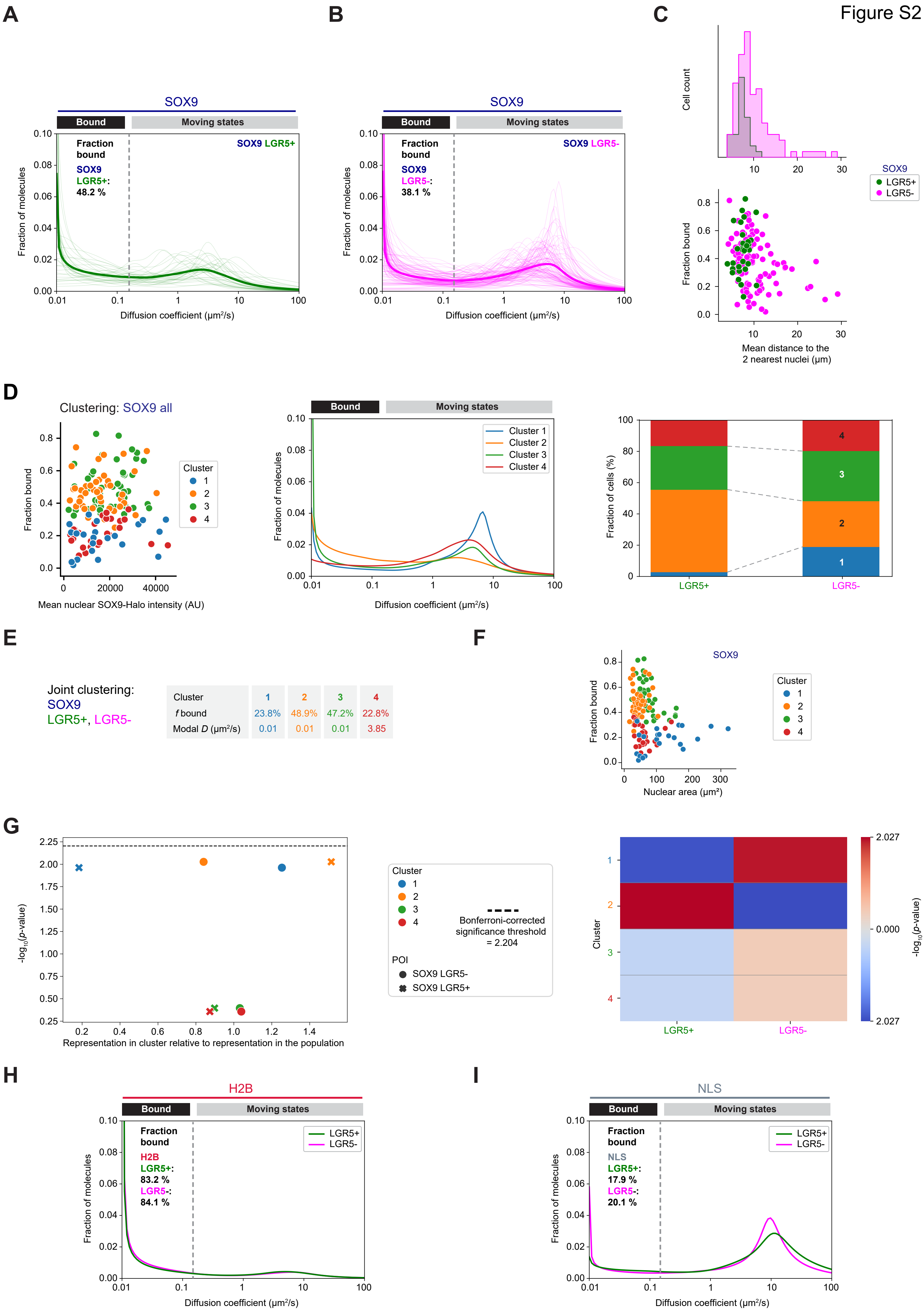
A
B
C
SOX9
Bound
Moving states
Fraction bound
SOX9 LGR5+: 48.2 %
SOX9 LGR5+
SOX9 LGR5-: 38.1 %
SOX9 LGR5-
Fraction of molecules
Diffusion coefficient (µm²/s)
Cell count
Fraction bound
Mean distance to the 2 nearest nuclei (µm)
LGR5+
LGR5-
D
Clustering: SOX9 all
Cluster
1
2
3
4
Mean nuclear SOX9-Halo intensity (AU)
Cluster 1
Cluster 2
Cluster 3
Cluster 4
Fraction of cells (%)
E
Joint clustering:
SOX9
LGR5+, LGR5-
Cluster 1 2 3 4
f bound 23.8% 48.9% 47.2% 22.8%
Modal D (µm²/s) 0.01 0.01 0.01 3.85
F
Nuclear area (µm²)
G
-log10(p-value)
Representation in cluster relative to representation in the population
Bonferroni-corrected significance threshold = 2.204
POI
SOX9 LGR5-
SOX9 LGR5+
2.027
0.000
H
H2B
H2B LGR5+: 83.2 %
LGR5-: 84.1 %
I
NLS
NLS LGR5+: 17.9 %
LGR5-: 20.1 %

Figure S2: Differences in the diffusive behavior of SOX9-Halo in stem- and differentiated cell populations, but not for Halo-tagged immobile H2B and freely diffusing NLS CTRLs, related to Figure 2. (A,B) Single-cell diffusion spectra for 4 independent automated experiments for SOX9-Halo classified into LGR5+ (green) (A) and LGR5- (magenta) (B) subpopulations with *n*=36 and *n*=116 cells, respectively. **(C)** Top: Distribution of the single-cell morphological characteristic mean distance to the two nearest nuclei for LGR5+/- (green/magenta) cells extracted from SMT data. Bottom: Single-cell correlation of SMT-derived fraction bound with mean distance to the two nearest nuclei for LGR5+/- (green/magenta) cells. **(D-G)** Hierarchical clustering of all SOX9-Halo cells based on single-cell diffusion spectra using the Jensen-Shannon distance metric. (D) Left: Fractions bound for each cell plotted against the mean nuclear SOX9-Halo intensity with color-coded diffusion clusters. Middle: Mean diffusion spectra for each cluster. Right: Distribution of cells into diffusion clusters for LGR5+/- (left/right). (E) Cluster statistics. (F) Fractions bound for each cell plotted against the nuclear area with color-coded diffusion clusters. (G) Left: *p*-values indicating the representation of LGR5+ (x mark) and LGR5- (circle) subpopulations in each diffusion cluster relative to the representation in the population; Bonferroni-corrected significance threshold (dashed line). Right: Heatmap of *p*-values indicating the representation of each subpopulation in each diffusion cluster (red: overrepresentation; blue: underrepresentation). Cluster: 1-blue, 2-orange, 3-green, 4-red. Shown are *n*=152 cells from 4 combined automated experiments (*n*=10,44,42,56 cells). **(H,I)** Mean diffusion spectra for LGR5+/- (green/magenta) subpopulations of (H) H2B-Halo (LGR5+: *n*=174 cells; LGR5-: *n*=190 cells) and (I) Halo-NLS (LGR5+: *n*=47 cells; LGR5-: *n*=53 cells). Bootstrap analysis of combined experiments with *n*=65,30,12,50,123,81,6 cells for H2B and *n*=12,47,41 cells for NLS determined mean fractions bound of 83.2% (95% CI: 81.1-85.3%) and 84.1% (95% CI: 81.7-86.4%) as well as 17.9% (95% CI: 13.7-22.5%) and 20.1% (95% CI: 15.9-23.6%) for LGR5+/- subpopulations, respectively. Comparison of the fraction bound distributions between LGR5+ and LGR5- cells yielded the following *p*-values: H2B – 0.125, NLS – 0.914. Representative SMT movies in Videos S4,5. The SOX9 data are the same as in Fig.1E-I, Fig. S1E,G,I, Fig. 2B-D, and Fig. 5C. The H2B and NLS data are the same as in Fig. 1G and Fig. S1E.

Figure S3

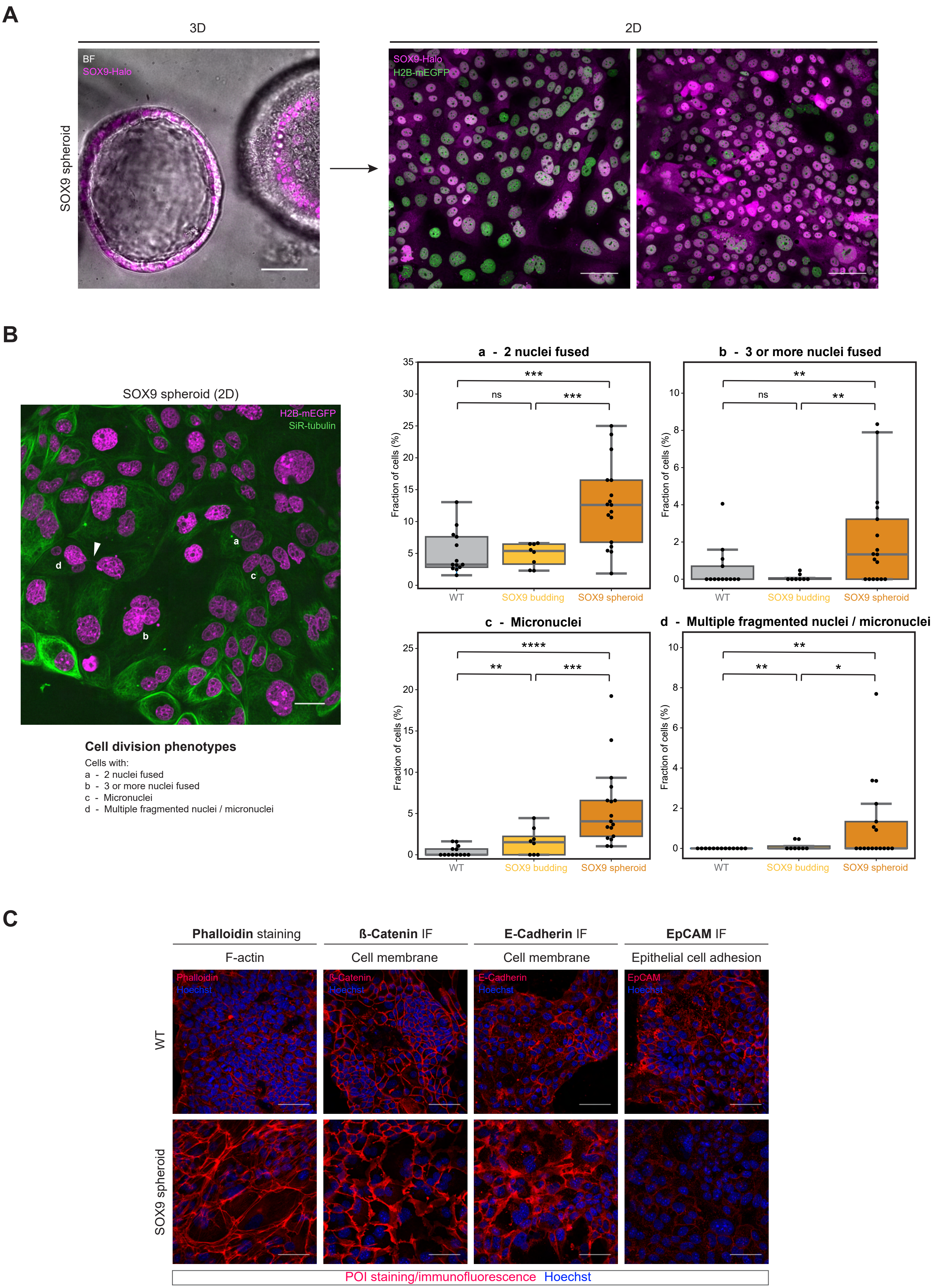
A
3D
2D
SOX9 spheroid
BF
SOX9-Halo
SOX9-Halo
H2B-mEGFP
B
SOX9 spheroid (2D)
H2B-mEGFP
SiR-tubulin
a
b
c
d
Cell division phenotypes
Cells with:
a - 2 nuclei fused
b - 3 or more nuclei fused
c - Micronuclei
d - Multiple fragmented nuclei / micronuclei
a - 2 nuclei fused
b - 3 or more nuclei fused
c - Micronuclei
d - Multiple fragmented nuclei / micronuclei
Fraction of cells (%)
ns
***
**
****
*
WT
SOX9 budding
SOX9 spheroid
C
Phalloidin staining
ß-Catenin IF
E-Cadherin IF
EpCAM IF
F-actin
Cell membrane
Cell membrane
Epithelial cell adhesion
Phalloidin
ß-Catenin
E-Cadherin
EpCAM
Hoechst
WT
SOX9 spheroid
POI staining/immunofluorescence Hoechst

Figure S3: SOX9-Halo spheroids display cell division errors, actin stress fibers, and signs of cell membrane remodeling, related to Figure 3. (A) Confocal live imaging of SOX9-Halo spheroids co-expressing H2B-mEGFP 5 d post-seeding. Left: 3D spheroid (BF: gray; SOX9-Halo: magenta). Right: 2D EMC derived from 3D spheroid (SOX9-Halo: magenta; H2B-mEGFP: green). Scale bars: 50 µm. **(B)** Cell division phenotypes in 2D EMCs derived from SOX9-Halo spheroids. Left: Representative confocal image of live SOX9 spheroid-derived 2D EMC (H2B-mEGFP: magenta; SiR-tubulin: green) with cell division phenotypes (a-d) and anaphase bridge (arrow) indicated. Scale bar: 20 µm. Right: Quantifications of cell division phenotypes (a: two nuclei fused; b: three or more nuclei fused; c: micronuclei; d: multiple fragmented nuclei or micronuclei) in 2D EMCs derived from WT (gray), SOX9-Halo_budding (yellow), or SOX9-Halo_spheroid (orange) organoids. Quantifications were based on 13 images with n=1821 cells for WT, 8 images with n=1626 cells for SOX9_budding, and 17 images with n=1756 cells for SOX9_spheroid. Each point represents one FOV; median: gray line, first/third quartile: whiskers; statistical testing based on Mann-Whitney U tests (see experimental procedures for details); (ns) non-significant, $p>0.5$; (*) $p \leq 0.5$; (**) $p \leq 0.01$; (***) $p \leq 0.001$; (****) $p \leq 0.0001$. **(C)** Confocal images of immunostained (POI: red) 2D EMCs derived from WT organoids (top) or SOX9-Halo spheroids (bottom) co-stained with Hoechst (blue). Scale bars: 50 µm.

Figure S4

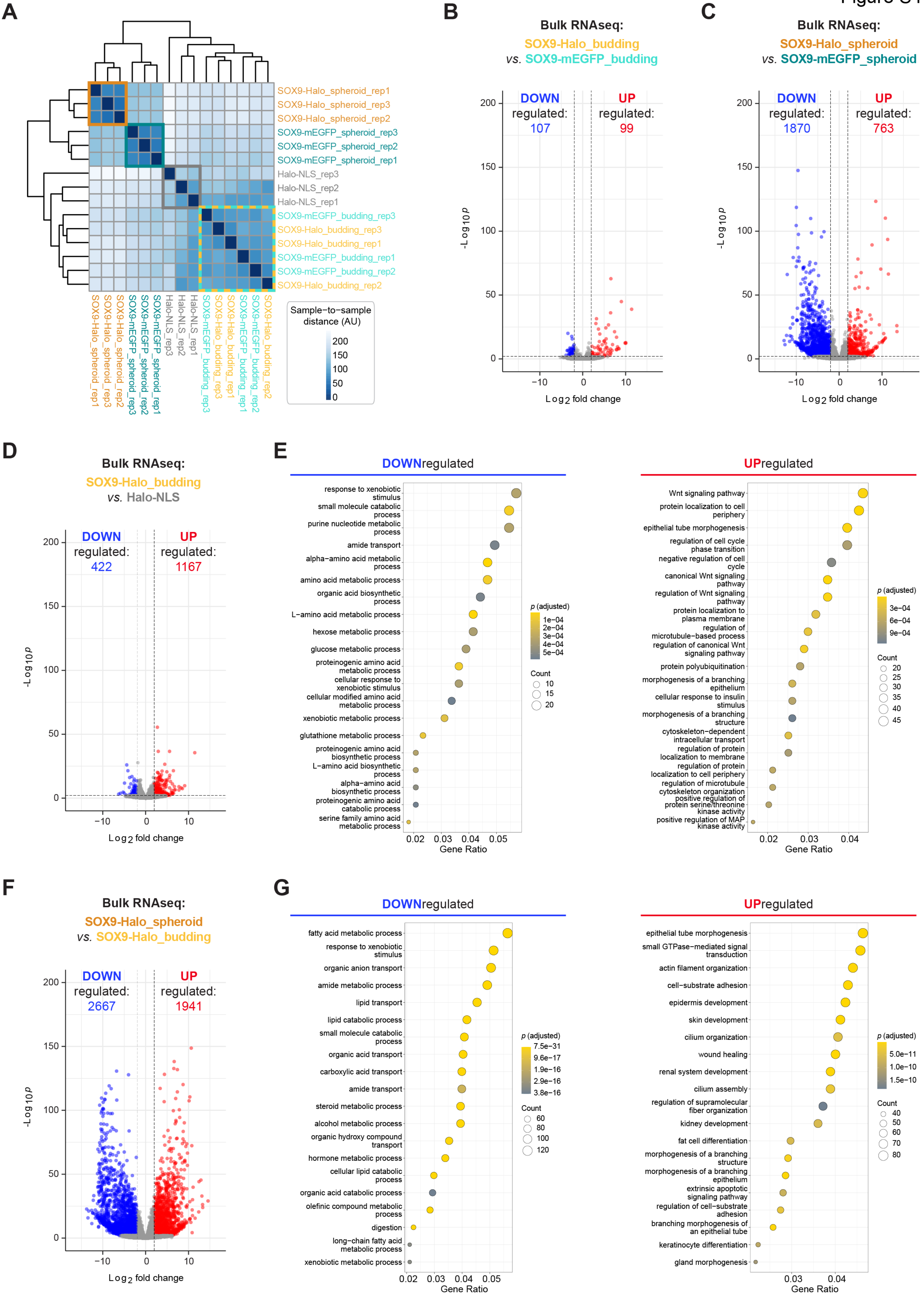
A
SOX9-Halo_spheroid_rep1
SOX9-Halo_spheroid_rep3
SOX9-Halo_spheroid_rep2
SOX9-mEGFP_spheroid_rep3
SOX9-mEGFP_spheroid_rep2
SOX9-mEGFP_spheroid_rep1
Halo-NLS_rep3
Halo-NLS_rep2
Halo-NLS_rep1
SOX9-mEGFP_budding_rep3
SOX9-Halo_budding_rep3
SOX9-Halo_budding_rep1
SOX9-mEGFP_budding_rep1
SOX9-mEGFP_budding_rep2
SOX9-Halo_budding_rep2
Sample−to−sample distance (AU)
B
Bulk RNAseq:
SOX9-Halo_budding vs. SOX9-mEGFP_budding
DOWN regulated: 107
UP regulated: 99
-Log10 p
Log2 fold change
C
Bulk RNAseq:
SOX9-Halo_spheroid vs. SOX9-mEGFP_spheroid
DOWN regulated: 1870
UP regulated: 763
D
Bulk RNAseq:
SOX9-Halo_budding vs. Halo-NLS
DOWN regulated: 422
UP regulated: 1167
E
DOWNregulated
response to xenobiotic stimulus
small molecule catabolic process
purine nucleotide metabolic process
amide transport
alpha−amino acid metabolic process
amino acid metabolic process
organic acid biosynthetic process
L−amino acid metabolic process
hexose metabolic process
glucose metabolic process
proteinogenic amino acid metabolic process
cellular response to xenobiotic stimulus
cellular modified amino acid metabolic process
xenobiotic metabolic process
glutathione metabolic process
proteinogenic amino acid biosynthetic process
L−amino acid biosynthetic process
alpha−amino acid biosynthetic process
proteinogenic amino acid catabolic process
serine family amino acid metabolic process
Gene Ratio
p (adjusted)
Count
UPregulated
Wnt signaling pathway
protein localization to cell periphery
epithelial tube morphogenesis
regulation of cell cycle phase transition
negative regulation of cell cycle
canonical Wnt signaling pathway
regulation of Wnt signaling pathway
protein localization to plasma membrane
regulation of microtubule−based process
regulation of canonical Wnt signaling pathway
protein polyubiquitination
morphogenesis of a branching epithelium
cellular response to insulin stimulus
morphogenesis of a branching structure
cytoskeleton−dependent intracellular transport
regulation of protein localization to membrane
regulation of protein localization to cell periphery
regulation of microtubule cytoskeleton organization
positive regulation of protein serine/threonine kinase activity
positive regulation of MAP kinase activity
F
Bulk RNAseq:
SOX9-Halo_spheroid vs. SOX9-Halo_budding
DOWN regulated: 2667
UP regulated: 1941
G
DOWNregulated
fatty acid metabolic process
response to xenobiotic stimulus
organic anion transport
amide metabolic process
lipid transport
lipid catabolic process
small molecule catabolic process
organic acid transport
carboxylic acid transport
amide transport
steroid metabolic process
alcohol metabolic process
organic hydroxy compound transport
hormone metabolic process
cellular lipid catabolic process
organic acid catabolic process
olefinic compound metabolic process
digestion
long−chain fatty acid metabolic process
xenobiotic metabolic process
UPregulated
epithelial tube morphogenesis
small GTPase−mediated signal transduction
actin filament organization
cell−substrate adhesion
epidermis development
skin development
cilium organization
wound healing
renal system development
cilium assembly
regulation of supramolecular fiber organization
kidney development
fat cell differentiation
morphogenesis of a branching structure
morphogenesis of a branching epithelium
extrinsic apoptotic signaling pathway
regulation of cell−substrate adhesion
branching morphogenesis of an epithelial tube
keratinocyte differentiation
gland morphogenesis

<u>Figure S4:</u> Enteroid reversion to a fetal-like state upon SOX9 overexpression is tag-independent and occurs through intermediate states with upregulated Wnt signaling-dependent pathways, related to Figure 4. (A) Sample-to-sample distances of the organoid samples Halo-NLS (gray), SOX9-mEGFP_budding (light turquoise), SOX9-Halo_budding (yellow), SOX9-mEGFP_spheroid (dark turquoise), and SOX9-Halo_spheroid (orange) in biological triplicates of bulk RNAseq experiments. **(B,C)** Volcano plot displaying DEGs (adjusted *p*-value ≤0.01, fold change ≥2 and mean counts ≥10; red/blue: up-/downregulated) in (B) SOX9-Halo_budding in comparison to SOX9-mEGFP_budding organoids or (C) SOX9-Halo spheroids in comparison to SOX9-mEGFP spheroids determined by bulk RNAseq. **(D)** Volcano plot displaying DEGs (adjusted *p*-value ≤0.01, fold change ≥2 and mean counts ≥10; red/blue: up-/downregulated) in SOX9-Halo_budding in comparison to Halo-NLS CTRL organoids determined by bulk RNAseq. **(E)** GO analysis for the top 20 biological pathways enriched in DEGs down- (left, blue) or upregulated (right, red) in SOX9-Halo_budding in comparison to Halo-NLS CTRL organoids with adjusted *p*-values and gene counts indicated. **(F)** Volcano plot displaying DEGs (adjusted *p*-value ≤0.01, fold change ≥2 and mean counts ≥10; red/blue: up-/downregulated) in SOX9-Halo spheroids in comparison to SOX9-Halo_budding organoids determined by bulk RNAseq. **(G)** GO analysis for the top 20 biological pathways enriched in DEGs down- (left, blue) or upregulated (right, red) in SOX9-Halo spheroids in comparison to SOX9-Halo_budding organoids with adjusted *p*-values and gene counts indicated. Data shown refers to the bulk RNAseq experiment from Fig. 4B-D and Fig. S5.

Figure S5

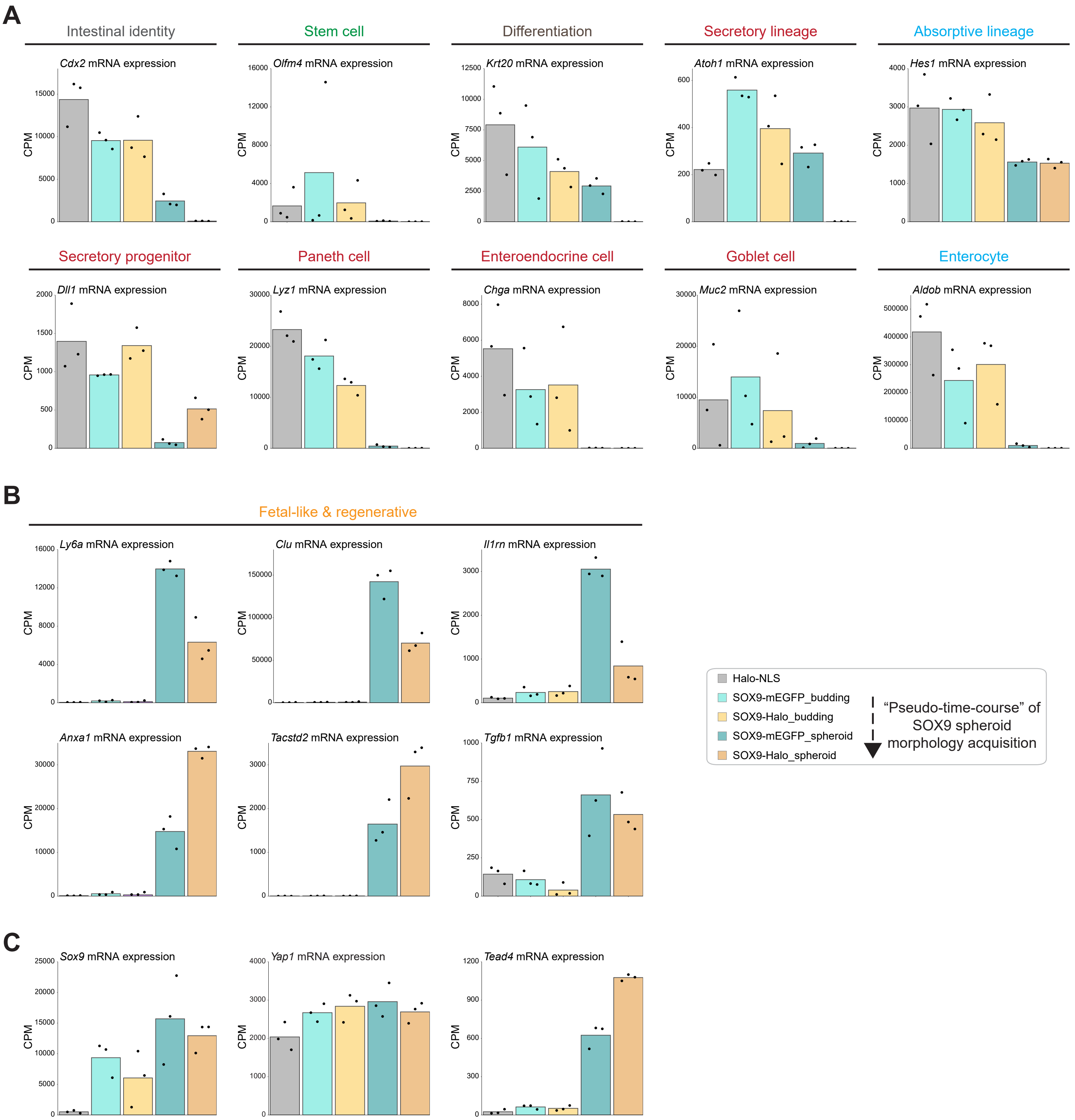
A
Intestinal identity
Cdx2 mRNA expression
Stem cell
Olfm4 mRNA expression
Differentiation
Krt20 mRNA expression
Secretory lineage
Atoh1 mRNA expression
Absorptive lineage
Hes1 mRNA expression
Secretory progenitor
Dll1 mRNA expression
Paneth cell
Lyz1 mRNA expression
Enteroendocrine cell
Chga mRNA expression
Goblet cell
Muc2 mRNA expression
Enterocyte
Aldob mRNA expression
B
Fetal-like & regenerative
Ly6a mRNA expression
Clu mRNA expression
Il1rn mRNA expression
Anxa1 mRNA expression
Tacstd2 mRNA expression
Tgfb1 mRNA expression
C
Sox9 mRNA expression
Yap1 mRNA expression
Tead4 mRNA expression
CPM
Halo-NLS
SOX9-mEGFP_budding
SOX9-Halo_budding
SOX9-mEGFP_spheroid
SOX9-Halo_spheroid
"Pseudo-time-course" of SOX9 spheroid morphology acquisition

__Figure S5:__ **A "pseudo-time-course" of spheroid morphology acquisition upon SOX9 overexpression in enteroids reveals a reduction in intestinal epithelial signatures across lineages counteracted by the induction of a gene expression program resembling fetal-like reversion, which includes the upregulation of *Tead* acting downstream of YAP, related to Figure 4. (A-C)** Selected DEGs in the stable organoid lines Halo-NLS (gray), SOX9-mEGFP_budding (light turquoise), SOX9-Halo_budding (yellow), SOX9-mEGFP_spheroid (dark turquoise), and SOX9-Halo_spheroid (orange) determined by bulk RNAseq in biological triplicates. The mean counts per million mapped reads (CPM) of three replicates (bar) and the CPMs for each replicate (points) are indicated. Samples are ordered according to passage number (time in culture) and degree of spheroid phenotype acquisition. (A) Intestinal identity, stem and differentiation markers of both secretory and absorptive lineages. (B) Regenerative fetal-like markers. (C) *Sox9*, *Yap1*, *Tead4*. Data shown refer to the bulk RNAseq experiment in Fig. 3F,G, Fig. 4B-D and Fig. S4.

Figure S6

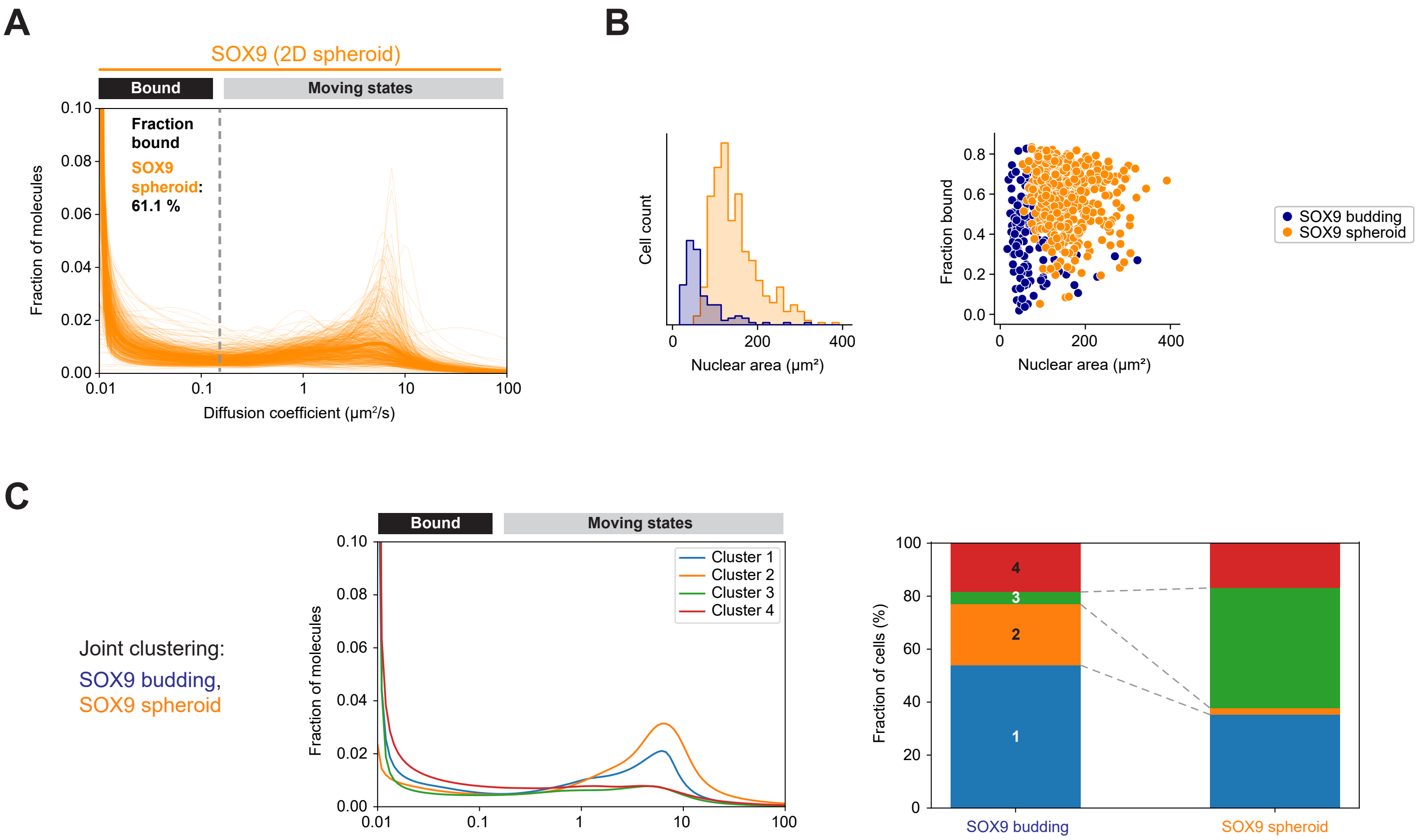

D

Joint clustering:
SOX9 budding,
SOX9 spheroid

Cluster	1	2	3	4
f bound	44.8%	22.2%	69.2%	62.5%
Modal *D* (μm²/s)	0.01	6.14	0.01	0.01

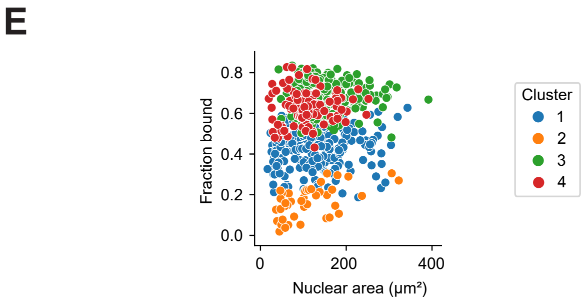

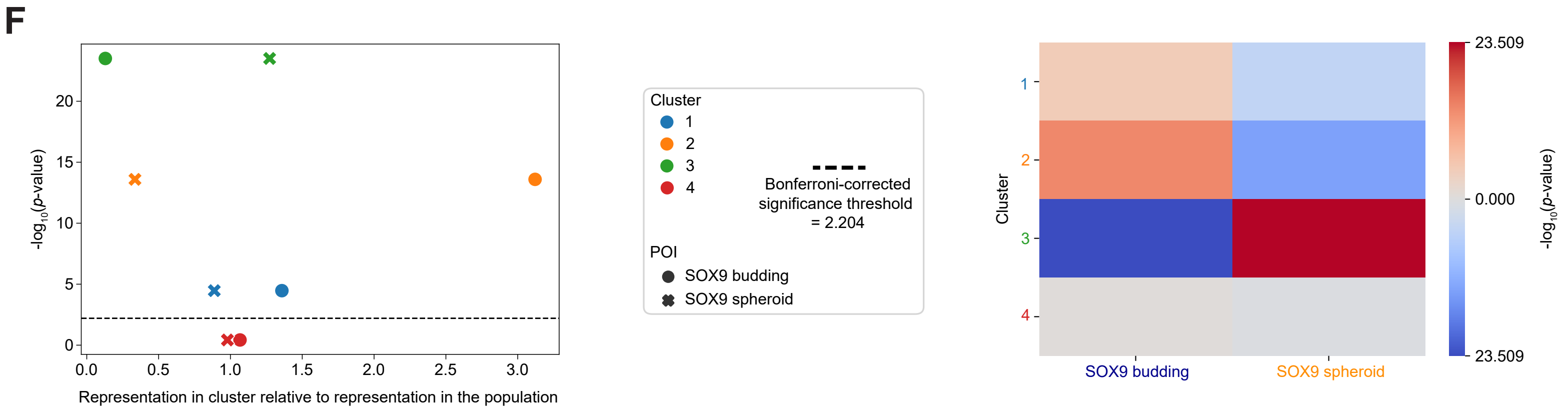

Figure S6: **Unlike in budding enteroids, a larger fraction of immobile SOX9-Halo molecules is present in spheroid-derived EMCs with larger nuclei, related to Figure 5. (A)** Single-cell diffusion spectra for 10 independent automated experiments for SOX9-Halo in spheroid-derived 2D EMCs. **(B)** Left: Nuclear area distribution for SOX9-Halo_budding (dark blue) and SOX9-Halo_spheroid (orange) cells determined from SMT data in 2D EMCs. Right: Single-cell correlation of SMT-derived fraction bound with nuclear area for SOX9-Halo_budding (dark blue) and SOX9-Halo_spheroid (orange). **(C-F)** Joint hierarchical clustering of SOX9-Halo_budding and SOX9-Halo_spheroid based on single-cell diffusion spectra using the Jensen-Shannon distance metric. (C) Left: Mean diffusion spectra of each cluster. Right: Distribution of cells from both SOX9-Halo samples into diffusion clusters. (D) Cluster statistics. (E) Fractions bound for each cell against the nuclear area with color-coded diffusion clusters. (F) Left: *p*-values indicating the representation of SOX9-Halo_budding (circle) and SOX9-Halo_spheroid (x mark) in each diffusion cluster relative to the representation in the population; Bonferroni-corrected significance threshold (dashed line). Right: Heatmap of *p*-values indicating the representation of each sample in each diffusion cluster (red: overrepresentation; blue: underrepresentation). Cluster: 1-blue, 2-orange, 3-green, 4-red. The SOX9-Halo_budding data are the same as in Fig. 1E-I, Fig. 2B-D, Fig. 5C, Fig. S1E,G,I, and Fig. S2A-G. The SOX9-Halo_spheroid data are the same as in Fig. 5B-D.

Figure S7

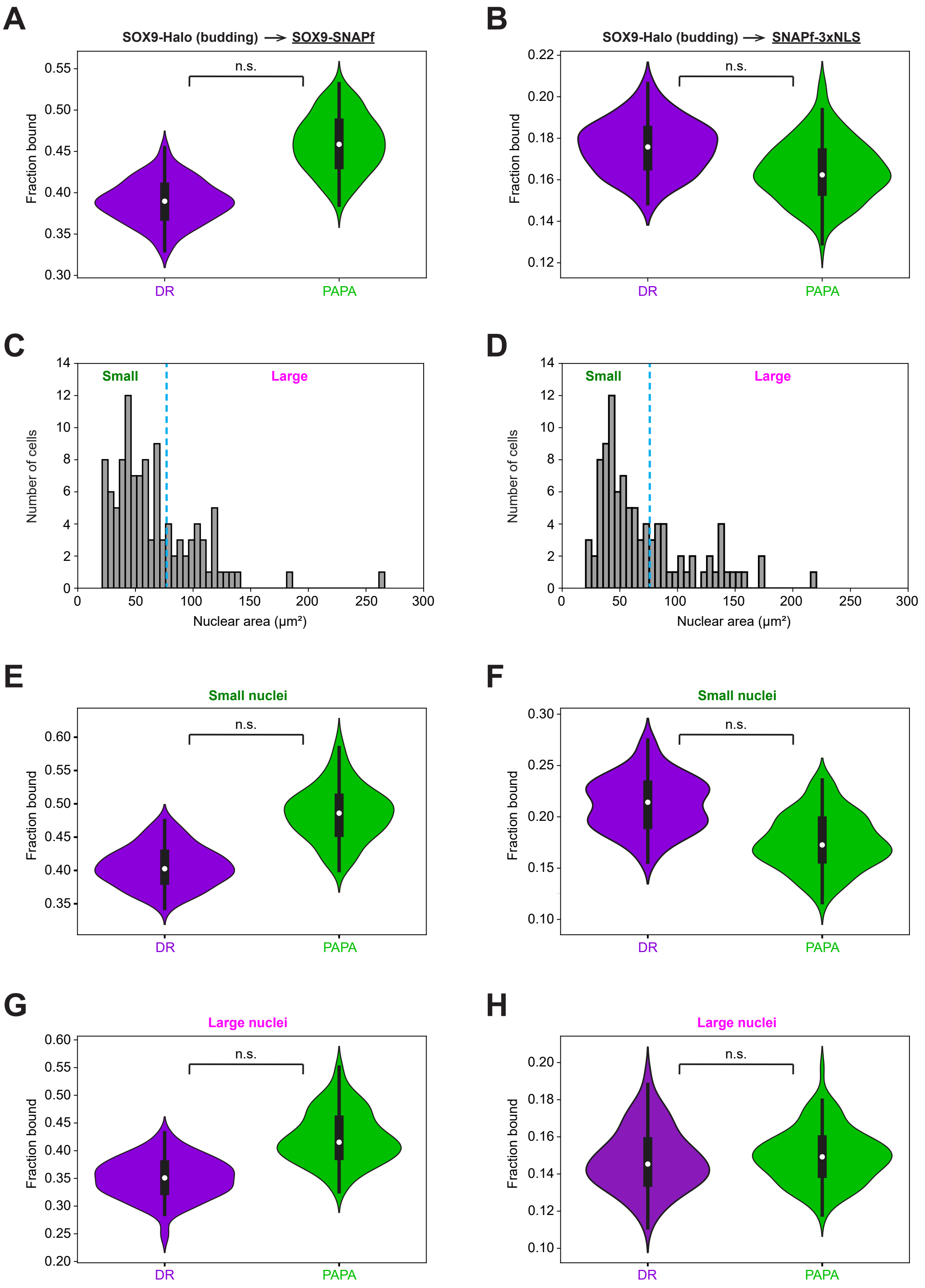

A
SOX9-Halo (budding) → SOX9-SNAPf
n.s.
Fraction bound
DR
PAPA
B
SOX9-Halo (budding) → SNAPf-3xNLS
n.s.
Fraction bound
DR
PAPA
C
Small
Large
Number of cells
Nuclear area (μm²)
D
Small
Large
Number of cells
Nuclear area (μm²)
E
Small nuclei
n.s.
Fraction bound
DR
PAPA
F
Small nuclei
n.s.
Fraction bound
DR
PAPA
G
Large nuclei
n.s.
Fraction bound
DR
PAPA
H
Large nuclei
n.s.
Fraction bound
DR
PAPA

Figure S7: PAPA-SMT reveals a chromatin-bound pool of self-associated SOX9 in live 2D EMCs derived from budding SOX9-Halo enteroids, related to Figure 6. **(A,B)** PAPA experiments in 2D EMCs derived from SOX9-Halo_budding organoids. Violin plots for fractions bound (median: white point; first/third quartile: whiskers) of SOX9-Halo→SOX9-SNAPf (A) and SOX9-Halo→SNAPf-3xNLS (B) determined from DR/PAPA (purple/green) trajectories. Data in (A) are from 6 combined experiments with *n*=108 cells (*n*=6,25,32,7,29,9 cells; 1661 DR and 1191 PAPA trajectories; bootstrapped fractions bound: 38.9±4.9% (DR) and 46.0±6.6% (PAPA)). Data in (B) are from 5 combined experiments with *n*=97 cells (*n*=22,44,2,14,15 cells; 3579 DR and 2644 PAPA trajectories; bootstrapped fractions bound: 17.6±2.5% (DR) and 16.4±2.8% (PAPA)). **(C,D)** Histograms of the nuclear area of cells from (A,B) with indicated threshold of 75 μm^2 (blue dotted line) used to split the whole cell population into two subpopulations with small or large nuclei. **(E-H)** PAPA results on subpopulations of cells with (E,F) small (*n*=148 or *n*=126 cells, respectively) or (G,H) large nuclei (*n*=68 cells for both conditions) from the experiments shown in (A,B). Bootstrapped fractions bound: (E) – 40.5±5.9% (DR) and 48.6±7.9% (PAPA); (F) – 21.3±5.1% (DR) and 17.6±5.2% (PAPA); (G) – 35.0±7.0% (DR) and 42.3±9.1% (PAPA); (H) – 14.8±3.2% (DR) and 15.0±2.8% (PAPA). (n.s.) p>0.05. For statistical details see experimental procedures.

Figure S8

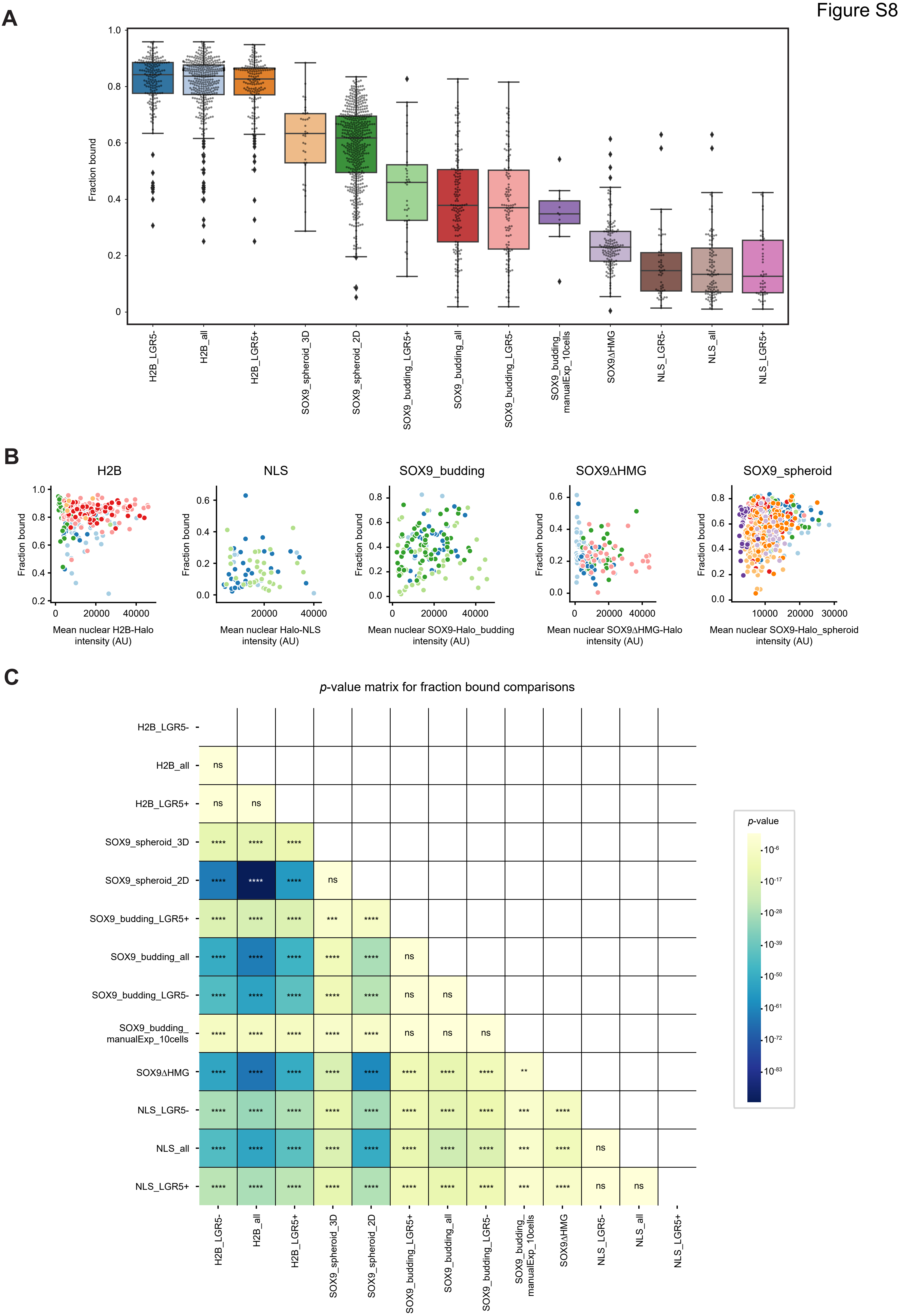
A
Fraction bound
1.0
0.8
0.6
0.4
0.2
0
H2B_LGR5-
H2B_all
H2B_LGR5+
SOX9_spheroid_3D
SOX9_spheroid_2D
SOX9_budding_LGR5+
SOX9_budding_all
SOX9_budding_LGR5-
SOX9_budding_manualExp_10cells
SOX9ΔHMG
NLS_LGR5-
NLS_all
NLS_LGR5+
B
H2B
NLS
SOX9_budding
SOX9ΔHMG
SOX9_spheroid
Fraction bound
Mean nuclear H2B-Halo intensity (AU)
Mean nuclear Halo-NLS intensity (AU)
Mean nuclear SOX9-Halo_budding intensity (AU)
Mean nuclear SOX9ΔHMG-Halo intensity (AU)
Mean nuclear SOX9-Halo_spheroid intensity (AU)
C
p-value matrix for fraction bound comparisons
H2B_LGR5-
H2B_all ns
H2B_LGR5+ ns ns
SOX9_spheroid_3D **** **** ****
SOX9_spheroid_2D **** **** **** ns
SOX9_budding_LGR5+ **** **** **** *** ****
SOX9_budding_all **** **** **** **** **** ns
SOX9_budding_LGR5- **** **** **** **** **** ns ns
SOX9_budding_manualExp_10cells **** **** **** **** **** ns ns ns
SOX9ΔHMG **** **** **** **** **** **** **** **** **
NLS_LGR5- **** **** **** **** **** **** **** **** *** ****
NLS_all **** **** **** **** **** **** **** **** *** **** ns
NLS_LGR5+ **** **** **** **** **** **** **** **** *** **** ns ns
p-value
10^{-6}
10^{-17}
10^{-28}
10^{-39}
10^{-50}
10^{-61}
10^{-72}
10^{-83}

Figure S8: **Fraction bound distributions and comparisons. (A)** Single-cell fraction bound distributions for all POI-Halo conditions measured by fast SMT in intestinal organoid models in this study. Boxplots with the fraction bound for each cell plotted (gray line: median; whiskers: first/third quartile). **(B)** Single-cell fraction bound distributions across experiments determined by fast SMT in 2D EMCs are plotted against the mean nuclear POI-Halo intensity for POI-Halo conditions H2B, NLS, SOX9_budding, SOX9ΔHMG, and SOX9_spheroid (left to right) with cells differentially colored by experiment. **(C)** Significance matrix of pairwise comparisons of fractions bound of all POI-Halo samples acquired by fast SMT in intestinal organoid models in this study ((ns) non-significant, $p>0.05$; (**) $p\leq0.01$; (***) $p\leq0.001$; (****) $p\leq0.0001$; *p*-value color-coded). Mann-Whitney U-tests were performed to compare fraction bound distributions between two conditions. The Benjamini–Hochberg procedure was used to correct for multiple comparisons.

Figure S9

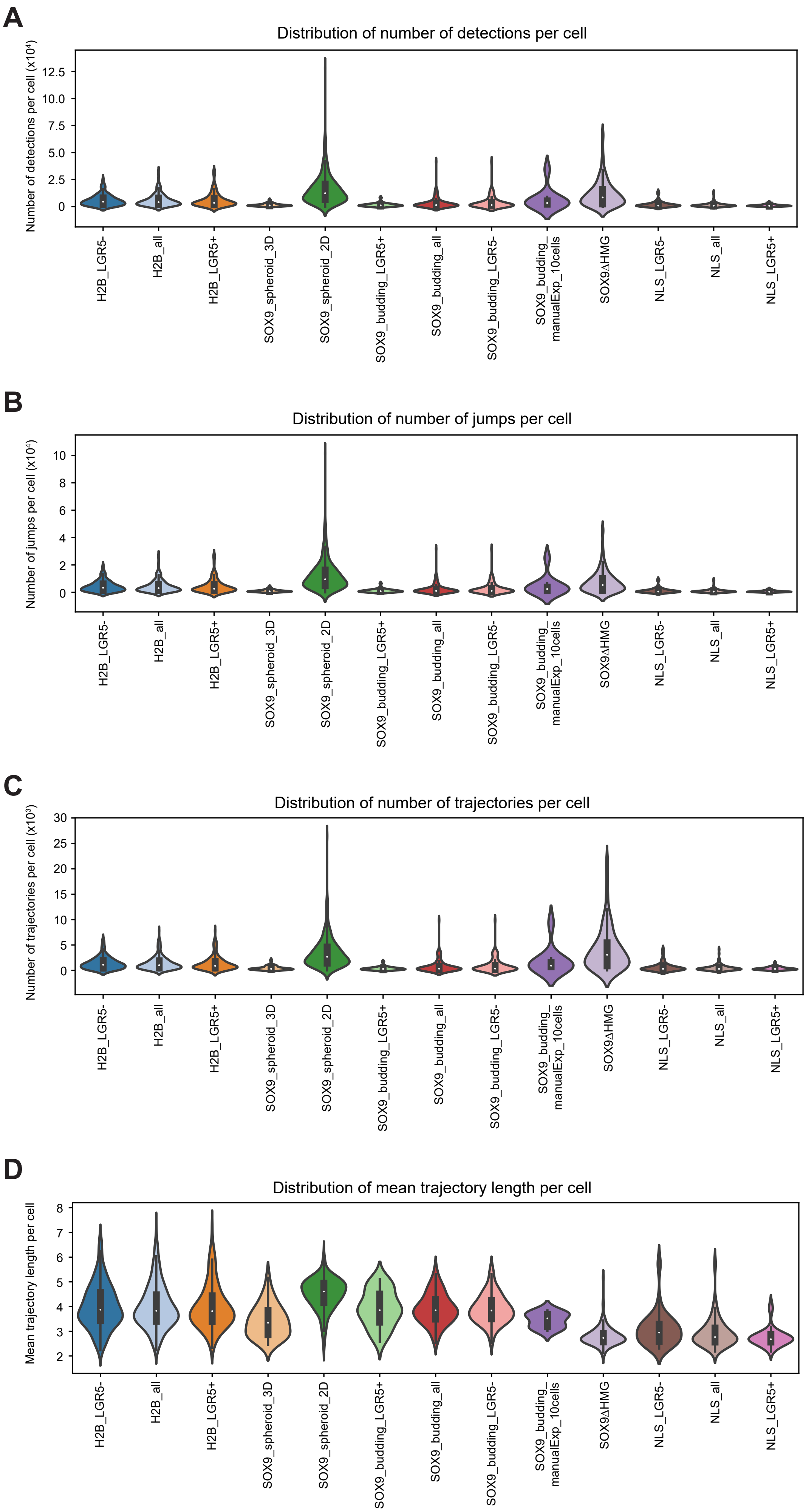
A
Distribution of number of detections per cell
Number of detections per cell (x10^4)
12.5
10.0
7.5
5.0
2.5
0
B
Distribution of number of jumps per cell
Number of jumps per cell (x10^4)
10
8
6
4
2
0
C
Distribution of number of trajectories per cell
Number of trajectories per cell (x10^3)
30
25
20
15
10
5
0
D
Distribution of mean trajectory length per cell
Mean trajectory length per cell
8
7
6
5
4
3
2
H2B_LGR5-
H2B_all
H2B_LGR5+
SOX9_spheroid_3D
SOX9_spheroid_2D
SOX9_budding_LGR5+
SOX9_budding_all
SOX9_budding_LGR5-
SOX9_budding_
manualExp_10cells
SOX9ΔHMG
NLS_LGR5-
NLS_all
NLS_LGR5+

<u>Figure S9:</u> Statistics for single-molecule detection and tracking across fast SMT conditions. Violin plots for the distribution of **(A)** the number of detections, **(B)** the number of jumps, **(C)** the number of trajectories, and **(D)** the mean trajectory length per cell for each POI-Halo sample measured by fast SMT in intestinal organoid models in this study (white point: median; whiskers: first/third quartile).

Figure S10

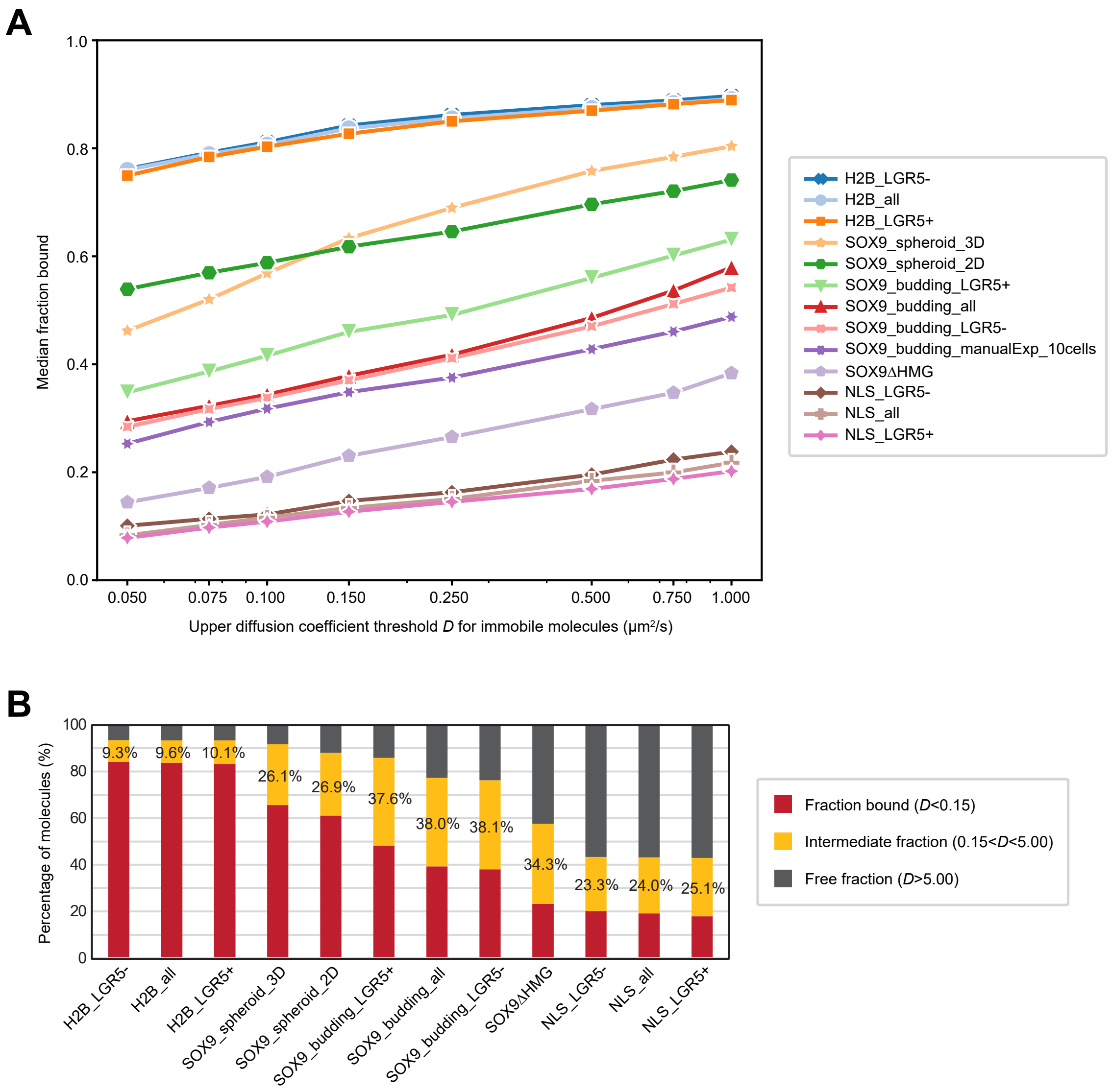

A
Median fraction bound
Upper diffusion coefficient threshold D for immobile molecules (μm²/s)
0.0
0.2
0.4
0.6
0.8
1.0
0.050
0.075
0.100
0.150
0.250
0.500
0.750
1.000
H2B_LGR5-
H2B_all
H2B_LGR5+
SOX9_spheroid_3D
SOX9_spheroid_2D
SOX9_budding_LGR5+
SOX9_budding_all
SOX9_budding_LGR5-
SOX9_budding_manualExp_10cells
SOX9ΔHMG
NLS_LGR5-
NLS_all
NLS_LGR5+
B
Percentage of molecules (%)
0
20
40
60
80
100
9.3%
9.6%
10.1%
26.1%
26.9%
37.6%
38.0%
38.1%
34.3%
23.3%
24.0%
25.1%
H2B_LGR5-
H2B_all
H2B_LGR5+
SOX9_spheroid_3D
SOX9_spheroid_2D
SOX9_budding_LGR5+
SOX9_budding_all
SOX9_budding_LGR5-
SOX9ΔHMG
NLS_LGR5-
NLS_all
NLS_LGR5+
Fraction bound (D<0.15)
Intermediate fraction (0.15<D<5.00)
Free fraction (D>5.00)

Figure S10: **Immobile and freely diffusing fractions are the primary determinants of POI-Halo diffusion behavior. (A)** Median fractions bound for each POI-Halo sample measured by fast SMT in intestinal organoid models in this study using various diffusion coefficients D (0.05 – 1.00 $\mu m^2/s$) as the upper limit (exclusive) for molecules considered as immobile and thus accounted for in the fraction bound. **(B)** Distribution of molecules into immobile ($D < 0.15$ $\mu m^2/s$; red), slowly diffusing (0.15 $\mu m^2/s < D < 5$ $\mu m^2/s$; yellow) and freely diffusing ($D > 5$ $\mu m^2/s$; gray) fractions for all POI-Halo samples measured by fast SMT in intestinal organoid models in this study. The percentage of molecules present in the intermediate fraction is indicated.

Supplemental methods

Supplemental experimental procedures

Cloning of DNA constructs

For the production of lentivirus to deliver protein of interest (POI)-tag transgenes into organoids, the third generation lentiviral pHAGE vector originally developed in the lab of Richard Mulligan (Murphy et al., 2006) was used together with the second generation lentiviral packaging plasmid psPAX2 (gift from Didier Trono (Addgene plasmid #12260; http://n2t.net/addgene:12260; RRID:Addgene_12260)) and the VSV-G envelope expressing plasmid pMD2.G (gift from Didier Trono (Addgene plasmid #12259; http://n2t.net/addgene:12259; RRID:Addgene_12259)). For constitutive expression of POI-tag under the weak L30 promoter, pHAGE L30 IRES Puro (Walther et al., 2024), pHAGE L30 IRES Zeo, and pHAGE L30 IRES Neo backbones were created. Transgenes encoding the POI-tags H2B-Halo-V5 (Walther et al., 2024), V5-Halo-NLS (Walther et al., 2024), mSOX9-Halo-V5, and mSOX9-mEGFP-V5 were cloned into the Puro vector, H2B-mEGFP-V5 was cloned into the Zeo vector, and H2B-mScarletI-V5 was cloned into the Neo vector. For mSOX9, the DNA sequence encoding the reference 507aa isoform of UniProt entry Q04887 was cloned (NCBI Gene ID 20682, NM_011448.4). The final pHAGE constructs were confirmed by Sanger sequencing.

For the preparation of crude rAAV to deliver POI-tag encoding transgenes transiently into 2D EMCs, expression cassettes of L30prom_mSOX9deltaHMG-NLS-Halo-V5 or L30prom_mSOX9-SNAPf-ALFA (Götzke et al., 2019) were cloned in a pAAV vector derived from pAAV.CMV.Luc.IRES.EGFP.SV40 (gift from James M. Wilson (Addgene plasmid #105533; http://n2t.net/addgene:105533)) to create *cis* plasmids. The helper plasmid pAdDeltaF6 (gift from James M. Wilson (Addgene plasmid #112867; http://n2t.net/addgene:112867)) and the *trans* rep-cap AAV-KP1 plasmid (gift from Mark Kay (Addgene plasmid #206504; http://n2t.net/addgene:206504; (Pekrun et al., 2019)) were used together with a *cis* plasmid. pAAV bacterial cultures were grown at 30°C and the final rAAV constructs were confirmed by whole plasmid sequencing.

For further details, the plasmid maps are available on GitLab (https://gitlab.com/nikewalther/walther_sox9organoid_2025/-/tree/main/PlasmidMaps?ref_type=heads).

Lentivirus preparation

Lentivirus preparation using Hek293T Lenti-X cells (Takara, cat.# 632180) including lentivirus concentration using Lenti-X concentrator (Takara, cat.# 631231) was performed as described (Walther et al., 2024).

Generation of crude rAAV vector preparations

The generation of crude rAAV vector preparations was based on the protocol described (Benyamini et al., 2023). In brief, $3x10^5$ Hek293T cells (CRL-3216, ATCC) were seeded per well of a 6-well plate (Thermo Fisher Scientific, cat.# 140675) in high-glucose Dulbecco's modified eagle medium (DMEM) containing GlutaMAX-I (Gibco, cat.# 2537044), supplemented with 10% (vol/vol) fetal bovine serum (FBS; HyClone, cat.# SH30910.03, LOT# AXJ47554), and 1 mM sodium pyruvate (Gibco, cat.# 11360070), and grown to 75-90% confluency. For transfection of a 6-well, a plasmid mixture of 1.3 µg *trans* rep-cap AAV-KP1 plasmid, 2.6 µg helper plasmid pAdDeltaF6, and 1.3 µg gene of interest (GOI)-encoding *cis* plasmid (see "Cloning of DNA constructs") was prepared in serum-free DMEM to 100 µL per well. After addition of 5.2 µL 1 µg/µL polyethylenimine (PEI) hydrochloride MAX (Polysciences, cat.# 24765-1) in distilled H_2O (pH 7.1, filter sterilized), the mixture was pulsed 10-15x on a vortexer. Following incubation for 15 min at RT, 1.9 mL serum-free DMEM was added and mixing was performed by gently pipetting up and down two times. After removal of the culture medium, 2 mL of transfection mixture was added per well and the plate was incubated at 37°C and 5% CO_2 for 72 h. The 6-well plate was frozen at -80°C for 30 min, followed by thawing at 37°C for 30 min. Freezing and thawing was repeated for a total of three cycles. Each well was mixed by pipetting and the cell lysate was transferred to a 2 mL tube. To remove cell debris, centrifugation was performed at 15,000 x *g* for 15 min at RT. The supernatant (SN) was removed and transferred to a new 2 mL tube. This crude rAAV vector preparation was stored at 4°C for several weeks before being used for transduction of 2D EMCs.

Mouse small intestinal crypt preparation for growing small intestinal organoids

Mouse small intestinal crypt preparations for growing small intestinal organoids were performed as described (Walther et al., 2024) with the exception that female mice at an age of 8-16 weeks were used for this study.

Mouse small intestinal organoid culture

Mouse small intestinal organoids were grown in Matrigel domes (90% (vol/vol) Matrigel Matrix for Organoid Culture (Corning, cat.# 08774406)) and cultured in IntestiCult Organoid Growth Medium (Mouse) (Stem Cell Technologies, cat.# 06005) supplemented with 1% (vol/vol) Penicillin/Streptomycin (Pen/Strep; Gibco, cat.# 15070063) as described (Walther et al., 2024). mSIOs were passaged every week using Gentle Cell Dissociation Reagent (Stem Cell Technologies, cat.# 100-0485) with one to two media changes in between depending on mSIO density except for SOX9 spheroids which required at least two media changes due to their increased growth/proliferation rate.

Preparation of Wnt3a-conditioned medium

Wnt3a-conditioned medium was prepared as described before (Walther et al., 2024) using L-Wnt3a cells (CRL-2647, ATCC) and Advanced DMEM/F-12 (ADMEM; Gibco, cat.# 12634028) supplemented with 10% (vol/vol) FBS, 1% (vol/vol) GlutaMAX (Gibco, cat.# 35050061), 10 mM HEPES (Sigma-Aldrich, cat.# H0887-20mL), 1 mM N-acetyl-L-cysteine (Sigma-Aldrich, cat.# A9165-5G; 500 mM stock in ddH_2O, sterile-filtered).

Lentiviral transduction of mouse small intestinal organoids and selection of stable organoid lines

Lentiviral transduction of mSIOs was performed as described before (Walther et al., 2024). In brief, mSIOs were seeded in pre-transduction medium consisting of 50% (vol/vol) IntestiCult without Pen/Strep and 50% (vol/vol) Wnt3a-conditioned medium, supplemented with 100 mM nicotinamide (Sigma, cat.# N0636-100G; 1M stock in ddH_2O), 10 µM ROCK inhibitor Y-27632 (Stem Cell Technologies, cat.# 72304; 10 mM stock in ddH_2O), and 2.5 µM CHIR99021 (Stem Cell Technologies, cat.# 72052; 10 mM stock in dimethylsulfoxide (DMSO; Sigma-Aldrich, cat.# D2650)). 4-5 d post-seeding, mSIOs were broken down into single cells using TrypLE Express (Thermo Scientific, cat.# 12604013) prior to spinoculation (1 h at 37°C and 600 g) with lentivirus

in pre-transduction medium containing 10 µg/mL polybrene Millipore Sigma, cat.# TR-1003-G) and incubation for 6 h at 37°C. Transduced cells were seeded in 90% (vol/vol) Matrigel Matrix for Organoid Culture in IntestiCult Organoid Growth Medium supplemented with 1% (vol/vol) Pen/Strep and grown in pre-transduction medium. 2-3 d post-transduction, the medium was exchanged to pre-transduction medium containing selection antibiotics (2 µg/mL puromycin (Thermo Scientific, cat.# A11138-03)) and the selection medium was exchanged every 2-3 d until large, selected spheroids were obtained. Upon passaging, selected spheroids were cultured in IntestiCult supplemented with 10 µM ROCK inhibitor Y-27632 and 2.5 µM CHIR99021 including selection antibiotics. After 2-3 d the ROCK inhibitor was removed, followed by the removal of CHIR99021 after another 2-3 d to culture selected stable organoid lines as described before including selection antibiotics.

Established stable mSIO lines were cryopreserved in CryoStor CS10 (Stem Cell Technologies, cat.# 07931) as described before (Walther et al., 2024) and stored in liquid nitrogen.

Except for SOX9 spheroids and when specifically noted otherwise, mSIOs were used for experiments until 8 weeks after line establishment or an organoid aliquot cryopreserved at an early passage was thawn as described before (Walther et al., 2024).

For stable SOX9-Halo or SOX9-mEGFP organoids (WT background), a second transduction was performed at P15(P12) according to the same protocol to achieve double stable organoid lines co-expressing H2B-mEGFP or H2B-mScarletI, whereby antibiotic selection was performed using 40 µg/mL Zeocin (Fisher Scientific, cat.# NC9002627) or 200 µg/mL Geneticin (Thermo Fisher Scientific, cat.# 10131027; neomycin resistance), respectively.

Brightfield imaging of organoid lines and 2D enteroid monolayer cultures

Brightfield (BF) imaging of mSIOs or 2D EMCs growing on 24-well culture plates (Corning, cat.# 353047) or 8-well Labteks II #1.5 (Nunc, cat.# 12-565-338) to record 3D/2D morphology was performed on an EVOS M5000 Imaging System (Invitrogen) microscope using 10x (Fig. 3A) or 4x (Fig. 4A) air objectives 5 d post-seeding.

Confocal imaging of SOX9-Halo organoid lines

For confocal imaging of SOX9-Halo organoid lines, POI-Halo organoids were seeded in 10-20 µL 90% Matrigel in IntestiCult droplets into 8-well Labteks II #1.5 (Nunc, cat.# 12-565-338) and overlaid with 500 µL IntestiCult. After 3 d the medium was exchanged. 5 d post-seeding, organoids were stained with 500 nM HTL-JF635 (kind gift from Luke Lavis) in DMEM/F-12 without phenol red (Gibco, cat.# 11039-021) for at least 1 h and imaged 1-6 h after staining.

Confocal imaging of 3D mSIOs was performed on a Yokogawa CSU-W1 SoRa spinning disk with a Nikon Ti2 inverted microscope operated by the NIS Elements AR 5.42.03 software (Nikon). The microscope was equipped with a temperature- and CO_2-controlled incubation chamber (Okolab), and the temperature was set to 37°C and the CO_2 to 5% for imaging of live organoids. For combined BF/SOX9-Halo organoid images (Fig. 1C, Fig. S3A), acquisition was performed using an Apo LWD 40× WI λS DIC N2 water immersion objective (N.A. 1.15; Nikon). For imaging SOX9-Halo-JF635 and LGR5-DTR-GFP (budding; Fig. 1C) or H2B-mEGFP (spheroid; Fig. S3A), JF635 was excited with 640 nm (laser at 80% (budding) or 40% (spheroid)) and detected with Hamamatsu Orca Flash 4.0 cameras at 200 ms exposure using a 561 nm long-pass (LP) filter. Consecutively, the widefield fluorescence imaging modality was used for BF detection with a Hamamatsu Orca Flash 4.0 camera at 100 ms exposure. For each modality, multiple *z* planes (*xy* pixel size: 0.1625 × 0.1625 µm; *z* interval 0.4 µm, number of *z* slices varying depending on the thickness of organoids) were imaged using a NIDAQ *z* piezo. One selected *z* plane is shown in Fig. 1C and Fig. S3A.

Generation of 2D enteroid monolayer cultures from 3D mouse small intestinal organoids and seeding on imaging dishes

2D EMCs were derived from 3D mSIOs as described (Sanman et al., 2020; Walther et al., 2024). In brief, a 1:40 (vol:vol) mixture of Matrigel Growth Factor Reduced Basement Membrane Matrix (Corning, cat.# 356231) in DMEM/F-12 without phenol red was used for coating imaging dishes by incubation for at least 30 min up to one week in the cell culture incubator prior to seeding. 100-500 µL of $1x10^6$ cells/mL mSIO-derived single-cell suspensions were seeded in plating medium (IntestiCult containing 20 µM ROCK inhibitor and 3 µM CHIR) to achieve a total of 500 µL/well of an 8-well

Labtek II #1.5. 1-2 d after seeding and every other day thereafter the medium was exchanged to IntestiCult.

Confocal imaging of POI-Halo enteroid monolayer cultures

2D EMCs were seeded and cultured as described above. 5 d post-seeding, EMCs were stained with 200 nM HTL-JFX549 (kind gift from Luke Lavis) in DMEM/F-12 without phenol red for 30 min. After two washes with DMEM/F-12 without phenol red for 15 min each, the medium was exchanged one more time to DMEM/F-12 without phenol red before proceeding with imaging.

Confocal imaging of live 2D EMCs (Fig. 1C, Fig. S3A) was performed on an LSM900 Airyscan 2 laser-scanning microscope with an inverted Axio Observer.Z1 / 7 operated by the ZEN 3.1 blue software (ZEISS). The microscope was equipped with a temperature- and CO_2-controlled incubation chamber (Zeiss/PeCon), and the temperature was set to 37°C and the CO_2 to 5%. Images were acquired using a Plan-Apochromat 40×/N.A. 1.3 Oil DIC (UV) VIS-IR M27 oil-immersion objective (ZEISS). One *z* plane was imaged consecutively in the 561 nm and 488 nm channels (*xy* pixel size: 0.092 × 0.092 µm; 1.21 µs pixel dwell time; bidirectional scanning; 4-times averaging). JFX549 was excited with 561 nm (diode (SH) laser at 2.0%) and detected with GaAsP (spectral gallium arsenide) detectors at 566-635 nm. Green fluorescence was excited with 488 nm (diode laser at 4.5% for LGR5-DTR-GFP (Fig. 1C) or 0.1% for H2B-mEGFP (Fig. S3A)) and detected with a multialkali-photomultiplier (MA-PMT) detector at 410-545 nm.

Images acquired as described above were also used for SOX9-Halo transgene expression level quantification in SOX9 budding organoids *versus* spheroids (Fig. 3C, right).

For confocal imaging of rAAV-transduced conditions, 2D EMCs were prepared and stained as described under “Transient transduction of 2D enteroid monolayer cultures using crude rAAV vector preparations for SMT and PAPA-SMT” and imaged on a spinning disk confocal microscope described under “Confocal imaging of SOX9-Halo organoid lines” using an Apo LWD 40× WI λS DIC N2 water immersion objective (N.A. 1.15; Nikon). For imaging SOX9ΔHMG-Halo-JFX650 (Fig. S1D), JFX650 was excited with 640 nm (laser at 40%) and detected with a Hamamatsu Orca Flash 4.0 camera at 500 ms exposure using a 640 nm LP filter. For imaging SOX9-Halo-JFX549/SOX9-

SNAPf-JFX650 (Fig. 6C) and SOX9-Halo-JFX549/JFX650-SNAPf-3xNLS (Fig. 6D), JFX650 was excited with 640 nm (laser at 50%) and JFX549 was excited with 561 nm (laser at 50%). Fluorescence was detected with Hamamatsu Orca Flash 4.0 cameras at 300 ms exposure using a 640 nm LP filter. Multiple *z* planes (*xy* pixel size: 0.1625 × 0.1625 µm; 21 *z* slices at *z* interval 0.4 µm) were imaged using a NIDAQ *z* piezo. One selected *z* plane is shown in Fig. S1D and Fig. 6C,D.

For confocal imaging of cell division phenotypes in 2D EMCs derived from SOX9-Halo spheroids co-expressing H2B-mEGFP (Fig. S3B), EMCs were prepared as described above. Instead of SOX9-Halo, staining for microtubules was performed by adding 500 nM SiR-tubulin and 10 µM Verapamil (SiR-tubulin kit; Cytoskeleton, cat.# CY-SC002) in DMEM/F-12. After incubation at 37°C for 1 h, live imaging was performed on a spinning disk confocal microscope described under "Confocal imaging of SOX9-Halo organoid lines" using an Apo LWD 40× WI λS DIC N2 water immersion objective (N.A. 1.15; Nikon). SiR-tubulin was excited with 640 nm (laser at 50%) and H2B-mEGFP was excited with 488 nm (laser at 5%). Fluorescence was detected with Hamamatsu Orca Flash 4.0 cameras at 200 ms exposure using a 561 nm LP filter. Multiple *z* planes (*xy* pixel size: 0.1083 × 0.1083 µm; 37 *z* slices at *z* interval 0.3 µm) were imaged using a NIDAQ *z* piezo. One selected *z* plane is shown in Fig. S3B.

IF of 2D enteroid monolayer cultures and confocal imaging

IF of 2D EMCs and confocal imaging was performed 5 d post-seeding as described (Walther et al., 2024). In brief, EMCs were fixed with 4% paraformaldehyde (PFA; Electron Microscopy Sciences, cat.# EMS14710) in PBS for 20 min at RT, washed three times with PBS for 5 min and stored at 4°C or directly subjected to IF staining. Permeabilization was performed with 0.5% Triton X-100 (TX-100; Sigma-Aldrich, cat.# T9284) in PBS for 1 h. Following three washes for 5 min each with PBS, blocking was performed in 3% (vol/vol) donkey serum (Sigma-Aldrich, cat.# D9663) in 0.1% (vol/vol) TX-100 in PBS (blocking buffer) for 4 h at RT or ON at 4°C. Incubation with the primary antibody (AB) in blocking buffer was performed ON at 4°C in a humidified chamber. Following three washes in blocking buffer for 5 min each, incubation with the secondary AB was performed for 1 h at RT. Following three washes with PBS for 5 min each, DNA was stained with 1 µg/mL Hoechst 33342 (Thermo Scientific, cat.# H3570) in PBS for 10-15 min, followed by another three washes with PBS for 5 min

each. Immunostained samples were either imaged directly or sealed with parafilm and stored for short-term at 4°C prior to imaging.

The following primary ABs and dilutions were used: rabbit anti-SOX9 (Abcam, cat.# ab185966; 1:200), rabbit anti-KI67 (Abcam, cat.# ab16667; 1:200), rabbit anti-OLFM4 (Cell Signaling, cat.# 39141; 1:50), rabbit anti-Lysozyme (Agilent, cat.# A009902-2; 1:300), sheep anti-DLL1 (Biotechne, cat.# AF3970; 1:20), rabbit anti-Aldolase B/C (Abcam, cat.# ab75751; 1:50), rabbit anti-SCA-1 (Abcam ab124688, cat.# ab124688; 1:50), rabbit anti-YAP (Cell Signaling, cat.# 14074S; 1:100), rabbit anti-ß-catenin (Abcam, cat.# ab32572; 1:250), rabbit anti-E-Cadherin (Abcam, cat.# ab40772; 1:1000), rabbit anti-EpCAM (Thermo Fisher Scientific, cat.# MA5-35283; 1:100). A secondary donkey anti-rabbit-AlexaFluor568 AB (Thermo Fisher Scientific, cat.# A10042) was used for all conditions except for DLL1 for which a donkey anti-sheep-AlexaFluor568 AB (Thermo Fisher Scientific, cat. #A21099) was used. For F-actin staining, incubation with a 1x phalloidin-AlexaFluor568 (Thermo Fisher Scientific, cat.# A12380) staining solution in PBS was performed for 1 h at RT instead of incubation with primary/secondary ABs.

Confocal imaging of immunostained monolayers (Fig. 3B; Fig. 4E; Fig. S3C) was performed on an LSM900 Airyscan 2 laser-scanning microscope with an inverted Axio Observer.Z1 / 7 operated by the ZEN 3.1 blue software (ZEISS) at RT. Images were acquired using a Plan-Apochromat 40×/ N.A. 1.3 Oil DIC (UV) VIS-IR M27 oil-immersion objective (ZEISS). One *z* plane was imaged in the 561 nm and 405 nm channels (*xy* pixel size: 0.11 × 0.11 µm; 2.06 µs pixel dwell time; bidirectional scanning; 4-times averaging). AlexaFluor568 was excited with 561 nm (diode (SH) laser at 0.5-5.0% depending on the brightness of the immunostaining) and detected with MA-PMT detector at 576-700 nm. Hoechst was excited with 405 nm (diode laser at 0.2% except for Aldolase condition (0.5%)) and detected with MA-PMT detector at 410-493 nm. Laser excitation powers were kept constant across all samples per immunostaining condition.

Images immunostained for SOX9 and KI67 and acquired as described above were also used for quantifications of the total SOX9 expression level (Fig. 3C left) or the fraction of proliferative cells (Fig. 3E) in SOX9 spheroids *versus* WT organoids.

Image analysis of SOX9 immunostained 2D enteroid monolayer cultures to estimate total SOX9 expression levels

Hoechst-stained nuclei in confocal images of WT or Sox9_spheroid 2D EMCs immunostained for SOX9 (see "IF of 2D enteroid monolayer cultures and confocal imaging") were manually segmented using ImageJ (Schindelin et al., 2012). Following conversion of manual segmentation masks into Cellpose (Stringer et al., 2021)-compatible masks, mean SOX9 intensities were extracted using the Scikit-image library (Van Der Walt et al., 2014) and compared between WT and SOX9_spheroid conditions (Fig. 3C left). For the WT condition, cells were classified into SOX9-positive and SOX9-negative and analyzed separately to distinguish on-target from background fluorescence. Quantifications were based on 3 images with 456 cells (162 SOX9+, 294 SOX9-) for WT and 3 images with 159 cells for SOX9_spheroid from one IF experiment, whereby cells in each image were aggregated and the mean was calculated. Custom-written analysis code can be found on GitLab via the following link: https://gitlab.com/nikewalther/walther_sox9organoid_2025/-/tree/main/ConfocalAnalysis_Scripts/Sox9ExpressionLevelQuantification?ref_type=heads

Image analysis of HaloTag-stained live SOX9-Halo 2D enteroid monolayer cultures to estimate SOX9-Halo transgene expression levels

Nuclei in confocal images of WT or SOX9_spheroid live 2D EMCs (see "Confocal imaging of POI-Halo enteroid monolayer cultures") were manually segmented using ImageJ (Schindelin et al., 2012) based on the predominant nuclear localization of HTL-JFX549-labeled SOX9-Halo. Following conversion of manual segmentation masks into Cellpose (Stringer et al., 2021)-compatible masks, mean SOX9-Halo intensities were extracted using the Scikit-image library (Van Der Walt et al., 2014) and compared between SOX9_budding and SOX9_spheroid conditions (Fig. 3C right). Quantifications were based on 3 images with 864 cells for SOX9_budding and 3 images with 537 cells for SOX9_spheroid from one live-cell imaging experiment, whereby cells in each image were aggregated and the mean was calculated. Custom-written analysis code can be found on GitLab via the following link: https://gitlab.com/nikewalther/walther_sox9organoid_2025/-

/tree/main/ConfocalAnalysis_Scripts/Sox9ExpressionLevelQuantification?ref_type=heads

Image analysis of KI67-immunostained 2D enteroid monolayer cultures to quantify the fraction of proliferative cells

Hoechst-stained nuclei in confocal images of 2D EMCs immunostained for KI67 (see "IF of 2D enteroid monolayer cultures and confocal imaging") were manually segmented using ImageJ (Schindelin et al., 2012) and classified into KI67-positive and KI67-negative. Following conversion of manual segmentation masks into Cellpose (Stringer et al., 2021)-compatible masks, mean KI67 intensities were extracted using the Scikit-image library (Van Der Walt et al., 2014) and compared between WT and SOX9_spheroid conditions (Fig. 3E). Quantifications were based on 7 images with 752 cells for WT and 7 images with 648 cells for SOX9_spheroid from one IF experiment, whereby cells in each image were aggregated and the mean was calculated. Custom-written analysis code can be found on GitLab via the following link: https://gitlab.com/nikewalther/walther_sox9organoid_2025/-/tree/main/ConfocalAnalysis_Scripts/Ki67FrequencyDetermination?ref_type=heads

Image analysis of enteroid monolayer cultures to quantify cellular morphological features

To quantify the cellular morphological features nuclear area and nearest-nuclei-distance (Fig. 3D), WT and SOX9_spheroid confocal images of live or immunostained 2D EMCs were pooled, including the three experiments above (determination of total SOX9 expression level (IF), SOX9-Halo transgene expression level (live), and proliferation frequency (IF)). Based on the manually segmented and converted nuclear masks, the Scikit-image library (Van Der Walt et al., 2014) was used to compute the nuclear area and the mean distance from the centroid of a nucleus to the two nearest centroids of its neighboring nuclei (nearest-nuclei-distance). Quantifications were based on 23 images with 3029 cells for WT and 30 images with 3100 cells for SOX9_spheroid from a total of 4 experiments, whereby cells in each image were aggregated and the mean was calculated. Multinucleated cells were excluded from this analysis. Custom-written analysis code can be found on GitLab via the following link: https://gitlab.com/nikewalther/walther_sox9organoid_2025/-/tree/main/ConfocalAnalysis_Scripts/CellMorphologyQuantification?ref_type=heads

Classification and quantification of cell division errors in 2D enteroid monolayer cultures

To classify and quantify aberrant cell division phenotypes in WT, SOX9_budding, and SOX9_spheroid conditions, confocal images of live or immunostained 2D EMCs were used. The cell division phenotypes (a) two nuclei fused, (b) three nuclei fused, (c) micronuclei, and (d) multiple fragmented nuclei or micronuclei were classified according to the examples in Fig. S3B (left) and their occurrence per total number of cells per image was quantified. Quantifications were based on 13 images with 1821 cells for WT, 8 images with 1626 cells for SOX9_budding, and 17 images with 1756 cells for SOX9_spheroid from a total of 4 experiments, whereby cells in each image were aggregated and the mean was calculated. Not all conditions were included in each experiment.

Preparation of 2D enteroid monolayer cultures for SMT and PAPA-SMT

2D EMCs derived from stable SOX9-Halo mSIO lines were seeded and cultured as described above. 2-6 d post-seeding (depending on the degree of monolayer confluency and the formation of crypt- and villus-like morphological characteristics), monolayers were stained for SMT with 50 nM HTL-JFX549 (bulk labeling for nuclear segmentation and feature extraction; kind gift from Luke Lavis) and 1 nM HTL-JFX650 (sparse labeling for SMT; kind gift from Luke Lavis) in DMEM/F-12 without phenol red for 15 min. Following two washes with DMEM/F-12 without phenol red for 15 min each, the medium was replaced one more time to DMEM/F-12 without phenol red before proceeding with imaging.

Transient transduction of 2D enteroid monolayer cultures using crude rAAV vector preparations for SMT and PAPA-SMT

2D EMCs derived from WT mSIOs (for SMT of SOX9ΔHMG-Halo) or stable SOX9-Halo budding organoid or spheroid lines (for PAPA-SMT) were seeded and cultured as described above. 1-3 d post-seeding, the medium was replaced with a transduction mixture of 250 µL crude rAAV preparation and 250 µL plating medium. On the next day, the transduction mixture was removed. As best transgene expression was reached about 2 d post-transduction, IntestiCult w/o Pen/Strep and selection antibiotics was added for incubation for another day before staining for SMT with 50 nM HTL-JFX549 and 1 nM HTL-JFX650 in DMEM/F12 without phenol read for 15 min

at 37°C. For PAPA-SMT, staining with 50 nM HTL-JFX549 together with 50 nM STL-JFX650 (SOX9-SNAPf) or 5 nM STL-JFX650 (SNAPf-3xNLS) in IntestiCult w/o Pen/Strep and selection antibiotics was performed ON at 37°C before PAPA-SMT on the next day. Upon labeling for SMT or PAPA-SMT, two washes for 30 min each in DMEM/F12 without phenol red were performed prior to imaging in DMEM/F12 without phenol red.

Preparation of SOX9-Halo spheroids for SMT

For SMT of 3D spheroids, SOX9-Halo spheroids were seeded in 10-20µL droplets in 50% Matrigel in IntestiCult in 8-well Labteks II #1.5 and cultured as described before. Spheroids were grown until they were almost or even slightly touching the glass bottom of the culture dish, resulting in a restricted area within spheroids in which a layer of cells was oriented almost in parallel to the glass bottom. Such a configuration, required for successful HILO-based SMT, was typically reached 5 d post-seeding. Staining was performed with 50 nM HTL-JFX549 and 1-2.5 nM HTL-JFX650 in DMEM/F12 without phenol red for 1 h at 37°C. Following four washes in DMEM/F12 without phenol red for 30 min each and replacing the medium one more time with fresh DMEM/F12, SMT was performed.

TIRF microscope for HILO-based fast SMT and PAPA-SMT

All SMT and PAPA-SMT experiments were performed using a custom-built microscope as previously described (Hansen et al., 2017). In brief, a Nikon Ti microscope was equipped with a 100×/N.A. 1.49 oil-immersion TIRF objective (Nikon apochromat CFI Apo TIRF 100× Oil), a motorized mirror, a perfect focus system, an EM-CCD camera (Andor iXon Ultra 897), a laser launch with 405 nm (140 mW, OBIS, Coherent), 488 nm, 561 nm and 639 nm (1 W, Genesis Coherent) laser lines, and an incubation chamber maintaining a humidified atmosphere with 5% CO_2 at 37°C. The NIS-Elements software (Nikon) was used for controlling all microscope, camera and hardware components. Imaging was performed at laser power densities of approximately 52 W/cm^2 for 405 nm (violet), 91 W/cm^2 for 488 nm (blue), 100 W/cm^2 for 561 nm (green), and 2.3 kW/cm^2 for 639 nm (red).

Microscope automation for SMT and PAPA-SMT

A custom-built inverted Nikon Ti microscope described above was automated using code written in Python and the NIS Elements Macro Language as previously described (Dahal et al., 2025; Graham et al., 2025; Walther et al., 2024). In brief, the microscope stage rastered in a grid-like pattern to move the sample. At each grid position, a 512 px x 512 px (6710.9 μm^2) image was recorded in the densely labeled JFX549 channel or in the H2B-mEGFP channel for SOX9-Halo_spheroid. Segmentation of nuclei was performed using the pre-trained Versatile Fluorescent Nuclei model of the Python package StarDist (Schmidt et al., 2018). After randomly choosing one of the nuclei within a range of user-defined brightness and size parameters, the stage was moved to center it in the large FOV. The FOV was then resized to contain a smaller 150 px x 150 px (576 μm^2) square (small FOV; zoom-in) centered on the chosen nucleus. Images of the large and small FOVs were recorded in multiple channels corresponding to the fluorophores in the sample (GFP, JFX549, JFX650). JFX650 fluorophores in the small FOV were pre-bleached with red light at 639 nm to achieve sparse and thus trackable single molecules, following which an illumination sequence for fast SMT or PAPA-SMT was executed. On-the-fly localization density assessment was performed using quot (https://github.com/alecheckert/quot; (Heckert et al., 2022)). Microscope automation was based on our previously published code ((Walther et al., 2024); https://gitlab.com/tjian-darzacq-lab/walther_2dmsios_automatedsmt_2024/-/tree/main/Microscope_Automation_Scripts?ref_type=heads).

Fast SMT of POI-Halo in enteroid monolayer cultures

For imaging in GFP, JFX549 or JFX650 channels, laser powers were set to 100 mW for 488 nm, 110 mW for 561 nm, and to 1100 mW for 639 nm. The exposure time was set between 20 ms and 200 ms with a laser excitation power between 1.35% and 20% to achieve segmentable bulk labeling of POI-Halo depending on its expression level and to avoid saturation. Imaging conditions were kept constant across experiments for the same POI-Halo condition. Semrock 536/40 nm, 593/40 nm or 676/37 nm bandpass filters, respectively, were used.

For fast SMT experiments, the following bleaching durations in the 639 nm channel (100% laser excitation power) prior to executing an illumination sequence were used:

H2B – 15s, NLS – 10s, SOX9_budding – 15s or 10s, SOX9_spheroid – 15s, SOX9ΔHMG – 15s.

The triggered illumination sequence consisted of the following phases at a frame rate of 7.48 ms/frame:

1. Imaging: 5000 frames of red light (639 nm, one 2 ms stroboscopic pulse per frame) with 5% 405 nm reactivation (pulsed during the 0.48 ms camera transition time between 7 ms detection windows)

2. Imaging: 5000 frames of red light (639 nm, one 2 ms stroboscopic pulse per frame) with 10% 405 nm reactivation (pulsed during the 0.48 ms camera transition time between 7 ms detection windows)

To reduce the motion blur of moving molecules (Hansen et al., 2018), red illumination was restricted to stroboscopic pulses during each frame of a single-molecule movie.

Fast SMT of SOX9-Halo in SOX9-Halo spheroids

Fast SMT in 3D spheroids was performed in analogy to SMT in 2D EMCs with the exception that cells within spheroids that were close enough (10-20 μm) to the glass bottom of the cover slip to be reachable by HILO illumination were manually selected and centered within the FOV before triggering a sequence of epifluorescence imaging and SMT.

The following modified SMT illumination sequence was used:

1) As the bleaching efficiency in the 639 nm channel depended on the distance of the cell from the glass bottom of the culture dish, its duration had to be adjusted manually for each cell and was carried out until a trackable density of single-molecule localizations was achieved.

2) Embedding in Matrigel and the mostly larger distance of selected cells in 3D spheroids from the glass bottom of the culture dish in comparison to 2D EMCs required a higher 405 nm reactivation. The triggered illumination sequence thus consisted of the following phases at a frame rate of 7.48 ms/frame:

1. Imaging: 5000 frames of red light (639 nm, one 2 ms stroboscopic pulse per frame) with 0% 405 nm reactivation (primarily used to estimate background reactivation and non-specific fluorescence to judge suitability of selected cell for SMT)

2. Imaging: 5000 frames of red light (639 nm, one 2 ms stroboscopic pulse per frame) with 20% 405 nm reactivation (pulsed during the 0.48 ms camera transition time between 7 ms detection windows)

3. Imaging: 5000 frames of red light (639 nm, one 2 ms stroboscopic pulse per frame) with 100% 405 nm reactivation (pulsed during the 0.48 ms camera transition time between 7 ms detection windows)

PAPA-SMT of POI-Halo in enteroid monolayer cultures

Imaging for PAPA-SMT was performed as described for fast SMT.

For all PAPA-SMT conditions, an initial bleaching in the 639 nm channel (100% laser excitation power) was performed for 15 s prior to executing an illumination sequence.

For PAPA-SMT experiments, the illumination sequence consisted of 5 cycles of the following phases at a frame rate of 7.48 ms/frame:

1. Imaging: 250 frames of red light (639 nm), one 2 ms stroboscopic pulse per frame, recorded

2. DR pulse: 10 frames of violet light (405 nm), continuously during 7 ms detection window, recorded

3. DR imaging: 250 frames of red light, one 2 ms stroboscopic pulse per frame, recorded

4. Bleaching: 200 frames of red light, continuously during 7 ms detection window, not recorded

5. Imaging: 250 frames of red light, one 2 ms stroboscopic pulse per frame, recorded

6. PAPA pulse: 100 frames of green light (561 nm), continuously during 7 ms detection window, recorded

7. PAPA imaging: 250 frames of red light, one 2 ms stroboscopic pulse per frame, recorded

8. Bleaching: 200 frames of red light (639 nm), continuously during 7 ms detection window, not recorded

SMT and PAPA-SMT data processing and analysis

Image analysis for SMT and PAPA-SMT

Image analysis for SMT and PAPA-SMT was performed as previously described (Walther et al., 2024). In brief, nuclei in epifluorescence images corresponding to SMT or PAPA-SMT movies were segmented using StarDist (Schmidt et al., 2018). Segmented nuclei were subjected to manual QC for downstream analysis using CellPicker ((Walther et al., 2024); https://github.com/tgwgraham/basic_PAPASMT_analysis). Hereby, nuclei were filtered based on their intensities in each channel and a QC was performed to exclude nuclei with erroneous segmentation masks, unusual textures, or masks corresponding to segmented autofluorescent debris. In addition, CellPicker enabled the manual classification of cells based on the expression of the additionally recorded LGR5-DTR-GFP marker for stem/early progenitor cells. For each category, the cell morphological parameter nuclear area as well as the mean POI intensity in different channels as a proxy for relative POI expression level were computed for each of the chosen nuclei using the Python library Scikit-image (Van Der Walt et al., 2014) and the nuclear masks generated by StarDist. For nuclei in FOVs with three or more nuclei, the distance from the centroid of the nucleus of interest to the centroid of every other nucleus in the FOV was computed and the mean distance of the centroid of the nucleus of interest to its two nearest neighbors was recorded (nearest-nuclei-distance).

SMT data processing and analysis

SMT movies were processed using quot (https://github.com/alecheckert/quot; (Heckert et al., 2022)), which identifies single-molecule localizations and generates trajectories, as previously described (Walther et al., 2024). The following settings were used: [filter] start = 0; method = 'identity'; chunk_size = 100; [detect] method = 'llr'; k=1.0; w=9, t=18; [localize] method = 'ls_int_gaussian', window size = 9; sigma = 1.0; ridge = 0.001; max_iter = 10; damp = 0.3; camera_gain = 109.0; camera_bg = 470.0; [track] method = 'conservative'; pixel_size_µm = 0.160; frame interval = 0.00748; search radius = 1; max_blinks = 0; min_IO = 0; scale = 7.0. To construct trajectories, a conservative method was used, in which only trajectories with unambiguously assigned localizations were considered. Tracking statistics are summarized in Fig. S9. Trajectories were assigned to cells using the StarDist-generated nuclear masks.

Diffusion coefficient distributions were obtained using the state array method of saSPT (https://github.com/alecheckert/saspt; (Heckert et al., 2022)). Here, a regular Brownian motion with a normally distributed, mean-zero localization error (RBME) model was fit to populations of single-molecule trajectories in each cell to obtain a state array, which consists of posterior occupations of states defined by their localization errors and diffusion coefficients. To obtain diffusion spectra, these state array distributions were marginalized on the diffusion coefficients. Mean diffusion spectra for each POI were constructed by averaging over single-cell diffusion spectra, whereby cells were weighted by the number of trajectories. The fraction of bound trajectories for a POI/condition was defined as the fraction of trajectories with diffusion coefficients below 0.15 $\mu m^2/s$ (fraction bound), as molecules diffusing at such slow rates are indistinguishable from H2B-Halo (Walther et al., 2024). As diffusion coefficient thresholds of both 0.1 and 0.15 $\mu m^2/s$ for the upper limit of trajectories accounted for in the bound fraction were used in the literature, we analyzed all fast SMT POI-Halo conditions acquired in this study with various thresholds ranging from 0.05 to 1 $\mu m^2/s$. This revealed no change in the rank order of conditions based on their fractions bound independent of the diffusion threshold used except for SOX9_budding_2D and SOX9_budding_3D with expected similar diffusion behavior (Fig. S10A). However, we note that – at least for large fraction bound threshold deviations from 0.15 $\mu m^2/s$ – this is partially due to all POIs measured in this study being characterized by distinct immobile and free diffusion peaks. Indeed, changes in the diffusive behavior between LGR5+/- SOX9_budding and SOX9_spheroid conditions occurred between these immobile and freely diffusing fractions (e.g. between bound and unbound states), whereas a slowly diffusing SOX9-Halo pool with intermediate mobility remained unchanged between LGR5+ and LGR5- conditions (Fig. S10B). Fractions of bound trajectories were also computed for individual nuclei based on the single-cell diffusion spectra to correlate them with extracted morphological features, such as nuclear area and nearest-nuclei-distance, as well as the POI expression level. To perform these processing and analysis steps, we used our previously published (Walther et al., 2024) custom-written Jupyter notebook for an all-in-one cell level-based diffusion analysis and correlation with extracted cellular features (https://gitlab.com/tjian-darzacq-lab/walther_2dmsios_automatedsmt_2024/-/tree/main/SMT_analysis_scripts?ref_type=heads).

For fast SMT data in SOX9-Halo spheroids, a filter was set to include only cells with at least 100 non-singlet trajectories for downstream analysis.

Cluster analysis based on single-cell diffusion spectra

Cluster-based analyses of single-cell diffusion spectra derived from fast SMT data were performed as previously described (Walther et al., 2024): The Jensen-Shannon distance as the distance metric (Nielsen, 2019) was used to compute a matrix of pairwise distances between pairs of single-cell diffusion spectra. Cells with complete linkage were then hierarchically clustered using the AgglomerativeClustering class of the Scikit-learn Python library (Pedregosa et al., 2011). The over- and under-representation of cells from different conditions in different clusters was quantified with a *p*-value computed from the hypergeometric distribution. To account for multiple comparisons, the significance threshold was Bonferroni-corrected. The option for diffusion-based cluster analysis is included in our previously published (Walther et al., 2024) custom-written Jupyter notebook (https://gitlab.com/tjian-darzacq-lab/walther_2dmsios_automatedsmt_2024/-/tree/main/SMT_analysis_scripts?ref_type=heads).

PAPA data processing and analysis

PAPA movies were processed and analyzed as described for SMT with the following specifications: All trajectory segments occurring within the first 30 frames after pulses of 561 nm light (PAPA trajectories) or 405 nm light (DR trajectories) were extracted using custom MATLAB code reported previously ((Graham et al., 2022; Walther et al., 2024); https://github.com/tgwgraham/basic_PAPASMT_analysis). PAPA and DR trajectories were then separately analyzed similar to SMT data. To distinguish undifferentiated from differentiated cells in SOX9_budding PAPA experiments, the whole cell population was split into small (proxy for stem and early progenitor cells) and large nuclei (proxy for late progenitor and differentiated cells) using a nuclear area of 75 μm^2 as threshold based on our previously established correlation between differentiation state and nuclear size (Walther et al., 2024). A custom-written Jupyter notebook is available here: https://gitlab.com/nikewalther/walther_sox9organoid_2025/-/tree/main/PAPA_analysis_scripts.

Bulk RNAseq

Organoid collection for RNAseq

For bulk RNAseq experiments in biological triplicates, mSIOs were used at the passages indicated in Fig. 4A plus/minus two passages. Organoids were seeded into 6 wells of a 24-well plate per line and cultured as described before. 5 d post-seeding, the medium was removed and organoids from the 6 wells were harvested with 1 mL TRIzol (Thermo Fisher Scientific, cat.# 15596026) into low-binding 1.7 mL tubes (Sorenson, cat.# 39640T) by rigorously pipetting up and down with a 1 mL pipette to dislodge and homogenize the organoid-containing Matrigel domes. Organoid-TRIzol samples were directly stored at -20°C until RNA extraction.

RNA extraction and poly-A RNAseq library preparation

Poly-A RNAseq was performed in three biological replicates per condition. Total RNA was extracted with TRIzol according to the manufacturer's instructions by performing the optional centrifugation step of the lysates (5 min at 12,000 g at 4–10°C) and an additional wash with one volume of chloroform after the recommended phenol:chloroform extraction (UltraPure Phenol:Chloroform:Isoamyl Alcohol, 25:24:1, v/v, cat.# 15593-01). RNA was quantified by spectrophotometer (NanoDrop, ThermoFisher Scientific) and checked for integrity by capillary electrophoresis (Fragment Analyzer, Agilent). 100-500 ng of total RNA were subjected to poly-A purification and library preparation with the NEBNext Poly(A) mRNA Magnetic Isolation Module (NEB, cat.# E7490S) in combination with the NEBNext Ultra II RNA Library Prep Kit for Illumina (NEB, cat.# E7770S). The NEBNext Adaptor for Illumina was diluted 1:5 (for 500 ng input RNA) or 1:25 (for 100 ng input RNA) in Tris/NaCl, pH 8.0 (10 mM Tris-HCl pH 8.0, 10 mM NaCl) and the ligation step was extended to 30 min. Libraries were enriched with 9-11 PCR cycles with the NEBNext Multiplex Oligos for Illumina (Dual Index Primers Set 1; NEB, cat.# E7600S). Library concentration was assessed by Qubit quantification (Qubit dsDNA HS Assay Kit; Invitrogen, cat.# Q32851). Multiplexed libraries were pooled and sequenced on the Illumina NovaSeq X Plus platform (150 bp, paired end reads) by MedGenome Inc. (Foster City, CA, USA).

RNAseq analysis

RNAseq raw reads were quality checked with FastQC (http://www.bioinformatics.babraham.ac.uk/projects/fastqc), trimmed with cutadapt (DOI: https://doi.org/10.14806/ej.17.1.200; version 4.5 with Python 3.10.13) and aligned onto the mouse genome (mm39) using STAR RNA-Seq aligner (Dobin et al., 2013) with the following options: --outSJfilterReads: Unique, --outFilterMultimapNmax: 1, --outFilterIntronMotifs: RemoveNoncanonical, --outSAMstrandField: intronMotif. Samtools (Li et al., 2009) (version 1.9) was used to convert STAR output .sam files into .bam files, and to sort and index them. After counting how many reads overlapped an annotated gene (Ensembl GRCm39 annotations) using HTSeq (Anders et al., 2015) (options: --htseq-count; --stranded=no -f bam; --additional-attr=gene_name -m union), the output counts files were used to find DEGs with DESeq2 (Love et al., 2014), run with default parameters within the Galaxy platform (Blankenberg et al., 2010; Giardine et al., 2005; Goecks et al., 2010). DEGs were called using an adjusted *p*-value ≤0.01, a fold change ≥2 and ≥10 mean counts. Gene transcript levels were visualized on the mm39 genome with the Integrative Genomics Viewer (IGV) (Robinson et al., 2011; Thorvaldsdóttir et al., 2013) using the bigWig output files from deepTools' bamCoverage (Ramírez et al., 2014) (version: 3.5.1; options: --binSize: 50; --extendReads: 250; --normalizeUsing: BPM; --samFlagInclude: 64). PCA and sample-to-sample distance analysis were part of the DESeq2 output.

Based on the DESeq2 output, pairwise comparison of conditions was performed by visualizing DEGs as called above via the EnhancedVolcano package (https://github.com/kevinblighe/EnhancedVolcano) within Bioconductor (Huber et al., 2015) (release 3.20) using R (version 4.4.2) and indicating the number of up- or downregulated genes. In addition, DEGs of pairwise comparisons were further analyzed with respect to their enrichment in biological pathways by GO analysis using the clusterProfiler (Yu, 2024) and AnnotationDbi (https://bioconductor.org/packages/AnnotationDbi) packages and selecting the ontology class BP for Biological processes.

Using the ComplexHeatmap package (Gu, 2022; Gu et al., 2016), a comparison of DEGs in all five conditions was visualized in a heatmap displaying *z*-scores, whereby either all DEGs (Fig. 4C) or selected DEGs (Fig. 4D) were plotted. In addition, CPMs

of some of these selected DEGs were plotted across all five conditions (Fig. S5; barplot: mean of triplicates; CPM values of individual replicates as points).

Quantification and statistical analysis

The significance of the differences in the distribution of SOX9 expression levels (Fig. 3C), the distributions in cellular morphological features (Fig. 3D), as well as the percentage of proliferating KI67-positive cells (Fig. 3E) and aberrant cell division phenotypes (Fig. S3B) was computed using a Mann-Whitney U test after aggregating all cells in one image and calculating a mean for each image. For SOX9 expression level analyses (Fig. 3B), alternative hypotheses in which cells in the SOX9 spheroid condition had stochastically greater metric values were used. For cellular morphologies (Fig. 3D), proliferation (Fig. 3E), and cell division phenotypes (Fig. S3B), a two-sided alternative hypothesis was used (Fig. 4D). *p*-values indicated in Fig. 3C-E and Fig. S3B were rated as follows: (ns) non-significant, $p>0.05$; (*) $p\leq0.05$; (**) $p\leq0.01$; (***) $p\leq0.001$; (****) $p\leq0.0001$. The exact *p*-values for pairwise comparisons in Fig. 3B-E are as follows: Fig. 3C left: $p=0.05$; Fig. 3C right: $p=0.35$; Fig. 3D left: $p=7.2e-5$; Fig. 3D right: $p=7.2e-5$; Fig. 3E: $p=0.01$. The exact *p*-values for WT *vs.* SOX9_budding, WT *vs.* SOX9_spheroid, and SOX9_budding *vs.* SOX9_spheroid in Fig. S3B are as follows: Fig. S3B top left (a): 1.00, 1.95e-3, 3.07e-3; Fig. S3B top right (b): 5.55e-1, 4.91e-2, 2.06e-2; Fig. S3B bottom left (c): 7.84e-2, 1.01e-5, 6.70e-3; Fig. S3B bottom right (d): 7.57e-2, 1.11e-2, 2.35e-1.

For SMT experiments, bootstrapping on all combined experiments per fast SMT condition was performed by drawing 1000 samples from the population with replacement, whereby each sample contained as many nuclei as the total number of nuclei in the population under analysis. For each bootstrap replicate, diffusion spectra marginalized on the diffusion coefficients were generated and used to compute CIs for the fraction of bound trajectories. Code for SMT bootstrap analysis is included in our previously published (Walther et al., 2024) custom-written Jupyter notebook (https://gitlab.com/tjian-darzacq-lab/walther_2dmsios_automatedsmt_2024/-/tree/main/SMT_analysis_scripts?ref_type=heads).

For determining the significance of the difference in the fraction bound distributions between two conditions based on the single-cell fractions bound, Mann-Whitney U

tests were performed. The Benjamini-Hochberg procedure was used to correct for multiple comparisons. *p*-values were rated as follows: (ns) non-significant, $p>0.05$; (*) $p\leq0.05$; (**) $p\leq0.01$; (***) $p\leq0.001$; (****) $p\leq0.0001$. Statistics of fraction bound comparisons between conditions are summarized in Fig. S8C.

For determining a potential correlation between the relative POI expression level and the fraction bound in SMT experiments (Fig. 2D, Fig. 5D; Fig. S1G,H), nuclear SOX9 intensities were extracted from StarDist (Schmidt et al., 2018) masks and plotted against the fraction bound of the POI in each nucleus inferred using saSPT's (Heckert et al., 2022) State Array Dataset class for each POI. For each POI, a potential correlation between the fraction bound and the relative SOX9 expression level was calculated by performing a linear least-squares regression using the SciPy Python library (Virtanen et al., 2020), which yielded an *R*-value (Pearson correlation coefficient). *p*-values were computed using a Wald test with a t-distribution test statistic and a two-sided alternative hypothesis. Code is included in our previously published (Walther et al., 2024) custom-written Jupyter notebook (https://gitlab.com/tjian-darzacq-lab/walther_2dmsios_automatedsmt_2024/-/tree/main/SMT_analysis_scripts?ref_type=heads).

The corresponding figure legends contain statistical details for SMT experiments and fraction bound/intensity correlations mentioned above.

For PAPA-SMT experiments, statistical analyses were performed as described before using custom-written MATLAB scripts ((Dahal et al., 2025; Walther et al., 2024); https://github.com/tgwgraham/basic_PAPASMT_analysis). For a side-by-side comparison of the distributions for DR and PAPA trajectories, they were randomly subsampled without replacement for the condition with more trajectories. Following subsampling, bootstrapping analysis with replacement was performed on all combined experiments per condition for the PAPA datasets for SOX9_spheroid/SOX9, SOX9_spheroid/NLS, SOX9_budding/SOX9, and SOX9_budding/NLS. For each combined dataset, a random sample of size *n*, where *n* is the total number of cells in the combined dataset, was drawn 100 times. We reported the mean and standard deviation from these analyses (Fig. 6G,H; Fig. S7). For significance testing between DR and PAPA conditions, two-tailed *p*-values were calculated based on a normal distribution (SciPy function (Virtanen et al., 2020), scipy.stats.norm.sf) with mean

equal to the difference between sample means and variance equal to the sum of the variances from the bootstrap resampling. The statistical details for the PAPA experiments shown in Fig. 6E-H are as follows: SOX9→SOX9: Subsampling of 9255 trajectories determined fractions bound to 40.3% (DR) and 52.0% (PAPA). Bootstrap resampling with 100 replicates determined fractions bound to 40.6±2.9% (DR) and 51.9±3.1% (PAPA); 2-sided *p*-value: 2.3e-7. SOX9→NLS: Subsampling of 4117 trajectories determined fractions bound to 26.4% (DR) and 26.1% (PAPA). Bootstrap resampling with 100 replicates determined fractions bound to 27.2±2.5% (DR) and 26.1±2.8% (PAPA); 2-sided *p*-value: 1.4. The statistical details for the PAPA experiments shown in Fig. S7A,B are as follows: SOX9→SOX9: Subsampling of 1191 trajectories determined fractions bound to 38.2% (DR) and 46.2% (PAPA). Bootstrap resampling with 100 replicates determined fractions bound to 38.9±4.9% (DR) and 46.0±6.6% (PAPA); 2-sided *p*-value: 0.09. SOX9→NLS: Subsampling of 2644 trajectories determined fractions bound to 16.8% (DR) and 16.3% (PAPA). Bootstrap resampling with 100 replicates determined fractions bound to 17.6±2.5% (DR) and 16.4±2.8% (PAPA); 2-sided *p*-value: 1.44. For Fig. S7E-H, bootstrapped fractions bound are as follows: (E) – 40.5±5.9% (DR) and 48.6±7.9% (PAPA); (F) – 21.3±5.1% (DR) and 17.6±5.2% (PAPA); (G) – 35.0±7.0% (DR) and 42.3±9.1% (PAPA); (H) – 14.8±3.2% (DR) and 15.0±2.8% (PAPA). The corresponding 2-sided *p*-values are as follows: (E) 0.111; (F) 1.679; (G) 0.215; (H) 0.923. *p*-values indicated in Fig. 6G,H and Fig. S7 were rated as follows: (ns) non-significant, $p>0.05$; (*) $p\leq0.05$; (**) $p\leq0.01$; (***) $p\leq0.001$; (****) $p\leq0.0001$.

Supplemental references

Anders, S., Pyl, P.T., and Huber, W. (2015). HTSeq-A Python framework to work with high-throughput sequencing data. Bioinformatics *31*, 166–169. https://doi.org/10.1093/bioinformatics/btu638.

Benyamini, B., Esbin, M.N., Whitney, O., Walther, N., and Maurer, A.C. (2023). Transgene Expression in Cultured Cells Using Unpurified Recombinant Adeno-Associated Viral Vectors. J. Vis. Exp. *2023*, 1–22. https://doi.org/10.3791/65572.

Blankenberg, D., Kuster, G. Von, Coraor, N., Ananda, G., Lazarus, R., Mangan, M., Nekrutenko, A., and Taylor, J. (2010). Galaxy: A web-based genome analysis tool for experimentalists. Curr. Protoc. Mol. Biol. 1–21. https://doi.org/10.1002/0471142727.mb1910s89.

Dahal, L., Graham, T.G.W., Dailey, G.M., Heckert, A., Tjian, R., and Darzacq, X. (2025). Surprising features of nuclear receptor interaction networks revealed by live-cell single-molecule imaging. Elife *12*, RP92979. https://doi.org/10.7554/eLife.92979.

Dobin, A., Davis, C.A., Schlesinger, F., Drenkow, J., Zaleski, C., Jha, S., Batut, P., Chaisson, M., and Gingeras, T.R. (2013). STAR: Ultrafast universal RNA-seq aligner. Bioinformatics *29*, 15–21. https://doi.org/10.1093/bioinformatics/bts635.

Giardine, B., Riemer, C., Hardison, R.C., Burhans, R., Elnitski, L., Shah, P., Zhang, Y., Blankenberg, D., Albert, I., Taylor, J., et al. (2005). Galaxy: A platform for interactive large-scale genome analysis. Genome Res. *15*, 1451–1455. https://doi.org/10.1101/gr.4086505.

Goecks, J., Nekrutenko, A., Taylor, J., Afgan, E., Ananda, G., Baker, D., Blankenberg, D., Chakrabarty, R., Coraor, N., Von Kuster, G., et al. (2010). Galaxy: a comprehensive approach for supporting accessible, reproducible, and transparent computational research in the life sciences. Genome Biol. *11*. https://doi.org/10.1186/gb-2010-11-8-r86.

Götzke, H., Kilisch, M., Martínez-Carranza, M., Sograte-Idrissi, S., Rajavel, A., Schlichthaerle, T., Engels, N., Jungmann, R., Stenmark, P., Opazo, F., et al. (2019). The ALFA-tag is a highly versatile tool for nanobody-based bioscience applications. Nat. Commun. *10*, 1–12. https://doi.org/10.1038/s41467-019-12301-7.

Graham, T.G.W., Ferrie, J.J., Dailey, G.M., Tjian, R., and Darzacq, X. (2022). Detecting molecular interactions in live-cell single-molecule imaging with proximity-assisted photoactivation (PAPA). Elife *11*, 1–46. https://doi.org/10.7554/eLife.76870.

Graham, T.G.W., Dugast-Darzacq, C., Dailey, G.M., Weng, B., Anantakrishnan, S., Darzacq, X., and Tjian, R. (2025). Single-molecule live imaging of subunit interactions and exchange within cellular regulatory complexes. Mol. Cell *85*, 2854-2868.e7. https://doi.org/https://doi.org/10.1016/j.molcel.2025.06.028.

Gu, Z. (2022). Complex heatmap visualization. IMeta *1*, 1–15. https://doi.org/10.1002/imt2.43.

Gu, Z., Eils, R., and Schlesner, M. (2016). Complex heatmaps reveal patterns and correlations in multidimensional genomic data. Bioinformatics *32*, 2847–2849. https://doi.org/10.1093/bioinformatics/btw313.

Hansen, A.S., Pustova, I., Cattoglio, C., Tjian, R., and Darzacq, X. (2017). CTCF and cohesin regulate chromatin loop stability with distinct dynamics. Elife *6*, 1–33. https://doi.org/10.7554/eLife.25776.

Hansen, A.S., Woringer, M., Grimm, J.B., Lavis, L.D., Tjian, R., and Darzacq, X. (2018). Robust model-based analysis of single-particle tracking experiments with Spot-On. Elife *7*, e33125. https://doi.org/10.7554/eLife.33125.

Heckert, A., Dahal, L., Tijan, R., and Darzacq, X. (2022). Recovering mixtures of fast-diffusing states from short single-particle trajectories. Elife *11*, 1–32. https://doi.org/10.7554/ELIFE.70169.

Huber, W., Carey, V.J., Gentleman, R., Anders, S., Carlson, M., Carvalho, B.S., Bravo, H.C., Davis, S., Gatto, L., Girke, T., et al. (2015). Orchestrating high-throughput genomic analysis with Bioconductor. Nat. Methods *12*, 115–121. https://doi.org/10.1038/nmeth.3252.

Li, H., Handsaker, B., Wysoker, A., Fennell, T., Ruan, J., Homer, N., Marth, G., Abecasis, G., and Durbin, R. (2009). The Sequence Alignment/Map format and SAMtools. Bioinformatics *25*, 2078–2079. https://doi.org/10.1093/bioinformatics/btp352.

Love, M.I., Huber, W., and Anders, S. (2014). Moderated estimation of fold change and dispersion for RNA-seq data with DESeq2. Genome Biol. *15*, 1–21. https://doi.org/10.1186/s13059-014-0550-8.

Murphy, G.J., Mostoslavsky, G., Kotton, D.N., and Mulligan, R.C. (2006). Exogenous control of mammalian gene expression via modulation of translational termination. Nat. Med. *12*, 1093–1099. https://doi.org/10.1038/nm1376.

Nielsen, F. (2019). On the Jensen-Shannon symmetrization of distances relying on abstract means. Entropy *21*, 1–23. https://doi.org/10.3390/e21050485.

Pedregosa, F., Varoquaux, G., Gramfort, A., Michel, V., Thirion, B., Grisel, O., Blondel, M., Prettenhofer, P., Weiss, R., Dubourg, V., et al. (2011). Scikit-learn: Machine Learning in Python. J. Mach. Learn. Res. *12*, 2825–2830. https://doi.org/10.1289/EHP4713.

Pekrun, K., De Alencastro, G., Luo, Q.J., Liu, J., Kim, Y., Nygaard, S., Galivo, F., Zhang, F., Song, R., Tiffany, M.R., et al. (2019). Using a barcoded AAV capsid library to select for clinically relevant gene therapy vectors. JCI Insight *4*. https://doi.org/10.1172/jci.insight.131610.

Ramírez, F., Dündar, F., Diehl, S., Grüning, B.A., and Manke, T. (2014). DeepTools: A flexible platform for exploring deep-sequencing data. Nucleic Acids Res. *42*, 187–191. https://doi.org/10.1093/nar/gku365.

Robinson, J.T., Thorvaldsdóttir, H., Winckler, W., Guttman, M., Lander, E.S., Getz, G., and Mesirov, J.P. (2011). Integrative genomics viewer. Nat. Biotechnol. *29*, 24–26. https://doi.org/10.1038/nbt.1754.

Sanman, L.E., Chen, I.W., Bieber, J.M., Thorne, C.A., Wu, L.F., and Altschuler, S.J. (2020). Chapter 6. Methods Mol. Biol. *2171*, 99–113. .

Schindelin, J., Arganda-Carreras, I., Frise, E., Kaynig, V., Longair, M., Pietzsch, T., Preibisch, S., Rueden, C., Saalfeld, S., Schmid, B., et al. (2012). Fiji: An open-source

platform for biological-image analysis. Nat. Methods *9*, 676–682. https://doi.org/10.1038/nmeth.2019.

Schmidt, U., Weigert, M., Broaddus, C., and Myers, G. (2018). Cell detection with star-convex polygons (Springer International Publishing).

Stringer, C., Wang, T., Michaelos, M., and Pachitariu, M. (2021). Cellpose: a generalist algorithm for cellular segmentation. Nat. Methods *18*, 100–106. https://doi.org/10.1038/s41592-020-01018-x.

Thorvaldsdóttir, H., Robinson, J.T., and Mesirov, J.P. (2013). Integrative Genomics Viewer (IGV): High-performance genomics data visualization and exploration. Brief. Bioinform. *14*, 178–192. https://doi.org/10.1093/bib/bbs017.

Virtanen, P., Gommers, R., Oliphant, T.E., Haberland, M., Reddy, T., Cournapeau, D., Burovski, E., Peterson, P., Weckesser, W., Bright, J., et al. (2020). SciPy 1.0: fundamental algorithms for scientific computing in Python. Nat. Methods *17*, 261–272. https://doi.org/10.1038/s41592-019-0686-2.

Van Der Walt, S., Schönberger, J.L., Nunez-Iglesias, J., Boulogne, F., Warner, J.D., Yager, N., Gouillart, E., and Yu, T. (2014). Scikit-image: Image processing in python. PeerJ *2014*, 1–18. https://doi.org/10.7717/peerj.453.

Walther, N., Anantakrishnan, S., Graham, T.G.W., Dailey, G.M., and Tjian, R. (2024). Automated live-cell single-molecule tracking in enteroid monolayers reveals transcription factor dynamics probing lineage-determining function. Cell Rep. *43*, 114914. https://doi.org/10.1016/j.celrep.2024.114914.

Yu, G. (2024). Thirteen years of clusterProfiler. Innovation *5*, 5–6. https://doi.org/10.1016/j.xinn.2024.100722.
