# Supplementary material for: Distinct SOX9 single-molecule dynamics characterize adult differentiation and fetal-like reprogrammed states in intestinal organoids
Source: Stem Cell Reports. 2026 Jan 22;21(2):102787. doi: 10.1016/j.stemcr.2025.102787 (PMC12903095; doi:10.1016/j.stemcr.2025.102787)
Supplement: Document S2. Article plus supplemental information [file mmc9.pdf]

STEM CELL REPORTS
Article
OPEN ACCESS

Distinct SOX9 single-molecule dynamics characterize adult differentiation and fetal-like reprogrammed states in intestinal organoids

Nike Walther,[1,2,5,6,9,*] Sathvik Anantakrishnan,[1,3] Gina M. Dailey,[1] Anna C. Maurer,[1,7,8] and Claudia Cattoglio[1,4]
[1]Department of Molecular and Cell Biology, Li Ka Shing Center for Biomedical and Health Sciences, California Institute for Regenerative Medicine (CIRM) Center of Excellence, University of California, Berkeley, Berkeley, CA 94720, USA
[2]Department of Genetics, University of Bayreuth, 95440 Bayreuth, Germany
[3]Biophysics Graduate Group, University of California, Berkeley, Berkeley, CA 94720, USA
[4]Howard Hughes Medical Institute, Berkeley, CA 94720, USA
[5]Present address: Institut Curie, PSL Université, Sorbonne Université, CNRS UMR168, Physics of Cells and Cancer Unit, 75005 Paris, France
[6]Present address: Department of Developmental and Stem Cell Biology, CNRS UMR3738 Paris Cité, Institut Pasteur, 75015 Paris, France
[7]Present address: Department of Biophysics, University of Michigan, Ann Arbor, Michigan 48109, USA
[8]Present address: Department of Microbiology and Immunology, University of Michigan Medical School, Ann Arbor, Michigan 48109, USA
[9]Lead contact
*Correspondence: nikewalther.science@gmail.com
https://doi.org/10.1016/j.stemcr.2025.102787

SUMMARY

Transcription factors (TFs) mediate gene expression changes during differentiation and development. However, how TF biophysical properties and abundance dynamically regulate specific cell state transitions remains poorly understood. Using automated live-cell single-molecule tracking (SMT) in intestinal organoid models, we revealed an expression-level-independent decrease in the fraction of immobile sex-determining region Y box 9 (SOX9) molecules during differentiation from ~48% to ~38%, largely dependent on DNA binding. Strikingly, long-term SOX9 overexpression caused organoids to transition from budding to spheroid morphology accompanied by increased proliferation and a loss in gene expression signatures for intestinal identity and function. In this fetal-like reprogrammed state, a larger fraction of partially self-interacting SOX9 molecules (~61%) binds to DNA. Our results suggest context-dependent SOX9 single-molecule dynamics during adult intestinal differentiation and fetal-like reversion in consequence to long-term SOX9 overexpression. Our work underpins the power of our automated live-cell SMT framework to generate testable hypotheses toward unraveling molecular mechanisms underlying tissue-level phenotypes.

INTRODUCTION

Gene expression programs are rewired during tissue development and homeostasis to ensure the formation and maintenance of healthy organs. This is accomplished by the differentiation of stem cells into various specialized cell types in a highly spatiotemporally controlled manner. One such regulatory layer is provided by lineage-specific transcription factors (TFs) (Weidemüller et al., 2021). Acting downstream of signaling pathways, TFs bind with co-factors to *cis*-regulatory elements of target genes, inducing or repressing their expression (Cramer, 2019). Misexpression of TFs conferring cell fate decisions can lead to cellular reprogramming and is associated with disease, including cancer (Huilgol et al., 2019; Lee and Young, 2013). It is thus crucial that lineage TFs are expressed in the correct tissue place at the correct dose. However, it remains poorly understood how the abundance and biophysical properties of cell fate-determining TFs change during stem cell differentiation to mature cell types. It is further unclear what TF dosage is tolerated for faithful differentiation and tissue formation, and whether TF overexpression alters their molecular dynamics. To address these questions, TF abundance and single-molecule dynamics need to be probed in differentiating multicellular systems, necessitating the choice of a model system that recapitulates *in vivo* differentiation trajectories and is amenable to live-cell single-molecule imaging across scales.

The mammalian intestine as the fastest renewing organ, which consists of crypts containing intestinal stem cells (ISCs), early progenitor cells, and niche-providing Paneth cells and villi composed of mature cell types, provides such a system: a spatial differentiation hierarchy guides the directional movement of ISCs during differentiation and maturation (Beumer and Clevers, 2016; Gehart and Clevers, 2019) (Figure 1A, left). This feature is recapitulated in *in vitro* tissue models of the gut: in budding structures of 3D mouse small intestinal organoids (mSIOs; enteroids), ISC-containing domains are located at the tips, and differentiating ISCs move inward (Date and Sato, 2015; Sato et al., 2009) (Figure 1A, middle). In 2D enteroid monolayer cultures (EMCs) (Altay et al., 2019; Sanman et al., 2020; Thorne et al., 2018), ISCs in proliferative centers, also containing differentiated Paneth cells, move outward along a radial differentiation trajectory (Thorne et al., 2018) (Figure 1A, right). Such 3D/2D organoid models retain all intestinal epithelial cell types and are amenable to live imaging, which enabled the study of cellular behavior within

Published by Elsevier Inc. on behalf of International Society for Stem Cell Research.
This is an open access article under the CC BY license (http://creativecommons.org/licenses/by/4.0/).

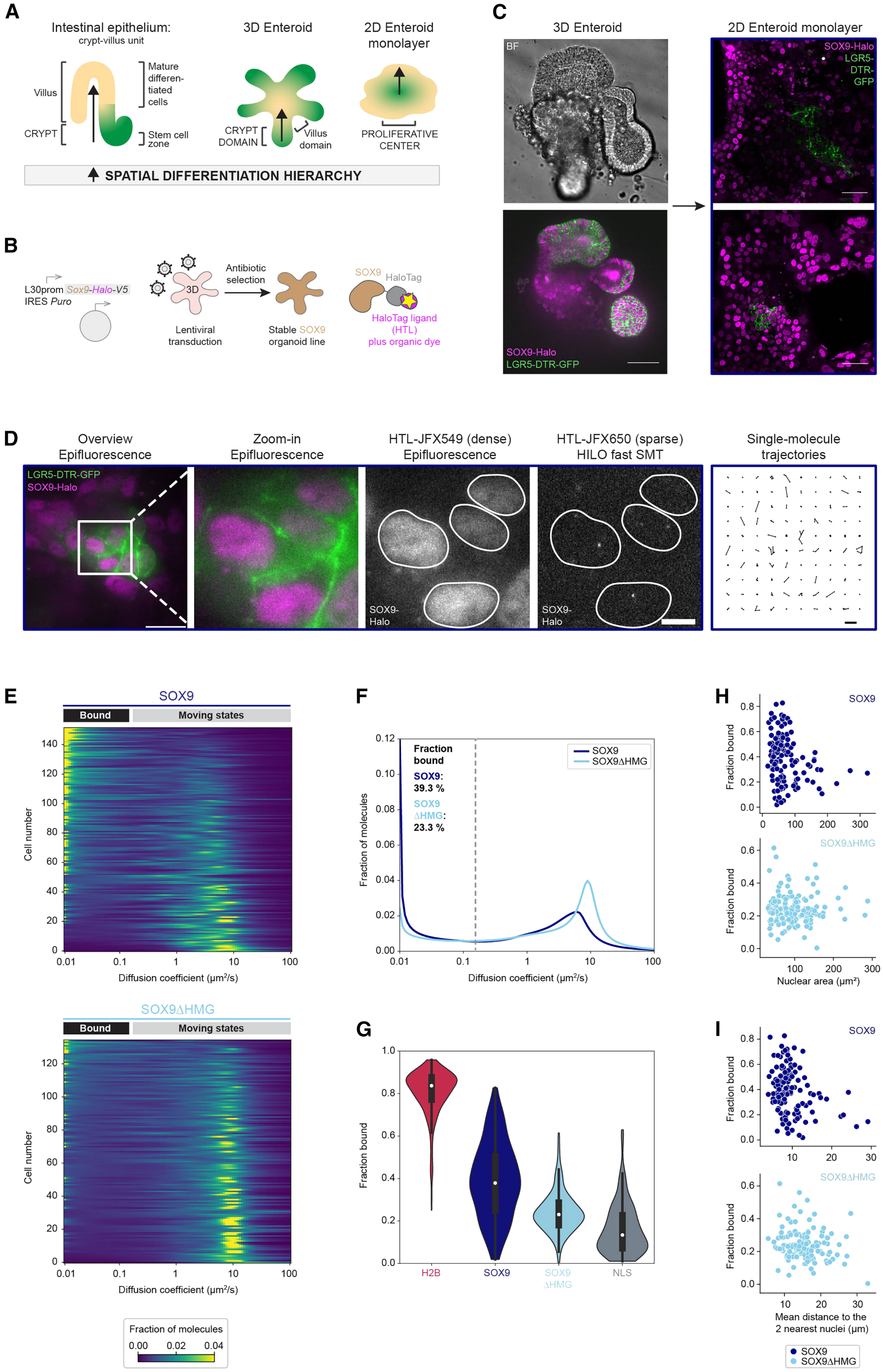

(legend on next page)

multicellular tissue-resembling systems (Lukonin et al., 2020; McKinley et al., 2018; Schöneberg et al., 2018; Serra et al., 2019; Tallapragada et al., 2021), including live-cell TF dynamics at the cellular and single-molecule level (Walther et al., 2024).

TFs diffuse in the nucleus in search of their cognate DNA-binding motifs, to which they eventually bind to regulate target gene expression (Cramer, 2019). Diffusion and binding properties of single TF molecules can be measured in live cells by single-molecule tracking (SMT). In contrast to other bulk fluorescence imaging methods (Boka et al., 2021), SMT directly allows the detection, localization, and tracking of individual molecules, enabling the resolution of subpopulations with different diffusive behaviors within the same cell (Dahal et al., 2023). The obtained diffusion spectra provide the following parameters: (1) the diffusion coefficients of differentially mobile subpopulations (e.g., freely, fast, and slowly diffusing) and (2) the fraction of immobile molecules, which, in the case of TFs, are mainly chromatin-bound (fraction bound) (Dahal et al., 2023; Mazzocca et al., 2021).

Highly inclined and laminated optical sheet (HILO)-based SMT has been used extensively to probe TF dynamics in 2D cell culture systems (Chen et al., 2022; Dahal et al., 2025; Ferrie et al., 2024; Hansen et al., 2017; Hsieh et al., 2022; Kuchler et al., 2022; Mazzocca et al., 2023; Szczurek et al., 2024; Zhou et al., 2023), including cancer cells (Chen et al., 2022; Dahal et al., 2025; Ferrie et al., 2024; Mazzocca et al., 2023), embryonic stem cells (Hansen et al., 2017; Hsieh et al., 2022; Szczurek et al., 2024), primary neurons (Zhou et al., 2023), and distinct cell states obtained through directed differentiation protocols (Esbin et al., 2024; Kuchler et al., 2022), but not yet in a cell-type-resolved manner within a multicellular differentiation context. 2D EMCs, as multicellular differentiation systems, are highly heterogenous since they comprise various cell types. To deal with such complexity, we recently developed an automated SMT imaging and analysis pipeline in 2D EMCs that records hundreds of cells and resolves their heterogeneity in TF diffusion correlated with cellular features indicative of differentiation states (Walther et al., 2024). Additionally, we implemented proximity-assisted photoactivation (PAPA)-SMT in 2D EMCs (Walther et al., 2024), a technique probing molecular interactions in live cells at single-molecule resolution (Graham et al., 2022) and thus resolving diffusion parameters of specific molecular complexes (Dahal et al., 2023; 2025; Graham et al., 2025; Walther et al., 2024).

The TF sex-determining region Y (SRY) box 9 (SOX9) exerts important functions in multiple organs (Barrionuevo et al., 2006; Bi et al., 1999; Ming et al., 2022; Poché et al., 2008; Rockich et al., 2013; Seymour et al., 2007; Thomsen et al., 2008; Vidal et al., 2005), including the intestine (Mori-Akiyama et al., 2007), both during embryonic development and adult tissue differentiation. SOX9

Figure 1. Automated live-cell fast single-molecule tracking in 2D enteroid monolayer cultures reveals a heterogenous diffusive behavior of SOX9-Halo with the fraction of immobile molecules largely depending on DNA binding

(A) In the mammalian intestinal epithelium (left), a spatial differentiation hierarchy (arrow) guides directional movement of differentiating intestinal stem cells (ISCs) along the crypt-villus (green-beige) axis, also reflected in *in vitro* models of the intestinal epithelium: in mouse small intestinal organoids (mSIOs; enteroids; middle), ISCs in the crypt domain at bud tips (green) migrate inward upon differentiation, whereas 2D EMCs (right) grow outward from ISC-containing proliferative centers (green).

(B) Generation of an organoid line stably expressing a SOX9-HaloTag(Halo)-V5 transgene through lentiviral transduction and antibiotic selection allows fluorescence detection (yellow star) of SOX9-Halo covalently labeled with dye-coupled HaloTag ligands (HTLs).

(C) Confocal imaging of mSIOs (left) derived from LGR5::DTR-GFP mice (labeling ISCs/early progenitors [green]) stably expressing SOX9-Halo (magenta) and corresponding 2D EMCs (right) 5 days post-seeding. BF, bright field. Scale bars, 50 μm.

(D) Double labeling of SOX9-Halo 2D EMCs with two different HTLs allows bulk labeling for nuclear segmentation (images 1–3 from left; white masks) and sparse labeling for HILO-based fast SMT (image 4; one representative frame of an SMT movie) resulting in single-molecule trajectories (image 5; 100 randomly selected single-molecule trajectories). Scale bars: 20 μm (overview), 5 μm (zoom-in), 1 μm (trajectories).

(E) Single-cell diffusion heatmaps for 4 combined automated SMT experiments for SOX9-Halo (dark blue; n = 152 cells) and 5 combined manual fast SMT experiments for SOX9ΔHMG-Halo (light blue; n = 135 cells). Cells are ordered by decreasing fraction bound (top-bottom). Representative SMT movies in Videos S1 and S2.

(F) Mean diffusion spectra for SOX9-Halo (dark blue) and SOX9ΔHMG-Halo (light blue). Bootstrap analysis of combined experiments with n = 10, 44, 42, and 56 cells for SOX9 and n = 43, 23, 1, 34, and 34 cells for SOX9ΔHMG determined a mean fraction bound ($D < 0.15\ \mu m^2/s$) of 39.3% (95% confidence interval [CI]: 32.9%–46.7%) and 23.3% (95% CI: 19.5%–28.5%), respectively.

(G) Violin plots for fractions bound (white point: median; whiskers: first/third quartile) of Halo-tagged H2B (red), SOX9 (dark blue), SOX9ΔHMG (light blue), and nuclear localization sequence (NLS; gray). SOX9 and SOX9ΔHMG data are the same as in (E) and (F). Data are from 7 H2B and 3 NLS experiments with combined n = 367 and n = 100 cells (n = 65, 30, 12, 50, 123, 81, and 6 and n = 12, 47, and 41 cells per experiment).

(H and I) Single-cell correlation of SMT-derived fraction bound for SOX9 (top, dark blue) or SOX9ΔHMG (bottom, light blue) with the morphological characteristics nuclear area (H) or mean distance to the two nearest nuclei (I).

See also Figure S1.

mutations and an aberrant SOX9 dosage are implicated in a wide range of diseases, ranging from skeletal dysplasia and sex reversal (Wagner et al., 1994) to colorectal cancer (Abdel-Samad et al., 2011; Prévostel and Blache, 2017).

SOX9 contains a high-mobility group (HMG) box DNA-binding domain (Mertin et al., 1999) and can homodimerize via its dimerization domain (DIM). Dimerization is required for DNA binding and transactivation of cartilage-specific genes (Bernard et al., 2003; Coustry et al., 2010), but it is dispensable in other contexts (Bernard et al., 2003), where SOX9 functions as a monomer.

During mouse intestinal development, SOX9 is expressed in all epithelial cells at E13.5, but it becomes restricted to proliferating progenitor cells at E15.5 (Mori-Akiyama et al., 2007). Upon adulthood, SOX9 is expressed in ISCs, progenitor cells, and secretory Paneth cells (Mori-Akiyama et al., 2007). Here, SOX9 is required for progenitor cell maintenance (Blache et al., 2004) and Paneth cell differentiation (Bastide et al., 2007; Mori-Akiyama et al., 2007), whereby distinct SOX9 expression levels characterize various cell populations within the murine small intestinal crypt (Formeister et al., 2009).

Due to its diverse roles and expression levels during intestinal differentiation and beyond, we chose SOX9 to study how abundance and diffusive behavior of a cell fate-conferring TF change during differentiation. Using our automated SMT pipeline (Walther et al., 2024), we investigated the dynamics of SOX9 under homeostatic conditions in differentiating 2D EMCs. By directly recording cell type markers, we determined an expression-level-independent correlation between the fraction of immobile SOX9 molecules and the progression of differentiation, largely dependent on DNA binding. We further observed that long-term overexpression of SOX9 in mSIOs causes a change in organoid morphology, accompanied by increased proliferation, as well as a loss of intestinal gene expression signatures and the acquisition of a regenerative fetal-like gene expression program. Applying our (PAPA)-SMT pipelines (Walther et al., 2024) to 3D spheroids and spheroid-derived 2D EMCs, we observed increased DNA occupancy by SOX9 and evidence of oligomerization. Our results suggest context-dependent molecular dynamics of SOX9 during differentiation in adult intestinal homeostasis and upon SOX9 overexpression-induced fetal-like reprogramming.

RESULTS

Automated fast SMT in live 2D EMCs reveals heterogenous SOX9-Halo diffusion across differentiating cells independent of expression levels

To use SOX9 for interrogating the diffusive behavior of a cell fate-conferring TF in the intestinal differentiation paradigm (Figure 1A), we used lentiviral transgene delivery (Figure 1B) to generate a stable mSIO line expressing SOX9-Halo from a weak ubiquitous L30 promoter (Chen et al., 2022). Nuclear SOX9-Halo expression in 3D mSIOs and 2D EMCs derived thereof was confirmed by live imaging (Figure 1C) after covalent labeling of the HaloTag (Halo) with HaloTag ligands (HTLs) coupled to bright and photostable dyes (Figure 1B) (Grimm et al., 2017), which was also key for SMT (Figure 1D). Here, we used HILO-based live-cell fast SMT on a total internal reflection fluorescence (TIRF) microscope and employed a stroboscopic illumination scheme to reduce motion blur (Dahal et al., 2023; Hansen et al., 2017). As previously observed for a different lineage TF in this system (Walther et al., 2024), a typical manual fast SMT experiment of 10 randomly selected cells did not provide a conclusive picture about the SOX9-Halo diffusion behavior in these heterogenous EMCs (Figures S1A and S1B), demonstrating the need to acquire larger datasets. We thus used our previously developed automated SMT pipeline (Walther et al., 2024) to acquire hundreds of cells (Figures 1D and 1E, top; Figure S1E). Bulk-labeled SOX9-Halo (Figure 1D, subpanels 1–3) allowed the automated detection of nuclei and triggered an SMT sequence in another channel with SOX9-Halo sparsely labeled with a second HTL-coupled fluorophore (Figure 1D, subpanel 4) to obtain typically hundreds to thousands of single-molecule trajectories with an average trajectory length of ~3–5 localizations (Walther et al., 2024) (Figure 1D, subpanel 5; for tracking statistics, see methods). Automated SMT in hundreds of randomly chosen cells confirmed a large heterogeneity in the diffusive behavior of SOX9-Halo with cellular diffusion peaks ranging from freely diffusing (D ~10 $\mu m^2/s$) to immobile (D ~0.01 $\mu m^2/s$) (Figure 1E, top; Figure S1E) and an average fraction of immobile ($D < 0.15$ $\mu m^2/s$; diffusion behavior indistinguishable from that of histone H2B-Halo (Walther et al., 2024; for details, see methods) SOX9-Halo molecules of 39.3% (95% confidence interval [CI]: 32.9%–46.7%; Figures 1F and 1G). Notably, cell-to-cell differences in the fraction bound covered the whole spectrum between the average fractions bound determined for the immobile H2B-Halo (83.7%; 95% CI: 81.4%–85.9%) and freely diffusing Halo-nuclear localization sequence (NLS; 19.2%; 95% CI: 15.0%–23.2%) controls (CTRLs) (Figures 1G and S1I). On a cell-by-cell basis, the fractions bound of SOX9-Halo did not correlate with the average nuclear SOX9-Halo intensity (Figure S1G), a proxy for transgenic SOX9-Halo levels, arguing against heterogeneity being caused by variable transgene expression in the initially polyclonal organoid line.

Immobile SOX9-Halo molecules largely reflect DNA binding

A plausible explanation for the observation of immobile states of TFs in the cell nucleus is TF binding to DNA. To

test whether this is the case for the immobile SOX9-Halo molecules detected by SMT, we transiently expressed a SOX9-Halo mutant lacking the HMG DNA-binding domain (SOX9ΔHMG-Halo) in wild-type (WT) organoid-derived 2D EMCs via recombinant adeno-associated viral (rAAV) vector delivery (Benyamini et al., 2023) (Figures S1C and S1D). Automated fast SMT of SOX9ΔHMG-Halo revealed a more uniform diffusive behavior (Figure 1E, bottom; Figures 1G and S1F) with a lower average fraction bound of 23.3% (95% CI: 19.5%–28.5%) compared to full-length SOX9-Halo (Figures 1F and 1G; *p* value of fraction bound distribution comparison between SOX9 and SOX9ΔHMG: 7.03e−14) independent of its nuclear expression level (Figure S1H). Hereby, most cells were characterized by a diffusion peak in the freely diffusing range, confirming that the measured immobile fraction largely represents SOX9-Halo molecules bound to DNA. Nevertheless, the average fraction bound of SOX9ΔHMG-Halo did not fully decrease to the 19.2% determined for the Halo-NLS freely diffusing CTRL (Figures 1G and S1I), suggesting that additional SOX9 protein domains might contribute to the bound fraction measured by SMT.

Cells with more immobile SOX9-Halo molecules display morphological features of intestinal stem/early progenitor cells in 2D EMCs

Hypothesizing that the observed cell-to-cell heterogeneity in the diffusive behavior of SOX9-Halo might reflect differentiation states, we inspected our single-cell diffusion data with respect to nuclear area and the mean distance of a nucleus to its two nearest nuclei, two parameters extracted from our images that we previously found to be correlated with differentiation (Walther et al., 2024). For SOX9-Halo, we indeed identified a subpopulation of cells with smaller nuclei or a smaller nearest-nuclei distance, indicative of stem/early progenitor cells in proliferative centers (Walther et al., 2024) (Figure 1C, right), which was characterized by larger fractions bound (Figure 1H, top). In contrast, a subpopulation of cells with larger nuclei or a larger nearest-nuclei distance, indicative of differentiated cells further away from proliferative centers (Walther et al., 2024) (Figure 1C, right), was characterized by a smaller fraction bound (Figure 1I, top). These subpopulations with distinct diffusive and morphological features were not discernible for the SOX9ΔHMG-Halo mutant (Figures 1H and 1I), suggesting that the immobile fraction of SOX9-Halo in stem/early progenitor cells mainly reflects DNA binding.

DNA binding of SOX9-Halo decreases upon intestinal differentiation

To directly test our observation of distinct diffusive subpopulations with morphological characteristics indicative of differentiation states, we implemented the recording of fluorescent cell type markers and marker-based cell classification in our SMT pipeline (Walther et al., 2024). Using the green fluorescent ISC/early progenitor marker LGR5, present in our SOX9-Halo organoid line derived from LGR5::DTR-GFP mice (Tian et al., 2011), we classified the SOX9-Halo population into LGR5-positive ($LGR5^{+}$) stem/early progenitor cells and LGR5-negative ($LGR5^{-}$) late progenitor/differentiated cells (Figure 2A). The average fraction bound of SOX9-Halo decreased during differentiation from 48.2% (95% CI: 44.2%–52.3%) in stem/early progenitor cells to 38.1% (95% CI: 31.9%–44.5%) in late progenitor/differentiated cells (Figures 2B and 2C, right; Figures S2A, S2B, and S2C, bottom; *p* value of SOX9 fraction bound distribution comparison between $LGR5^{+/-}$: 0.055). Such decrease was also apparent upon hierarchical clustering of $LGR5^{+/-}$ single-cell diffusion spectra together (Walther et al., 2024) (Figures S2D–S2G). Notably, cell-to-cell variability persisted within both $LGR5^{+/-}$ subpopulations (Figure 2C, right; Figures S2A, S2B, and S2C, bottom), indicating a potentially more complex grading of the SOX9 diffusion behavior into cell states and types within these heterogenous subpopulations. As observed for the whole population (Figure S1G), the differences in the diffusive behavior of SOX9-Halo were independent of its expression level in both $LGR5^{+/-}$ subpopulations (Figure 2D). Importantly, for both H2B-Halo and Halo-NLS immobile and freely diffusing CTRLs, respectively, no difference in the diffusive behavior was measured between $LGR5^{+/-}$ subpopulations (Figures S2H and S2I; *p* values of fraction bound distribution comparisons between $LGR5^{+/-}$: H2B, 0.125; NLS, 0.914), further supporting that the observed difference in the SOX9-Halo diffusion behavior stems from differentiation and not from other differences between stem and differentiated cells, such as nuclear size or crowding. Notably, the $LGR5^{+}$ subpopulation was characterized by smaller nuclei and a smaller nearest-nuclei distance in comparison to the $LGR5^{-}$ subpopulation (Figure 2C, left; Figure S2C, top), further validating our cell morphology-based approach for an approximated discrimination of undifferentiated from differentiated cells in 2D EMCs (Walther et al., 2024) (Figures 1H and 1I). Nevertheless, we noted some $LGR5^{-}$ cells with smaller nuclei and larger fractions bound (Figure 2C), for which one possible explanation could be differentiated Paneth cells residing within proliferative centers. Taken together, consistent with SOX9 functioning and natively being expressed in the proliferative intestinal crypt, our results suggest that the $LGR5^{+}$ subpopulation is characterized by more SOX9-Halo molecules bound to DNA, possibly resulting from the occupancy of more DNA-binding sites.

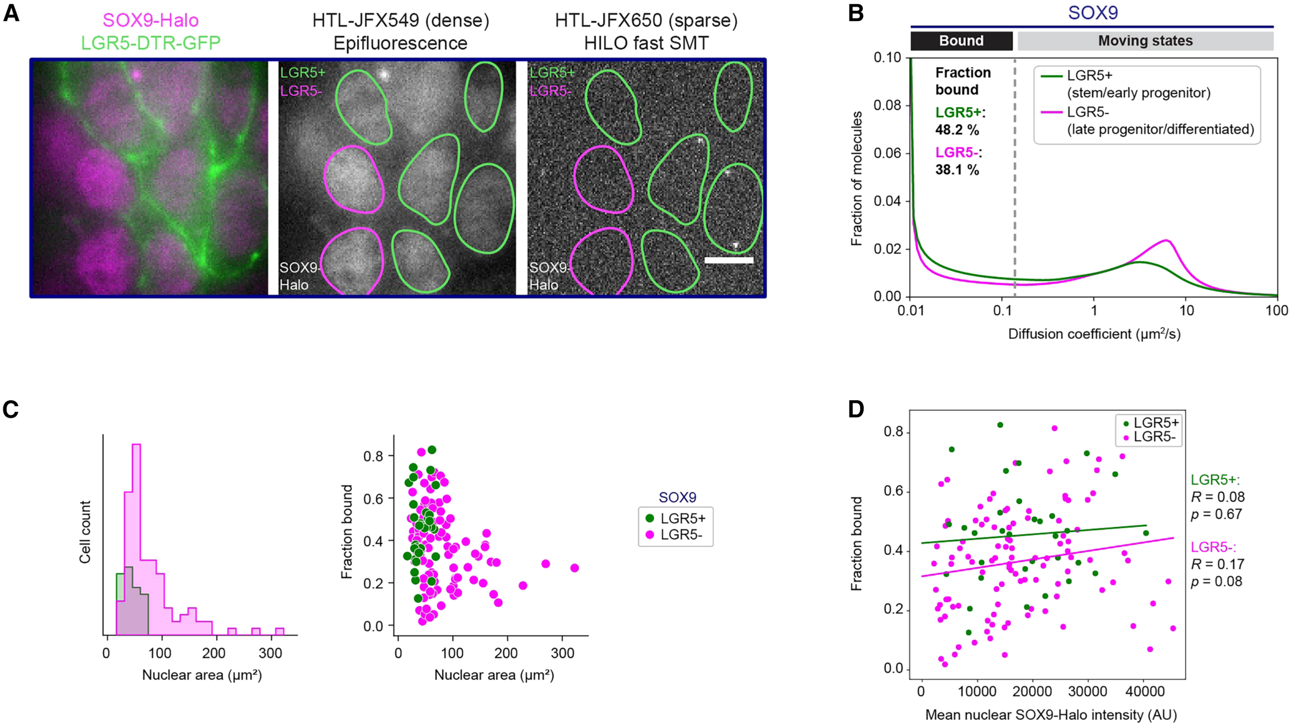

Figure 2. LGR5-based distinction of differentiation states demonstrates an expression-level-independent decrease in the fraction of immobile SOX9-Halo molecules upon differentiation

(A) The green fluorescent LGR5-DTR-GFP marker enables classification into stem/early progenitor ($LGR5^+$) and late progenitor/differentiated cells ($LGR5^-$) for SMT in 2D EMCs. Left: epifluorescence of bulk-labeled SOX9-Halo (magenta) and LGR5-DTR-GFP (green); middle: epifluorescence of bulk-labeled SOX9-Halo (gray) with segmented nuclear masks color-coded according to classification into $LGR5^{+/-}$ (green/magenta) categories; right: representative frame of a fast SMT movie of sparsely labeled SOX9-Halo (gray) with same classified masks depicted. Scale bars: 5 μm. Representative SMT movie in Video S3.

(B) Mean diffusion spectra of SOX9-Halo for $LGR5^{+/-}$ (green/magenta) subpopulations. Bootstrap analysis of 4 combined experiments with $n = 3$, 21, 2, and 10 $LGR5^+$ and $n = 7$, 23, 40, and 46 $LGR5^-$ cells determined a mean fraction bound of 48.2% (95% CI: 44.2%–52.3%) and 38.1% (95% CI: 31.9%–44.5%), respectively. Comparison of the fraction bound distributions between $LGR5^+$ and $LGR5^-$ cells yielded a p value of 0.0554.

(C) Left: nuclear area distribution of $LGR5^{+/-}$ (green/magenta) cells extracted from SMT data. Right: single-cell correlation of SMT-derived fraction bound with nuclear area for $LGR5^{+/-}$ cells.

(D) Fractions bound for each cell plotted against the mean nuclear SOX9-Halo intensity for $LGR5^{+/-}$ cells. Correlations (fitted lines) were computed for each subpopulation; Pearson correlation coefficients (R) and p values are indicated.

The data in (B)–(D) are the same as in Figures 1E–1I, 5C, S1E, S1G, S1I, and S2A–S2G. See also Figure S2.

Long-term overexpression of SOX9-Halo results in a proliferative cell state transition accompanied by an organoid morphology change to spheroids devoid of signatures for intestinal identity and function

Prolonged culture of a stable SOX9-Halo organoid line resulted in progressive changes in organoid morphology: starting from the initially typical budding morphology (until ∼10 weeks after line establishment [LE]), organoids got rounder with smaller buds (∼15–25 weeks after LE) and finally completely spherical (∼30 weeks after LE) (Figures 3A and S3A, left). In contrast, non-transduced WT organoids continued to grow as budding structures until our longest observed culture time of ∼22 weeks (Figure 3A). This morphological change in 3D SOX9-Halo organoids was also reflected in 2D EMCs derived from them, which were characterized by smoother edges of the monolayer in comparison to WT EMCs (Figure 3A) and grew to complete confluency (Figure S3A, right), suggesting a loss in cell contact inhibition. While SOX9 expression was no longer restricted to proliferative centers (Figure 3B, left) (Mori-Akiyama et al., 2007), as expected for ubiquitous SOX9-Halo transgene expression, the average expression level of total (endogenous plus transgene) SOX9 protein in spheroids was only ∼2.3× higher than the endogenous SOX9 level in proliferative centers of WT organoids (Figure 3C, left), while the average SOX9-Halo

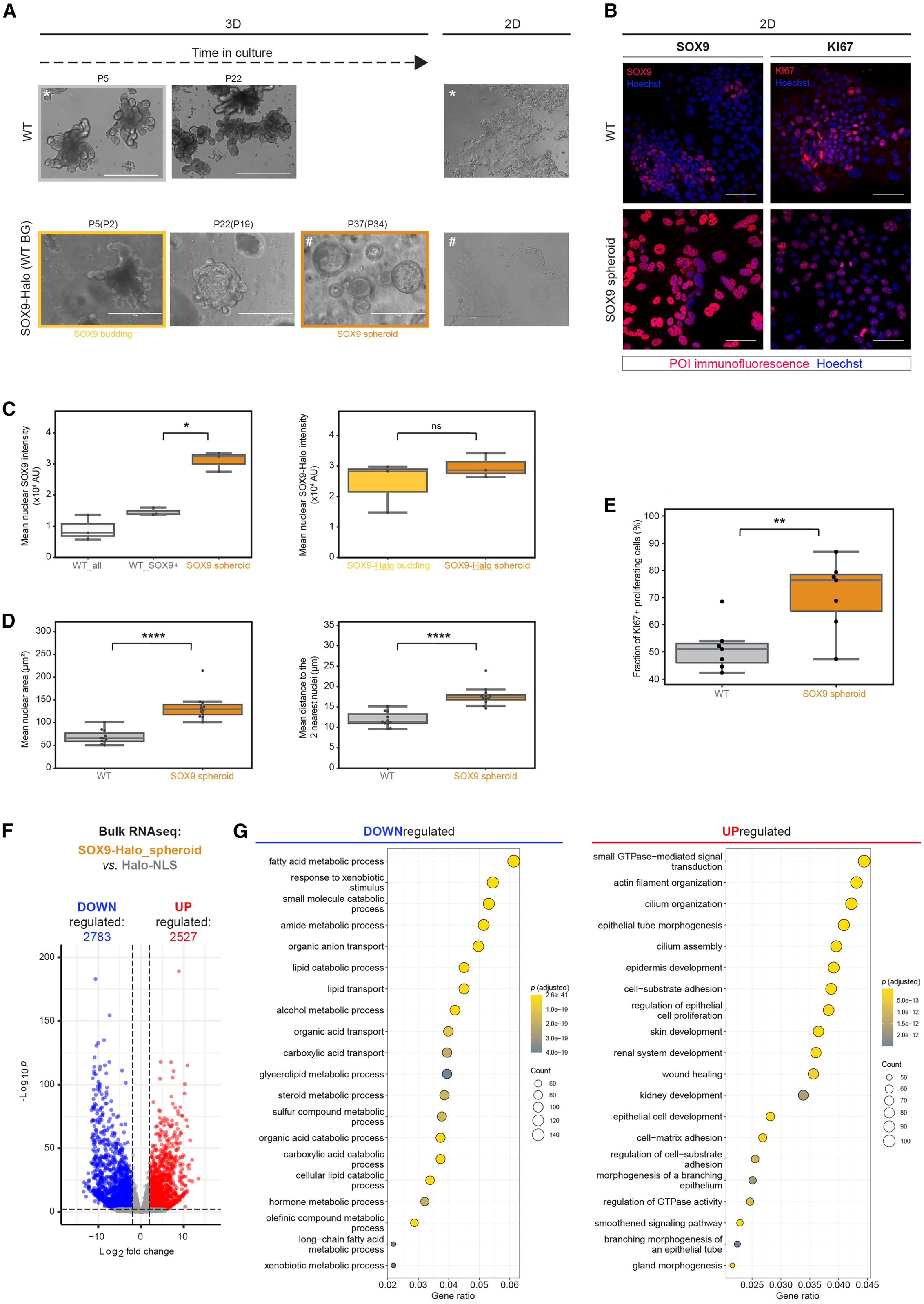

(legend on next page)

transgene expression level was similar to that in budding SOX9-Halo organoids (Figure 3C, right). Notably, SOX9 spheroids were characterized by on average larger nuclei (Figure 3D, left) and a larger nearest-nuclei distance (Figure 3D, right) than WT organoids, indicative of a more differentiated cell population (Walther et al., 2024) (Figures 2C and S2C). Despite such morphology, spheroids contained more (76.4%) proliferative cells than WT organoids (51.1%) as determined by immunofluorescence (IF) for the proliferation marker KI67 (Miller et al., 2018) (Figures 3B, right, and 3E), consistent with the described role for SOX9 in maintaining proliferative progenitor cells (Blache et al., 2004). This increased proliferative capacity was accompanied by several aberrant cell division phenotypes, e.g., fusion of two or more nuclei, micronuclei, and fragmented nuclei (Figure S3B).

To reconcile the seemingly opposing effects of long-term SOX9-Halo overexpression, we performed bulk RNA sequencing (RNA-seq) of SOX9-Halo organoids in comparison to CTRL organoids stably expressing Halo-NLS, revealing thousands of differentially expressed genes (DEGs) (Figure 3F). Gene Ontology (GO) analysis of the 2,783 genes downregulated in spheroids revealed the top 20 biological processes to be metabolic and metabolite transport processes (Figure 3G, left), pointing toward a loss of the intestinal epithelial function in nutrient absorption and metabolism. Conversely, GO analysis of the 2,527 upregulated genes revealed pronounced association with epithelial morphogenesis and the development and differentiation of various non-intestinal tissues and organs (Figure 3G, right), indicating a loss in intestinal identity and the activation of a gene expression program resembling embryonic-like development. Wound healing, cell adhesion, actin filament organization, and cilium assembly were among other biological processes associated with genes upregulated in spheroids (Figure 3G, right), some of which we phenotypically confirmed by IF, including the formation of actin stress fibers and membrane spikes (Figure S3C). These results altogether suggest a large functional and epithelial rearrangement underlying SOX9-Halo spheroid formation.

SOX9 overexpression results in a transient upregulation of stem cell markers followed by YAP activation and a regenerative fetal-like gene expression program

Given the extensive gene expression rearrangement in SOX9 spheroids, we questioned whether the HaloTag fused to the SOX9 transgene might have contributed to its emergence. Reassuringly, the budding-to-spheroid morphology transition was reproduced in a stable SOX9-mEGFP organoid line (Figure 4A) on a similar timescale, ruling out potential effects due to the introduced HaloTag. To further compare the two stable SOX9 organoid lines and gain insights into the gene expression changes underlying organoid morphology transition, we selected one budding and one spheroid phenotype for each line and ordered them phenotypically based on the progression of spheroid morphology

Figure 3. Long-term overexpression of SOX9-Halo in enteroids results in an organoid morphology change coinciding with a proliferative cell state transition and a loss of gene expression signatures for intestinal identity and function

(A) In contrast to wild-type (WT) organoids (top), stable SOX9-Halo organoids (WT background; bottom) transitioned from budding to spheroid morphology upon long-term culture. This morphology change of 3D organoids (left) is also reflected in 2D EMCs derived from them (right). Epifluorescence images 5 days post-seeding with organoid passage number P(P) after crypt isolation (after lentiviral transduction to make stable line) indicated. */# denotes the passage of 2D EMC derivation from 3D organoids. Scale bars: 400 μm.

(B) Confocal images of 2D EMCs derived from WT organoids (top) or SOX9-Halo spheroids (bottom) immunostained (red) for SOX9 (left) or the proliferation marker KI67 (right) and co-stained with Hoechst (blue) 5 days post-seeding. Scale bars: 50 μm.

(C) Quantification of total (left) or transgene (right) SOX9 protein levels in 2D EMCs derived from WT (gray), SOX9-Halo_budding (yellow), or SOX9-Halo_spheroid (orange) organoids based on fixed and immunostained (left) or live and HTL-stained (right) confocal images 5 days post-seeding. Total SOX9 levels: 3 FOVs with 613 cells (309 $SOX9^+$, 304 $SOX9^-$) (WT), 3 FOVs with 337 cells (SOX9_spheroid). Transgene SOX9-Halo levels: 3 FOVs with 864 cells (SOX9_budding), 3 FOVs with 537 cells (SOX9_spheroid).

(D) Quantification of nuclear area (left) and mean distance to the two nearest nuclei (right) in 2D EMCs derived from WT organoids (gray) or SOX9-Halo spheroids (orange) 5 days post-seeding based on confocal images (WT: 13 FOVs, 1,821 cells; SOX9_spheroid: 16 FOVs, 1,681 cells).

(E) Percentage of $KI67^+$ proliferative cells in 2D EMCs derived from WT organoids (gray) or SOX9 spheroids (orange) quantified from confocal images as in (B). WT: 7 FOVs, 752 cells; SOX9_spheroid: 7 FOVs, 648 cells. For (C)–(E), each point represents one FOV; median: gray line, first/third quartile: whiskers; statistical testing based on Mann-Whitney U tests (see methods for details); ns, non-significant, $p > 0.5$; *$p \leq 0.5$; **$p \leq 0.01$; ****$p \leq 0.0001$.

(F and G) Bulk RNA-seq experiment of SOX9-Halo spheroids versus Halo-NLS control (CTRL) organoids in biological triplicates. (F) Volcano plot displaying differentially expressed genes (DEGs; adjusted p value ≤ 0.01, fold change ≥ 2 and mean counts ≥ 10; red/blue: up-/downregulated). (G) Gene ontology (GO) analysis of the top 20 biological pathways enriched in DEGs down- (left, blue) or upregulated (right, red) with adjusted p values and gene counts indicated.

Bulk RNA-seq data are the same as for Halo-NLS and SOX9-Halo spheroid samples in Figures 4B–4D, S4, and S5. See also Figure S3.

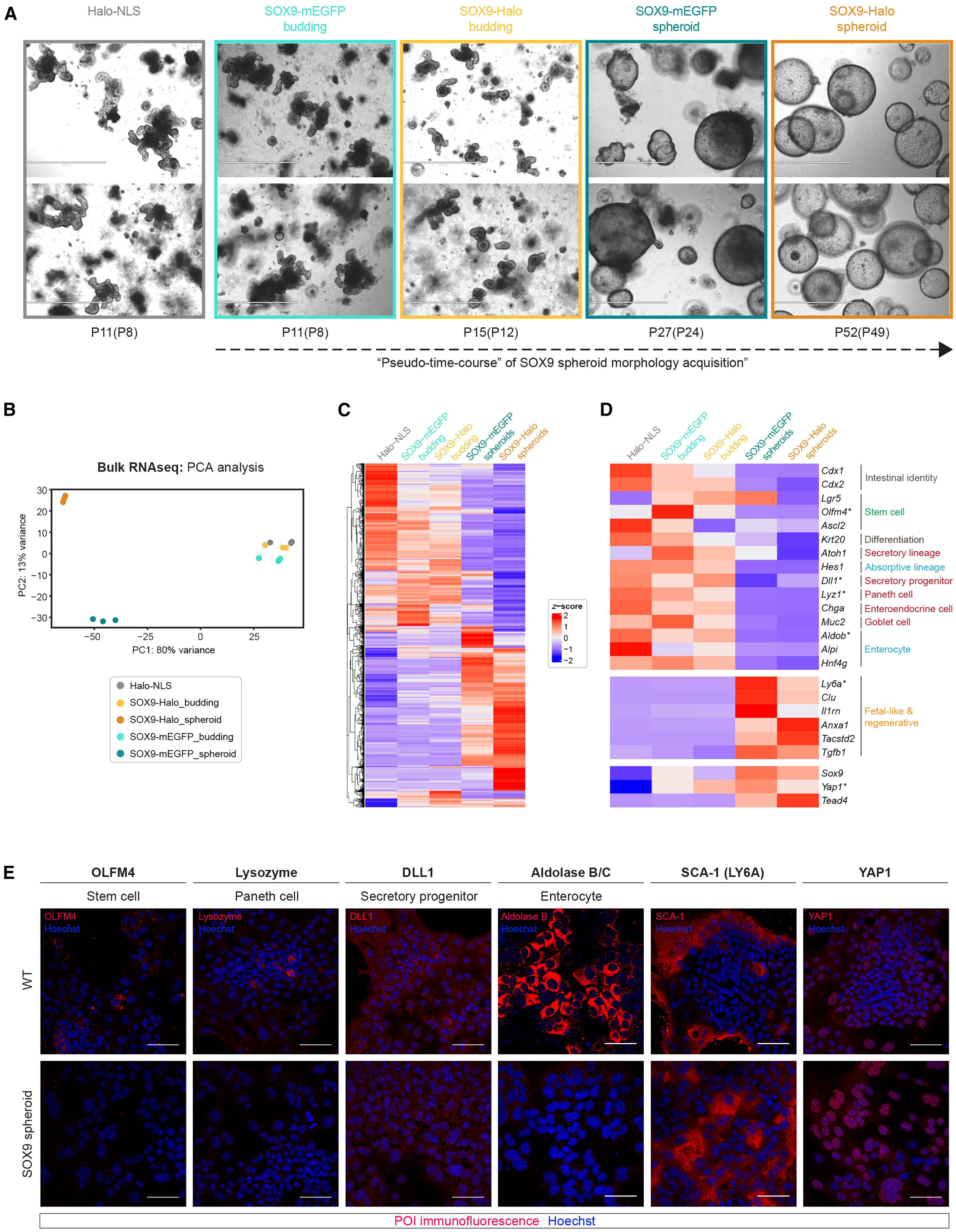

(legend on next page)

acquisition, which also correlated with their time in culture (Figure 4A). The addition of three intermediate phenotypes (SOX9-mEGFP_budding, SOX9-Halo_budding, and SOX9-mEGFP_spheroid) (Figure 4A) to our previously analyzed SOX9-Halo spheroid and Halo-NLS organoid samples (Figures 3F and 3G) allowed us to perform a morphology "pseudo-time-course analysis" (Figure 4A). Both spheroid samples were very different from the two budding and the Halo-NLS CTRL samples (Figure S4A), which clustered together upon principal-component analysis (PCA) (Figure 4B). Nevertheless, comparing SOX9-mEGFP versus SOX9-Halo budding and spheroid samples, about a hundred (Figure S4B) or one thousand DEGs (Figure S4C) were detected, respectively, confirming that gene expression changes underlie the slightly different morphological phenotypes (Figure 4C) and ultimately lead to SOX9 spheroid formation (Figures 3F and 4A).

Focusing on selected markers of intestinal epithelial lineages, we confirmed our initial GO analysis results (Figure 3G) with a loss of expression in intestinal identity genes, stem cell markers, and markers of absorptive and secretory lineages (Beumer and Clevers, 2021) (Figures 4D and S5A), some of which we validated at the protein level by IF (Figure 4E). Inspecting more carefully the three samples Halo-NLS, SOX9-Halo_budding, and SOX9-Halo_spheroid revealed an initial mild gene expression deregulation (Figure S4D) followed by a second wave of major entity (Figure S4F). While both of these transitions were characterized by an overall downregulation of metabolic and metabolite transport processes (Figures S4E and S4G, left), including digestion for the second transition (Figure S4G left), genes upregulated in the first transition were associated with the Wnt signaling pathway and its regulation (Figure S4E, right), followed by upregulation of genes involved in development and differentiation of various non-intestinal tissues and organs in the second transition (Figure S4G, right). This transient upregulation of genes associated with Wnt signaling was in agreement with a transient increase in canonical stem cell markers including *Olfm4* (van der Flier et al., 2009) and the Wnt target gene *Lgr5* (Barker et al., 2007) in budding SOX9 organoids (Figures 4D and S5A). Instead of intestinal epithelial genes, spheroids were characterized by the expression of several genes previously associated with fetal-like and regenerative signatures (e.g., *Ly6a*, *Clu*, and *Anxa1*) (Figures 4D, S5B, and 4E), typically arising upon tissue regeneration after exposure of the intestinal epithelium to various sources of damage (Viragova et al., 2024).

The effector of the Hippo signaling pathway, Yes-associated protein (YAP1), has been associated with intestinal regeneration (Gregorieff et al., 2015; Namoto et al., 2024; Serra et al., 2019). While mRNA levels of *Yap1* were not significantly altered in the course of SOX9 spheroid phenotype acquisition (Figure S5C), 2D EMCs derived from these spheroids were characterized by an overall nuclear localization of YAP1 (Figure 4E), indicative of YAP1 activation (Yu et al., 2015). Furthermore, inspection of our bulk RNA-seq data revealed an increased expression of *Tead4* (Figures 4D and S5C), encoding a TEA domain (TEAD) TF interacting with nuclear YAP1 and thereby activating downstream genes (Zhao et al., 2008).

Taken together, our "pseudo-time-course" of spheroid morphology acquisition upon SOX9 overexpression revealed that a loss of adult homeostatic intestinal identity signatures was counteracted by a gain in fetal-like regenerative signatures. Hereby, a transient activation of Wnt signaling seems to be accompanied by a persistent activation of YAP1 signaling, resulting in a regenerative fetal-like reprogrammed phenotype.

Increased DNA binding of SOX9-Halo in SOX9 spheroid-derived EMCs in an expression-level-dependent manner

To investigate whether the diffusive behavior of SOX9-Halo changed during fetal-like reversion, we performed automated fast SMT in 2D EMCs derived from SOX9-Halo spheroids (Figure 5A). Here, the diffusive behavior of SOX9-Halo was more uniform with a dominating immobile diffusion peak for most of the about 500 cells measured (Figures 5B and S6A). Indeed, SOX9-Halo in

Figure 4. A "pseudo-time-course" of spheroid morphology acquisition upon SOX9 overexpression reveals a transient increase in stem cell markers followed by a reduction in intestinal epithelial signatures toward a fetal-like reversion

(A) Representative images of the stable organoid lines Halo-NLS (gray), SOX9-mEGFP_budding (light turquoise), SOX9-Halo_budding (yellow), SOX9-mEGFP_spheroid (dark turquoise), and SOX9-Halo_spheroid (orange) at the indicated passage numbers P(P) after crypt isolation (after lentiviral transduction to make stable lines) 5 days post-seeding, chronologically ordered. Scale bars: 1 mm.

(B) Principal-component analysis (PCA) of bulk RNA-seq experiments of the samples (biological triplicates) in (A).

(C and D) *Z* score heatmaps of all DEGs (C) or selected genes (D) determined by bulk RNA-seq (adjusted p value ≤ 0.01, fold change ≥ 2 and mean counts ≥ 10; red/blue: up-/downregulated).

(E) Confocal images of immunostained (POI, protein of interest) 2D EMCs derived from WT organoids (top) or SOX9-Halo spheroids (bottom) co-stained with Hoechst (blue) 5 days post-seeding. Scale bars: 50 μm.

RNA-seq data in (B)–(D) are the same as in Figures S4 and S5. RNA-seq data from Halo-NLS and SOX9-Halo_spheroid samples are the same as in Figures 3F and 3G. See also Figures S4 and S5.

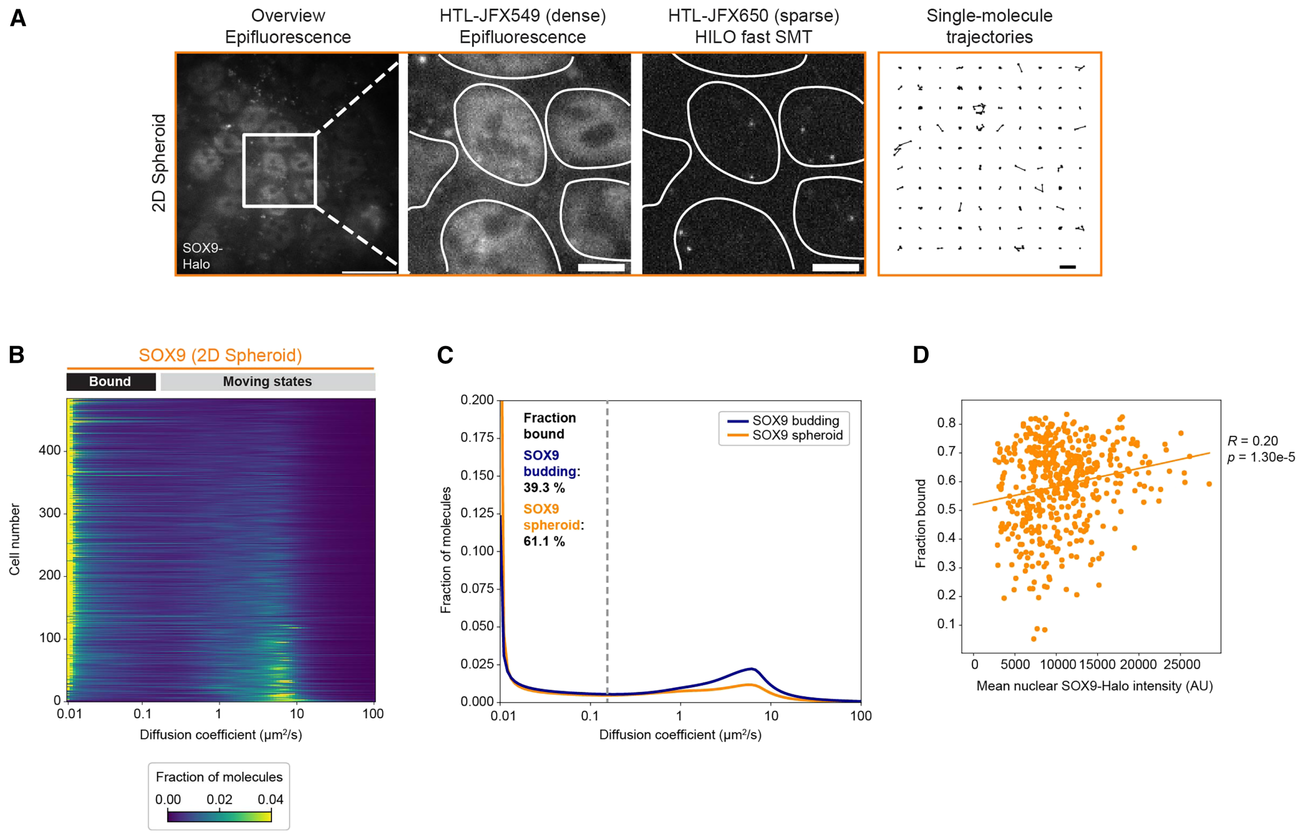

Figure 5. Automated fast SMT in SOX9 spheroid-derived EMCs reveals an expression-level-dependent increase in the fraction of immobile SOX9-Halo molecules

(A) Double labeling of SOX9-Halo (gray) with two different HTLs allows bulk labeling (images 1, 2 from left) for nuclear segmentation (images 2, 3; white masks) and sparse labeling for fast SMT (image 3; one representative frame of an SMT movie) resulting in single-molecule trajectories (image 4; 100 randomly selected single-molecule trajectories). Scale bars: 20 μm (overview), 5 μm (zoom-in), 1 μm (trajectories).

(B) Single-cell diffusion heatmap for 10 combined automated SMT experiments for SOX9-Halo in spheroid-derived 2D EMCs (n = 485 cells with 77, 73, 13, 24, 30, 58, 81, 71, 26, and 32 cells per experiment). Cells are ordered by decreasing fraction bound (top to bottom). Representative SMT movie in Video S6.

(C) Mean diffusion spectra for SOX9-Halo in 2D EMCs derived from budding organoids (LGR5::DTR-GFP background; dark blue) or spheroids (WT background; orange). Bootstrap analysis of combined experiments with n = 10, 44, 42, and 56 cells for SOX9-Halo_budding and n = 77, 73, 13, 24, 30, 58, 81, 71, 26, and 32 cells for SOX9-Halo_spheroid determined a mean fraction bound of 39.3% (95% CI: 32.9%–46.7%) and 61.1% (95% CI: 55.4%–66.1%), respectively. Comparison of the fraction bound distributions between SOX9_budding and SOX9_spheroid yielded a p value of 3.39e−28.

(D) Fractions bound for each spheroid-derived cell plotted against the mean nuclear SOX9-Halo intensity. The correlation (fitted line) was computed; Pearson correlation coefficient (R) and p value are indicated.

The SMT data for SOX9_budding are the same as in Figures 1E–1I, 2B–2D, S1E, S1G, S1I, and S2A–S2G. See also Figure S6.

2D EMCs derived from spheroids was characterized by a higher fraction bound of 61.1% (95% CI: 55.4%–66.1%) in comparison to 39.3% in those derived from budding organoids (Figures 5C, S6A, and S6B; p value of fraction bound distribution comparison between SOX9_budding and SOX9_spheroid: 3.39e−28), raising the possibility that more SOX9 molecules bound to DNA served as a molecular driver of the observed proliferative cell state transition underlying spheroid formation. This increase in fraction bound was also apparent upon hierarchical clustering of the fast SMT data (Figures S6C–S6F). Notably, unlike the budding state, in the spheroid state, we measured a modest positive correlation between SOX9-Halo expression levels and fraction of immobile molecules (Figure 5D), indicating that, despite the presence of more SOX9-Halo molecules (Figure 3C), a larger fraction of them was immobile in the cell nucleus and thus presumably DNA-bound.

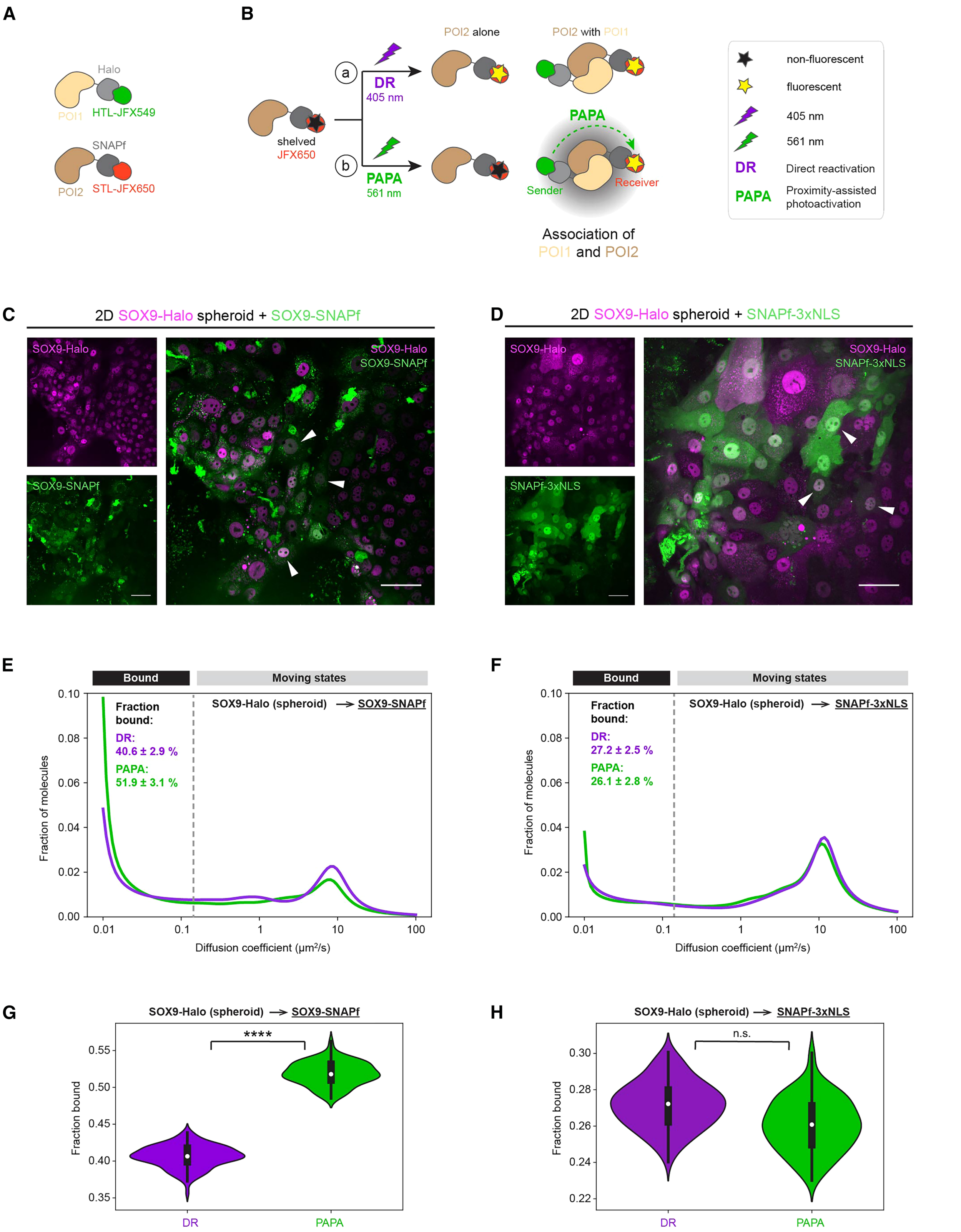

(legend on next page)

PAPA-SMT reveals a chromatin-bound pool of self-associated SOX9 in live 2D EMCs

The increased fraction of SOX9 molecules bound to DNA in spheroids raised the question of whether SOX9 molecules might bind to DNA in an oligomerized state, as both SOX9 monomers and dimers have been reported as transcriptionally active units depending on the differentiation context (Bernard et al., 2003; Coustry et al., 2010). To address this, we employed PAPA-SMT, a recently developed single-molecule method allowing to detect whether differentially labeled sender and receiver molecules (Figure 6A) are associated or in vicinity to each other by using a PAPA illumination scheme (PAPA) distinct from that used in standard SMT (direct reactivation [DR]) (Figure 6B). To this end, we transiently expressed SOX9-SNAPf (Figure 6C) or a SNAPf-3xNLS CTRL (Figure 6D) via rAAV-based delivery (Benyamini et al., 2023) in SOX9-Halo spheroid-derived EMCs. PAPA-SMT using SOX9-Halo as a sender and SOX9-SNAPf as a receiver (Figure 6C) revealed both freely diffusing and immobile oligomerized states (Figure 6E) with a ∼11% higher bound fraction for PAPA versus DR trajectories (Figures 6E and 6G), indicating a chromatin-bound pool of oligomerized SOX9. In contrast, no difference between the bound fractions of PAPA versus DR trajectories was detected for CTRL experiments with SNAPf-3xNLS as the receiver, measuring non-specific background PAPA (Graham et al., 2025) (Figures 6F and 6H). Notably, self-associated SOX9 was also found to be partially chromatin bound in both undifferentiated and differentiated cells of 2D EMCs derived from budding SOX9-Halo organoids (Figure S7), arguing against DNA binding of an oligomerized form of SOX9 as a feature exclusive to spheroids. We nevertheless cannot exclude the existence of differences in the fraction of SOX9 molecules present in an oligomerized state or the number of SOX9 units that DNA-bound SOX9 oligomers consist of, as this PAPA-SMT assay does not provide information on whether more or larger SOX9-Halo oligomers might be immobile in the spheroid compared to the budding state.

Fast SMT in 3D SOX9 spheroids confirms increased SOX9 binding upon fetal-like reversion

Finally, we sought to confirm the SOX9-Halo diffusive behavior observed in spheroid-derived 2D EMCs directly in 3D spheroids. As our HILO-based SMT approach is limited to the 10–20 μm of the sample directly above the culture glass surface, we optimized our organoid seeding procedure so that growing round spheroids could reach the glass surface, making them amenable to SMT. Using an experimental approach analogous to 2D EMCs (Figure 7A), SMT in spheroids revealed a rather homogenous diffusive behavior of SOX9-Halo across the 35 manually imaged cells (Figures 7B and 7C) with most of them being characterized by an immobile diffusion peak (Figure 7B). With an average fraction bound of 65.6% (95% CI: 62.5%–68.8%) (Figure 7C), the diffusive behavior of SOX9-Halo in 3D spheroids is thus in agreement with that measured in spheroid-derived 2D EMCs (Figure 5C; p value of fraction bound distribution comparison between 2D and 3D SOX9_spheroid: 0.435).

DISCUSSION

In this study, we combined *in vitro* models of the rapidly renewing small intestinal epithelium (Figure 1A) with automated live-cell SMT (Walther et al., 2024) to investigate

Figure 6. Proximity-assisted photoactivation-SMT reveals a chromatin-bound pool of self-associated SOX9 in spheroid-derived live 2D EMCs

(A) Differential labeling of POI1-Halo (beige-light gray) and POI2-SNAPf (brown-dark gray) with different HTL/SNAPfTag ligand (STL)-coupled fluorophores (JFX549-sender, green; JFX650-receiver, red) for PAPA-SMT.

(B) Principle underlying PAPA-SMT: through excitation using 639 nm light, shelved JFX650-labeled receiver molecules (red; black star) can either undergo (a) direct reactivation (DR; purple) upon illumination with a violet 405 nm light pulse (purple lightening arrow) making them detectable independent of any potential oligomerization states as in standard SMT, or (b) PAPA (green) upon illumination with a green 561 nm light pulse (green lightening arrow) making them only detectable if in proximity (gray cloud) to a JFX549-labeled sender molecule (green).

(C and D) Confocal images of SOX9-Halo spheroid-derived 2D EMCs transduced with crude recombinant adeno-associated viral (rAAV) vectors for transient expression of (C) SOX9-SNAPf or (D) SNAPf-3xNLS (CTRL) for PAPA experiments (sender: SOX9-Halo [magenta]; receiver: SNAPf-tagged component [green]). Scale bars: 50 μm.

(E and F) Mean PAPA (green) versus DR (purple) diffusion spectra for PAPA-SMT of SOX9-Halo_spheroid→SOX9-SNAPf (E) or SOX9-Halo_spheroid→SNAPf-3xNLS (F) in 2D EMCs.

(G and H) Violin plots for fractions bound (white point: median; whiskers: first/third quartile) of SOX9→SOX9 (G) and SOX9→NLS (H) determined from DR/PAPA (purple/green) trajectories.

Data in (E) and (G) are from 4 combined experiments with $n = 129$ cells ($n = 35$, 30, 28, and 36 cells; 14,156 DR and 9,255 PAPA trajectories; bootstrapped fractions bound: 40.6% ± 2.9% [DR], 51.9% ± 3.1% [PAPA]). Data in (F) and (H) are from 3 combined experiments with $n = 70$ cells ($n = 21$, 30, and 19 cells; 7,781 DR and 4,117 PAPA trajectories; bootstrapped fractions bound: 27.2% ± 2.5% [DR], 26.1% ± 2.8% [PAPA]). (n.s.) $p > 0.05$, $^{****}p \leq 0.0001$. For statistical details, see methods. See also Figure S7.

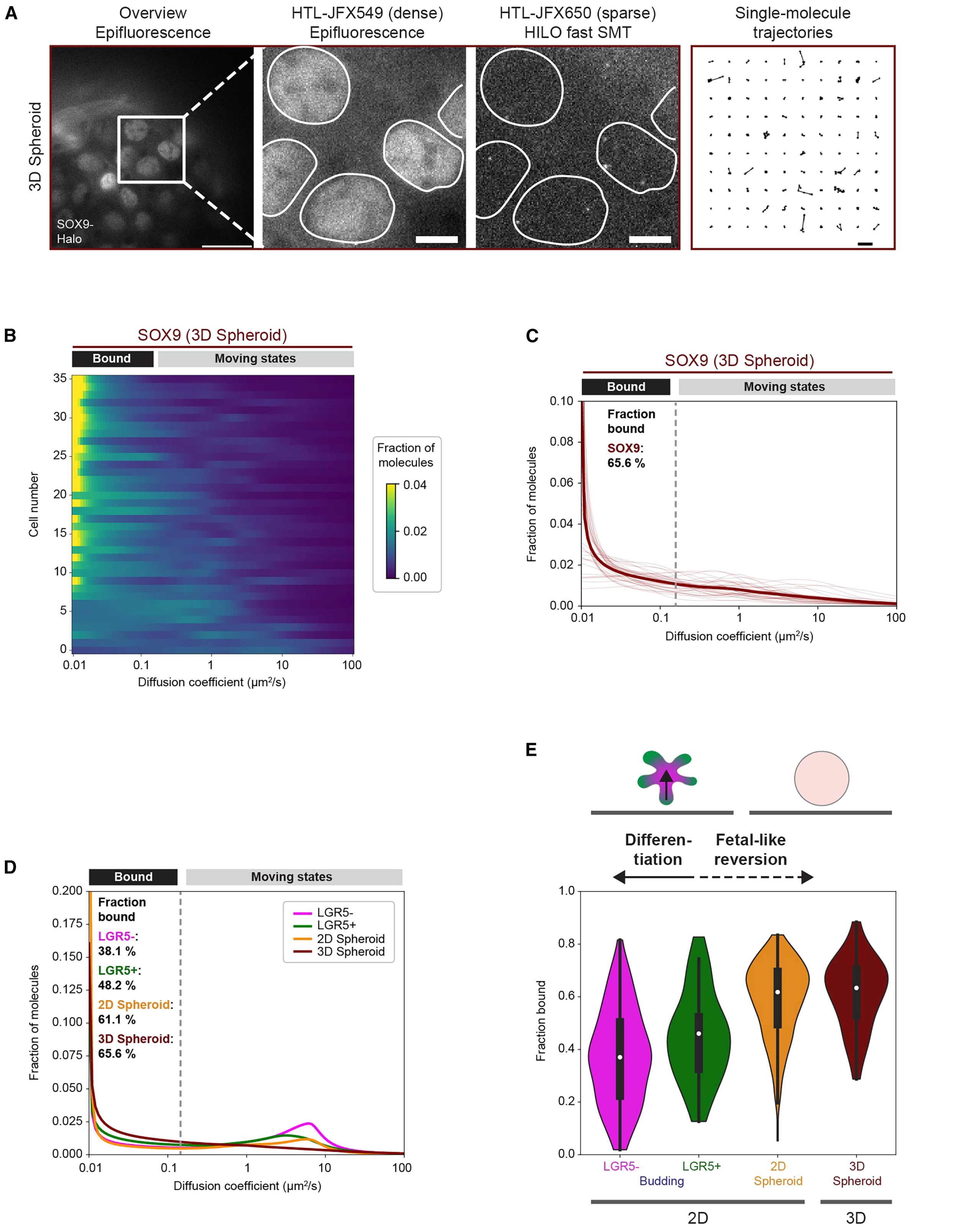

(legend on next page)

how the molecular dynamics and abundance of a cell fate-conferring TF change during differentiation. We further interrogated the robustness of cell fate determination to aberrant TF expression and how TF excess might alter TF molecular dynamics. Focusing on SOX9, our results show that different cellular states in adult intestinal organoid models are characterized by distinct SOX9-Halo diffusion dynamics (Figure 7D and 7E). Under homeostatic conditions, the fraction of DNA-bound SOX9-Halo molecules decreases during differentiation from ~48% in $LGR5^+$ stem/early progenitor cells to ~38% in $LGR5^-$ late progenitor/differentiated cells (Figures 2B, 7D, and 7E), consistent with SOX9 functioning in ISC-containing crypts (Blache et al., 2004; Mori-Akiyama et al., 2007). In contrast, long-term SOX9 overexpression eventually results in an increased fraction bound of SOX9-Halo molecules (>60%) (Figures 5C and 7C–7E), underlying a proliferative cell state transition (Figures 3B and 3E) and a morphological transition from budding to spherical organoids (Figures 3A and 4A). Spheroids lack canonical epithelial cell type markers across lineages (Figures 4D, 4E, and S5A) and acquire a gene expression program resembling fetal-like reversion (Figures 4D, 4E, and S5B).

The extension of our previously developed automated SMT pipeline (Walther et al., 2024) to the recording of fluorescent cell type markers (LGR5) allowed us to measure TF diffusion dynamics in a cell-type-resolved manner (Figure 2A), validating our previously established morphology-based broad distinction between ISCs/early progenitors and late progenitors/differentiated cells (Walther et al., 2024) (Figures 1H and 1I). Parsing $LGR5^{+/-}$ cells revealed differences in the diffusive behavior of SOX9 that correlated with differentiation (Figures 2 and S2A–S2D), while a subpopulation of small $LGR5^-$ cells displaying SOX9 diffusion characteristics of $LGR5^+$ cells (Figure 2C) potentially constitutes differentiated Paneth cells residing in proliferative centers, testable with additional cell type markers. Similar to our previous study on HES1 (Walther et al., 2024) and work in other differentiation contexts (Esbin et al., 2024; Kuchler et al., 2022), SOX9 is characterized by a larger fraction of immobile and hence DNA-bound molecules in the $LGR5^+$ stem/early progenitor cell subpopulation (Figures 2B, 2D, and S2A–S2D) in which it has been described to function (Blache et al., 2004; Mori-Akiyama et al., 2007). Consistent with HES1 (Walther et al., 2024), also functioning during early differentiation, the diffusive behavior of SOX9 is characterized by a rather small average fraction bound of ~39%, which could be explained by ISCs/early progenitor cells being broadly permissive epigenetic cell states that enable fast transitions to differentiated states (Jadhav et al., 2017; Kim et al., 2014). In addition, for both HES1 (Walther et al., 2024) and SOX9, the overall cell population (Figures 1E and S1E) and the proliferative/$LGR5^+$ population (Figures 2C, S2A, and S2C) show a highly heterogenous TF diffusive behavior, suggesting the existence of multiple cell states, consistent with single-cell RNA-seq data (Zwick et al., 2024), and possibly distinct differentiation trajectories for each $LGR5^+$ cell. Reasons for the latter could include differences in the cellular microenvironment (neighboring cell types), the progression along the same differentiation trajectory (distance from the proliferative center), or different lineages a progenitor cell has committed to. In future work, these could be probed using additional live-cell-compatible fluorescent cell type markers in combination with correlative IF for determining endpoint cell types and by tracking the same cell during differentiation with time-lapse SMT (Walther et al., 2024).

While the differences in SOX9 diffusive behavior were concentration-independent within the probed range of SOX9-Halo transgene expression levels (Figures S1G and 2D), long-term overexpression of SOX9 in mSIOs resulted in increased chromatin binding accompanied by a change in organoid morphology toward spherical (Figures 3A and 4A). In line with a recent report on SOX9 dosage robustness in the craniofacial context (Naqvi et al., 2023), these observations suggest that the intestinal epithelium can buffer

Figure 7. SMT in 3D spheroids confirms an increased fraction of DNA-bound SOX9-Halo molecules upon fetal-like reversion

(A) Manual SMT pipeline for SOX9-Halo in 3D spheroids. Double labeling of SOX9-Halo (gray) with two different HTLs allows bulk labeling (images 1, 2) for nuclear segmentation (images 2, 3; white masks) and sparse labeling for HILO-based fast SMT (image 3; one representative frame of an SMT movie) resulting in single-molecule trajectories (image 4; 100 randomly selected single-molecule trajectories). Scale bars: 20 μm (overview), 5 μm (zoom-ins), 1 μm (trajectories).

(B) Single-cell diffusion heatmap for 2 combined manual SMT experiments for SOX9-Halo in 3D spheroids (combined $n = 36$ cells with $n = 26$ and 10 cells per experiment). Cells are ordered by decreasing fraction bound from top to bottom. Representative SMT movie in Video S7.

(C) Single-cell diffusion spectra for data in (B). Bootstrap analysis of combined experiments with $n = 26$ and 10 cells determined a mean fraction bound of 65.6% (95% CI: 62.5%–68.8%).

(D and E) Summary of the diffusive behavior of SOX9-Halo determined by fast SMT under homeostatic differentiation conditions (budding) and upon SOX9 overexpression-induced fetal-like reversion (spheroid). (D) Mean diffusion spectra for the SOX9-Halo_budding $LGR5^{+/-}$ (green/magenta) subpopulations and for SOX9-Halo_spheroid determined in 3D spheroids (brown) or 2D EMCs (orange) with fractions bound indicated. (E) Violin plots for fractions bound (white point: median; whiskers: first/third quartile) of samples in (D).

modestly increased SOX9 levels (Figure 3C) for a limited time to maintain homeostasis (Figures 4A, S4D, and S4E). However, long-term SOX9 overexpression appears to break such robustness (Figures 3A, 3F, 3G, and 4). While we cannot completely rule out contributions to spheroid formation due to long-term organoid culture, our WT CTRL retaining budding morphology suggests that SOX9 overexpression at least accelerated spheroid formation if it was not the sole cause.

In addition to SOX9 dosage, future research will be required to unravel how other layers of control, such as posttranslational modifications, the availability of tissue-specific co-factors, and DNA methylation, and thus chromatin accessibility to gene-regulatory DNA motifs, come into play for SOX9 to achieve its various tissue- and context-dependent functions (Ming et al., 2022). In another differentiation system, SOX9 has been shown to act both via its capability to access closed chromatin and by competing for epigenetic factors to switch stem cell fates (Yang et al., 2023). Related to the SOX9 overexpression-induced spheroids described here, both in the fetal epithelium and upon fetal-like reversion in the adult intestinal epithelium, an increased accessibility was reported for genomic regions enriched for TF-binding sites of the SOX and TEAD families (Chen et al., 2023; Pikkupeura et al., 2023), both shown to form complexes with YAP/TAZ (Zanconato et al., 2015). In the future, it will be interesting to employ PAPA-SMT (Graham et al., 2022; 2025) (Figures 6A and 6B) to detect the interactions and study the molecular dynamics of such complexes in differentiation and reprogramming contexts. While our PAPA experiments suggest that SOX9 binds to DNA in an oligomerized form (Figures 6E, 6G, and S7A), possibly dimers as reported in other contexts (Bernard et al., 2003; Coustry et al., 2010), this assay cannot determine the degree of SOX9 oligomerization, including putative nuances in SOX9 oligomerization between undifferentiated and differentiated cells (Figures S7E and S7G), and whether such immobile self-associated SOX9 moieties are all bound at *cis*-regulatory elements of SOX9 target genes. Thus, another possible yet purely speculative scenario could be the formation of gene regulatory SOX9 hubs via weak multivalent interactions mediated by intrinsically disordered regions (IDRs) (Chong and Mir, 2021), testable with IDR mutants of SOX9 in future work. Beyond a potential link between the SOX9 expression level and its oligomerization, it remains to be tested whether an increased SOX9 dosage resulting in more immobile SOX9 molecules additionally extends the canonical DNA sites occupied by SOX9 in a "spill-over" effect, promoting fetal-like reprogramming.

Constitutive SOX9 expression is a common pathway to cancers through the activation of oncogenic transcription regulators (Grimm et al., 2020; Kumar and Mistri, 2020), consistent with the gain in proliferative capacity in the SOX9 overexpression-induced spheroids (Figures 3B and 3E). However, these spheroids were characterized by a loss in epithelial identity and function toward the activation of a gene expression program resembling that of regenerative fetal-like reversion upon tissue damage (Viragova et al., 2024) (Figures 3G, 4C–4E, and S5), consistent with SOX9's essential role in several developmental pathways (Ming et al., 2022). A similar gene expression signature is characteristic of fetal intestinal epithelial cultures in comparison to enteroids generated from adult intestinal cells (Fordham et al., 2013; Mustata et al., 2013). Hyperproliferative cells and fetal-like gene expression signatures were further found in crypts associated with granulomas resulting from helminth infection (Nusse et al., 2018) and enriched in the colonic epithelium upon damage induced by dextran sodium sulfate (Yui et al., 2018). Similar fetal-like transcriptional programs have been reported upon other injuries to ISCs or the intestinal crypt, such as diphtheria toxin-mediated ISC ablation, ionizing radiation, and chemotherapy (Ayyaz et al., 2019; Iqbal et al., 2025; Malagola et al., 2024; Mustata et al., 2013; Singh et al., 2022). Consistent with the observation of YAP1 activation in SOX9 spheroids (Figure 4E), such a regenerative response of fetal-like reversion is typically mediated by YAP1, which has a well-established role in driving pattern formation during intestinal regeneration (Gregorieff et al., 2015; Serra et al., 2019). While during an intestinal tissue damage response this transient fetal-like state is exited and intestinal homeostasis is reestablished, it is unclear whether this can be achieved in the SOX9 overexpression-induced spheroid context. To this end, a downregulation of YAP activity together with an upregulation of Wnt and Notch signals to sustain homeostatic ISCs might be required (Tian et al., 2015), while retinoid X receptor signaling could also be involved (Lukonin et al., 2020).

The activation of a YAP1-SOX9 circuit is both necessary and sufficient to induce a regenerative fetal-like reversion (Viragova et al., 2024). However, it has not been reported that SOX9 overexpression alone, whether directly or through intermediate steps, can act as a driver of this circuit. Nevertheless, a recent study suggests that SOX9 can promote the nuclear translocation and hence activation of YAP through direct interaction (Qian et al., 2024). Further studies will be required to elucidate whether SOX9 overexpression in the intestinal epithelial context directly results in YAP activation and fetal-like reprogramming or whether this involves a more complex cascade of molecular events, including triggering of YAP activation through changes in cell-cell contacts or physical properties of the extracellular matrix (Aragona et al., 2013; Dupont et al., 2011). In this respect, the long timescale of several months required for the acquisition of the described fetal-like reprogrammed state will be advantageous for resolving

intermediate steps during this organoid morphology transition in multi-pronged, scale-bridging time-course experiments. In addition, it will be interesting to investigate how the epithelial response across scales depends on varying initial SOX9 levels. Furthermore, a reduction in SOX9 expression at various time points during spheroid morphology acquisition will address a potential reversibility of this process. Altogether, while keeping the need of strict control mechanisms to avoid tumor initiation upon constitutive SOX9 expression in mind (Grimm et al., 2020; Kumar and Mistri, 2020), such studies will inform about the potential of the described SOX9 overexpression-induced fetal-like reversion to be exploited for applications in regenerative medicine.

Limitations of the study

Here, we used SOX9-Halo fusions (Figure 1B), which were key for live imaging experiments including SMT (Figures 1C and 1D). By determining a decreased fraction bound for a SOX9 mutant lacking its DNA-binding domain (Figures 1E–1I), we confirmed functionality of SOX9-Halo for DNA binding. Furthermore, we could reproduce a SOX9 overexpression-induced spheroid phenotype with both Halo and mEGFP fusions (Figure 4A). Moreover, we modestly overexpressed transgene-encoded SOX9-Halo in addition to unlabeled endogenous SOX9 (Figure 3C), necessitating the consideration of the presence of a mixed SOX9 population for data interpretation, which might have resulted in underestimated fractions bound (Figures 1F, 1G, 2B, 5C, 7C–7E, S1A, S1E, S1F, S1I, S2A, S2B, S2H, S2I, and S6A) and oligomerized states (Figures 6E–6H and S7). Future studies could be improved by endogenous tagging (Koch et al., 2018) to investigate SOX9 molecular dynamics at physiological expression levels and to manipulate endogenous levels for interrogating TF dosage effects.

We found a large heterogeneity in the cellular diffusion behavior of SOX9-Halo (Figures 1E, 1G, 1H, 1I, 5D, S1E, S1G, S6A, and S6B), even within cell state-enriched subpopulations (Figures 2B–2D and 5D). Notably, we also observed some cell-to-cell heterogeneity in the bound fractions of the SOX9ΔHMG-Halo DNA-binding mutant (Figures 1E, 1H and 1I) as well as in the H2B-Halo and Halo-NLS CTRLs (Figure S8A). Potential sources for the observed cell-to-cell variability in our CTRLs are extracellular microenvironment, cell-intrinsic differences in the cell cycle or metabolic state, and cell fitness, with a few likely abnormal or dying cells not identified as such in manual QC captured across experiments (Figure S8B). By comparing LGR5^{+} vs. LGR5^{-} subpopulations (Figures S2H, S2I, and S8C) for these CTRLs, we ruled out cellular differentiation state as a source of variability in fractions bound. In contrast, the large cell-to-cell variability within LGR5^{+} (stem and early progenitor cells) and LGR5^{-} (late progenitor and differentiated cells) subpopulations for SOX9-Halo (Figures 2B–2D, S2A–S2C, and S8A) is largely of biological nature, as they contain several differentiation states. Nevertheless, despite the observed cell-to-cell heterogeneities, pairwise comparisons of the fractions bound between the various proteins measured in this study were all significant (Figure S8C).

3D mSIOs and 2D EMCs are well-characterized differentiation systems recapitulating important features of the intestinal epithelium (Figure 1A). Nevertheless, they are simplistic *in vitro* models, and therefore, further studies are required to test the validity of our results in the *in vivo* context and their clinical applicability. SMT deep in tissues remains challenging, and different microscopy techniques capable of imaging single molecules inside thicker specimens (Chen et al., 2014) are required for SMT in whole 3D mSIOs. However, our optimization of seeding and imaging conditions enabling HILO-based fast SMT in 3D spheroids (Figures 7A–7C) is an important step toward a broader use in the outer layer of organoid models for connecting multicellular phenotypes with molecular mechanisms.

METHODS

Experimental model and subject participant details

Mice

C57BL/6J mice (WT) were obtained from The Jackson Laboratory (strain #000664, RRID:IMSR_JAX:000664). LGR5::DTR-EGFP mice (Tian et al., 2011) were kindly provided by Fred de Sauvage (Genentech). WT and LGR5::DTR-EGFP mice were bred with C57BL/6J mice and housed in an AAALAS-certified level 3 facility on a 14 h light cycle. Pups were weaned 21 days after birth and housed with four littermates per cage. Female offspring was used for mouse small intestinal crypt isolation and organoid generation at an age of 8–16 weeks. All procedures to maintain and use the mice were approved by the Institutional Animal Care and Use Committee of the University of California, Berkeley (IACUC protocol number AUP-2015-09-7988-2).

Cell lines

L-Wnt3a cells (CRL-2647, ATCC) were used to produce Wnt3a-conditioned medium. HEK293T cells (CRL-3216, ATCC) were used to generate rAAV vectors for crude rAAV vector preparations, and HEK293T Lenti-X cells (Takara, cat.# 632180) for making lentivirus. Cell lines were obtained via the UC Berkeley Cell Culture Facility. Cell lines and organoids were confirmed to be mycoplasma-free by regular PCR testing.

Experimental procedures

Detailed methods can be found in the supplemental experimental procedures. Key methods are described in brief in the following text.

Culture of mouse small intestinal organoids and 2D enteroid monolayers

Intestinal organoids were derived from mouse small intestinal crypts and cultured as described before (Walther et al., 2024). 2D EMCs were derived from mSIOs and cultured as described before (Walther et al., 2024). For details, see supplemental experimental procedures.

Generation of stable organoid lines

Stable organoid lines were generated by lentivirus transduction and antibiotic selection as described before (Walther et al., 2024). For details, see supplemental experimental procedures.

Transient transduction of 2D enteroid monolayers using crude rAAV preparations

2D EMCs were transiently transduced using crude rAAV preparations, which were generated as previously described (Benyamini et al., 2023). For details, see supplemental experimental procedures.

Immunostaining

IF experiments on 2D EMCs were performed as described (Walther et al., 2024). For details, see supplemental experimental procedures.

Confocal imaging

Confocal imaging of mSIOs and 2D EMCs was performed as previously described (Walther et al., 2024). For details and modifications, see supplemental experimental procedures.

Single-molecule imaging and automation

Fast SMT and PAPA-SMT experiments were performed on a TIRF microscope and analyzed as described before (Walther et al., 2024), whereby most experiments were executed using the automated pipeline for SMT and analysis described before (Walther et al., 2024) with the add-on to record additional fluorescence markers. For details and modifications, see supplemental experimental procedures.

Bulk RNA-seq

RNA for bulk RNA-seq was extracted from intestinal organoids using TRIzol and phenol-chloroform extraction, followed by poly-A RNA-seq library preparation. RNA-seq data were analyzed using common bioinformatics tools. For details, see supplemental experimental procedures.

Statistical testing

Quantifications and comparisons between conditions were statistically tested as described in the supplemental experimental procedures.

RESOURCE AVAILABILITY

Lead contact

Further information and requests for resources and reagents should be directed to and will be fulfilled by the lead contact and corresponding author, Nike Walther (nikewalther.science@gmail.com).

Materials availability

Plasmids generated in this study are available upon request with a completed material transfer agreement and with reasonable compensation by the requestor for shipping. There are restrictions to the availability of stable organoid lines generated in this study due to the lack of an external centralized repository for their distribution and our need to maintain the stock.

Data and code availability

- Microscopy data have been deposited to Mendeley Data and will be publicly available upon publication via the links indicated here: https://gitlab.com/nikewalther/walther_sox9organoid_2025/-/tree/main/DataDeposit.
- RNA-seq data have been deposited to the NCBI Gene Expression Omnibus (GEO) under accession number GSE287653 and will be publicly available upon publication.
- Original code has been deposited to GitLab (https://gitlab.com/nikewalther/walther_sox9organoid_2025/-/tree/main?ref_type=heads) and will be publicly available upon publication.
- Information required to reanalyze the data has been included.

ACKNOWLEDGMENTS

We are grateful to Robert Tjian and Xavier Darzacq for hosting the experimental part of this study in their joint lab at the University of California, Berkeley, and for providing funding (Robert Tjian via the Howard Hughes Medical Institute [34430] and Xavier Darzacq via a Dynamic Imaging Grant from the Chan Zuckerberg Initiative [Dynamic-0000000091]). We thank current and past members of the Tjian/Darzacq lab for scientific discussions and comments on the manuscript. We are grateful to Qiulin Zhu and Brendan Wu for cloning assistance, Sanchitha Kannabran and Sophia Lim for assistance with plasmid preparations and organoid cultures, Xinyin Lu for plasmid preparations, and Shuang Zheng for assistance with mouse colony maintenance. We thank Thomas Graham for providing code for (PAPA)-SMT automation and analysis. We are thankful to Fred de Sauvage for providing LGR5::DTR-EGFP mice, Mark Kay for KP1 capsid plasmids, and Luke Lavis for JF dyes. We thank the UC Berkeley Cell Culture Facility supported by The University of California, Berkeley, for providing cell lines. We are grateful to Ophir Klein and current and past members of the Klein lab gut group for sharing protocols and providing a platform for discussion and feedback. We would like to thank Fred de Sauvage and Kim Boonekamp for critically reading and commenting on the manuscript. N.W. extends her gratitude to Olaf Stemmann and the Department of Genetics at the University of Bayreuth for hosting her for parts of this study. N.W. acknowledges funding from the Berkeley Stem Cell Center via a Siebel postdoctoral fellowship as well as from the German Research Foundation (DFG) via a Walter Benjamin postdoctoral fellowship (453309976) and a return grant of the Walter Benjamin program (552461306). S.A. was supported by a Donner 160 fellowship. A.C.M. acknowledges support via the California Institute for Regenerative Medicine Training Program (EDUC4-12790).

AUTHOR CONTRIBUTIONS

N.W. conceptualized, designed, and supervised the study, developed experimental strategies for rAAV-based transgene delivery into 2D EMCs as well as for SMT in 3D spheroids, devised the further development of the automated SMT imaging and analysis pipeline, manually segmented confocal images, analyzed imaging and RNA-seq data, and wrote the manuscript (original draft and review/editing). N.W. executed all experiments except for RNA extraction and library preparation for RNA-seq, which was performed by C.C., and confocal imaging of one IF condition, which was performed by S.A. S.A. further developed the automated SMT pipeline to accommodate the acquisition of additional fluorescent markers in 2D EMCs and to enable the separate analysis of manually classified SMT data with input from N.W., wrote tailored SMT and image analysis code including statistical analyses, and analyzed a subset of confocal data. C.C. processed RNA-seq data and performed initial analyses. S.A. and C.C. discussed data and their interpretation with N.W., provided a subset of raw figure panels as well as method details, and reviewed and edited the manuscript. G.M.D. designed and cloned DNA constructs. A.C.M. provided critical reagents and advice for rAAV-based transgene delivery. N.W. provided funding for this project. All authors agreed on the final version of the manuscript.

DECLARATION OF INTERESTS

The authors declare no competing interests.

SUPPLEMENTAL INFORMATION

Supplemental information can be found online at https://doi.org/10.1016/j.stemcr.2025.102787.

Received: June 29, 2025
Revised: December 19, 2025
Accepted: December 23, 2025
Published: January 22, 2026

REFERENCES

Abdel-Samad, R., Zalzali, H., Rammah, C., Giraud, J., Naudin, C., Dupasquier, S., Poulat, F., Boizet-Bonhoure, B., Lumbroso, S., Mouzat, K., et al. (2011). MiniSOX9, a dominant-negative variant in colon cancer cells. Oncogene *30*, 2493–2503. https://doi.org/10.1038/onc.2010.621.

Altay, G., Larrañaga, E., Tosi, S., Barriga, F.M., Batlle, E., Fernández-Majada, V., and Martínez, E. (2019). Self-organized intestinal epithelial monolayers in crypt and villus-like domains show effective barrier function. Sci. Rep. *9*, 1–14. https://doi.org/10.1038/s41598-019-46497-x.

Aragona, M., Panciera, T., Manfrin, A., Giulitti, S., Michielin, F., Elvassore, N., Dupont, S., and Piccolo, S. (2013). A mechanical checkpoint controls multicellular growth through YAP/TEAD regulation by actin-processing factors. Cell *154*, 1047–1059. https://doi.org/10.1016/j.cell.2013.07.042.

Ayyaz, A., Kumar, S., Sangiorgi, B., Ghoshal, B., Gosio, J., Ouladan, S., Fink, M., Barutcu, S., Trcka, D., Shen, J., et al. (2019). Single-cell transcriptomes of the regenerating intestine reveal a revival stem cell. Nature *569*, 121–125. https://doi.org/10.1038/s41586-019-1154-y.

Barker, N., Van Es, J.H., Kuipers, J., Kujala, P., Van Den Born, M., Cozijnsen, M., Haegebarth, A., Korving, J., Begthel, H., Peters, P.J., and Clevers, H. (2007). Identification of stem cells in small intestine and colon by marker gene Lgr5. Nature *449*, 1003–1007. https://doi.org/10.1038/nature06196.

Barrionuevo, F., Bagheri-Fam, S., Klattig, J., Kist, R., Taketo, M.M., Englert, C., and Scherer, G. (2006). Homozygous inactivation of Sox9 causes complete XY sex reversal in mice. Biol. Reprod. *74*, 195–201. https://doi.org/10.1095/biolreprod.105.045930.

Bastide, P., Darido, C., Pannequin, J., Kist, R., Robine, S., Marty-Double, C., Bibeau, F., Scherer, G., Joubert, D., Hollande, F., et al. (2007). Sox9 regulates cell proliferation and is required for Paneth cell differentiation in the intestinal epithelium. J. Cell Biol. *178*, 635–648. https://doi.org/10.1083/jcb.200704152.

Benyamini, B., Esbin, M.N., Whitney, O., Walther, N., and Maurer, A.C. (2023). Transgene Expression in Cultured Cells Using Unpurified Recombinant Adeno-Associated Viral Vectors. J. Vis. Exp. *2023*, 1–22. https://doi.org/10.3791/65572.

Bernard, P., Tang, P., Liu, S., Dewing, P., Harley, V.R., and Vilain, E. (2003). Dimerization of SOX9 is required for chondrogenesis, but not for sex determination. Hum. Mol. Genet. *12*, 1755–1765. https://doi.org/10.1093/hmg/ddg182.

Beumer, J., and Clevers, H. (2016). Regulation and plasticity of intestinal stem cells during homeostasis and regeneration. Dev *143*, 3639–3649. https://doi.org/10.1242/dev.133132.

Beumer, J., and Clevers, H. (2021). Cell fate specification and differentiation in the adult mammalian intestine. Nat. Rev. Mol. Cell Biol. *22*, 39–53. https://doi.org/10.1038/s41580-020-0278-0.

Bi, W., Deng, J.M., Zhang, Z., Behringer, R.R., and De Crombrugghe, B. (1999). Sox9 is required for cartilage formation. Nat. Genet. *22*, 85–89. https://doi.org/10.1038/8792.

Blache, P., Van De Wetering, M., Duluc, I., Domon, C., Berta, P., Freund, J.N., Clevers, H., and Jay, P. (2004). SOX9 is an intestine crypt transcription factor, is regulated by the Wnt pathway, and represses the CDX2 and MUC2 genes. J. Cell Biol. *166*, 37–47. https://doi.org/10.1083/jcb.200311021.

Boka, A.P., Mukherjee, A., and Mir, M. (2021). Single-molecule tracking technologies for quantifying the dynamics of gene regulation in cells, tissue and embryos. Dev *148*, dev199744. https://doi.org/10.1242/DEV.199744.

Chen, B.-C., Legant, W.R., Wang, K., Shao, L., Milkie, D.E., Davidson, M.W., Janetopoulos, C., Wu, X.S., Hammer, J.A., Liu, Z., et al. (2014). Lattice light-sheet microscopy: Imaging molecules to embryos at high spatiotemporal resolution. Science *346*, 1257998. https://doi.org/10.1126/science.1257998.

Chen, L., Qiu, X., Dupre, A., Pellon-Cardenas, O., Fan, X., Xu, X., Rout, P., Walton, K.D., Burclaff, J., Zhang, R., et al. (2023). TGFB1 induces fetal reprogramming and enhances intestinal regeneration. Cell Stem Cell *30*, 1520–1537.e8. https://doi.org/10.1016/j.stem.2023.09.015.

Chen, Y., Cattoglio, C., Dailey, G.M., Zhu, Q., Tjian, R., and Darzacq, X. (2022). Mechanisms governing target search and binding

dynamics of hypoxia-inducible factors. eLife *11*, e75064. https://doi.org/10.7554/eLife.75064.

Chong, S., and Mir, M. (2021). Towards Decoding the Sequence-Based Grammar Governing the Functions of Intrinsically Disordered Protein Regions. J. Mol. Biol. *433*, 166724. https://doi.org/10.1016/j.jmb.2020.11.023.

Coustry, F., Oh, C.d., Hattori, T., Maity, S.N., de Crombrugghe, B., and Yasuda, H. (2010). The dimerization domain of SOX9 is required for transcription activation of a chondrocyte-specific chromatin DNA template. Nucleic Acids Res. *38*, 6018–6028. https://doi.org/10.1093/nar/gkq417.

Cramer, P. (2019). Organization and regulation of gene transcription. Nature *573*, 45–54. https://doi.org/10.1038/s41586-019-1517-4.

Dahal, L., Walther, N., Tjian, R., Darzacq, X., and Graham, T.G.W. (2023). Single-molecule tracking (SMT): A window into live-cell transcription biochemistry. Biochem. Soc. Trans. *51*, 557–569. https://doi.org/10.1042/BST20221242.

Dahal, L., Graham, T.G.W., Dailey, G.M., Heckert, A., Tjian, R., and Darzacq, X. (2025). Surprising features of nuclear receptor interaction networks revealed by live-cell single-molecule imaging. eLife *12*, RP92979. https://doi.org/10.7554/eLife.92979.

Date, S., and Sato, T. (2015). Mini-Gut Organoids: Reconstitution of the Stem Cell Niche. Annu. Rev. Cell Dev. Biol. *31*, 269–289. https://doi.org/10.1146/annurev-cellbio-100814-125218.

Dupont, S., Morsut, L., Aragona, M., Enzo, E., Giulitti, S., Cordenonsi, M., Zanconato, F., Le Digabel, J., Forcato, M., Bicciato, S., et al. (2011). Role of YAP/TAZ in mechanotransduction. Nature *474*, 179–183. https://doi.org/10.1038/nature10137.

Esbin, M.N., Dahal, L., Fan, V.B., McKenna, J., Yin, E., Darzacq, X., and Tjian, R. (2024). TFEB controls expression of human syncytins during cell–cell fusion. Genes Dev. *38*, 718–737. https://doi.org/10.1101/gad.351633.124.

Ferrie, J.J., Karr, J.P., Graham, T.G.W., Dailey, G.M., Zhang, G., Tjian, R., and Darzacq, X. (2024). P300 Is an Obligate Integrator of Combinatorial Transcription Factor Inputs. Mol. Cell *84*, 234–243.e4. https://doi.org/10.1016/j.molcel.2023.12.004.

Fordham, R.P., Yui, S., Hannan, N.R.F., Soendergaard, C., Madgwick, A., Schweiger, P.J., Nielsen, O.H., Vallier, L., Pedersen, R.A., Nakamura, T., et al. (2013). Transplantation of expanded fetal intestinal progenitors contributes to colon regeneration after injury. Cell Stem Cell *13*, 734–744. https://doi.org/10.1016/j.stem.2013.09.015.

Formeister, E.J., Sionas, A.L., Lorance, D.K., Barkley, C.L., Lee, G.H., and Magness, S.T. (2009). Distinct SOX9 levels differentially mark stem/progenitor populations and enteroendocrine cells of the small intestine epithelium. Am. J. Physiol. Gastrointest. Liver Physiol. *296*, 1108–1118. https://doi.org/10.1152/ajpgi.00004.2009.

Gehart, H., and Clevers, H. (2019). Tales from the crypt: new insights into intestinal stem cells. Nat. Rev. Gastroenterol. Hepatol. *16*, 19–34. https://doi.org/10.1038/s41575-018-0081-y.

Graham, T.G.W., Ferrie, J.J., Dailey, G.M., Tjian, R., and Darzacq, X. (2022). Detecting molecular interactions in live-cell single-molecule imaging with proximity-assisted photoactivation (PAPA). eLife *11*, e76870. https://doi.org/10.7554/eLife.76870.

Graham, T.G.W., Dugast-Darzacq, C., Dailey, G.M., Weng, B., Anantakrishnan, S., Darzacq, X., and Tjian, R. (2025). Single-molecule live imaging of subunit interactions and exchange within cellular regulatory complexes. Mol. Cell *85*, 2854–2868.e7. https://doi.org/10.1016/j.molcel.2025.06.028.

Gregorieff, A., Liu, Y., Inanlou, M.R., Khomchuk, Y., and Wrana, J.L. (2015). Yap-dependent reprogramming of Lgr5+ stem cells drives intestinal regeneration and cancer. Nature *526*, 715–718. https://doi.org/10.1038/nature15382.

Grimm, D., Bauer, J., Wise, P., Krüger, M., Simonsen, U., Wehland, M., Infanger, M., and Corydon, T.J. (2020). The role of SOX family members in solid tumours and metastasis. Semin. Cancer Biol. *67*, 122–153. https://doi.org/10.1016/j.semcancer.2019.03.004.

Grimm, J.B., Muthusamy, A.K., Liang, Y., Brown, T.A., Lemon, W.C., Patel, R., Lu, R., Macklin, J.J., Keller, P.J., Ji, N., and Lavis, L.D. (2017). A general method to fine-tune fluorophores for live-cell and in vivo imaging. Nat. Methods *14*, 987–994. https://doi.org/10.1038/nmeth.4403.

Hansen, A.S., Pustova, I., Cattoglio, C., Tjian, R., and Darzacq, X. (2017). CTCF and cohesin regulate chromatin loop stability with distinct dynamics. eLife *6*, e25776. https://doi.org/10.7554/eLife.25776.

Hsieh, T.H.S., Cattoglio, C., Slobodyanyuk, E., Hansen, A.S., Darzacq, X., and Tjian, R. (2022). Enhancer–promoter interactions and transcription are largely maintained upon acute loss of CTCF, cohesin, WAPL or YY1. Nat. Genet. *54*, 1919–1932. https://doi.org/10.1038/s41588-022-01223-8.

Huilgol, D., Venkataramani, P., Nandi, S., and Bhattacharjee, S. (2019). Transcription factors that govern development and disease: An achilles heel in cancer. Genes *10*, 794. https://doi.org/10.3390/genes10100794.

Iqbal, S., Andersson, S., Nesta, E., Pentinmikko, N., Kumar, A., Kumar Jha, S., Borshagovski, D., Webb, A., Gebert, N., Viitala, E.W., et al. (2025). Fetal-like reversion in the regenerating intestine is regulated by mesenchymal asporin. Cell Stem Cell *32*, 613–626.e8. https://doi.org/10.1016/j.stem.2025.02.009.

Jadhav, U., Saxena, M., O’Neill, N.K., Saadatpour, A., Yuan, G.C., Herbert, Z., Murata, K., and Shivdasani, R.A. (2017). Dynamic Reorganization of Chromatin Accessibility Signatures during Dedifferentiation of Secretory Precursors into Lgr5+ Intestinal Stem Cells. Cell Stem Cell *21*, 65–77.e5. https://doi.org/10.1016/j.stem.2017.05.001.

Kim, T.H., Li, F., Ferreiro-Neira, I., Ho, L.L., Luyten, A., Nalapareddy, K., Long, H., Verzi, M., and Shivdasani, R.A. (2014). Broadly permissive intestinal chromatin underlies lateral inhibition and cell plasticity. Nature *506*, 511–515. https://doi.org/10.1038/nature12903.

Koch, B., Nijmeijer, B., Kueblbeck, M., Cai, Y., Walther, N., and Ellenberg, J. (2018). Generation and validation of homozygous fluorescent knock-in cells using CRISPR-Cas9 genome editing. Nat. Protoc. *13*, 1465–1487. https://doi.org/10.1038/nprot.2018.042.

Kuchler, O., Gerlach, J., Vomhof, T., Hettich, J., Steinmetz, J., Gebhardt, J.C.M., Michaelis, J., and Knöll, B. (2022). Single-molecule

tracking (SMT) and localization of SRF and MRTF transcription factors during neuronal stimulation and differentiation. Open Biol. *12*, 210383. https://doi.org/10.1098/rsob.210383.

Kumar, P., and Mistri, T.K. (2020). Transcription factors in SOX family: Potent regulators for cancer initiation and development in the human body. Semin. Cancer Biol. *67*, 105–113. https://doi.org/10.1016/j.semcancer.2019.06.016.

Lee, T.I., and Young, R.A. (2013). Transcriptional regulation and its misregulation in disease. Cell *152*, 1237–1251. https://doi.org/10.1016/j.cell.2013.02.014.

Lukonin, I., Serra, D., Challet Meylan, L., Volkmann, K., Baaten, J., Zhao, R., Meeusen, S., Colman, K., Maurer, F., Stadler, M.B., et al. (2020). Phenotypic landscape of intestinal organoid regeneration. Nature *586*, 275–280. https://doi.org/10.1038/s41586-020-2776-9.

Malagola, E., Vasciaveo, A., Ochiai, Y., Kim, W., Zheng, B., Zanella, L., Wang, A.L.E., Middelhoff, M., Nienhüser, H., Deng, L., et al. (2024). Isthmus progenitor cells contribute to homeostatic cellular turnover and support regeneration following intestinal injury. Cell *187*, 3056–3071.e17. https://doi.org/10.1016/j.cell.2024.05.004.

Mazzocca, M., Fillot, T., Loffreda, A., Gnani, D., and Mazza, D. (2021). The needle and the haystack: Single molecule tracking to probe the transcription factor search in eukaryotes. Biochem. Soc. Trans. *49*, 1121–1132. https://doi.org/10.1042/BST20200709.

Mazzocca, M., Loffreda, A., Colombo, E., Fillot, T., Gnani, D., Falletta, P., Monteleone, E., Capozi, S., Bertrand, E., Legube, G., et al. (2023). Chromatin organization drives the search mechanism of nuclear factors. Nat. Commun. *14*, 6433. https://doi.org/10.1038/s41467-023-42133-5.

McKinley, K.L., Stuurman, N., Royer, L.A., Schartner, C., Castillo-Azofeifa, D., Delling, M., Klein, O.D., and Vale, R.D. (2018). Cellular aspect ratio and cell division mechanics underlie the patterning of cell progeny in diverse mammalian epithelia. eLife *7*, e36739. https://doi.org/10.7554/eLife.36739.

Mertin, S., McDowall, S.G., and Harley, V.R. (1999). The DNA-binding specificity of SOX9 and other SOX proteins. Nucleic Acids Res. *27*, 1359–1364. https://doi.org/10.1093/nar/27.5.1359.

Miller, I., Min, M., Yang, C., Tian, C., Gookin, S., Carter, D., and Spencer, S.L. (2018). Ki67 is a Graded Rather than a Binary Marker of Proliferation versus Quiescence. Cell Rep. *24*, 1105–1112.e5. https://doi.org/10.1016/j.celrep.2018.06.110.

Ming, Z., Vining, B., Bagheri-Fam, S., and Harley, V. (2022). SOX9 in organogenesis: shared and unique transcriptional functions. Cell. Mol. Life Sci. *79*, 522. https://doi.org/10.1007/s00018-022-04543-4.

Mori-Akiyama, Y., van den Born, M., van Es, J.H., Hamilton, S.R., Adams, H.P., Zhang, J., Clevers, H., and de Crombrugghe, B. (2007). SOX9 Is Required for the Differentiation of Paneth Cells in the Intestinal Epithelium. Gastroenterology *133*, 539–546. https://doi.org/10.1053/j.gastro.2007.05.020.

Mustata, R.C., Vasile, G., Fernandez-Vallone, V., Strollo, S., Lefort, A., Libert, F., Monteyne, D., Pérez-Morga, D., Vassart, G., and Garcia, M.I. (2013). Identification of Lgr5-Independent Spheroid-Generating Progenitors of the Mouse Fetal Intestinal Epithelium. Cell Rep. *5*, 421–432. https://doi.org/10.1016/j.celrep.2013.09.005.

Namoto, K., Baader, C., Orsini, V., Landshammer, A., Breuer, E., Dinh, K.T., Ungricht, R., Pikiolek, M., Laurent, S., Lu, B., et al. (2024). NIBR-LTSi is a selective LATS kinase inhibitor activating YAP signaling and expanding tissue stem cells in vitro and in vivo. Cell Stem Cell *31*, 554–569.e17. https://doi.org/10.1016/j.stem.2024.03.003.

Naqvi, S., Kim, S., Hoskens, H., Matthews, H.S., Spritz, R.A., Klein, O.D., Hallgrímsson, B., Swigut, T., Claes, P., Pritchard, J.K., and Wysocka, J. (2023). Precise modulation of transcription factor levels identifies features underlying dosage sensitivity. Nat. Genet. *55*, 841–851. https://doi.org/10.1038/s41588-023-01366-2.

Nusse, Y.M., Savage, A.K., Marangoni, P., Rosendahl-Huber, A.K.M., Landman, T.A., De Sauvage, F.J., Locksley, R.M., and Klein, O.D. (2018). Parasitic helminths induce fetal-like reversion in the intestinal stem cell niche. Nature *559*, 109–113. https://doi.org/10.1038/s41586-018-0257-1.

Pikkupeura, L.M., Bressan, R.B., Guiu, J., Chen, Y., Maimets, M., Mayer, D., Schweiger, P.J., Hansen, S.L., Maciag, G.J., Larsen, H.L., et al. (2023). Transcriptional and epigenomic profiling identifies YAP signaling as a key regulator of intestinal epithelium maturation. Sci. Adv. *9*, eadf9460. https://doi.org/10.1126/sciadv.adf9460.

Poché, R.A., Furuta, Y., Chaboissier, M.C., Schedl, A., and Behringer, R.R. (2008). Sox9 is expressed in mouse multipotent retinal progenitor cells and functions in Müller Glial cell development. J. Comp. Neurol. *510*, 237–250. https://doi.org/10.1002/cne.21746.

Prévostel, C., and Blache, P. (2017). The dose-dependent effect of SOX9 and its incidence in colorectal cancer. Eur. J. Cancer *86*, 150–157. https://doi.org/10.1016/j.ejca.2017.08.037.

Qian, H., Ding, C.H., Liu, F., Chen, S.J., Huang, C.K., Xiao, M.C., Hong, X.L., Wang, M.C., Yan, F.Z., Ding, K., et al. (2024). SRY-Box transcription factor 9 triggers YAP nuclear entry via direct interaction in tumors. Signal Transduct. Target. Ther. *9*, 96. https://doi.org/10.1038/s41392-024-01805-4.

Rockich, B.E., Hrycaj, S.M., Shih, H.P., Nagy, M.S., Ferguson, M.A.H., Kopp, J.L., Sander, M., Wellik, D.M., and Spence, J.R. (2013). Sox9 plays multiple roles in the lung epithelium during branching morphogenesis. Proc. Natl. Acad. Sci. USA *110*, E4456–E4464. https://doi.org/10.1073/pnas.1311847110.

Sanman, L.E., Chen, I.W., Bieber, J.M., Thorne, C.A., Wu, L.F., and Altschuler, S.J. (2020). Chapter 6. Methods Mol. Biol. *2171*, 99–113.

Sato, T., Vries, R.G., Snippert, H.J., Van De Wetering, M., Barker, N., Stange, D.E., Van Es, J.H., Abo, A., Kujala, P., Peters, P.J., and Clevers, H. (2009). Single Lgr5 stem cells build crypt-villus structures in vitro without a mesenchymal niche. Nature *459*, 262–265. https://doi.org/10.1038/nature07935.

Schöneberg, J., Dambournet, D., Liu, T.L., Forster, R., Hockemeyer, D., Betzig, E., and Drubin, D.G. (2018). 4D cell biology: big data image analytics and lattice light-sheet imaging reveal dynamics of clathrin-mediated endocytosis in stem cell–derived intestinal

organoids. Mol. Biol. Cell *29*, 2959–2968. https://doi.org/10.1091/mbc.E18-06-0375.

Serra, D., Mayr, U., Boni, A., Lukonin, I., Rempfler, M., Challet Meylan, L., Stadler, M.B., Strnad, P., Papasaikas, P., Vischi, D., et al. (2019). Self-organization and symmetry breaking in intestinal organoid development. Nature *569*, 66–72. https://doi.org/10.1038/s41586-019-1146-y.

Seymour, P.A., Freude, K.K., Tran, M.N., Mayes, E.E., Jensen, J., Kist, R., Scherer, G., and Sander, M. (2007). SOX9 is required for maintenance of the pancreatic progenitor cell pool. Proc. Natl. Acad. Sci. USA *104*, 1865–1870. https://doi.org/10.1073/pnas.0609217104.

Singh, P.N.P., Madha, S., Leiter, A.B., and Shivdasani, R.A. (2022). Cell and chromatin transitions in intestinal stem cell regeneration. Genes Dev. *36*, 684–698. https://doi.org/10.1101/gad.349412.122.

Szczurek, A.T., Dimitrova, E., Kelley, J.R., Blackledge, N.P., and Klose, R.J. (2024). The Polycomb system sustains promoters in a deep OFF state by limiting pre-initiation complex formation to counteract transcription. Nat. Cell Biol. *26*, 1700–1711. https://doi.org/10.1038/s41556-024-01493-w.

Tallapragada, N.P., Cambra, H.M., Wald, T., Keough Jalbert, S., Abraham, D.M., Klein, O.D., and Klein, A.M. (2021). Inflation-collapse dynamics drive patterning and morphogenesis in intestinal organoids. Cell Stem Cell *28*, 1516–1532.e14. https://doi.org/10.1016/j.stem.2021.04.002.

Thomsen, M.K., Butler, C.M., Shen, M.M., and Swain, A. (2008). Sox9 is required for prostate development. Dev. Biol. *316*, 302–311. https://doi.org/10.1016/j.ydbio.2008.01.030.

Thorne, C.A., Chen, I.W., Sanman, L.E., Cobb, M.H., Wu, L.F., and Altschuler, S.J. (2018). Enteroid Monolayers Reveal an Autonomous WNT and BMP Circuit Controlling Intestinal Epithelial Growth and Organization. Dev. Cell *44*, 624–633.e4. https://doi.org/10.1016/j.devcel.2018.01.024.

Tian, H., Biehs, B., Warming, S., Leong, K.G., Rangell, L., Klein, O.D., and De Sauvage, F.J. (2011). A reserve stem cell population in small intestine renders Lgr5-positive cells dispensable. Nature *478*, 255–259. https://doi.org/10.1038/nature10408.

Tian, H., Biehs, B., Chiu, C., Siebel, C.W., Wu, Y., Costa, M., De Sauvage, F.J., and Klein, O.D. (2015). Opposing activities of notch and wnt signaling regulate intestinal stem cells and gut homeostasis. Cell Rep. *11*, 33–42. https://doi.org/10.1016/j.celrep.2015.03.007.

van der Flier, L.G., Haegebarth, A., Stange, D.E., van de Wetering, M., and Clevers, H. (2009). OLFM4 Is a Robust Marker for Stem Cells in Human Intestine and Marks a Subset of Colorectal Cancer Cells. Gastroenterology *137*, 15–17. https://doi.org/10.1053/j.gastro.2009.05.035.

Vidal, V.P.I., Chaboissier, M.C., Lützkendorf, S., Cotsarelis, G., Mill, P., Hui, C.C., Ortonne, N., Ortonne, J.P., and Schedl, A. (2005). Sox9 is essential for outer root sheath differentiation and the formation of the hair stem cell compartment. Curr. Biol. *15*, 1340–1351. https://doi.org/10.1016/j.cub.2005.06.064.

Viragova, S., Li, D., and Klein, O.D. (2024). Activation of fetal-like molecular programs during regeneration in the intestine and beyond. Cell Stem Cell *31*, 949–960. https://doi.org/10.1016/j.stem.2024.05.009.

Wagner, T., Wirth, J., Meyer, J., Zabel, B., Held, M., Zimmer, J., Pasantes, J., Bricarelli, F.D., Keutel, J., Hustert, E., et al. (1994). Autosomal sex reversal and campomelic dysplasia are caused by mutations in and around the SRY-related gene SOX9. Cell *79*, 1111–1120. https://doi.org/10.1016/0092-8674(94)90041-8.

Walther, N., Anantakrishnan, S., Graham, T.G.W., Dailey, G.M., Tjian, R., and Darzacq, X. (2024). Automated live-cell single-molecule tracking in enteroid monolayers reveals transcription factor dynamics probing lineage-determining function. Cell Rep. *43*, 114914. https://doi.org/10.1016/j.celrep.2024.114914.

Weidemüller, P., Kholmatov, M., Petsalaki, E., and Zaugg, J.B. (2021). Transcription factors: Bridge between cell signaling and gene regulation. Proteomics *21*, 1–14. https://doi.org/10.1002/pmic.202000034.

Yang, Y., Gomez, N., Infarinato, N., Adam, R.C., Sribour, M., Baek, I., Laurin, M., and Fuchs, E. (2023). The pioneer factor SOX9 competes for epigenetic factors to switch stem cell fates. Nat. Cell Biol. *25*, 1185–1195. https://doi.org/10.1038/s41556-023-01184-y.

Yu, F.X., Zhao, B., and Guan, K.L. (2015). Hippo Pathway in Organ Size Control, Tissue Homeostasis, and Cancer. Cell *163*, 811–828. https://doi.org/10.1016/j.cell.2015.10.044.

Yui, S., Azzolin, L., Maimets, M., Pedersen, M.T., Fordham, R.P., Hansen, S.L., Larsen, H.L., Guiu, J., Alves, M.R.P., Rundsten, C.F., et al. (2018). YAP/TAZ-Dependent Reprogramming of Colonic Epithelium Links ECM Remodeling to Tissue Regeneration. Cell Stem Cell *22*, 35–49.e7. https://doi.org/10.1016/j.stem.2017.11.001.

Zanconato, F., Forcato, M., Battilana, G., Azzolin, L., Quaranta, E., Bodega, B., Rosato, A., Bicciato, S., Cordenonsi, M., and Piccolo, S. (2015). Genome-wide association between YAP/TAZ/TEAD and AP-1 at enhancers drives oncogenic growth. Nat. Cell Biol. *17*, 1218–1227. https://doi.org/10.1038/ncb3216.

Zhao, B., Ye, X., Yu, J., Li, L., Li, W., Li, S., Yu, J., Lin, J.D., Wang, C.Y., Chinnaiyan, A.M., et al. (2008). TEAD mediates YAP-dependent gene induction and growth control. Genes Dev. *22*, 1962–1971. https://doi.org/10.1101/gad.1664408.

Zhou, J., Cattoglio, C., Shao, Y., Tirumala, H.P., Vetralla, C., Bajikar, S.S., Li, Y., Chen, H., Wang, Q., Wu, Z., et al. (2023). A novel pathogenic mutation of MeCP2 impairs chromatin association independent of protein levels. Genes Dev. *37*, 883–900. https://doi.org/10.1101/gad.350733.123.

Zwick, R.K., Kasparek, P., Palikuqi, B., Viragova, S., Weichselbaum, L., McGinnis, C.S., McKinley, K.L., Rathnayake, A., Vaka, D., Nguyen, V., et al. (2024). Epithelial zonation along the mouse and human small intestine defines five discrete metabolic domains. Nat. Cell Biol. *26*, 250–262. https://doi.org/10.1038/s41556-023-01337-z.

Stem Cell Reports, Volume *21*

Supplemental Information

Distinct SOX9 single-molecule dynamics characterize adult differentiation and fetal-like reprogrammed states in intestinal organoids

Nike Walther, Sathvik Anantakrishnan, Gina M. Dailey, Anna C. Maurer, and Claudia Cattoglio

Figure S1

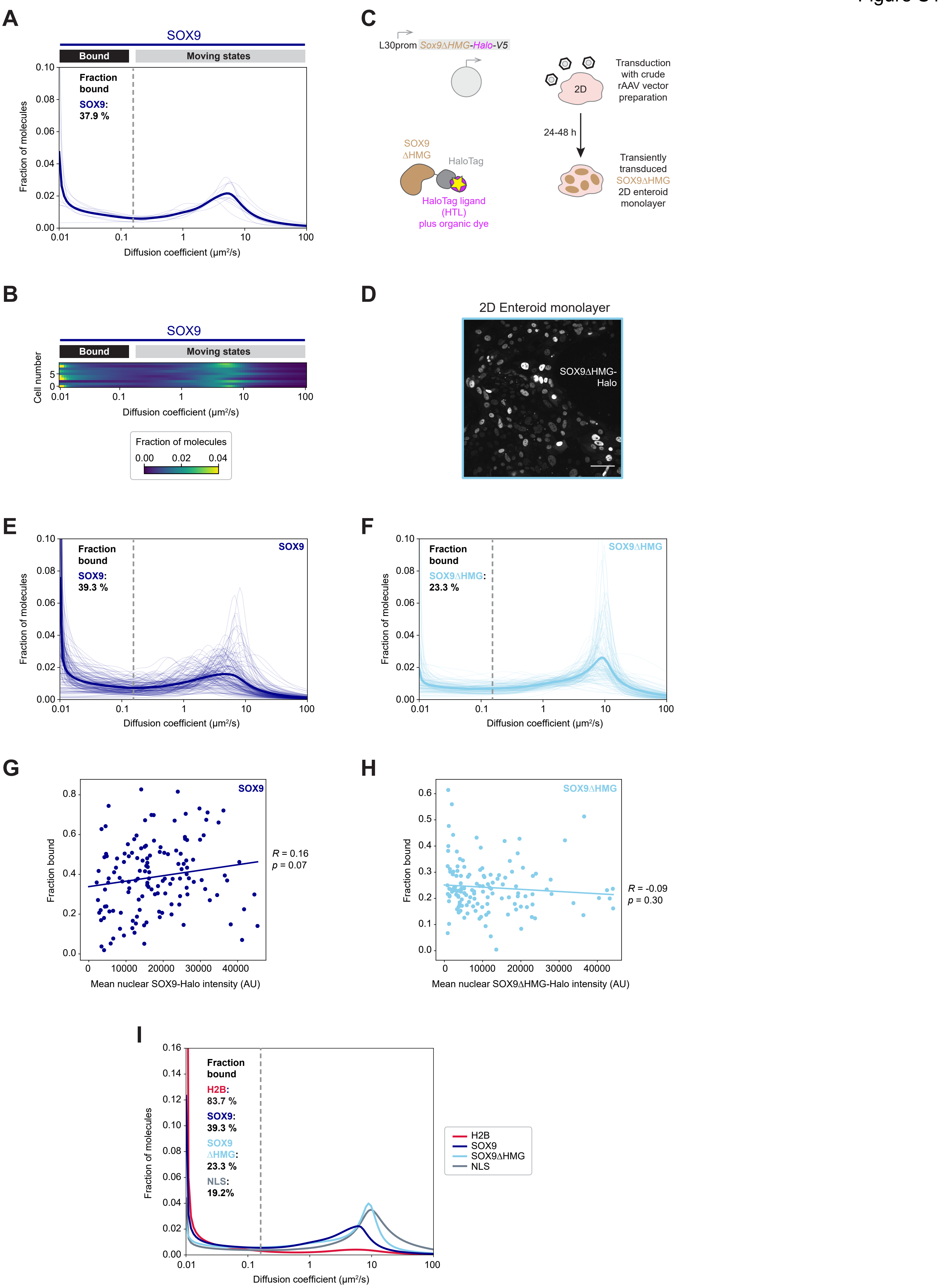
A
SOX9
Bound
Moving states
Fraction bound
SOX9: 37.9 %
Fraction of molecules
Diffusion coefficient (μm²/s)
B
SOX9
Bound
Moving states
Cell number
Diffusion coefficient (μm²/s)
Fraction of molecules
C
L30prom Sox9ΔHMG-Halo-V5
Transduction with crude rAAV vector preparation
2D
24-48 h
SOX9 ΔHMG
HaloTag
HaloTag ligand (HTL) plus organic dye
Transiently transduced SOX9ΔHMG 2D enteroid monolayer
D
2D Enteroid monolayer
SOX9ΔHMG-Halo
E
Fraction bound
SOX9: 39.3 %
SOX9
Fraction of molecules
Diffusion coefficient (μm²/s)
F
Fraction bound
SOX9ΔHMG: 23.3 %
SOX9ΔHMG
Fraction of molecules
Diffusion coefficient (μm²/s)
G
SOX9
Fraction bound
R = 0.16
p = 0.07
Mean nuclear SOX9-Halo intensity (AU)
H
SOX9ΔHMG
Fraction bound
R = -0.09
p = 0.30
Mean nuclear SOX9ΔHMG-Halo intensity (AU)
I
Fraction bound
H2B: 83.7 %
SOX9: 39.3 %
SOX9 ΔHMG: 23.3 %
NLS: 19.2%
H2B
SOX9
SOX9ΔHMG
NLS
Fraction of molecules
Diffusion coefficient (μm²/s)

<u>Figure S1:</u> The diffusive behaviors of SOX9-Halo and its DNA binding domain mutant SOX9∆HMG-Halo are expression level-independent, related to Figure 1. **(A,B)** Single-cell diffusion spectra (A) or diffusion heatmap (B) of a manual fast SMT experiment in SOX9-Halo 2D EMCs with a mean fraction bound of 37.9% (95% CI: 35.8-39.4%). In (B) cells are ordered by decreasing fraction bound from top to bottom (*n*=10 cells). **(C)** Generation of an organoid line transiently overexpressing a SOX9∆HMG-HaloTag-V5 transgene through transduction with a crude rAAV vector preparation allows fluorescence detection (yellow star) of SOX9∆HMG-Halo (brown-gray) covalently labeled with a dye-coupled HTL (magenta). **(D)** Confocal live imaging of a 2D EMC 48h post-transduction with a crude rAAV preparation to transiently express SOX9∆HMG-Halo. Scale bar: 50 µm. **(E,F)** Single-cell diffusion spectra for 4 independent automated experiments for SOX9-Halo (E) and 5 independent manual experiments for SOX9∆HMG-Halo (F). **(G,H)** Fractions bound for each cell are plotted against the mean nuclear fluorescence intensity for SOX9-Halo (G) or SOX9∆HMG-Halo (H). Correlations between POI intensity and fraction bound (fitted line) were computed for each POI and the Pearson correlation coefficients (*R*) and *p*-values are indicated. **(I)** Mean diffusion spectra for H2B-Halo (red), SOX9-Halo (dark blue), SOX9∆HMG-Halo (light blue), and Halo-NLS (gray). Bootstrap analysis of combined experiments with *n*=65,30,12,50,123,81,6 cells for H2B, *n*=10,44,42,56 cells for SOX9, *n*=16,42,34 cells for SOX9∆HMG, and *n*=12,47,41 cells for NLS determined mean fractions bound of 83.7% (95% CI: 81.4-85.9%), 39.3% (95% CI: 32.9-46.7%), 23.3% (95% CI: 19.5-28.5%), and 19.2% (95% CI: 15.0-23.2%) respectively. Cells in (E-I) correspond to the 4 experiments for SOX9 (dark blue; combined *n*=152 cells) and the 5 experiments for SOX9∆HMG (light blue; combined *n*=135 cells) plotted in Fig. 1E-I. Cells in (I) further correspond to the 7 experiments for H2B (red; combined *n*=367 cells) and the 3 experiments for NLS (gray; combined *n*=100 cells) plotted in Fig. 1G and Fig. S2H,I.

Figure S2

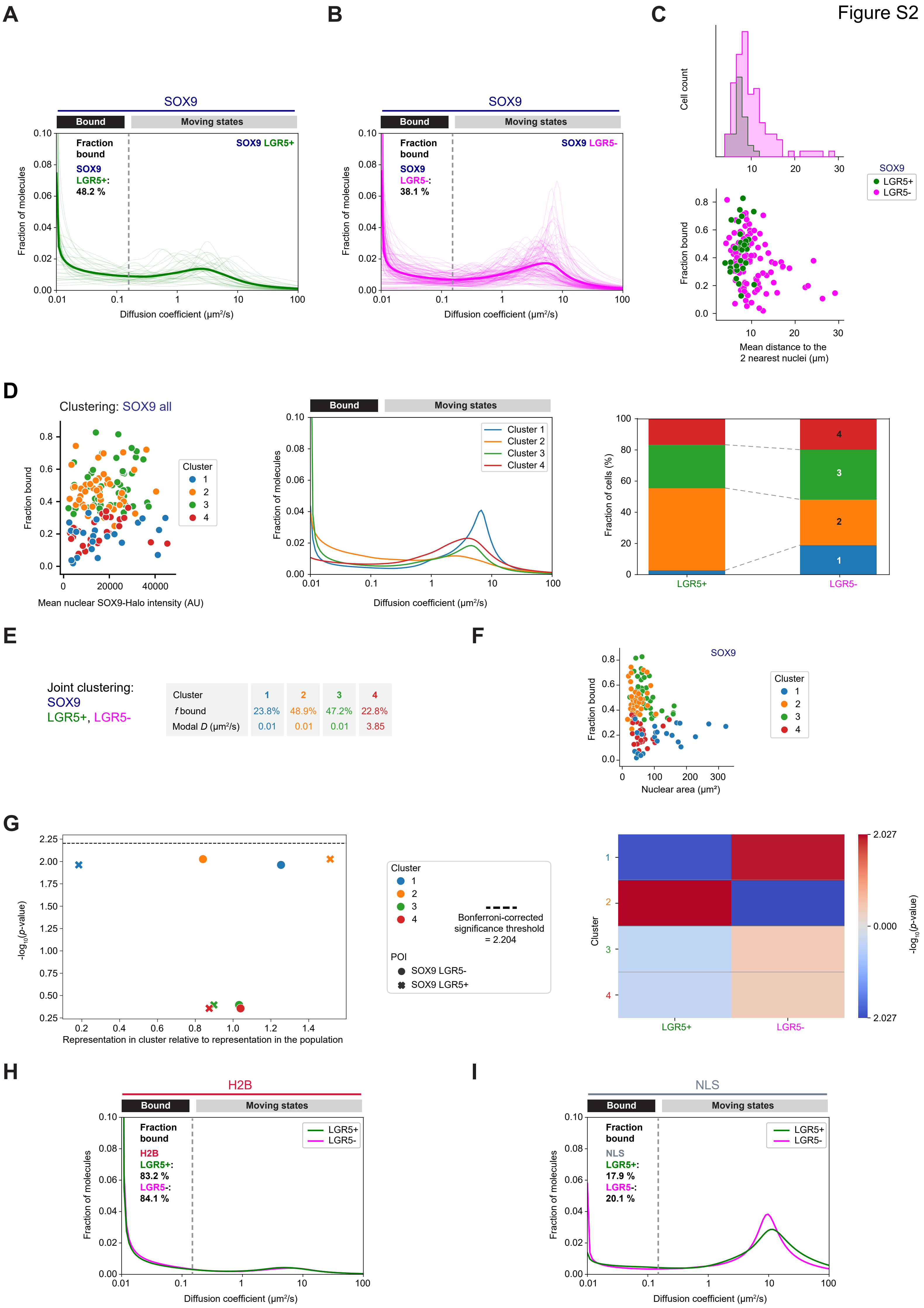

A
SOX9
Bound
Moving states
Fraction bound
SOX9
LGR5+:
48.2 %
SOX9 LGR5+
Fraction of molecules
Diffusion coefficient (µm²/s)
B
SOX9
Bound
Moving states
Fraction bound
SOX9
LGR5-:
38.1 %
SOX9 LGR5-
Fraction of molecules
Diffusion coefficient (µm²/s)
C
Cell count
SOX9
LGR5+
LGR5-
Fraction bound
Mean distance to the
2 nearest nuclei (µm)
D
Clustering: SOX9 all
Fraction bound
Cluster
Mean nuclear SOX9-Halo intensity (AU)
Bound
Moving states
Cluster 1
Cluster 2
Cluster 3
Cluster 4
Fraction of molecules
Diffusion coefficient (µm²/s)
Fraction of cells (%)
LGR5+
LGR5-
E
Joint clustering:
SOX9
LGR5+, LGR5-
Cluster 1 2 3 4
f bound 23.8% 48.9% 47.2% 22.8%
Modal D (µm²/s) 0.01 0.01 0.01 3.85
F
SOX9
Fraction bound
Cluster
Nuclear area (µm²)
G
-log10(p-value)
Representation in cluster relative to representation in the population
Cluster
Bonferroni-corrected
significance threshold
= 2.204
POI
SOX9 LGR5-
SOX9 LGR5+
Cluster
LGR5+
LGR5-
2.027
0.000
2.027
-log10(p-value)
H
H2B
Bound
Moving states
Fraction bound
H2B
LGR5+:
83.2 %
LGR5-:
84.1 %
LGR5+
LGR5-
Fraction of molecules
Diffusion coefficient (µm²/s)
I
NLS
Bound
Moving states
Fraction bound
NLS
LGR5+:
17.9 %
LGR5-:
20.1 %
LGR5+
LGR5-
Fraction of molecules
Diffusion coefficient (µm²/s)

<u>Figure S2:</u> Differences in the diffusive behavior of SOX9-Halo in stem- and differentiated cell populations, but not for Halo-tagged immobile H2B and freely diffusing NLS CTRLs, related to Figure 2. (A,B) Single-cell diffusion spectra for 4 independent automated experiments for SOX9-Halo classified into LGR5+ (green) (A) and LGR5- (magenta) (B) subpopulations with *n*=36 and *n*=116 cells, respectively. **(C)** Top: Distribution of the single-cell morphological characteristic mean distance to the two nearest nuclei for LGR5+/- (green/magenta) cells extracted from SMT data. Bottom: Single-cell correlation of SMT-derived fraction bound with mean distance to the two nearest nuclei for LGR5+/- (green/magenta) cells. **(D-G)** Hierarchical clustering of all SOX9-Halo cells based on single-cell diffusion spectra using the Jensen-Shannon distance metric. (D) Left: Fractions bound for each cell plotted against the mean nuclear SOX9-Halo intensity with color-coded diffusion clusters. Middle: Mean diffusion spectra for each cluster. Right: Distribution of cells into diffusion clusters for LGR5+/- (left/right). (E) Cluster statistics. (F) Fractions bound for each cell plotted against the nuclear area with color-coded diffusion clusters. (G) Left: *p*-values indicating the representation of LGR5+ (x mark) and LGR5- (circle) subpopulations in each diffusion cluster relative to the representation in the population; Bonferroni-corrected significance threshold (dashed line). Right: Heatmap of *p*-values indicating the representation of each subpopulation in each diffusion cluster (red: overrepresentation; blue: underrepresentation). Cluster: 1-blue, 2-orange, 3-green, 4-red. Shown are *n*=152 cells from 4 combined automated experiments (*n*=10,44,42,56 cells). **(H,I)** Mean diffusion spectra for LGR5+/- (green/magenta) subpopulations of (H) H2B-Halo (LGR5+: *n*=174 cells; LGR5-: *n*=190 cells) and (I) Halo-NLS (LGR5+: *n*=47 cells; LGR5-: *n*=53 cells). Bootstrap analysis of combined experiments with *n*=65,30,12,50,123,81,6 cells for H2B and *n*=12,47,41 cells for NLS determined mean fractions bound of 83.2% (95% CI: 81.1-85.3%) and 84.1% (95% CI: 81.7-86.4%) as well as 17.9% (95% CI: 13.7-22.5%) and 20.1% (95% CI: 15.9-23.6%) for LGR5+/- subpopulations, respectively. Comparison of the fraction bound distributions between LGR5+ and LGR5- cells yielded the following *p*-values: H2B – 0.125, NLS – 0.914. Representative SMT movies in Videos S4,5. The SOX9 data are the same as in Fig.1E-I, Fig. S1E,G,I, Fig. 2B-D, and Fig. 5C. The H2B and NLS data are the same as in Fig. 1G and Fig. S1E.

Figure S3

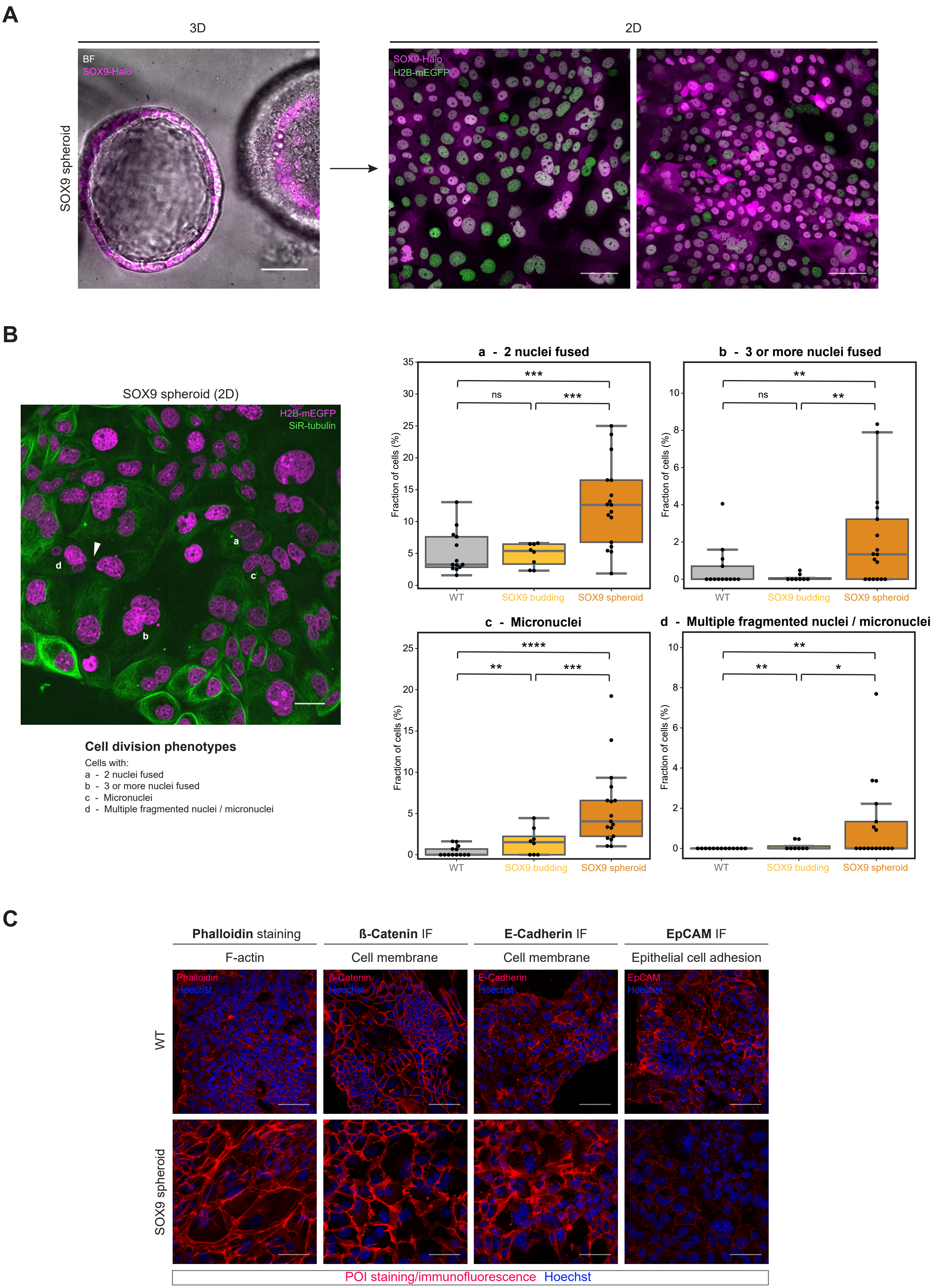
A
3D
2D
SOX9 spheroid
BF
SOX9-Halo
SOX9-Halo
H2B-mEGFP
B
SOX9 spheroid (2D)
H2B-mEGFP
SiR-tubulin
a
b
c
d
Cell division phenotypes
Cells with:
a - 2 nuclei fused
b - 3 or more nuclei fused
c - Micronuclei
d - Multiple fragmented nuclei / micronuclei
a - 2 nuclei fused
b - 3 or more nuclei fused
c - Micronuclei
d - Multiple fragmented nuclei / micronuclei
Fraction of cells (%)
WT
SOX9 budding
SOX9 spheroid
ns
*
**
***
****
C
Phalloidin staining
ß-Catenin IF
E-Cadherin IF
EpCAM IF
F-actin
Cell membrane
Cell membrane
Epithelial cell adhesion
Phalloidin
Hoechst
ß-Catenin
Hoechst
E-Cadherin
Hoechst
EpCAM
Hoechst
WT
SOX9 spheroid
POI staining/immunofluorescence Hoechst

<u>Figure S3:</u> SOX9-Halo spheroids display cell division errors, actin stress fibers, and signs of cell membrane remodeling, related to Figure 3. (A) Confocal live imaging of SOX9-Halo spheroids co-expressing H2B-mEGFP 5 d post-seeding. Left: 3D spheroid (BF: gray; SOX9-Halo: magenta). Right: 2D EMC derived from 3D spheroid (SOX9-Halo: magenta; H2B-mEGFP: green). Scale bars: 50 µm. **(B)** Cell division phenotypes in 2D EMCs derived from SOX9-Halo spheroids. Left: Representative confocal image of live SOX9 spheroid-derived 2D EMC (H2B-mEGFP: magenta; SiR-tubulin: green) with cell division phenotypes (a-d) and anaphase bridge (arrow) indicated. Scale bar: 20 µm. Right: Quantifications of cell division phenotypes (a: two nuclei fused; b: three or more nuclei fused; c: micronuclei; d: multiple fragmented nuclei or micronuclei) in 2D EMCs derived from WT (gray), SOX9-Halo_budding (yellow), or SOX9-Halo_spheroid (orange) organoids. Quantifications were based on 13 images with n=1821 cells for WT, 8 images with n=1626 cells for SOX9_budding, and 17 images with n=1756 cells for SOX9_spheroid. Each point represents one FOV; median: gray line, first/third quartile: whiskers; statistical testing based on Mann-Whitney U tests (see experimental procedures for details); (ns) non-significant, $p>0.5$; (*) $p\leq0.5$; (**) $p\leq0.01$; (***) $p\leq0.001$; (****) $p\leq0.0001$. **(C)** Confocal images of immunostained (POI: red) 2D EMCs derived from WT organoids (top) or SOX9-Halo spheroids (bottom) co-stained with Hoechst (blue). Scale bars: 50 µm.

Figure S4

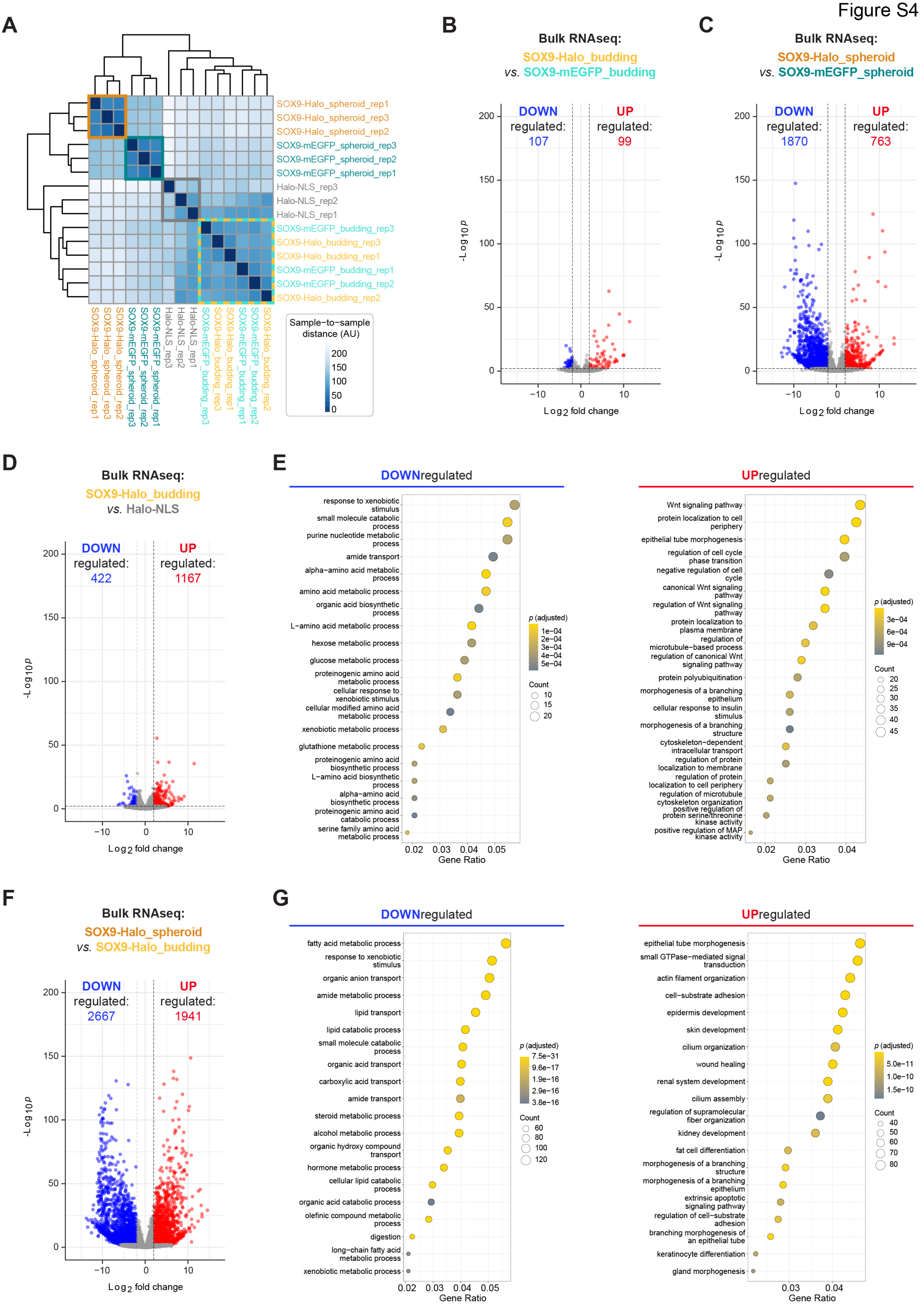

A
SOX9-Halo_spheroid_rep1
SOX9-Halo_spheroid_rep3
SOX9-Halo_spheroid_rep2
SOX9-mEGFP_spheroid_rep3
SOX9-mEGFP_spheroid_rep2
SOX9-mEGFP_spheroid_rep1
Halo-NLS_rep3
Halo-NLS_rep2
Halo-NLS_rep1
SOX9-mEGFP_budding_rep3
SOX9-Halo_budding_rep3
SOX9-Halo_budding_rep1
SOX9-mEGFP_budding_rep1
SOX9-mEGFP_budding_rep2
SOX9-Halo_budding_rep2
Sample−to−sample distance (AU)
200
150
100
50
0
B
Bulk RNAseq:
SOX9-Halo_budding
vs. SOX9-mEGFP_budding
DOWN regulated: 107
UP regulated: 99
-Log10 p
Log2 fold change
C
Bulk RNAseq:
SOX9-Halo_spheroid
vs. SOX9-mEGFP_spheroid
DOWN regulated: 1870
UP regulated: 763
D
Bulk RNAseq:
SOX9-Halo_budding
vs. Halo-NLS
DOWN regulated: 422
UP regulated: 1167
E
DOWNregulated
response to xenobiotic stimulus
small molecule catabolic process
purine nucleotide metabolic process
amide transport
alpha−amino acid metabolic process
amino acid metabolic process
organic acid biosynthetic process
L−amino acid metabolic process
hexose metabolic process
glucose metabolic process
proteinogenic amino acid metabolic process
cellular response to xenobiotic stimulus
cellular modified amino acid metabolic process
xenobiotic metabolic process
glutathione metabolic process
proteinogenic amino acid biosynthetic process
L−amino acid biosynthetic process
alpha−amino acid biosynthetic process
proteinogenic amino acid catabolic process
serine family amino acid metabolic process
Gene Ratio
p (adjusted)
1e−04
2e−04
3e−04
4e−04
5e−04
Count
10
15
20
UPregulated
Wnt signaling pathway
protein localization to cell periphery
epithelial tube morphogenesis
regulation of cell cycle phase transition
negative regulation of cell cycle
canonical Wnt signaling pathway
regulation of Wnt signaling pathway
protein localization to plasma membrane
regulation of microtubule−based process
regulation of canonical Wnt signaling pathway
protein polyubiquitination
morphogenesis of a branching epithelium
cellular response to insulin stimulus
morphogenesis of a branching structure
cytoskeleton−dependent intracellular transport
regulation of protein localization to membrane
regulation of protein localization to cell periphery
regulation of microtubule cytoskeleton organization
positive regulation of protein serine/threonine kinase activity
positive regulation of MAP kinase activity
p (adjusted)
3e−04
6e−04
9e−04
Count
20
25
30
35
40
45
F
Bulk RNAseq:
SOX9-Halo_spheroid
vs. SOX9-Halo_budding
DOWN regulated: 2667
UP regulated: 1941
G
DOWNregulated
fatty acid metabolic process
response to xenobiotic stimulus
organic anion transport
amide metabolic process
lipid transport
lipid catabolic process
small molecule catabolic process
organic acid transport
carboxylic acid transport
amide transport
steroid metabolic process
alcohol metabolic process
organic hydroxy compound transport
hormone metabolic process
cellular lipid catabolic process
organic acid catabolic process
olefinic compound metabolic process
digestion
long−chain fatty acid metabolic process
xenobiotic metabolic process
p (adjusted)
7.5e−31
9.6e−17
1.9e−16
2.9e−16
3.8e−16
Count
60
80
100
120
UPregulated
epithelial tube morphogenesis
small GTPase−mediated signal transduction
actin filament organization
cell−substrate adhesion
epidermis development
skin development
cilium organization
wound healing
renal system development
cilium assembly
regulation of supramolecular fiber organization
kidney development
fat cell differentiation
morphogenesis of a branching structure
morphogenesis of a branching epithelium
extrinsic apoptotic signaling pathway
regulation of cell−substrate adhesion
branching morphogenesis of an epithelial tube
keratinocyte differentiation
gland morphogenesis
p (adjusted)
5.0e−11
1.0e−10
1.5e−10
Count
40
50
60
70
80

__Figure S4:__ **Enteroid reversion to a fetal-like state upon SOX9 overexpression is tag-independent and occurs through intermediate states with upregulated Wnt signaling-dependent pathways, related to Figure 4. (A)** Sample-to-sample distances of the organoid samples Halo-NLS (gray), SOX9-mEGFP_budding (light turquoise), SOX9-Halo_budding (yellow), SOX9-mEGFP_spheroid (dark turquoise), and SOX9-Halo_spheroid (orange) in biological triplicates of bulk RNAseq experiments. **(B,C)** Volcano plot displaying DEGs (adjusted *p*-value ≤0.01, fold change ≥2 and mean counts ≥10; red/blue: up-/downregulated) in (B) SOX9-Halo_budding in comparison to SOX9-mEGFP_budding organoids or (C) SOX9-Halo spheroids in comparison to SOX9-mEGFP spheroids determined by bulk RNAseq. **(D)** Volcano plot displaying DEGs (adjusted *p*-value ≤0.01, fold change ≥2 and mean counts ≥10; red/blue: up-/downregulated) in SOX9-Halo_budding in comparison to Halo-NLS CTRL organoids determined by bulk RNAseq. **(E)** GO analysis for the top 20 biological pathways enriched in DEGs down- (left, blue) or upregulated (right, red) in SOX9-Halo_budding in comparison to Halo-NLS CTRL organoids with adjusted *p*-values and gene counts indicated. **(F)** Volcano plot displaying DEGs (adjusted *p*-value ≤0.01, fold change ≥2 and mean counts ≥10; red/blue: up-/downregulated) in SOX9-Halo spheroids in comparison to SOX9-Halo_budding organoids determined by bulk RNAseq. **(G)** GO analysis for the top 20 biological pathways enriched in DEGs down- (left, blue) or upregulated (right, red) in SOX9-Halo spheroids in comparison to SOX9-Halo_budding organoids with adjusted *p*-values and gene counts indicated. Data shown refers to the bulk RNAseq experiment from Fig. 4B-D and Fig. S5.

Figure S5

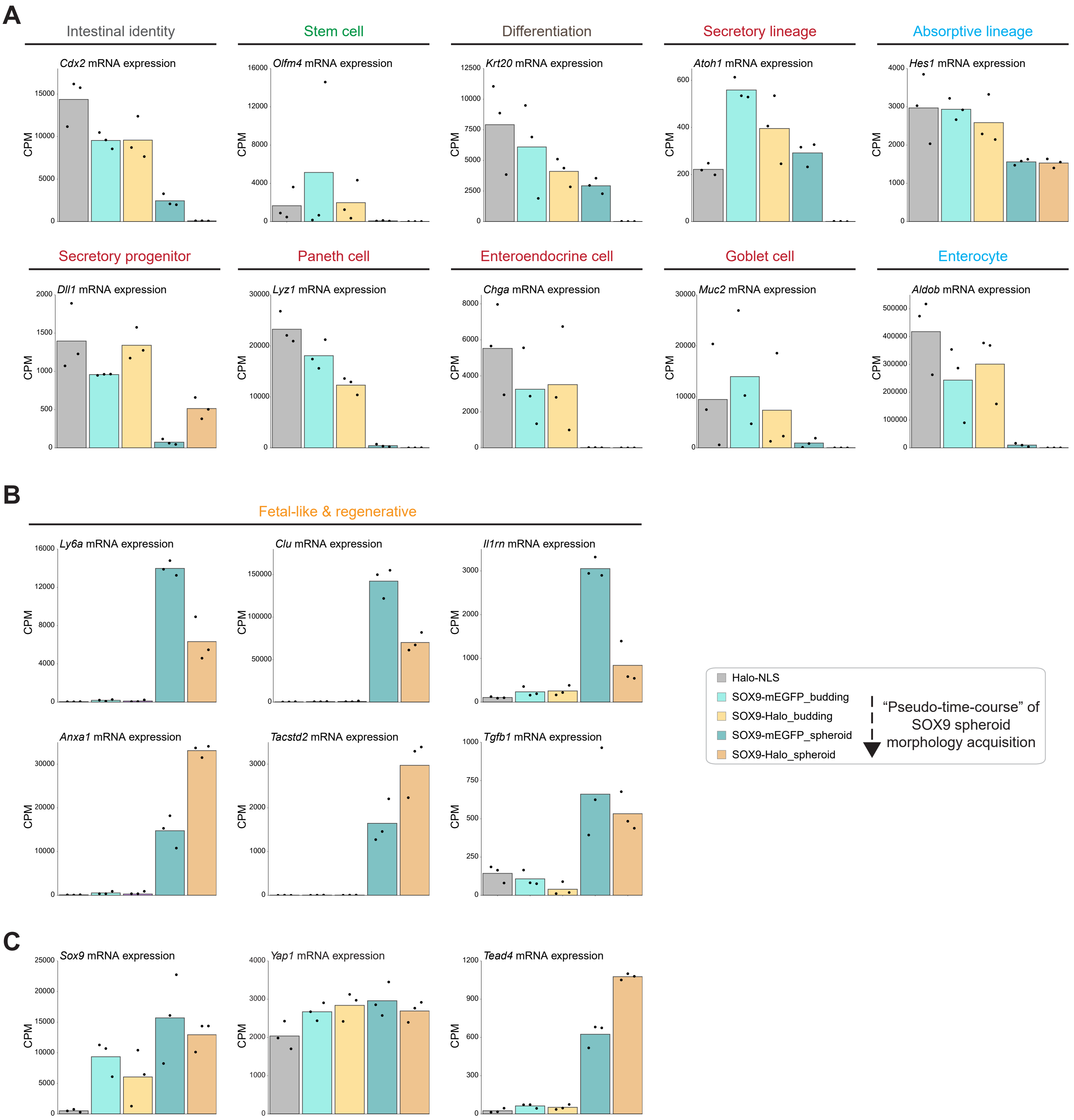

A
Intestinal identity
Cdx2 mRNA expression
Stem cell
Olfm4 mRNA expression
Differentiation
Krt20 mRNA expression
Secretory lineage
Atoh1 mRNA expression
Absorptive lineage
Hes1 mRNA expression
Secretory progenitor
Dll1 mRNA expression
Paneth cell
Lyz1 mRNA expression
Enteroendocrine cell
Chga mRNA expression
Goblet cell
Muc2 mRNA expression
Enterocyte
Aldob mRNA expression
B
Fetal-like & regenerative
Ly6a mRNA expression
Clu mRNA expression
Il1rn mRNA expression
Anxa1 mRNA expression
Tacstd2 mRNA expression
Tgfb1 mRNA expression
C
Sox9 mRNA expression
Yap1 mRNA expression
Tead4 mRNA expression
CPM
Halo-NLS
SOX9-mEGFP_budding
SOX9-Halo_budding
SOX9-mEGFP_spheroid
SOX9-Halo_spheroid
"Pseudo-time-course" of SOX9 spheroid morphology acquisition

__Figure S5:__ **A "pseudo-time-course" of spheroid morphology acquisition upon SOX9 overexpression in enteroids reveals a reduction in intestinal epithelial signatures across lineages counteracted by the induction of a gene expression program resembling fetal-like reversion, which includes the upregulation of *Tead* acting downstream of YAP, related to Figure 4. (A-C)** Selected DEGs in the stable organoid lines Halo-NLS (gray), SOX9-mEGFP_budding (light turquoise), SOX9-Halo_budding (yellow), SOX9-mEGFP_spheroid (dark turquoise), and SOX9-Halo_spheroid (orange) determined by bulk RNAseq in biological triplicates. The mean counts per million mapped reads (CPM) of three replicates (bar) and the CPMs for each replicate (points) are indicated. Samples are ordered according to passage number (time in culture) and degree of spheroid phenotype acquisition. (A) Intestinal identity, stem and differentiation markers of both secretory and absorptive lineages. (B) Regenerative fetal-like markers. (C) *Sox9*, *Yap1*, *Tead4*. Data shown refer to the bulk RNAseq experiment in Fig. 3F,G, Fig. 4B-D and Fig. S4.

Figure S6

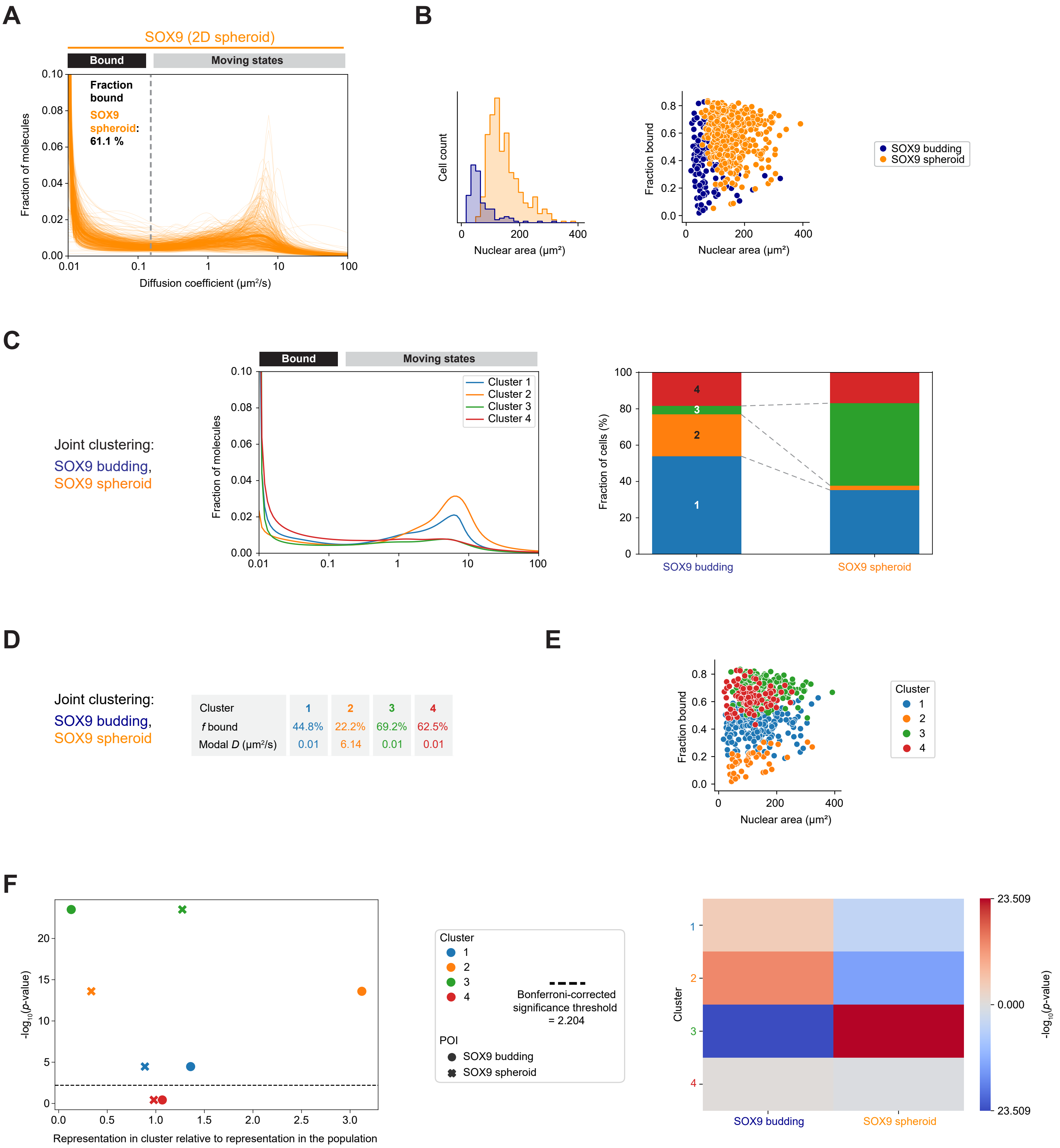
A
SOX9 (2D spheroid)
Bound
Moving states
Fraction bound
SOX9 spheroid: 61.1 %
Fraction of molecules
Diffusion coefficient (μm²/s)
B
Cell count
Nuclear area (μm²)
Fraction bound
SOX9 budding
SOX9 spheroid
C
Joint clustering:
SOX9 budding,
SOX9 spheroid
Bound
Moving states
Cluster 1
Cluster 2
Cluster 3
Cluster 4
Fraction of molecules
Fraction of cells (%)
D
Joint clustering:
SOX9 budding,
SOX9 spheroid
Cluster 1 2 3 4
f bound 44.8% 22.2% 69.2% 62.5%
Modal D (μm²/s) 0.01 6.14 0.01 0.01
E
Fraction bound
Nuclear area (μm²)
Cluster
F
-log10(p-value)
Representation in cluster relative to representation in the population
Cluster
Bonferroni-corrected significance threshold = 2.204
POI
SOX9 budding
SOX9 spheroid
23.509
0.000
23.509

Figure S6: Unlike in budding enteroids, a larger fraction of immobile SOX9-Halo molecules is present in spheroid-derived EMCs with larger nuclei, related to Figure 5. (A) Single-cell diffusion spectra for 10 independent automated experiments for SOX9-Halo in spheroid-derived 2D EMCs. **(B)** Left: Nuclear area distribution for SOX9-Halo_budding (dark blue) and SOX9-Halo_spheroid (orange) cells determined from SMT data in 2D EMCs. Right: Single-cell correlation of SMT-derived fraction bound with nuclear area for SOX9-Halo_budding (dark blue) and SOX9-Halo_spheroid (orange). **(C-F)** Joint hierarchical clustering of SOX9-Halo_budding and SOX9-Halo_spheroid based on single-cell diffusion spectra using the Jensen-Shannon distance metric. (C) Left: Mean diffusion spectra of each cluster. Right: Distribution of cells from both SOX9-Halo samples into diffusion clusters. (D) Cluster statistics. (E) Fractions bound for each cell against the nuclear area with color-coded diffusion clusters. (F) Left: *p*-values indicating the representation of SOX9-Halo_budding (circle) and SOX9-Halo_spheroid (x mark) in each diffusion cluster relative to the representation in the population; Bonferroni-corrected significance threshold (dashed line). Right: Heatmap of *p*-values indicating the representation of each sample in each diffusion cluster (red: overrepresentation; blue: underrepresentation). Cluster: 1-blue, 2-orange, 3-green, 4-red. The SOX9-Halo_budding data are the same as in Fig. 1E-I, Fig. 2B-D, Fig. 5C, Fig. S1E,G,I, and Fig. S2A-G. The SOX9-Halo_spheroid data are the same as in Fig. 5B-D.

Figure S7

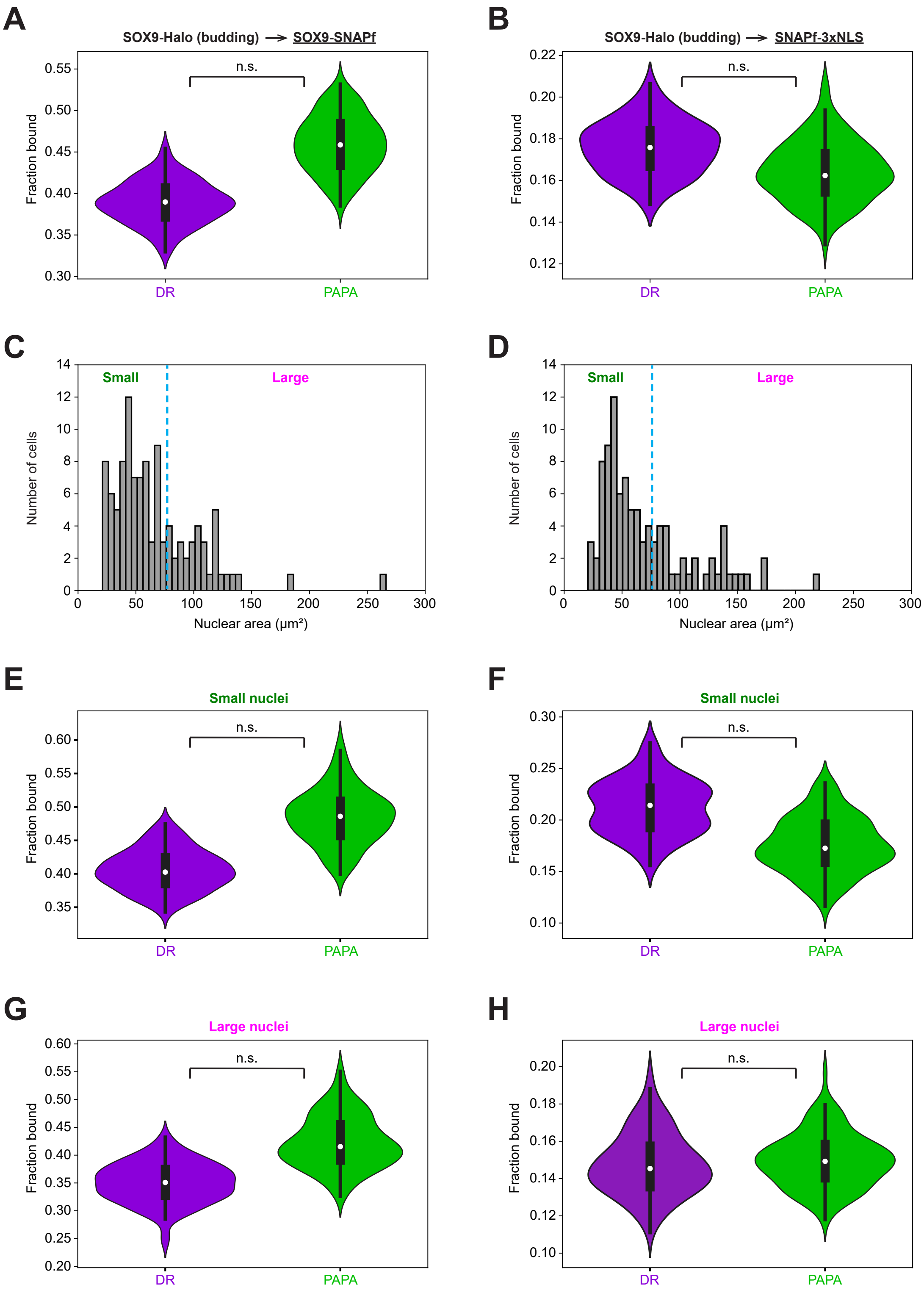
A
SOX9-Halo (budding) → SOX9-SNAPf
n.s.
Fraction bound
0.55
0.50
0.45
0.40
0.35
0.30
DR
PAPA
B
SOX9-Halo (budding) → SNAPf-3xNLS
n.s.
Fraction bound
0.22
0.20
0.18
0.16
0.14
0.12
DR
PAPA
C
Small
Large
Number of cells
Nuclear area (μm²)
D
Small
Large
Number of cells
Nuclear area (μm²)
E
Small nuclei
n.s.
Fraction bound
DR
PAPA
F
Small nuclei
n.s.
Fraction bound
DR
PAPA
G
Large nuclei
n.s.
Fraction bound
DR
PAPA
H
Large nuclei
n.s.
Fraction bound
DR
PAPA

<u>Figure S7:</u> PAPA-SMT reveals a chromatin-bound pool of self-associated SOX9 in live 2D EMCs derived from budding SOX9-Halo enteroids, related to Figure 6. (A,B) PAPA experiments in 2D EMCs derived from SOX9-Halo_budding organoids. Violin plots for fractions bound (median: white point; first/third quartile: whiskers) of SOX9-Halo→SOX9-SNAPf (A) and SOX9-Halo→SNAPf-3xNLS (B) determined from DR/PAPA (purple/green) trajectories. Data in (A) are from 6 combined experiments with *n*=108 cells (*n*=6,25,32,7,29,9 cells; 1661 DR and 1191 PAPA trajectories; bootstrapped fractions bound: 38.9±4.9% (DR) and 46.0±6.6% (PAPA)). Data in (B) are from 5 combined experiments with *n*=97 cells (*n*=22,44,2,14,15 cells; 3579 DR and 2644 PAPA trajectories; bootstrapped fractions bound: 17.6±2.5% (DR) and 16.4±2.8% (PAPA)). **(C,D)** Histograms of the nuclear area of cells from (A,B) with indicated threshold of 75 μm^2 (blue dotted line) used to split the whole cell population into two subpopulations with small or large nuclei. **(E-H)** PAPA results on subpopulations of cells with (E,F) small (*n*=148 or *n*=126 cells, respectively) or (G,H) large nuclei (*n*=68 cells for both conditions) from the experiments shown in (A,B). Bootstrapped fractions bound: (E) – 40.5±5.9% (DR) and 48.6±7.9% (PAPA); (F) – 21.3±5.1% (DR) and 17.6±5.2% (PAPA); (G) – 35.0±7.0% (DR) and 42.3±9.1% (PAPA); (H) – 14.8±3.2% (DR) and 15.0±2.8% (PAPA). (n.s.) $p>0.05$. For statistical details see experimental procedures.

Figure S8

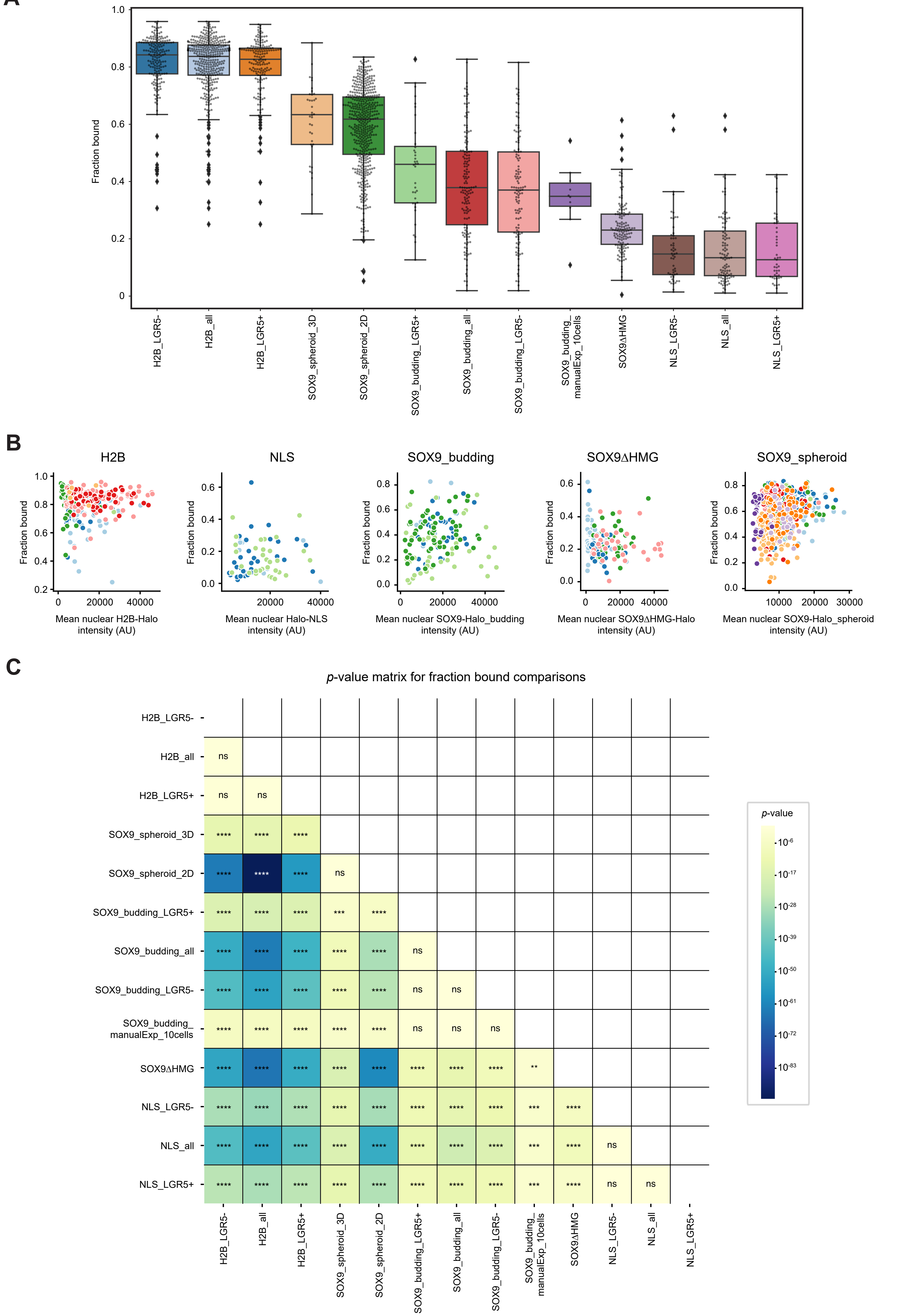
A
Fraction bound
H2B_LGR5-
H2B_all
H2B_LGR5+
SOX9_spheroid_3D
SOX9_spheroid_2D
SOX9_budding_LGR5+
SOX9_budding_all
SOX9_budding_LGR5-
SOX9_budding_manualExp_10cells
SOX9ΔHMG
NLS_LGR5-
NLS_all
NLS_LGR5+
B
H2B
NLS
SOX9_budding
SOX9ΔHMG
SOX9_spheroid
Mean nuclear H2B-Halo intensity (AU)
Mean nuclear Halo-NLS intensity (AU)
Mean nuclear SOX9-Halo_budding intensity (AU)
Mean nuclear SOX9ΔHMG-Halo intensity (AU)
Mean nuclear SOX9-Halo_spheroid intensity (AU)
C
p-value matrix for fraction bound comparisons
p-value
ns
****
***
**

<u>Figure S8:</u> Fraction bound distributions and comparisons. (A) Single-cell fraction bound distributions for all POI-Halo conditions measured by fast SMT in intestinal organoid models in this study. Boxplots with the fraction bound for each cell plotted (gray line: median; whiskers: first/third quartile). **(B)** Single-cell fraction bound distributions across experiments determined by fast SMT in 2D EMCs are plotted against the mean nuclear POI-Halo intensity for POI-Halo conditions H2B, NLS, SOX9_budding, SOX9ΔHMG, and SOX9_spheroid (left to right) with cells differentially colored by experiment. **(C)** Significance matrix of pairwise comparisons of fractions bound of all POI-Halo samples acquired by fast SMT in intestinal organoid models in this study ((ns) non-significant, p>0.05; (**) p≤0.01; (***) p≤0.001; (****) p≤0.0001; p-value color-coded). Mann-Whitney U-tests were performed to compare fraction bound distributions between two conditions. The Benjamini–Hochberg procedure was used to correct for multiple comparisons.

Figure S9

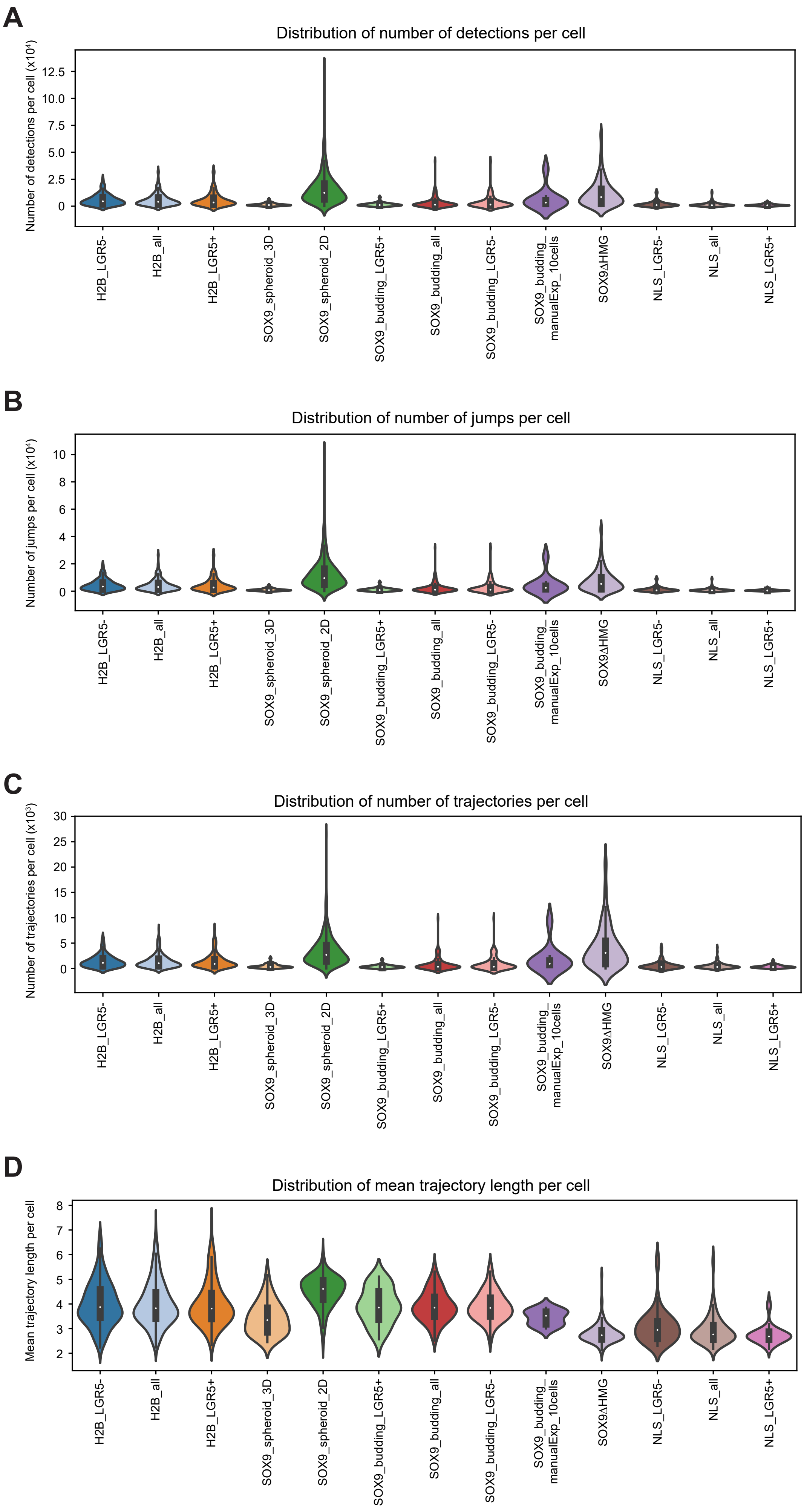
A
Distribution of number of detections per cell
Number of detections per cell (x10^4)
12.5
10.0
7.5
5.0
2.5
0
H2B_LGR5-
H2B_all
H2B_LGR5+
SOX9_spheroid_3D
SOX9_spheroid_2D
SOX9_budding_LGR5+
SOX9_budding_all
SOX9_budding_LGR5-
SOX9_budding_
manualExp_10cells
SOX9ΔHMG
NLS_LGR5-
NLS_all
NLS_LGR5+
B
Distribution of number of jumps per cell
Number of jumps per cell (x10^4)
10
8
6
4
2
0
H2B_LGR5-
H2B_all
H2B_LGR5+
SOX9_spheroid_3D
SOX9_spheroid_2D
SOX9_budding_LGR5+
SOX9_budding_all
SOX9_budding_LGR5-
SOX9_budding_
manualExp_10cells
SOX9ΔHMG
NLS_LGR5-
NLS_all
NLS_LGR5+
C
Distribution of number of trajectories per cell
Number of trajectories per cell (x10^3)
30
25
20
15
10
5
0
H2B_LGR5-
H2B_all
H2B_LGR5+
SOX9_spheroid_3D
SOX9_spheroid_2D
SOX9_budding_LGR5+
SOX9_budding_all
SOX9_budding_LGR5-
SOX9_budding_
manualExp_10cells
SOX9ΔHMG
NLS_LGR5-
NLS_all
NLS_LGR5+
D
Distribution of mean trajectory length per cell
Mean trajectory length per cell
8
7
6
5
4
3
2
H2B_LGR5-
H2B_all
H2B_LGR5+
SOX9_spheroid_3D
SOX9_spheroid_2D
SOX9_budding_LGR5+
SOX9_budding_all
SOX9_budding_LGR5-
SOX9_budding_
manualExp_10cells
SOX9ΔHMG
NLS_LGR5-
NLS_all
NLS_LGR5+

<u>Figure S9:</u> Statistics for single-molecule detection and tracking across fast SMT conditions. Violin plots for the distribution of **(A)** the number of detections, **(B)** the number of jumps, **(C)** the number of trajectories, and **(D)** the mean trajectory length per cell for each POI-Halo sample measured by fast SMT in intestinal organoid models in this study (white point: median; whiskers: first/third quartile).

Figure S10

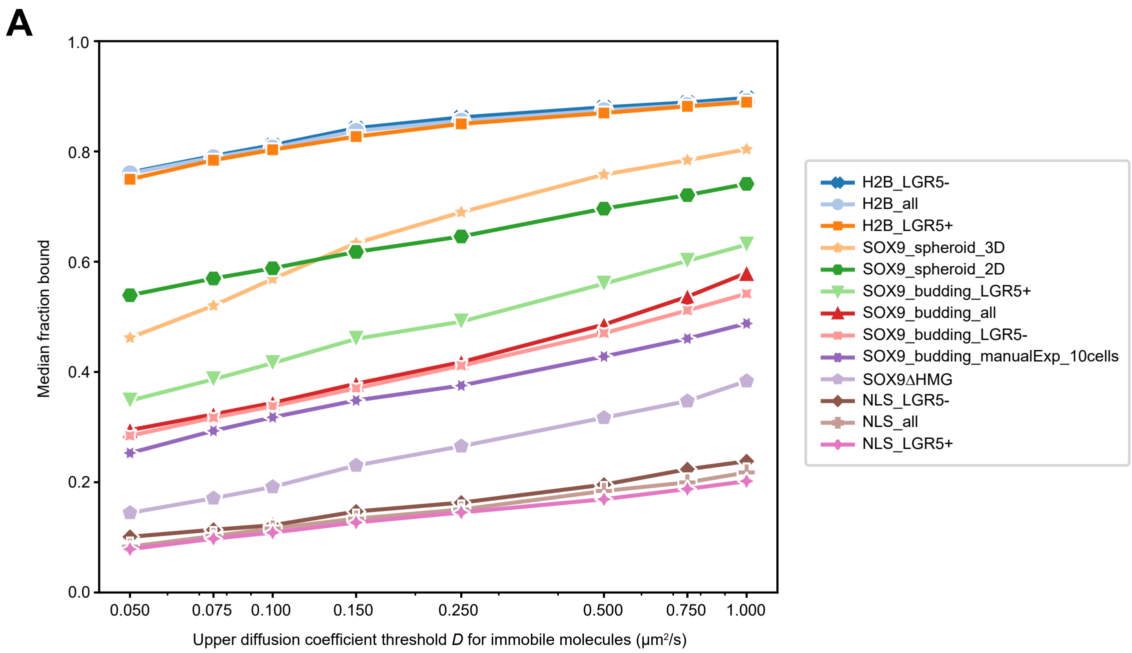
A
Median fraction bound
1.0
0.8
0.6
0.4
0.2
0.0
0.050
0.075
0.100
0.150
0.250
0.500
0.750
1.000
Upper diffusion coefficient threshold D for immobile molecules (µm²/s)
H2B_LGR5-
H2B_all
H2B_LGR5+
SOX9_spheroid_3D
SOX9_spheroid_2D
SOX9_budding_LGR5+
SOX9_budding_all
SOX9_budding_LGR5-
SOX9_budding_manualExp_10cells
SOX9ΔHMG
NLS_LGR5-
NLS_all
NLS_LGR5+

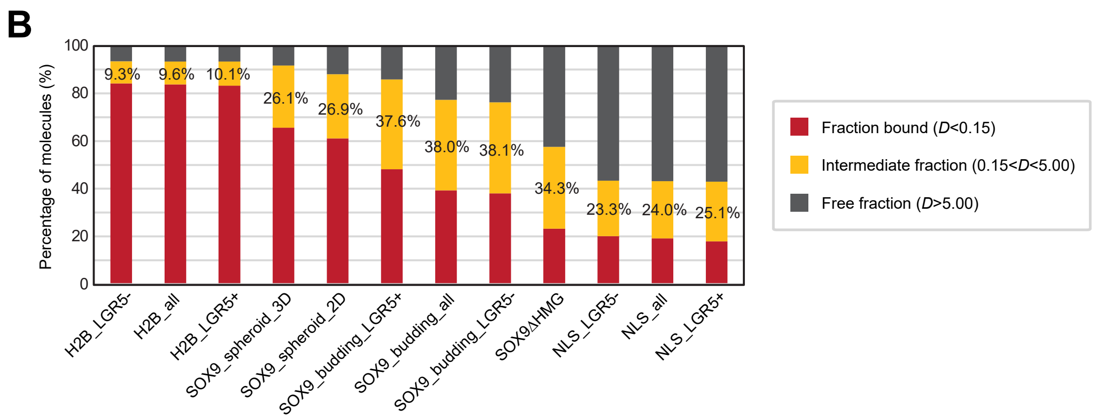
B
Percentage of molecules (%)
100
80
60
40
20
0
9.3%
9.6%
10.1%
26.1%
26.9%
37.6%
38.0%
38.1%
34.3%
23.3%
24.0%
25.1%
H2B_LGR5-
H2B_all
H2B_LGR5+
SOX9_spheroid_3D
SOX9_spheroid_2D
SOX9_budding_LGR5+
SOX9_budding_all
SOX9_budding_LGR5-
SOX9ΔHMG
NLS_LGR5-
NLS_all
NLS_LGR5+
Fraction bound (D<0.15)
Intermediate fraction (0.15<D<5.00)
Free fraction (D>5.00)

<u>Figure S10:</u> Immobile and freely diffusing fractions are the primary determinants of POI-Halo diffusion behavior. (A) Median fractions bound for each POI-Halo sample measured by fast SMT in intestinal organoid models in this study using various diffusion coefficients *D* (0.05 – 1.00 $\mu m^2/s$) as the upper limit (exclusive) for molecules considered as immobile and thus accounted for in the fraction bound. **(B)** Distribution of molecules into immobile ($D < 0.15$ $\mu m^2/s$; red), slowly diffusing (0.15 $\mu m^2/s < D < 5$ $\mu m^2/s$; yellow) and freely diffusing ($D > 5$ $\mu m^2/s$; gray) fractions for all POI-Halo samples measured by fast SMT in intestinal organoid models in this study. The percentage of molecules present in the intermediate fraction is indicated.

Supplemental methods

Supplemental experimental procedures

Cloning of DNA constructs

For the production of lentivirus to deliver protein of interest (POI)-tag transgenes into organoids, the third generation lentiviral pHAGE vector originally developed in the lab of Richard Mulligan (Murphy et al., 2006) was used together with the second generation lentiviral packaging plasmid psPAX2 (gift from Didier Trono (Addgene plasmid #12260; http://n2t.net/addgene:12260; RRID:Addgene_12260)) and the VSV-G envelope expressing plasmid pMD2.G (gift from Didier Trono (Addgene plasmid #12259; http://n2t.net/addgene:12259; RRID:Addgene_12259)). For constitutive expression of POI-tag under the weak L30 promoter, pHAGE L30 IRES Puro (Walther et al., 2024), pHAGE L30 IRES Zeo, and pHAGE L30 IRES Neo backbones were created. Transgenes encoding the POI-tags H2B-Halo-V5 (Walther et al., 2024), V5-Halo-NLS (Walther et al., 2024), mSOX9-Halo-V5, and mSOX9-mEGFP-V5 were cloned into the Puro vector, H2B-mEGFP-V5 was cloned into the Zeo vector, and H2B-mScarletI-V5 was cloned into the Neo vector. For mSOX9, the DNA sequence encoding the reference 507aa isoform of UniProt entry Q04887 was cloned (NCBI Gene ID 20682, NM_011448.4). The final pHAGE constructs were confirmed by Sanger sequencing.

For the preparation of crude rAAV to deliver POI-tag encoding transgenes transiently into 2D EMCs, expression cassettes of L30prom_mSOX9deltaHMG-NLS-Halo-V5 or L30prom_mSOX9-SNAPf-ALFA (Götzke et al., 2019) were cloned in a pAAV vector derived from pAAV.CMV.Luc.IRES.EGFP.SV40 (gift from James M. Wilson (Addgene plasmid #105533; http://n2t.net/addgene:105533)) to create *cis* plasmids. The helper plasmid pAdDeltaF6 (gift from James M. Wilson (Addgene plasmid #112867; http://n2t.net/addgene:112867)) and the *trans* rep-cap AAV-KP1 plasmid (gift from Mark Kay (Addgene plasmid #206504; http://n2t.net/addgene:206504; (Pekrun et al., 2019)) were used together with a *cis* plasmid. pAAV bacterial cultures were grown at 30°C and the final rAAV constructs were confirmed by whole plasmid sequencing.

For further details, the plasmid maps are available on GitLab (https://gitlab.com/nikewalther/walther_sox9organoid_2025/-/tree/main/PlasmidMaps?ref_type=heads).

Lentivirus preparation

Lentivirus preparation using Hek293T Lenti-X cells (Takara, cat.# 632180) including lentivirus concentration using Lenti-X concentrator (Takara, cat.# 631231) was performed as described (Walther et al., 2024).

Generation of crude rAAV vector preparations

The generation of crude rAAV vector preparations was based on the protocol described (Benyamini et al., 2023). In brief, $3x10^5$ Hek293T cells (CRL-3216, ATCC) were seeded per well of a 6-well plate (Thermo Fisher Scientific, cat.# 140675) in high-glucose Dulbecco's modified eagle medium (DMEM) containing GlutaMAX-I (Gibco, cat.# 2537044), supplemented with 10% (vol/vol) fetal bovine serum (FBS; HyClone, cat.# SH30910.03, LOT# AXJ47554), and 1 mM sodium pyruvate (Gibco, cat.# 11360070), and grown to 75-90% confluency. For transfection of a 6-well, a plasmid mixture of 1.3 µg *trans* rep-cap AAV-KP1 plasmid, 2.6 µg helper plasmid pAdDeltaF6, and 1.3 µg gene of interest (GOI)-encoding *cis* plasmid (see "Cloning of DNA constructs") was prepared in serum-free DMEM to 100 µL per well. After addition of 5.2 µL 1 µg/µL polyethylenimine (PEI) hydrochloride MAX (Polysciences, cat.# 24765-1) in distilled H_2O (pH 7.1, filter sterilized), the mixture was pulsed 10-15x on a vortexer. Following incubation for 15 min at RT, 1.9 mL serum-free DMEM was added and mixing was performed by gently pipetting up and down two times. After removal of the culture medium, 2 mL of transfection mixture was added per well and the plate was incubated at 37°C and 5% CO_2 for 72 h. The 6-well plate was frozen at -80°C for 30 min, followed by thawing at 37°C for 30 min. Freezing and thawing was repeated for a total of three cycles. Each well was mixed by pipetting and the cell lysate was transferred to a 2 mL tube. To remove cell debris, centrifugation was performed at 15,000 x *g* for 15 min at RT. The supernatant (SN) was removed and transferred to a new 2 mL tube. This crude rAAV vector preparation was stored at 4°C for several weeks before being used for transduction of 2D EMCs.

Mouse small intestinal crypt preparation for growing small intestinal organoids

Mouse small intestinal crypt preparations for growing small intestinal organoids were performed as described (Walther et al., 2024) with the exception that female mice at an age of 8-16 weeks were used for this study.

Mouse small intestinal organoid culture

Mouse small intestinal organoids were grown in Matrigel domes (90% (vol/vol) Matrigel Matrix for Organoid Culture (Corning, cat.# 08774406)) and cultured in IntestiCult Organoid Growth Medium (Mouse) (Stem Cell Technologies, cat.# 06005) supplemented with 1% (vol/vol) Penicillin/Streptomycin (Pen/Strep; Gibco, cat.# 15070063) as described (Walther et al., 2024). mSIOs were passaged every week using Gentle Cell Dissociation Reagent (Stem Cell Technologies, cat.# 100-0485) with one to two media changes in between depending on mSIO density except for SOX9 spheroids which required at least two media changes due to their increased growth/proliferation rate.

Preparation of Wnt3a-conditioned medium

Wnt3a-conditioned medium was prepared as described before (Walther et al., 2024) using L-Wnt3a cells (CRL-2647, ATCC) and Advanced DMEM/F-12 (ADMEM; Gibco, cat.# 12634028) supplemented with 10% (vol/vol) FBS, 1% (vol/vol) GlutaMAX (Gibco, cat.# 35050061), 10 mM HEPES (Sigma-Aldrich, cat.# H0887-20mL), 1 mM N-acetyl-L-cysteine (Sigma-Aldrich, cat.# A9165-5G; 500 mM stock in ddH$_2$O, sterile-filtered).

Lentiviral transduction of mouse small intestinal organoids and selection of stable organoid lines

Lentiviral transduction of mSIOs was performed as described before (Walther et al., 2024). In brief, mSIOs were seeded in pre-transduction medium consisting of 50% (vol/vol) IntestiCult without Pen/Strep and 50% (vol/vol) Wnt3a-conditioned medium, supplemented with 100 mM nicotinamide (Sigma, cat.# N0636-100G; 1M stock in ddH$_2$O), 10 µM ROCK inhibitor Y-27632 (Stem Cell Technologies, cat.# 72304; 10 mM stock in ddH$_2$O), and 2.5 µM CHIR99021 (Stem Cell Technologies, cat.# 72052; 10 mM stock in dimethylsulfoxide (DMSO; Sigma-Aldrich, cat.# D2650)). 4-5 d post-seeding, mSIOs were broken down into single cells using TrypLE Express (Thermo Scientific, cat.# 12604013) prior to spinoculation (1 h at 37°C and 600 g) with lentivirus

in pre-transduction medium containing 10 µg/mL polybrene Millipore Sigma, cat.# TR-1003-G) and incubation for 6 h at 37°C. Transduced cells were seeded in 90% (vol/vol) Matrigel Matrix for Organoid Culture in IntestiCult Organoid Growth Medium supplemented with 1% (vol/vol) Pen/Strep and grown in pre-transduction medium. 2-3 d post-transduction, the medium was exchanged to pre-transduction medium containing selection antibiotics (2 µg/mL puromycin (Thermo Scientific, cat.# A11138-03)) and the selection medium was exchanged every 2-3 d until large, selected spheroids were obtained. Upon passaging, selected spheroids were cultured in IntestiCult supplemented with 10 µM ROCK inhibitor Y-27632 and 2.5 µM CHIR99021 including selection antibiotics. After 2-3 d the ROCK inhibitor was removed, followed by the removal of CHIR99021 after another 2-3 d to culture selected stable organoid lines as described before including selection antibiotics.

Established stable mSIO lines were cryopreserved in CryoStor CS10 (Stem Cell Technologies, cat.# 07931) as described before (Walther et al., 2024) and stored in liquid nitrogen.

Except for SOX9 spheroids and when specifically noted otherwise, mSIOs were used for experiments until 8 weeks after line establishment or an organoid aliquot cryopreserved at an early passage was thawn as described before (Walther et al., 2024).

For stable SOX9-Halo or SOX9-mEGFP organoids (WT background), a second transduction was performed at P15(P12) according to the same protocol to achieve double stable organoid lines co-expressing H2B-mEGFP or H2B-mScarletI, whereby antibiotic selection was performed using 40 µg/mL Zeocin (Fisher Scientific, cat.# NC9002627) or 200 µg/mL Geneticin (Thermo Fisher Scientific, cat.# 10131027; neomycin resistance), respectively.

Brightfield imaging of organoid lines and 2D enteroid monolayer cultures

Brightfield (BF) imaging of mSIOs or 2D EMCs growing on 24-well culture plates (Corning, cat.# 353047) or 8-well Labteks II #1.5 (Nunc, cat.# 12-565-338) to record 3D/2D morphology was performed on an EVOS M5000 Imaging System (Invitrogen) microscope using 10x (Fig. 3A) or 4x (Fig. 4A) air objectives 5 d post-seeding.

Confocal imaging of SOX9-Halo organoid lines

For confocal imaging of SOX9-Halo organoid lines, POI-Halo organoids were seeded in 10-20 µL 90% Matrigel in IntestiCult droplets into 8-well Labteks II #1.5 (Nunc, cat.# 12-565-338) and overlaid with 500 µL IntestiCult. After 3 d the medium was exchanged. 5 d post-seeding, organoids were stained with 500 nM HTL-JF635 (kind gift from Luke Lavis) in DMEM/F-12 without phenol red (Gibco, cat.# 11039-021) for at least 1 h and imaged 1-6 h after staining.

Confocal imaging of 3D mSIOs was performed on a Yokogawa CSU-W1 SoRa spinning disk with a Nikon Ti2 inverted microscope operated by the NIS Elements AR 5.42.03 software (Nikon). The microscope was equipped with a temperature- and CO_2-controlled incubation chamber (Okolab), and the temperature was set to 37°C and the CO_2 to 5% for imaging of live organoids. For combined BF/SOX9-Halo organoid images (Fig. 1C, Fig. S3A), acquisition was performed using an Apo LWD 40× WI λS DIC N2 water immersion objective (N.A. 1.15; Nikon). For imaging SOX9-Halo-JF635 and LGR5-DTR-GFP (budding; Fig. 1C) or H2B-mEGFP (spheroid; Fig. S3A), JF635 was excited with 640 nm (laser at 80% (budding) or 40% (spheroid)) and detected with Hamamatsu Orca Flash 4.0 cameras at 200 ms exposure using a 561 nm long-pass (LP) filter. Consecutively, the widefield fluorescence imaging modality was used for BF detection with a Hamamatsu Orca Flash 4.0 camera at 100 ms exposure. For each modality, multiple z planes (xy pixel size: 0.1625 × 0.1625 µm; z interval 0.4 µm, number of z slices varying depending on the thickness of organoids) were imaged using a NIDAQ z piezo. One selected z plane is shown in Fig. 1C and Fig. S3A.

Generation of 2D enteroid monolayer cultures from 3D mouse small intestinal organoids and seeding on imaging dishes

2D EMCs were derived from 3D mSIOs as described (Sanman et al., 2020; Walther et al., 2024). In brief, a 1:40 (vol:vol) mixture of Matrigel Growth Factor Reduced Basement Membrane Matrix (Corning, cat.# 356231) in DMEM/F-12 without phenol red was used for coating imaging dishes by incubation for at least 30 min up to one week in the cell culture incubator prior to seeding. 100-500 µL of $1x10^6$ cells/mL mSIO-derived single-cell suspensions were seeded in plating medium (IntestiCult containing 20 µM ROCK inhibitor and 3 µM CHIR) to achieve a total of 500 µL/well of an 8-well

Labtek II #1.5. 1-2 d after seeding and every other day thereafter the medium was exchanged to IntestiCult.

Confocal imaging of POI-Halo enteroid monolayer cultures

2D EMCs were seeded and cultured as described above. 5 d post-seeding, EMCs were stained with 200 nM HTL-JFX549 (kind gift from Luke Lavis) in DMEM/F-12 without phenol red for 30 min. After two washes with DMEM/F-12 without phenol red for 15 min each, the medium was exchanged one more time to DMEM/F-12 without phenol red before proceeding with imaging.

Confocal imaging of live 2D EMCs (Fig. 1C, Fig. S3A) was performed on an LSM900 Airyscan 2 laser-scanning microscope with an inverted Axio Observer.Z1 / 7 operated by the ZEN 3.1 blue software (ZEISS). The microscope was equipped with a temperature- and CO_2-controlled incubation chamber (Zeiss/PeCon), and the temperature was set to 37°C and the CO_2 to 5%. Images were acquired using a Plan-Apochromat 40×/N.A. 1.3 Oil DIC (UV) VIS-IR M27 oil-immersion objective (ZEISS). One *z* plane was imaged consecutively in the 561 nm and 488 nm channels (*xy* pixel size: 0.092 × 0.092 µm; 1.21 µs pixel dwell time; bidirectional scanning; 4-times averaging). JFX549 was excited with 561 nm (diode (SH) laser at 2.0%) and detected with GaAsP (spectral gallium arsenide) detectors at 566-635 nm. Green fluorescence was excited with 488 nm (diode laser at 4.5% for LGR5-DTR-GFP (Fig. 1C) or 0.1% for H2B-mEGFP (Fig. S3A)) and detected with a multialkali-photomultiplier (MA-PMT) detector at 410-545 nm.

Images acquired as described above were also used for SOX9-Halo transgene expression level quantification in SOX9 budding organoids *versus* spheroids (Fig. 3C, right).

For confocal imaging of rAAV-transduced conditions, 2D EMCs were prepared and stained as described under “Transient transduction of 2D enteroid monolayer cultures using crude rAAV vector preparations for SMT and PAPA-SMT” and imaged on a spinning disk confocal microscope described under “Confocal imaging of SOX9-Halo organoid lines” using an Apo LWD 40× WI λS DIC N2 water immersion objective (N.A. 1.15; Nikon). For imaging SOX9ΔHMG-Halo-JFX650 (Fig. S1D), JFX650 was excited with 640 nm (laser at 40%) and detected with a Hamamatsu Orca Flash 4.0 camera at 500 ms exposure using a 640 nm LP filter. For imaging SOX9-Halo-JFX549/SOX9-

SNAPf-JFX650 (Fig. 6C) and SOX9-Halo-JFX549/JFX650-SNAPf-3xNLS (Fig. 6D), JFX650 was excited with 640 nm (laser at 50%) and JFX549 was excited with 561 nm (laser at 50%). Fluorescence was detected with Hamamatsu Orca Flash 4.0 cameras at 300 ms exposure using a 640 nm LP filter. Multiple *z* planes (*xy* pixel size: 0.1625 × 0.1625 µm; 21 *z* slices at *z* interval 0.4 µm) were imaged using a NIDAQ *z* piezo. One selected *z* plane is shown in Fig. S1D and Fig. 6C,D.

For confocal imaging of cell division phenotypes in 2D EMCs derived from SOX9-Halo spheroids co-expressing H2B-mEGFP (Fig. S3B), EMCs were prepared as described above. Instead of SOX9-Halo, staining for microtubules was performed by adding 500 nM SiR-tubulin and 10 µM Verapamil (SiR-tubulin kit; Cytoskeleton, cat.# CY-SC002) in DMEM/F-12. After incubation at 37°C for 1 h, live imaging was performed on a spinning disk confocal microscope described under "Confocal imaging of SOX9-Halo organoid lines" using an Apo LWD 40× WI λS DIC N2 water immersion objective (N.A. 1.15; Nikon). SiR-tubulin was excited with 640 nm (laser at 50%) and H2B-mEGFP was excited with 488 nm (laser at 5%). Fluorescence was detected with Hamamatsu Orca Flash 4.0 cameras at 200 ms exposure using a 561 nm LP filter. Multiple *z* planes (*xy* pixel size: 0.1083 × 0.1083 µm; 37 *z* slices at *z* interval 0.3 µm) were imaged using a NIDAQ *z* piezo. One selected *z* plane is shown in Fig. S3B.

IF of 2D enteroid monolayer cultures and confocal imaging

IF of 2D EMCs and confocal imaging was performed 5 d post-seeding as described (Walther et al., 2024). In brief, EMCs were fixed with 4% paraformaldehyde (PFA; Electron Microscopy Sciences, cat.# EMS14710) in PBS for 20 min at RT, washed three times with PBS for 5 min and stored at 4°C or directly subjected to IF staining. Permeabilization was performed with 0.5% Triton X-100 (TX-100; Sigma-Aldrich, cat.# T9284) in PBS for 1 h. Following three washes for 5 min each with PBS, blocking was performed in 3% (vol/vol) donkey serum (Sigma-Aldrich, cat.# D9663) in 0.1% (vol/vol) TX-100 in PBS (blocking buffer) for 4 h at RT or ON at 4°C. Incubation with the primary antibody (AB) in blocking buffer was performed ON at 4°C in a humidified chamber. Following three washes in blocking buffer for 5 min each, incubation with the secondary AB was performed for 1 h at RT. Following three washes with PBS for 5 min each, DNA was stained with 1 µg/mL Hoechst 33342 (Thermo Scientific, cat.# H3570) in PBS for 10-15 min, followed by another three washes with PBS for 5 min

each. Immunostained samples were either imaged directly or sealed with parafilm and stored for short-term at 4°C prior to imaging.

The following primary ABs and dilutions were used: rabbit anti-SOX9 (Abcam, cat.# ab185966; 1:200), rabbit anti-KI67 (Abcam, cat.# ab16667; 1:200), rabbit anti-OLFM4 (Cell Signaling, cat.# 39141; 1:50), rabbit anti-Lysozyme (Agilent, cat.# A009902-2; 1:300), sheep anti-DLL1 (Biotechne, cat.# AF3970; 1:20), rabbit anti-Aldolase B/C (Abcam, cat.# ab75751; 1:50), rabbit anti-SCA-1 (Abcam ab124688, cat.# ab124688; 1:50), rabbit anti-YAP (Cell Signaling, cat.# 14074S; 1:100), rabbit anti-ß-catenin (Abcam, cat.# ab32572; 1:250), rabbit anti-E-Cadherin (Abcam, cat.# ab40772; 1:1000), rabbit anti-EpCAM (Thermo Fisher Scientific, cat.# MA5-35283; 1:100). A secondary donkey anti-rabbit-AlexaFluor568 AB (Thermo Fisher Scientific, cat.# A10042) was used for all conditions except for DLL1 for which a donkey anti-sheep-AlexaFluor568 AB (Thermo Fisher Scientific, cat. #A21099) was used. For F-actin staining, incubation with a 1x phalloidin-AlexaFluor568 (Thermo Fisher Scientific, cat.# A12380) staining solution in PBS was performed for 1 h at RT instead of incubation with primary/secondary ABs.

Confocal imaging of immunostained monolayers (Fig. 3B; Fig. 4E; Fig. S3C) was performed on an LSM900 Airyscan 2 laser-scanning microscope with an inverted Axio Observer.Z1 / 7 operated by the ZEN 3.1 blue software (ZEISS) at RT. Images were acquired using a Plan-Apochromat 40×/ N.A. 1.3 Oil DIC (UV) VIS-IR M27 oil-immersion objective (ZEISS). One *z* plane was imaged in the 561 nm and 405 nm channels (*xy* pixel size: 0.11 × 0.11 µm; 2.06 µs pixel dwell time; bidirectional scanning; 4-times averaging). AlexaFluor568 was excited with 561 nm (diode (SH) laser at 0.5-5.0% depending on the brightness of the immunostaining) and detected with MA-PMT detector at 576-700 nm. Hoechst was excited with 405 nm (diode laser at 0.2% except for Aldolase condition (0.5%)) and detected with MA-PMT detector at 410-493 nm. Laser excitation powers were kept constant across all samples per immunostaining condition.

Images immunostained for SOX9 and KI67 and acquired as described above were also used for quantifications of the total SOX9 expression level (Fig. 3C left) or the fraction of proliferative cells (Fig. 3E) in SOX9 spheroids *versus* WT organoids.

Image analysis of SOX9 immunostained 2D enteroid monolayer cultures to estimate total SOX9 expression levels

Hoechst-stained nuclei in confocal images of WT or Sox9_spheroid 2D EMCs immunostained for SOX9 (see "IF of 2D enteroid monolayer cultures and confocal imaging") were manually segmented using ImageJ (Schindelin et al., 2012). Following conversion of manual segmentation masks into Cellpose (Stringer et al., 2021)-compatible masks, mean SOX9 intensities were extracted using the Scikit-image library (Van Der Walt et al., 2014) and compared between WT and SOX9_spheroid conditions (Fig. 3C left). For the WT condition, cells were classified into SOX9-positive and SOX9-negative and analyzed separately to distinguish on-target from background fluorescence. Quantifications were based on 3 images with 456 cells (162 SOX9+, 294 SOX9-) for WT and 3 images with 159 cells for SOX9_spheroid from one IF experiment, whereby cells in each image were aggregated and the mean was calculated. Custom-written analysis code can be found on GitLab via the following link: https://gitlab.com/nikewalther/walther_sox9organoid_2025/-/tree/main/ConfocalAnalysis_Scripts/Sox9ExpressionLevelQuantification?ref_type=heads

Image analysis of HaloTag-stained live SOX9-Halo 2D enteroid monolayer cultures to estimate SOX9-Halo transgene expression levels

Nuclei in confocal images of WT or SOX9_spheroid live 2D EMCs (see "Confocal imaging of POI-Halo enteroid monolayer cultures") were manually segmented using ImageJ (Schindelin et al., 2012) based on the predominant nuclear localization of HTL-JFX549-labeled SOX9-Halo. Following conversion of manual segmentation masks into Cellpose (Stringer et al., 2021)-compatible masks, mean SOX9-Halo intensities were extracted using the Scikit-image library (Van Der Walt et al., 2014) and compared between SOX9_budding and SOX9_spheroid conditions (Fig. 3C right). Quantifications were based on 3 images with 864 cells for SOX9_budding and 3 images with 537 cells for SOX9_spheroid from one live-cell imaging experiment, whereby cells in each image were aggregated and the mean was calculated. Custom-written analysis code can be found on GitLab via the following link: https://gitlab.com/nikewalther/walther_sox9organoid_2025/-

/tree/main/ConfocalAnalysis_Scripts/Sox9ExpressionLevelQuantification?ref_type=heads

Image analysis of KI67-immunostained 2D enteroid monolayer cultures to quantify the fraction of proliferative cells

Hoechst-stained nuclei in confocal images of 2D EMCs immunostained for KI67 (see "IF of 2D enteroid monolayer cultures and confocal imaging") were manually segmented using ImageJ (Schindelin et al., 2012) and classified into KI67-positive and KI67-negative. Following conversion of manual segmentation masks into Cellpose (Stringer et al., 2021)-compatible masks, mean KI67 intensities were extracted using the Scikit-image library (Van Der Walt et al., 2014) and compared between WT and SOX9_spheroid conditions (Fig. 3E). Quantifications were based on 7 images with 752 cells for WT and 7 images with 648 cells for SOX9_spheroid from one IF experiment, whereby cells in each image were aggregated and the mean was calculated. Custom-written analysis code can be found on GitLab via the following link: https://gitlab.com/nikewalther/walther_sox9organoid_2025/-/tree/main/ConfocalAnalysis_Scripts/Ki67FrequencyDetermination?ref_type=heads

Image analysis of enteroid monolayer cultures to quantify cellular morphological features

To quantify the cellular morphological features nuclear area and nearest-nuclei-distance (Fig. 3D), WT and SOX9_spheroid confocal images of live or immunostained 2D EMCs were pooled, including the three experiments above (determination of total SOX9 expression level (IF), SOX9-Halo transgene expression level (live), and proliferation frequency (IF)). Based on the manually segmented and converted nuclear masks, the Scikit-image library (Van Der Walt et al., 2014) was used to compute the nuclear area and the mean distance from the centroid of a nucleus to the two nearest centroids of its neighboring nuclei (nearest-nuclei-distance). Quantifications were based on 23 images with 3029 cells for WT and 30 images with 3100 cells for SOX9_spheroid from a total of 4 experiments, whereby cells in each image were aggregated and the mean was calculated. Multinucleated cells were excluded from this analysis. Custom-written analysis code can be found on GitLab via the following link: https://gitlab.com/nikewalther/walther_sox9organoid_2025/-/tree/main/ConfocalAnalysis_Scripts/CellMorphologyQuantification?ref_type=heads

Classification and quantification of cell division errors in 2D enteroid monolayer cultures

To classify and quantify aberrant cell division phenotypes in WT, SOX9_budding, and SOX9_spheroid conditions, confocal images of live or immunostained 2D EMCs were used. The cell division phenotypes (a) two nuclei fused, (b) three nuclei fused, (c) micronuclei, and (d) multiple fragmented nuclei or micronuclei were classified according to the examples in Fig. S3B (left) and their occurrence per total number of cells per image was quantified. Quantifications were based on 13 images with 1821 cells for WT, 8 images with 1626 cells for SOX9_budding, and 17 images with 1756 cells for SOX9_spheroid from a total of 4 experiments, whereby cells in each image were aggregated and the mean was calculated. Not all conditions were included in each experiment.

Preparation of 2D enteroid monolayer cultures for SMT and PAPA-SMT

2D EMCs derived from stable SOX9-Halo mSIO lines were seeded and cultured as described above. 2-6 d post-seeding (depending on the degree of monolayer confluency and the formation of crypt- and villus-like morphological characteristics), monolayers were stained for SMT with 50 nM HTL-JFX549 (bulk labeling for nuclear segmentation and feature extraction; kind gift from Luke Lavis) and 1 nM HTL-JFX650 (sparse labeling for SMT; kind gift from Luke Lavis) in DMEM/F-12 without phenol red for 15 min. Following two washes with DMEM/F-12 without phenol red for 15 min each, the medium was replaced one more time to DMEM/F-12 without phenol red before proceeding with imaging.

Transient transduction of 2D enteroid monolayer cultures using crude rAAV vector preparations for SMT and PAPA-SMT

2D EMCs derived from WT mSIOs (for SMT of SOX9ΔHMG-Halo) or stable SOX9-Halo budding organoid or spheroid lines (for PAPA-SMT) were seeded and cultured as described above. 1-3 d post-seeding, the medium was replaced with a transduction mixture of 250 µL crude rAAV preparation and 250 µL plating medium. On the next day, the transduction mixture was removed. As best transgene expression was reached about 2 d post-transduction, IntestiCult w/o Pen/Strep and selection antibiotics was added for incubation for another day before staining for SMT with 50 nM HTL-JFX549 and 1 nM HTL-JFX650 in DMEM/F12 without phenol read for 15 min

at 37°C. For PAPA-SMT, staining with 50 nM HTL-JFX549 together with 50 nM STL-JFX650 (SOX9-SNAPf) or 5 nM STL-JFX650 (SNAPf-3xNLS) in IntestiCult w/o Pen/Strep and selection antibiotics was performed ON at 37°C before PAPA-SMT on the next day. Upon labeling for SMT or PAPA-SMT, two washes for 30 min each in DMEM/F12 without phenol red were performed prior to imaging in DMEM/F12 without phenol red.

Preparation of SOX9-Halo spheroids for SMT

For SMT of 3D spheroids, SOX9-Halo spheroids were seeded in 10-20µL droplets in 50% Matrigel in IntestiCult in 8-well Labteks II #1.5 and cultured as described before. Spheroids were grown until they were almost or even slightly touching the glass bottom of the culture dish, resulting in a restricted area within spheroids in which a layer of cells was oriented almost in parallel to the glass bottom. Such a configuration, required for successful HILO-based SMT, was typically reached 5 d post-seeding. Staining was performed with 50 nM HTL-JFX549 and 1-2.5 nM HTL-JFX650 in DMEM/F12 without phenol red for 1 h at 37°C. Following four washes in DMEM/F12 without phenol red for 30 min each and replacing the medium one more time with fresh DMEM/F12, SMT was performed.

TIRF microscope for HILO-based fast SMT and PAPA-SMT

All SMT and PAPA-SMT experiments were performed using a custom-built microscope as previously described (Hansen et al., 2017). In brief, a Nikon Ti microscope was equipped with a 100×/N.A. 1.49 oil-immersion TIRF objective (Nikon apochromat CFI Apo TIRF 100× Oil), a motorized mirror, a perfect focus system, an EM-CCD camera (Andor iXon Ultra 897), a laser launch with 405 nm (140 mW, OBIS, Coherent), 488 nm, 561 nm and 639 nm (1 W, Genesis Coherent) laser lines, and an incubation chamber maintaining a humidified atmosphere with 5% CO_2 at 37°C. The NIS-Elements software (Nikon) was used for controlling all microscope, camera and hardware components. Imaging was performed at laser power densities of approximately 52 W/cm^2 for 405 nm (violet), 91 W/cm^2 for 488 nm (blue), 100 W/cm^2 for 561 nm (green), and 2.3 kW/cm^2 for 639 nm (red).

Microscope automation for SMT and PAPA-SMT

A custom-built inverted Nikon Ti microscope described above was automated using code written in Python and the NIS Elements Macro Language as previously described (Dahal et al., 2025; Graham et al., 2025; Walther et al., 2024). In brief, the microscope stage rastered in a grid-like pattern to move the sample. At each grid position, a 512 px x 512 px (6710.9 μm^2) image was recorded in the densely labeled JFX549 channel or in the H2B-mEGFP channel for SOX9-Halo_spheroid. Segmentation of nuclei was performed using the pre-trained Versatile Fluorescent Nuclei model of the Python package StarDist (Schmidt et al., 2018). After randomly choosing one of the nuclei within a range of user-defined brightness and size parameters, the stage was moved to center it in the large FOV. The FOV was then resized to contain a smaller 150 px x 150 px (576 μm^2) square (small FOV; zoom-in) centered on the chosen nucleus. Images of the large and small FOVs were recorded in multiple channels corresponding to the fluorophores in the sample (GFP, JFX549, JFX650). JFX650 fluorophores in the small FOV were pre-bleached with red light at 639 nm to achieve sparse and thus trackable single molecules, following which an illumination sequence for fast SMT or PAPA-SMT was executed. On-the-fly localization density assessment was performed using quot (https://github.com/alecheckert/quot; (Heckert et al., 2022)). Microscope automation was based on our previously published code ((Walther et al., 2024); https://gitlab.com/tjian-darzacq-lab/walther_2dmsios_automatedsmt_2024/-/tree/main/Microscope_Automation_Scripts?ref_type=heads).

Fast SMT of POI-Halo in enteroid monolayer cultures

For imaging in GFP, JFX549 or JFX650 channels, laser powers were set to 100 mW for 488 nm, 110 mW for 561 nm, and to 1100 mW for 639 nm. The exposure time was set between 20 ms and 200 ms with a laser excitation power between 1.35% and 20% to achieve segmentable bulk labeling of POI-Halo depending on its expression level and to avoid saturation. Imaging conditions were kept constant across experiments for the same POI-Halo condition. Semrock 536/40 nm, 593/40 nm or 676/37 nm bandpass filters, respectively, were used.

For fast SMT experiments, the following bleaching durations in the 639 nm channel (100% laser excitation power) prior to executing an illumination sequence were used:

H2B – 15s, NLS – 10s, SOX9_budding – 15s or 10s, SOX9_spheroid – 15s, SOX9ΔHMG – 15s.

The triggered illumination sequence consisted of the following phases at a frame rate of 7.48 ms/frame:

1. Imaging: 5000 frames of red light (639 nm, one 2 ms stroboscopic pulse per frame) with 5% 405 nm reactivation (pulsed during the 0.48 ms camera transition time between 7 ms detection windows)

2. Imaging: 5000 frames of red light (639 nm, one 2 ms stroboscopic pulse per frame) with 10% 405 nm reactivation (pulsed during the 0.48 ms camera transition time between 7 ms detection windows)

To reduce the motion blur of moving molecules (Hansen et al., 2018), red illumination was restricted to stroboscopic pulses during each frame of a single-molecule movie.

Fast SMT of SOX9-Halo in SOX9-Halo spheroids

Fast SMT in 3D spheroids was performed in analogy to SMT in 2D EMCs with the exception that cells within spheroids that were close enough (10-20 μm) to the glass bottom of the cover slip to be reachable by HILO illumination were manually selected and centered within the FOV before triggering a sequence of epifluorescence imaging and SMT.

The following modified SMT illumination sequence was used:

1) As the bleaching efficiency in the 639 nm channel depended on the distance of the cell from the glass bottom of the culture dish, its duration had to be adjusted manually for each cell and was carried out until a trackable density of single-molecule localizations was achieved.

2) Embedding in Matrigel and the mostly larger distance of selected cells in 3D spheroids from the glass bottom of the culture dish in comparison to 2D EMCs required a higher 405 nm reactivation. The triggered illumination sequence thus consisted of the following phases at a frame rate of 7.48 ms/frame:

1. Imaging: 5000 frames of red light (639 nm, one 2 ms stroboscopic pulse per frame) with 0% 405 nm reactivation (primarily used to estimate background reactivation and non-specific fluorescence to judge suitability of selected cell for SMT)

2. Imaging: 5000 frames of red light (639 nm, one 2 ms stroboscopic pulse per frame) with 20% 405 nm reactivation (pulsed during the 0.48 ms camera transition time between 7 ms detection windows)

3. Imaging: 5000 frames of red light (639 nm, one 2 ms stroboscopic pulse per frame) with 100% 405 nm reactivation (pulsed during the 0.48 ms camera transition time between 7 ms detection windows)

PAPA-SMT of POI-Halo in enteroid monolayer cultures

Imaging for PAPA-SMT was performed as described for fast SMT.

For all PAPA-SMT conditions, an initial bleaching in the 639 nm channel (100% laser excitation power) was performed for 15 s prior to executing an illumination sequence.

For PAPA-SMT experiments, the illumination sequence consisted of 5 cycles of the following phases at a frame rate of 7.48 ms/frame:

1. Imaging: 250 frames of red light (639 nm), one 2 ms stroboscopic pulse per frame, recorded

2. DR pulse: 10 frames of violet light (405 nm), continuously during 7 ms detection window, recorded

3. DR imaging: 250 frames of red light, one 2 ms stroboscopic pulse per frame, recorded

4. Bleaching: 200 frames of red light, continuously during 7 ms detection window, not recorded

5. Imaging: 250 frames of red light, one 2 ms stroboscopic pulse per frame, recorded

6. PAPA pulse: 100 frames of green light (561 nm), continuously during 7 ms detection window, recorded

7. PAPA imaging: 250 frames of red light, one 2 ms stroboscopic pulse per frame, recorded

8. Bleaching: 200 frames of red light (639 nm), continuously during 7 ms detection window, not recorded

SMT and PAPA-SMT data processing and analysis

Image analysis for SMT and PAPA-SMT

Image analysis for SMT and PAPA-SMT was performed as previously described (Walther et al., 2024). In brief, nuclei in epifluorescence images corresponding to SMT or PAPA-SMT movies were segmented using StarDist (Schmidt et al., 2018). Segmented nuclei were subjected to manual QC for downstream analysis using CellPicker ((Walther et al., 2024); https://github.com/tgwgraham/basic_PAPASMT_analysis). Hereby, nuclei were filtered based on their intensities in each channel and a QC was performed to exclude nuclei with erroneous segmentation masks, unusual textures, or masks corresponding to segmented autofluorescent debris. In addition, CellPicker enabled the manual classification of cells based on the expression of the additionally recorded LGR5-DTR-GFP marker for stem/early progenitor cells. For each category, the cell morphological parameter nuclear area as well as the mean POI intensity in different channels as a proxy for relative POI expression level were computed for each of the chosen nuclei using the Python library Scikit-image (Van Der Walt et al., 2014) and the nuclear masks generated by StarDist. For nuclei in FOVs with three or more nuclei, the distance from the centroid of the nucleus of interest to the centroid of every other nucleus in the FOV was computed and the mean distance of the centroid of the nucleus of interest to its two nearest neighbors was recorded (nearest-nuclei-distance).

SMT data processing and analysis

SMT movies were processed using quot (https://github.com/alecheckert/quot; (Heckert et al., 2022)), which identifies single-molecule localizations and generates trajectories, as previously described (Walther et al., 2024). The following settings were used: [filter] start = 0; method = ‘identity’; chunk_size = 100; [detect] method = ‘llr’; k=1.0; w=9, t=18; [localize] method = ‘ls_int_gaussian’, window size = 9; sigma = 1.0; ridge = 0.001; max_iter = 10; damp = 0.3; camera_gain = 109.0; camera_bg = 470.0; [track] method = ‘conservative’; pixel_size_µm = 0.160; frame interval = 0.00748; search radius = 1; max_blinks = 0; min_IO = 0; scale = 7.0. To construct trajectories, a conservative method was used, in which only trajectories with unambiguously assigned localizations were considered. Tracking statistics are summarized in Fig. S9. Trajectories were assigned to cells using the StarDist-generated nuclear masks.

Diffusion coefficient distributions were obtained using the state array method of saSPT (https://github.com/alecheckert/saspt; (Heckert et al., 2022)). Here, a regular Brownian motion with a normally distributed, mean-zero localization error (RBME) model was fit to populations of single-molecule trajectories in each cell to obtain a state array, which consists of posterior occupations of states defined by their localization errors and diffusion coefficients. To obtain diffusion spectra, these state array distributions were marginalized on the diffusion coefficients. Mean diffusion spectra for each POI were constructed by averaging over single-cell diffusion spectra, whereby cells were weighted by the number of trajectories. The fraction of bound trajectories for a POI/condition was defined as the fraction of trajectories with diffusion coefficients below 0.15 $\mu m^2/s$ (fraction bound), as molecules diffusing at such slow rates are indistinguishable from H2B-Halo (Walther et al., 2024). As diffusion coefficient thresholds of both 0.1 and 0.15 $\mu m^2/s$ for the upper limit of trajectories accounted for in the bound fraction were used in the literature, we analyzed all fast SMT POI-Halo conditions acquired in this study with various thresholds ranging from 0.05 to 1 $\mu m^2/s$. This revealed no change in the rank order of conditions based on their fractions bound independent of the diffusion threshold used except for SOX9_budding_2D and SOX9_budding_3D with expected similar diffusion behavior (Fig. S10A). However, we note that – at least for large fraction bound threshold deviations from 0.15 $\mu m^2/s$ – this is partially due to all POIs measured in this study being characterized by distinct immobile and free diffusion peaks. Indeed, changes in the diffusive behavior between LGR5+/- SOX9_budding and SOX9_spheroid conditions occurred between these immobile and freely diffusing fractions (e.g. between bound and unbound states), whereas a slowly diffusing SOX9-Halo pool with intermediate mobility remained unchanged between LGR5+ and LGR5- conditions (Fig. S10B). Fractions of bound trajectories were also computed for individual nuclei based on the single-cell diffusion spectra to correlate them with extracted morphological features, such as nuclear area and nearest-nuclei-distance, as well as the POI expression level. To perform these processing and analysis steps, we used our previously published (Walther et al., 2024) custom-written Jupyter notebook for an all-in-one cell level-based diffusion analysis and correlation with extracted cellular features (https://gitlab.com/tjian-darzacq-lab/walther_2dmsios_automatedsmt_2024/-/tree/main/SMT_analysis_scripts?ref_type=heads).

For fast SMT data in SOX9-Halo spheroids, a filter was set to include only cells with at least 100 non-singlet trajectories for downstream analysis.

Cluster analysis based on single-cell diffusion spectra

Cluster-based analyses of single-cell diffusion spectra derived from fast SMT data were performed as previously described (Walther et al., 2024): The Jensen-Shannon distance as the distance metric (Nielsen, 2019) was used to compute a matrix of pairwise distances between pairs of single-cell diffusion spectra. Cells with complete linkage were then hierarchically clustered using the AgglomerativeClustering class of the Scikit-learn Python library (Pedregosa et al., 2011). The over- and under-representation of cells from different conditions in different clusters was quantified with a *p*-value computed from the hypergeometric distribution. To account for multiple comparisons, the significance threshold was Bonferroni-corrected. The option for diffusion-based cluster analysis is included in our previously published (Walther et al., 2024) custom-written Jupyter notebook (https://gitlab.com/tjian-darzacq-lab/walther_2dmsios_automatedsmt_2024/-/tree/main/SMT_analysis_scripts?ref_type=heads).

PAPA data processing and analysis

PAPA movies were processed and analyzed as described for SMT with the following specifications: All trajectory segments occurring within the first 30 frames after pulses of 561 nm light (PAPA trajectories) or 405 nm light (DR trajectories) were extracted using custom MATLAB code reported previously ((Graham et al., 2022; Walther et al., 2024); https://github.com/tgwgraham/basic_PAPASMT_analysis). PAPA and DR trajectories were then separately analyzed similar to SMT data. To distinguish undifferentiated from differentiated cells in SOX9_budding PAPA experiments, the whole cell population was split into small (proxy for stem and early progenitor cells) and large nuclei (proxy for late progenitor and differentiated cells) using a nuclear area of 75 μm^2 as threshold based on our previously established correlation between differentiation state and nuclear size (Walther et al., 2024). A custom-written Jupyter notebook is available here: https://gitlab.com/nikewalther/walther_sox9organoid_2025/-/tree/main/PAPA_analysis_scripts.

Bulk RNAseq

Organoid collection for RNAseq

For bulk RNAseq experiments in biological triplicates, mSIOs were used at the passages indicated in Fig. 4A plus/minus two passages. Organoids were seeded into 6 wells of a 24-well plate per line and cultured as described before. 5 d post-seeding, the medium was removed and organoids from the 6 wells were harvested with 1 mL TRIzol (Thermo Fisher Scientific, cat.# 15596026) into low-binding 1.7 mL tubes (Sorenson, cat.# 39640T) by rigorously pipetting up and down with a 1 mL pipette to dislodge and homogenize the organoid-containing Matrigel domes. Organoid-TRIzol samples were directly stored at -20°C until RNA extraction.

RNA extraction and poly-A RNAseq library preparation

Poly-A RNAseq was performed in three biological replicates per condition. Total RNA was extracted with TRIzol according to the manufacturer's instructions by performing the optional centrifugation step of the lysates (5 min at 12,000 g at 4–10°C) and an additional wash with one volume of chloroform after the recommended phenol:chloroform extraction (UltraPure Phenol:Chloroform:Isoamyl Alcohol, 25:24:1, v/v, cat.# 15593-01). RNA was quantified by spectrophotometer (NanoDrop, ThermoFisher Scientific) and checked for integrity by capillary electrophoresis (Fragment Analyzer, Agilent). 100-500 ng of total RNA were subjected to poly-A purification and library preparation with the NEBNext Poly(A) mRNA Magnetic Isolation Module (NEB, cat.# E7490S) in combination with the NEBNext Ultra II RNA Library Prep Kit for Illumina (NEB, cat.# E7770S). The NEBNext Adaptor for Illumina was diluted 1:5 (for 500 ng input RNA) or 1:25 (for 100 ng input RNA) in Tris/NaCl, pH 8.0 (10 mM Tris-HCl pH 8.0, 10 mM NaCl) and the ligation step was extended to 30 min. Libraries were enriched with 9-11 PCR cycles with the NEBNext Multiplex Oligos for Illumina (Dual Index Primers Set 1; NEB, cat.# E7600S). Library concentration was assessed by Qubit quantification (Qubit dsDNA HS Assay Kit; Invitrogen, cat.# Q32851). Multiplexed libraries were pooled and sequenced on the Illumina NovaSeq X Plus platform (150 bp, paired end reads) by MedGenome Inc. (Foster City, CA, USA).

RNAseq analysis

RNAseq raw reads were quality checked with FastQC (http://www.bioinformatics.babraham.ac.uk/projects/fastqc), trimmed with cutadapt (DOI: https://doi.org/10.14806/ej.17.1.200; version 4.5 with Python 3.10.13) and aligned onto the mouse genome (mm39) using STAR RNA-Seq aligner (Dobin et al., 2013) with the following options: --outSJfilterReads: Unique, --outFilterMultimapNmax: 1, --outFilterIntronMotifs: RemoveNoncanonical, --outSAMstrandField: intronMotif. Samtools (Li et al., 2009) (version 1.9) was used to convert STAR output .sam files into .bam files, and to sort and index them. After counting how many reads overlapped an annotated gene (Ensembl GRCm39 annotations) using HTSeq (Anders et al., 2015) (options: --htseq-count; --stranded=no -f bam; --additional-attr=gene_name -m union), the output counts files were used to find DEGs with DESeq2 (Love et al., 2014), run with default parameters within the Galaxy platform (Blankenberg et al., 2010; Giardine et al., 2005; Goecks et al., 2010). DEGs were called using an adjusted *p*-value ≤0.01, a fold change ≥2 and ≥10 mean counts. Gene transcript levels were visualized on the mm39 genome with the Integrative Genomics Viewer (IGV) (Robinson et al., 2011; Thorvaldsdóttir et al., 2013) using the bigWig output files from deepTools' bamCoverage (Ramírez et al., 2014) (version: 3.5.1; options: --binSize: 50; --extendReads: 250; --normalizeUsing: BPM; --samFlagInclude: 64). PCA and sample-to-sample distance analysis were part of the DESeq2 output.

Based on the DESeq2 output, pairwise comparison of conditions was performed by visualizing DEGs as called above via the EnhancedVolcano package (https://github.com/kevinblighe/EnhancedVolcano) within Bioconductor (Huber et al., 2015) (release 3.20) using R (version 4.4.2) and indicating the number of up- or downregulated genes. In addition, DEGs of pairwise comparisons were further analyzed with respect to their enrichment in biological pathways by GO analysis using the clusterProfiler (Yu, 2024) and AnnotationDbi (https://bioconductor.org/packages/AnnotationDbi) packages and selecting the ontology class BP for Biological processes.

Using the ComplexHeatmap package (Gu, 2022; Gu et al., 2016), a comparison of DEGs in all five conditions was visualized in a heatmap displaying *z*-scores, whereby either all DEGs (Fig. 4C) or selected DEGs (Fig. 4D) were plotted. In addition, CPMs

of some of these selected DEGs were plotted across all five conditions (Fig. S5; barplot: mean of triplicates; CPM values of individual replicates as points).

Quantification and statistical analysis

The significance of the differences in the distribution of SOX9 expression levels (Fig. 3C), the distributions in cellular morphological features (Fig. 3D), as well as the percentage of proliferating KI67-positive cells (Fig. 3E) and aberrant cell division phenotypes (Fig. S3B) was computed using a Mann-Whitney U test after aggregating all cells in one image and calculating a mean for each image. For SOX9 expression level analyses (Fig. 3B), alternative hypotheses in which cells in the SOX9 spheroid condition had stochastically greater metric values were used. For cellular morphologies (Fig. 3D), proliferation (Fig. 3E), and cell division phenotypes (Fig. S3B), a two-sided alternative hypothesis was used (Fig. 4D). *p*-values indicated in Fig. 3C-E and Fig. S3B were rated as follows: (ns) non-significant, $p>0.05$; (*) $p\leq0.05$; (**) $p\leq0.01$; (***) $p\leq0.001$; (****) $p\leq0.0001$. The exact *p*-values for pairwise comparisons in Fig. 3B-E are as follows: Fig. 3C left: $p=0.05$; Fig. 3C right: $p=0.35$; Fig. 3D left: $p=7.2e-5$; Fig. 3D right: $p=7.2e-5$; Fig. 3E: $p=0.01$. The exact *p*-values for WT *vs.* SOX9_budding, WT *vs.* SOX9_spheroid, and SOX9_budding *vs.* SOX9_spheroid in Fig. S3B are as follows: Fig. S3B top left (a): 1.00, 1.95e-3, 3.07e-3; Fig. S3B top right (b): 5.55e-1, 4.91e-2, 2.06e-2; Fig. S3B bottom left (c): 7.84e-2, 1.01e-5, 6.70e-3; Fig. S3B bottom right (d): 7.57e-2, 1.11e-2, 2.35e-1.

For SMT experiments, bootstrapping on all combined experiments per fast SMT condition was performed by drawing 1000 samples from the population with replacement, whereby each sample contained as many nuclei as the total number of nuclei in the population under analysis. For each bootstrap replicate, diffusion spectra marginalized on the diffusion coefficients were generated and used to compute CIs for the fraction of bound trajectories. Code for SMT bootstrap analysis is included in our previously published (Walther et al., 2024) custom-written Jupyter notebook (https://gitlab.com/tjian-darzacq-lab/walther_2dmsios_automatedsmt_2024/-/tree/main/SMT_analysis_scripts?ref_type=heads).

For determining the significance of the difference in the fraction bound distributions between two conditions based on the single-cell fractions bound, Mann-Whitney U

tests were performed. The Benjamini-Hochberg procedure was used to correct for multiple comparisons. *p*-values were rated as follows: (ns) non-significant, $p>0.05$; (*) $p\leq0.05$; (**) $p\leq0.01$; (***) $p\leq0.001$; (****) $p\leq0.0001$. Statistics of fraction bound comparisons between conditions are summarized in Fig. S8C.

For determining a potential correlation between the relative POI expression level and the fraction bound in SMT experiments (Fig. 2D, Fig. 5D; Fig. S1G,H), nuclear SOX9 intensities were extracted from StarDist (Schmidt et al., 2018) masks and plotted against the fraction bound of the POI in each nucleus inferred using saSPT's (Heckert et al., 2022) State Array Dataset class for each POI. For each POI, a potential correlation between the fraction bound and the relative SOX9 expression level was calculated by performing a linear least-squares regression using the SciPy Python library (Virtanen et al., 2020), which yielded an *R*-value (Pearson correlation coefficient). *p*-values were computed using a Wald test with a t-distribution test statistic and a two-sided alternative hypothesis. Code is included in our previously published (Walther et al., 2024) custom-written Jupyter notebook (https://gitlab.com/tjian-darzacq-lab/walther_2dmsios_automatedsmt_2024/-/tree/main/SMT_analysis_scripts?ref_type=heads).

The corresponding figure legends contain statistical details for SMT experiments and fraction bound/intensity correlations mentioned above.

For PAPA-SMT experiments, statistical analyses were performed as described before using custom-written MATLAB scripts ((Dahal et al., 2025; Walther et al., 2024); https://github.com/tgwgraham/basic_PAPASMT_analysis). For a side-by-side comparison of the distributions for DR and PAPA trajectories, they were randomly subsampled without replacement for the condition with more trajectories. Following subsampling, bootstrapping analysis with replacement was performed on all combined experiments per condition for the PAPA datasets for SOX9_spheroid/SOX9, SOX9_spheroid/NLS, SOX9_budding/SOX9, and SOX9_budding/NLS. For each combined dataset, a random sample of size *n*, where *n* is the total number of cells in the combined dataset, was drawn 100 times. We reported the mean and standard deviation from these analyses (Fig. 6G,H; Fig. S7). For significance testing between DR and PAPA conditions, two-tailed *p*-values were calculated based on a normal distribution (SciPy function (Virtanen et al., 2020), scipy.stats.norm.sf) with mean

equal to the difference between sample means and variance equal to the sum of the variances from the bootstrap resampling. The statistical details for the PAPA experiments shown in Fig. 6E-H are as follows: SOX9→SOX9: Subsampling of 9255 trajectories determined fractions bound to 40.3% (DR) and 52.0% (PAPA). Bootstrap resampling with 100 replicates determined fractions bound to 40.6±2.9% (DR) and 51.9±3.1% (PAPA); 2-sided *p*-value: 2.3e-7. SOX9→NLS: Subsampling of 4117 trajectories determined fractions bound to 26.4% (DR) and 26.1% (PAPA). Bootstrap resampling with 100 replicates determined fractions bound to 27.2±2.5% (DR) and 26.1±2.8% (PAPA); 2-sided *p*-value: 1.4. The statistical details for the PAPA experiments shown in Fig. S7A,B are as follows: SOX9→SOX9: Subsampling of 1191 trajectories determined fractions bound to 38.2% (DR) and 46.2% (PAPA). Bootstrap resampling with 100 replicates determined fractions bound to 38.9±4.9% (DR) and 46.0±6.6% (PAPA); 2-sided *p*-value: 0.09. SOX9→NLS: Subsampling of 2644 trajectories determined fractions bound to 16.8% (DR) and 16.3% (PAPA). Bootstrap resampling with 100 replicates determined fractions bound to 17.6±2.5% (DR) and 16.4±2.8% (PAPA); 2-sided *p*-value: 1.44. For Fig. S7E-H, bootstrapped fractions bound are as follows: (E) – 40.5±5.9% (DR) and 48.6±7.9% (PAPA); (F) – 21.3±5.1% (DR) and 17.6±5.2% (PAPA); (G) – 35.0±7.0% (DR) and 42.3±9.1% (PAPA); (H) – 14.8±3.2% (DR) and 15.0±2.8% (PAPA). The corresponding 2-sided *p*-values are as follows: (E) 0.111; (F) 1.679; (G) 0.215; (H) 0.923. *p*-values indicated in Fig. 6G,H and Fig. S7 were rated as follows: (ns) non-significant, $p>0.05$; (*) $p\leq0.05$; (**) $p\leq0.01$; (***) $p\leq0.001$; (****) $p\leq0.0001$.

Supplemental references

Anders, S., Pyl, P.T., and Huber, W. (2015). HTSeq-A Python framework to work with high-throughput sequencing data. Bioinformatics *31*, 166–169. https://doi.org/10.1093/bioinformatics/btu638.

Benyamini, B., Esbin, M.N., Whitney, O., Walther, N., and Maurer, A.C. (2023). Transgene Expression in Cultured Cells Using Unpurified Recombinant Adeno-Associated Viral Vectors. J. Vis. Exp. *2023*, 1–22. https://doi.org/10.3791/65572.

Blankenberg, D., Kuster, G. Von, Coraor, N., Ananda, G., Lazarus, R., Mangan, M., Nekrutenko, A., and Taylor, J. (2010). Galaxy: A web-based genome analysis tool for experimentalists. Curr. Protoc. Mol. Biol. 1–21. https://doi.org/10.1002/0471142727.mb1910s89.

Dahal, L., Graham, T.G.W., Dailey, G.M., Heckert, A., Tjian, R., and Darzacq, X. (2025). Surprising features of nuclear receptor interaction networks revealed by live-cell single-molecule imaging. Elife *12*, RP92979. https://doi.org/10.7554/eLife.92979.

Dobin, A., Davis, C.A., Schlesinger, F., Drenkow, J., Zaleski, C., Jha, S., Batut, P., Chaisson, M., and Gingeras, T.R. (2013). STAR: Ultrafast universal RNA-seq aligner. Bioinformatics *29*, 15–21. https://doi.org/10.1093/bioinformatics/bts635.

Giardine, B., Riemer, C., Hardison, R.C., Burhans, R., Elnitski, L., Shah, P., Zhang, Y., Blankenberg, D., Albert, I., Taylor, J., et al. (2005). Galaxy: A platform for interactive large-scale genome analysis. Genome Res. *15*, 1451–1455. https://doi.org/10.1101/gr.4086505.

Goecks, J., Nekrutenko, A., Taylor, J., Afgan, E., Ananda, G., Baker, D., Blankenberg, D., Chakrabarty, R., Coraor, N., Von Kuster, G., et al. (2010). Galaxy: a comprehensive approach for supporting accessible, reproducible, and transparent computational research in the life sciences. Genome Biol. *11*. https://doi.org/10.1186/gb-2010-11-8-r86.

Götzke, H., Kilisch, M., Martínez-Carranza, M., Sograte-Idrissi, S., Rajavel, A., Schlichthaerle, T., Engels, N., Jungmann, R., Stenmark, P., Opazo, F., et al. (2019). The ALFA-tag is a highly versatile tool for nanobody-based bioscience applications. Nat. Commun. *10*, 1–12. https://doi.org/10.1038/s41467-019-12301-7.

Graham, T.G.W., Ferrie, J.J., Dailey, G.M., Tjian, R., and Darzacq, X. (2022). Detecting molecular interactions in live-cell single-molecule imaging with proximity-assisted photoactivation (PAPA). Elife *11*, 1–46. https://doi.org/10.7554/eLife.76870.

Graham, T.G.W., Dugast-Darzacq, C., Dailey, G.M., Weng, B., Anantakrishnan, S., Darzacq, X., and Tjian, R. (2025). Single-molecule live imaging of subunit interactions and exchange within cellular regulatory complexes. Mol. Cell *85*, 2854-2868.e7. https://doi.org/https://doi.org/10.1016/j.molcel.2025.06.028.

Gu, Z. (2022). Complex heatmap visualization. IMeta *1*, 1–15. https://doi.org/10.1002/imt2.43.

Gu, Z., Eils, R., and Schlesner, M. (2016). Complex heatmaps reveal patterns and correlations in multidimensional genomic data. Bioinformatics *32*, 2847–2849. https://doi.org/10.1093/bioinformatics/btw313.

Hansen, A.S., Pustova, I., Cattoglio, C., Tjian, R., and Darzacq, X. (2017). CTCF and cohesin regulate chromatin loop stability with distinct dynamics. Elife *6*, 1–33. https://doi.org/10.7554/eLife.25776.

Hansen, A.S., Woringer, M., Grimm, J.B., Lavis, L.D., Tjian, R., and Darzacq, X. (2018). Robust model-based analysis of single-particle tracking experiments with Spot-On. Elife *7*, e33125. https://doi.org/10.7554/eLife.33125.

Heckert, A., Dahal, L., Tijan, R., and Darzacq, X. (2022). Recovering mixtures of fast-diffusing states from short single-particle trajectories. Elife *11*, 1–32. https://doi.org/10.7554/ELIFE.70169.

Huber, W., Carey, V.J., Gentleman, R., Anders, S., Carlson, M., Carvalho, B.S., Bravo, H.C., Davis, S., Gatto, L., Girke, T., et al. (2015). Orchestrating high-throughput genomic analysis with Bioconductor. Nat. Methods *12*, 115–121. https://doi.org/10.1038/nmeth.3252.

Li, H., Handsaker, B., Wysoker, A., Fennell, T., Ruan, J., Homer, N., Marth, G., Abecasis, G., and Durbin, R. (2009). The Sequence Alignment/Map format and SAMtools. Bioinformatics *25*, 2078–2079. https://doi.org/10.1093/bioinformatics/btp352.

Love, M.I., Huber, W., and Anders, S. (2014). Moderated estimation of fold change and dispersion for RNA-seq data with DESeq2. Genome Biol. *15*, 1–21. https://doi.org/10.1186/s13059-014-0550-8.

Murphy, G.J., Mostoslavsky, G., Kotton, D.N., and Mulligan, R.C. (2006). Exogenous control of mammalian gene expression via modulation of translational termination. Nat. Med. *12*, 1093–1099. https://doi.org/10.1038/nm1376.

Nielsen, F. (2019). On the Jensen-Shannon symmetrization of distances relying on abstract means. Entropy *21*, 1–23. https://doi.org/10.3390/e21050485.

Pedregosa, F., Varoquaux, G., Gramfort, A., Michel, V., Thirion, B., Grisel, O., Blondel, M., Prettenhofer, P., Weiss, R., Dubourg, V., et al. (2011). Scikit-learn: Machine Learning in Python. J. Mach. Learn. Res. *12*, 2825–2830. https://doi.org/10.1289/EHP4713.

Pekrun, K., De Alencastro, G., Luo, Q.J., Liu, J., Kim, Y., Nygaard, S., Galivo, F., Zhang, F., Song, R., Tiffany, M.R., et al. (2019). Using a barcoded AAV capsid library to select for clinically relevant gene therapy vectors. JCI Insight *4*. https://doi.org/10.1172/jci.insight.131610.

Ramírez, F., Dündar, F., Diehl, S., Grüning, B.A., and Manke, T. (2014). DeepTools: A flexible platform for exploring deep-sequencing data. Nucleic Acids Res. *42*, 187–191. https://doi.org/10.1093/nar/gku365.

Robinson, J.T., Thorvaldsdóttir, H., Winckler, W., Guttman, M., Lander, E.S., Getz, G., and Mesirov, J.P. (2011). Integrative genomics viewer. Nat. Biotechnol. *29*, 24–26. https://doi.org/10.1038/nbt.1754.

Sanman, L.E., Chen, I.W., Bieber, J.M., Thorne, C.A., Wu, L.F., and Altschuler, S.J. (2020). Chapter 6. Methods Mol. Biol. *2171*, 99–113. .

Schindelin, J., Arganda-Carreras, I., Frise, E., Kaynig, V., Longair, M., Pietzsch, T., Preibisch, S., Rueden, C., Saalfeld, S., Schmid, B., et al. (2012). Fiji: An open-source

platform for biological-image analysis. Nat. Methods *9*, 676–682. https://doi.org/10.1038/nmeth.2019.

Schmidt, U., Weigert, M., Broaddus, C., and Myers, G. (2018). Cell detection with star-convex polygons (Springer International Publishing).

Stringer, C., Wang, T., Michaelos, M., and Pachitariu, M. (2021). Cellpose: a generalist algorithm for cellular segmentation. Nat. Methods *18*, 100–106. https://doi.org/10.1038/s41592-020-01018-x.

Thorvaldsdóttir, H., Robinson, J.T., and Mesirov, J.P. (2013). Integrative Genomics Viewer (IGV): High-performance genomics data visualization and exploration. Brief. Bioinform. *14*, 178–192. https://doi.org/10.1093/bib/bbs017.

Virtanen, P., Gommers, R., Oliphant, T.E., Haberland, M., Reddy, T., Cournapeau, D., Burovski, E., Peterson, P., Weckesser, W., Bright, J., et al. (2020). SciPy 1.0: fundamental algorithms for scientific computing in Python. Nat. Methods *17*, 261–272. https://doi.org/10.1038/s41592-019-0686-2.

Van Der Walt, S., Schönberger, J.L., Nunez-Iglesias, J., Boulogne, F., Warner, J.D., Yager, N., Gouillart, E., and Yu, T. (2014). Scikit-image: Image processing in python. PeerJ *2014*, 1–18. https://doi.org/10.7717/peerj.453.

Walther, N., Anantakrishnan, S., Graham, T.G.W., Dailey, G.M., and Tjian, R. (2024). Automated live-cell single-molecule tracking in enteroid monolayers reveals transcription factor dynamics probing lineage-determining function. Cell Rep. *43*, 114914. https://doi.org/10.1016/j.celrep.2024.114914.

Yu, G. (2024). Thirteen years of clusterProfiler. Innovation *5*, 5–6. https://doi.org/10.1016/j.xinn.2024.100722.
